# Association of Radiotherapy Duration With Clinical Outcomes in Patients With Esophageal Cancer Treated in NRG Oncology Trials: A Secondary Analysis of NRG Oncology Randomized Clinical Trials

**DOI:** 10.1001/jamanetworkopen.2023.8504

**Published:** 2023-04-21

**Authors:** Christopher L. Hallemeier, Jennifer Moughan, Michael G. Haddock, Arnold M. Herskovic, Bruce D. Minsky, Mohan Suntharalingam, Kenneth L. Zeitzer, Madhur K. Garg, Bruce D. Greenwald, Ritsuko U. Komaki, Lindsay L. Puckett, Hyun Kim, Shane Lloyd, David A. Bush, Harold E. Kim, Thomas E. Lad, Joshua E. Meyer, Gordon S. Okawara, Adam Raben, Tracey E. Schefter, Jerry L. Barker, Carla I. Falkson, Gregory M. M. Videtic, Rojymon Jacob, Kathryn A. Winter, Christopher H. Crane

**Affiliations:** 1 Department of Radiation Oncology, Mayo Clinic, Rochester, Minnesota; 2 NRG Oncology Statistics and Data Management Center/American College of Radiology, Philadelphia, Pennsylvania; 3 Department of Radiation Oncology, Rush University Medical Center, Chicago, Illinois; 4 Department of Radiation Oncology, The University of Texas, MD Anderson Cancer Center, Houston; 5 Department of Radiation Oncology, University of Maryland and Greenebaum Comprehensive Cancer Center, Baltimore; 6 Department of Radiation Oncology, Albert Einstein Medical Center, Philadelphia, Pennsylvania; 7 Department of Radiation Oncology, Montefiore Medical Center–Moses Campus, Bronx, New York; 8 Department of Gastroenterology and Hepatology, University of Maryland and Greenebaum Cancer Center, Baltimore; 9 Department of Radiation Oncology, Medical College of Wisconsin and Zablocki Veterans' Administration Medical Center, Milwaukee; 10 Department of Radiation Oncology, Washington University School of Medicine in St Louis, St Louis, Missouri; 11 Department of Radiation Oncology, University of Utah Health Science Center, Salt Lake City; 12 Department of Radiation Oncology, Loma Linda University Cancer Institute, Loma Linda, California; 13 Department of Radiation Oncology, Wayne State University/Karmanos Cancer Institute, Detroit, Michigan; 14 Department of Medical Oncology, John H. Stroger Jr Hospital of Cook County, Chicago, Illinois; 15 Department of Radiation Oncology, Fox Chase Cancer Center, Philadelphia, Pennsylvania; 16 Department of Radiation Oncology, McMaster University, Juravinski Cancer Centre, Hamilton, Ontario, Canada; 17 Department of Radiation Oncology, Christiana Care Health Services Inc Community Clinical Oncology Program, Newark, Delaware; 18 Department of Radiation Oncology, University of Colorado, Aurora; 19 Department of Radiation Oncology, US Oncology Texas Oncology-Sugar Land, Fort Worth; 20 Department of Medicine, Hematology/Oncology, University of Rochester, Rochester, New York; 21 Department of Radiation Oncology, Cleveland Clinic Foundation, Cleveland, Ohio; 22 Department of Radiation Oncology, University of Alabama at Birmingham, Birmingham; 23 Department of Radiation Oncology, Memorial Sloan Kettering Cancer Center, New York, New York

## Abstract

**Importance:**

For many types of epithelial malignant neoplasms that are treated with definitive radiotherapy (RT), treatment prolongation and interruptions have an adverse effect on outcomes.

**Objective:**

To analyze the association between RT duration and outcomes in patients with esophageal cancer who were treated with definitive chemoradiotherapy (CRT).

**Design, Setting, and Participants:**

This study was an unplanned, post hoc secondary analysis of 3 prospective, multi-institutional phase 3 randomized clinical trials (Radiation Therapy Oncology Group [RTOG] 8501, RTOG 9405, and RTOG 0436) of the National Cancer Institute–sponsored NRG Oncology (formerly the National Surgical Adjuvant Breast and Bowel Project, RTOG, and Gynecologic Oncology Group). Enrolled patients with nonmetastatic esophageal cancer underwent definitive CRT in the trials between 1986 and 2013, with follow-up occurring through 2014. Data analyses were conducted between March 2022 to February 2023.

**Exposures:**

Treatment groups in the trials used standard-dose RT (50 Gy) and concurrent chemotherapy.

**Main Outcomes and Measures:**

The outcomes were local-regional failure (LRF), distant failure, disease-free survival (DFS), and overall survival (OS). Multivariable models were used to examine the associations between these outcomes and both RT duration and interruptions. Radiotherapy duration was analyzed as a dichotomized variable using an X-Tile software to choose a cut point and its median value as a cut point, as well as a continuous variable.

**Results:**

The analysis included 509 patients (median [IQR] age, 64 [57-70] years; 418 males [82%]; and 376 White individuals [74%]). The median (IQR) follow-up was 4.01 (2.93-4.92) years for surviving patients. The median cut point of RT duration was 39 days or less in 271 patients (53%) vs more than 39 days in 238 patients (47%), and the X-Tile software cut point was 45 days or less in 446 patients (88%) vs more than 45 days in 63 patients (12%). Radiotherapy interruptions occurred in 207 patients (41%). Female (vs male) sex and other (vs White) race and ethnicity were associated with longer RT duration and RT interruptions. In the multivariable models, RT duration longer than 45 days was associated with inferior DFS (hazard ratio [HR], 1.34; 95% CI, 1.01-1.77; *P* = .04). The HR for OS was 1.33, but the results were not statistically significant (95% CI, 0.99-1.77; *P* = .05). Radiotherapy duration longer than 39 days (vs ≤39 days) was associated with a higher risk of LRF (HR, 1.32; 95% CI, 1.06-1.65; *P* = .01). As a continuous variable, RT duration (per 1 week increase) was associated with DFS failure (HR, 1.14; 95% CI, 1.01-1.28; *P* = .03). The HR for LRF 1.13, but the result was not statistically significant (95% CI, 0.99-1.28; *P* = .07).

**Conclusions and Relevance:**

Results of this study indicated that in patients with esophageal cancer receiving definitive CRT, prolonged RT duration was associated with inferior outcomes; female patients and those with other (vs White) race and ethnicity were more likely to have longer RT duration and experience RT interruptions. Radiotherapy interruptions should be minimized to optimize outcomes.

## Key Points

**Question:**

Is there an association between radiotherapy (RT) duration and clinical outcomes in patients with esophageal cancer receiving definitive chemoradiotherapy?

**Findings:**

In this secondary analysis of 3 NRG Oncology randomized clinical trials, prolonged RT duration was associated with inferior disease-free survival. Female patients and those with other (vs White) race and ethnicity were more likely to have prolonged RT duration and to experience RT interruptions.

**Meaning:**

Findings of this study suggest that RT interruptions should be minimized to optimize outcomes.

## Teaser

This secondary analysis examines the local-regional failure, distant failure, disease-free survival, and overall survival in patients with esophageal cancer who underwent definitive chemoradiotherapy during NRG Oncology randomized clinical trials.

## Introduction

Radiotherapy (RT) and chemoradiotherapy (CRT) are used for the curative treatment of many epithelial malignant neoplasms. Treatment-related toxic effects may lead to treatment interruptions and prolonged treatment duration. In the curative treatment of head and neck, lung, cervical, and anal cancer with RT and/or CRT, treatment interruptions and prolonged treatment duration have been associated with inferior tumor local control and/or survival.^1,2,3,4,5,6,7,8,9^ Additionally, meta-analyses of randomized clinical trials in head and neck cancer and lung cancer have demonstrated that accelerated RT regimens that reduce the RT duration are associated with improved tumor control and survival compared with conventional fractionation RT regimens.^10,11^ It is hypothesized that these observations are related to the accelerated repopulation of tumor clonogen after the initiation of treatment.^1^

In patients with esophageal cancer who were treated with definitive CRT, the effect of RT treatment interruptions and prolonged RT duration on local control and survival has not been well studied. This topic is of elevated relevance and importance in the context of the COVID-19 pandemic, which may force difficult decisions regarding potential disruptions in delivery of RT for patients with esophageal cancer.^12^ The purpose of this study was to analyze the association between RT duration and outcomes in patients with esophageal cancer who were treated with definitive CRT in trials of the National Cancer Institute–sponsored NRG Oncology (formerly known as the National Surgical Adjuvant Breast and Bowel Project, Radiation Therapy Oncology Group [RTOG], and Gynecologic Oncology Group^13,14,15,16,17^). It was hypothesized that RT interruptions and prolonged RT duration would be associated with inferior outcomes.

## Methods

### Patient Cohorts and Treatment

This unplanned, post hoc secondary analysis included evaluable patients with nonmetastatic esophageal cancer who underwent definitive CRT in the RTOG 8501, RTOG 9405 (NCT00002631), and RTOG 0436 (NCT00655876) randomized clinical trials, with follow-up through 2014 (Figure).^13,14,15,16^ For each of the 3 NRG Oncology trials, patients provided written informed consent. The protocol, protocol amendments, and informed consent documents were approved by the institutional review board or ethics committee at each trial site (Supplements 1-3). We followed the Consolidated Standards of Reporting Trials (CONSORT) reporting guideline, where applicable.

**Figure.**
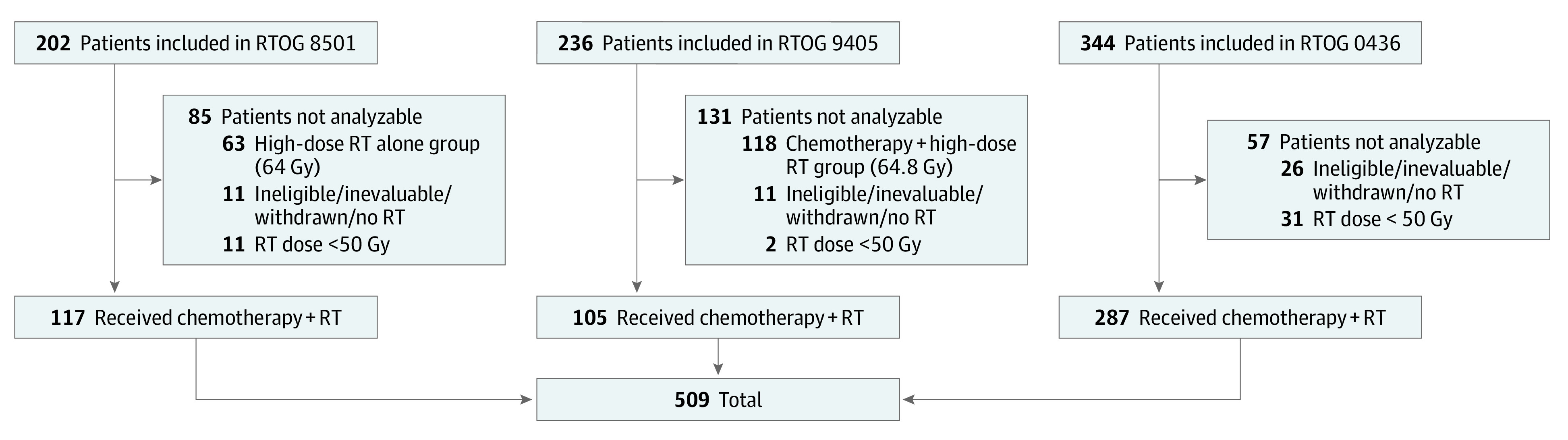
Study Flow Diagram RT indicates radiation therapy; RTOG, Radiation Therapy Oncology Group.

RTOG 8501, which was conducted between January 1986 and April 1990 and predated ClinicalTrials.gov, was a prospective, multi-institutional randomized phase 3 trial that compared high-dose RT alone with CRT.^13,14,15^ This secondary analysis included only patients in the group who received CRT. Radiotherapy was 50 Gy in 25 fractions delivered 5 days per week over 5 weeks. Chemotherapy was cisplatin, 75 mg/m^2^, delivered on day 1 of weeks 1, 5, 8, and 11 and fluorouracil, 1000 mg/m^2^, delivered on days 1 to 4 of weeks 1, 5, 8, and 11. The protocol mandated treatment interruption for an absolute neutrophil count less than 1 × 10^9^/L or platelets less than 100 × 10^9^/L.

RTOG 9405, which was conducted between June 1995 and July 1999, was a prospective, multi-institutional randomized phase 3 trial that compared standard-dose CRT with high-dose CRT.^17^ This secondary analysis included only patients in the group who received standard-dose CRT. Radiotherapy was 50.4 Gy in 28 fractions delivered 5 days per week over 5.5 weeks. Chemotherapy was cisplatin, 75 mg/m^2^, delivered on day 1 of weeks 1 and 5 and fluorouracil, 1000 mg/m^2^, delivered on days 1 to 4 of weeks 1 and 5. An additional cycle of fluorouracil and cisplatin was administered 4 weeks after completion of CRT. The protocol mandated treatment interruption for National Cancer Institute Common Toxicity Criteria grade 3 or higher toxic effects possibly related to RT.

RTOG 0436, which was conducted between June 2008 and February 2013, was a prospective, multi-institutional randomized phase 3 trial that compared standard-dose CRT without cetuximab and with cetuximab.^16^ Radiotherapy was 50.4 Gy in 28 fractions delivered 5 days per week over 5.5 weeks. Chemotherapy was cisplatin, 25 mg/m^2^, and paclitaxel, 50 mg/m^2^, delivered on days 1, 8, 15, 22, 29, and 36. Additionally, patients in the with-cetuximab group received cetuximab, 400 mg/m^2^, delivered on day 1 and cetuximab, 250 mg/m^2^, delivered on days 8, 15, 22, 29, and 36.

### RT Duration and RT Interruptions

Radiotherapy duration was defined as the interval from the date of the first fraction of RT to the date of the last fraction of RT. Radiotherapy duration was analyzed as a dichotomized variable using its median value as a cut point, using X-Tile software (Yale School of Medicine) to choose a cut-point value, and as a continuous variable.^18^ The X-Tile software cut point was generated using disease-free survival (DFS) as the end point. Radiotherapy interruption was defined as a temporary discontinuation of RT (for any reason) as indicated by the investigator on the data collection form.

### Outcomes Assessed

Outcomes assessed included local-regional failure (LRF), distant failure (DF), DFS, and overall survival (OS). Local failure was defined as disease recurrence or progression in the primary tumor. Patients who never had a local clearance were considered to have failure at day 1. Regional failure was defined as a recurrence or progression in regional lymph nodes. In the RTOG 8501 trial, nodal failure was considered to be disease present in the cervical, supraclavicular, scalene, celiac, abdominal (gastric), and other (esophageal, mediastinal, and intrathoracic) lymph nodes. In the RTOG 9405 trial, nodal failure was considered to be disease present in the cervical, supraclavicular, scalene, hilar, mediastinal, and abdominal (epigastric) lymph nodes. In the RTOG 0436 trial, nodal failure was considered to be nodal disease progression within the radiated field or recurrence after complete response in the radiated field. Local-regional failure was defined as local failure and/or regional failure. Distant failure was defined as disease progression in the liver, peritoneum, lung, distant lymph nodes, or other sites. Disease-free survival was defined as freedom from death, LRF, and DF. Overall survival was defined as freedom from death due to any cause. For LRF and DF, death without failure was a competing risk. All outcomes were measured from the date of randomization to the date of death or failure.

### Statistical Analysis

All data analyses were performed between March 2022 and February 2023 using SAS, version 9.4 (SAS Institute Inc). A 2-sided *P* < .05 was considered to be significant.

Local-regional failure and DF were estimated in the univariable analysis using the cumulative incidence method, and dichotomized RT duration and RT interruptions were compared using the Gray test.^19,20^ Overall survival and DFS were estimated in the univariable analysis with the Kaplan-Meier method, and dichotomized RT duration and RT interruptions were compared using the log-rank test.^21,22^ Univariable and multivariable Cox proportional hazards regression models were used to examine the correlation between RT duration and RT interruptions with OS and DFS. Univariable and multivariable Fine-Gray regression models were used to investigate the effects of RT duration and RT interruptions on LRF and DF.^23,24^ Separate models were built to assess RT duration and RT interruptions. In all modeling, the NRG Oncology trial (RTOG 8501, RTOG 9405, or RTOG 0436) was forced into the models along with RT duration and RT interruptions. The trial was used to account for time and the changing of treatment over time.

For the multivariable analyses, after RT duration or RT interruptions and the NRG Oncology trial were forced into the models, a stepwise selection procedure was used to choose other variables using an α ≥ .05 level as the entry and exit criteria for the model building. The following additional variables were assessed in the models: age (continuous); sex (male vs female); race and ethnicity, which were derived from electronic health records and categorized as White vs other (including Black, Asian, and other in the RTOG 8501 trial; Black or African American, Hispanic, Native American [Aleutian, American Indian, and Eskimo], Native Hawaiian or other Pacific Islander, and Asian [Chinese, Japanese, Korean, and Southeast Asian] in the RTOG 9405 trial; and American Indian or Alaska Native, Asian, Black or African American, more than 1 race, and unknown in the RTOG 0436 trial); Zubrod performance status score (0 vs 1 or 2); histological subtype (squamous cell vs adenocarcinoma); primary tumor size (<5cm vs ≥5 cm); weight loss (<10% vs ≥10%); N category (NX or N1 vs N0); and 80% or greater of protocol-specified concurrent chemotherapy (yes vs no).

## Results

The cohort included 509 patients, of whom 117 evaluable patients were from the RTOG 8501 trial, 105 evaluable patients were from the RTOG 9405 trial, and 287 evaluable patients were from the RTOG 0436 trial (Figure; Table 1). Patients had a median (IQR) age of 64 (57-70) years and included 418 males (82%) and 91 females (18%) as well as 376 White individuals (74%) and 133 (26%) individuals with other race and ethnicity. The median (IQR) RT duration was 39 (38-43) days. Radiotherapy interruptions occurred in 207 patients (41%). The median (IQR) follow-up was 1.55 (0.73-3.25) years for all patients and 4.01 (2.93-4.92) years for surviving patients (Table 1).

**Table 1.** Patient, Tumor, and Treatment Characteristics

Characteristic	No. (%)
No. of patients	509
Age, median (IQR), y	64 (57-70)
Sex	
Male	418 (82)
Female	91 (18)
Race and ethnicity^a^	
White	376 (74)
Other^b^	133 (26)
Zubrod score	
0	254 (50)
1 or 2	255 (50)
Histological subtype	
Adenocarcinoma	217 (43)
Squamous	292 (57)
Tumor size, cm	
<5	187 (37)
≥5	322 (63)
T category	
T1	33 (6)
T2	156 (31)
T3	271 (53)
T4	26 (5)
TX	22 (4)
Unknown	1 (<1)
N category	
N0	263 (52)
N1	222 (44)
NX	24 (5)
NRG Oncology trial	
RTOG 0436	287 (56)
RTOG 8501	117 (23)
RTOG 9405	105 (21)
≥80% Of protocol-specified concurrent chemotherapy	
No	42 (8)
Yes	467 (92)
RT dose, median (IQR), Gy	50.4 (50.4-50.4)
RT duration, median (IQR), d	39 (38-43)

Abbreviations: Gy, gray; RT, radiation therapy; RTOG, Radiation Therapy Oncology Group.

^a^ Race and ethnicity were derived from electronic health records.

^b^ Other category included Black, Asian, and other in the RTOG 8501 trial; Black or African American, Hispanic, Native American (Aleutian, American Indian, and Eskimo), Native Hawaiian or other Pacific Islander, and Asian (Chinese, Japanese, Korean, and Southeast Asian) in the RTOG 9405 trial; and American Indian or Alaska Native, Asian, Black or African American, more than 1 race, and unknown in the RTOG 0436 trial.

With the median value as a cut point, RT duration was 39 days or less in 271 patients (53%) and more than 39 days in 238 patients (47%) (eTable 2 in Supplement 4). With the X-Tile software cut point, RT duration was 45 days or less in 446 patients (88%) and more than 45 days in 63 patients (12%). In analysis of pretreatment characteristics by RT duration, female (vs male) sex and other (vs White) race and ethnicity were associated with RT duration longer than 45 days (eTable 1 in Supplement 4). Patients with RT duration of more than 45 days were more likely to be female vs male (18 of 63 [29%] vs 73 of 446 [16%]; *P* = .02), and there were more patients with other vs White race and ethnicity (23 of 63 [37%] vs 110 of 446 [25%]) with RT duration of more than 45 days. Patients with RT interruptions were more likely to have other vs White race and ethnicity (31% vs 23%; *P* = .04), have a Zubrod score of 1 or 2 vs 0 (57% vs 46%; *P* = .02), and have a tumor size of 5 cm or larger vs smaller than 5 cm (70% vs 59%; *P* = .01). More female than male patients had RT interruptions (22% vs 15%; *P* = .06) (eTable 3 in Supplement 4).

### Outcomes for RT Duration Dichotomized by X-Tile Software Cut Point

In univariable analysis, RT duration longer than 45 days (vs ≤45 days) was associated with inferior LRF and DFS and a pattern of inferior OS (eFigure 1 in Supplement 4). After controlling for an NRG Oncology trial, RT duration longer than 45 days (vs ≤45 days) remained associated with increased risk of having a DFS failure (hazard ratio [HR], 1.41; 95% CI, 1.07-1.86; *P* = .01). The HR for LRF was 1.38, but the results were not statistically significant (95% CI, 0.99-1.90; *P* = .05) (eTable 4 in Supplement 4). After controlling for an NRG Oncology trial, sex, Zubrod score, tumor size, N category, histological subtype, and 80% or greater protocol-specified concurrent chemotherapy, RT duration longer than 45 days (vs ≤45 days) was associated with increased risk of having a DFS failure (HR, 1.34; 95% CI, 1.01-1.77; *P* = .04). The HR for OS was 1.33, but the results were not statistically significant (95% CI, 0.99-1.77; *P* = .05) (Table 2). The association of RT duration longer than 45 days (vs ≤45 days) with LRF was not maintained in multivariable analysis (Table 2).

**Table 2.** Multivariable Models: Radiation Therapy Duration (N = 509 Patients)

Variable^a^	OS (n = 409 deaths)		DFS (n = 445 failures)		LRF (n = 288 failures)		DF (n = 219 failures)	
	HR (95% CI)	*P* value^b^	HR (95% CI)	*P* value^b^	HR (95% CI)	*P* value^b^	HR (95% CI)	*P* value^b^
RT duration								
≤45 d	1 [Reference]	NA	1 [Reference]	NA	1 [Reference]	NA	1 [Reference]	NA
>45 d	1.33 (0.99-1.77)	.05	1.34 (1.01-1.77)	.04	1.25 (0.90-1.75)	.18	0.80 (0.52-1.24)	.32
NRG Oncology trial								
RTOG 0436	1 [Reference]	NA	1 [Reference]	NA	1 [Reference]	NA	1 [Reference]	NA
RTOG 8501	1.10 (0.84-1.44)	.50	1.02 (0.77-1.35)	.88	1.19 (0.89-1.59)	.25	0.79 (0.54-1.15)	.22
RTOG 9405	0.95 (0.73-1.25)	.73	1.06 (0.81-1.40)	.67	1.33 (0.99-1.78)	.05	1.00 (0.69-1.45)	.99
Age (continuous; unit increase = 10 y)	NA		NA		0.86 (0.77-0.97)	.01	0.84 (0.73-0.97)	.02
Sex								
Female	1 [Reference]	NA	1 [Reference]	NA	NA	NA	NA	NA
Male	1.51 (1.15-1.99)	.003	1.32 (1.01-1.71)	.04	NA		NA	
Race and ethnicity^c^								
White	NA		NA		NA		NA	
Other^d^	NA		NA		NA		NA	
Zubrod score								
0	1 [Reference]	NA	1 [Reference]	NA	1 [Reference]	NA	NA	NA
1 or 2	1.26 (1.04-1.54)	.02	1.27 (1.05-1.53)	.01	1.31 (1.05-1.63)	.02	NA	
Tumor size, cm								
<5	1 [Reference]	NA	1 [Reference]	NA	NA	NA	1 [Reference]	NA
≥5	1.43 (1.15-1.78)	.001	1.31 (1.07-1.61)	.01	NA	NA	1.35 (1.01-1.80)	.04
N category								
N0	1 [Reference]	NA	1 [Reference]	NA	1 [Reference]	NA	NA	NA
NX or N1	1.37 (1.10-1.70)	.005	1.35 (1.10-1.67)	.005	1.36 (1.07-1.74)	.01	NA	NA
Histological subtype								
Adenocarcinoma	NA	NA	1 [Reference]	NA	NA	NA	1 [Reference]	NA
Squamous	NA		0.77 (0.61-0.96)	.02	NA		0.60 (0.44-0.80)	<.001
≥80% Of protocol-specified concurrent chemotherapy								
No	NA	NA	1 [Reference]	NA	NA	NA	NA	NA
Yes	NA	NA	0.67 (0.48-0.93)	.02	NA	NA	NA	NA

Abbreviations: DF, distant failure; DFS, disease-free survival; HR, hazard ratio; LRF, local-regional failure; NA, not applicable; OS, overall survival; RT, radiation therapy; RTOG, Radiation Therapy Oncology Group.

^a^ RT duration and NRG Oncology trial were included in each model, and other variables were included if *P* < .05.

^b^ *P* values were from the Cox proportional hazards regression model (OS/DFS) or the Fine-Gray regression model (LRF/DF).

^c^ Race and ethnicity were derived from electronic health records.

^d^ Other category included Black, Asian, and other in the RTOG 8501 trial; Black or African American, Hispanic, Native American (Aleutian, American Indian, and Eskimo), Native Hawaiian or other Pacific Islander, and Asian (Chinese, Japanese, Korean, and Southeast Asian) in the RTOG 9405 trial; and American Indian or Alaska Native, Asian, Black or African American, more than 1 race, and unknown in the RTOG 0436 trial.

Exploratory analysis was performed within cohorts of patients with a squamous cell carcinoma or adenocarcinoma histological subtype. In univariable analysis in the squamous cell carcinoma cohort, RT duration longer than 45 days (vs ≤45 days) was associated with inferior LRF and DFS (eTable 5 in Supplement 4). In univariable analysis in the adenocarcinoma cohort, RT duration longer than 45 days (vs ≤45 days) was not associated with outcomes (eTable 6 in Supplement 4).

### Outcomes for RT Duration as a Continuous Variable

After controlling for an NRG Oncology trial, a 1-week increase in RT duration was associated with a 17% increased risk of having a DFS failure (HR, 1.17; 95% CI, 1.04-1.32; *P* = .009) and an LRF (HR, 1.17; 95% CI, 1.04-1.31; *P* = .01) (eTable 7 in Supplement 4). After controlling for an NRG Oncology trial, sex, Zubrod score, tumor size, N category, histological subtype, and 80% or greater protocol-specified concurrent chemotherapy, a 1-week increase in RT duration was associated with a 14% increased risk of having a DFS failure (HR, 1.14; 95% CI, 1.01-1.28; *P* = .03) (Table 3). The HR for LRF was 1.13, but the results were not statistically significant (95% CI, 0.99-1.28; *P* = .07) (Table 3).

**Table 3.** Multivariable Models: Radiation Therapy Duration, Continuous (N = 509 Patients)

Variable^a^	OS (n = 409 deaths)		DFS (n = 445 failures)		LRF (n = 288 failures)		DF (n = 219 failures)	
	HR (95% CI)	*P* value^b^	HR (95% CI)	*P* value^b^	HR (95% CI)	*P* value^b^	HR (95% CI)	*P* value^b^
RT duration (continuous; unit increase = 7 d)	1.08 (0.97-1.22)	.17	1.14 (1.01-1.28)	.03	1.13 (0.99-1.28)	.07	0.87 (0.72-1.03)	.11
NRG Oncology trial								
RTOG 0436	1 [Reference]	NA	1 [Reference]	NA	1 [Reference]	NA	1 [Reference]	NA
RTOG 8501	1.11 (0.85-1.46)	.45	1.06 (0.80-1.40)	.71	1.21 (0.91-1.62)	.19	0.76 (0.52-1.11)	.15
RTOG 9405	0.96 (0.73-1.26)	.76	1.08 (0.82-1.42)	.59	1.33 (0.99-1.78)	.05	0.99 (0.68-1.44)	.95
Age (continuous; unit increase = 10 y)	NA		NA		0.86 (0.77-0.97)	.01	0.84 (0.73-0.97)	.02
Sex								
Female	1 [Reference]	NA	1 [Reference]	NA	NA	NA	NA	NA
Male	1.50 (1.14-1.96)	.004	1.31 (1.01-1.70)	.045	NA		NA	
Race and ethnicity^c^								
White	NA	NA	NA	NA	NA	NA	NA	NA
Other^d^	NA	NA	NA	NA	NA	NA	NA	NA
Zubrod score								
0	1 [Reference]	NA	1 [Reference]	NA	1 [Reference]	NA	NA	NA
1 or 2	1.26 (1.04-1.53)	.02	1.27 (1.05-1.53)	.01	1.30 (1.05-1.62)	.02	NA	NA
Tumor size, cm								
<5	1 [Reference]	NA	1 [Reference]	NA	NA	NA	1 [Reference]	NA
≥5	1.43 (1.15-1.78)	.001	1.30 (1.06-1.60)	.01	NA		1.36 (1.02-1.81)	.04
N category								
N0	1 [Reference]	NA	1 [Reference]	NA	1 [Reference]	NA	NA	NA
NX or N1	1.36 (1.10-1.69)	.005	1.35 (1.10-1.67)	.005	1.36 (1.07-1.73)	.01	NA	
Histological subtype								
Adenocarcinoma	NA	NA	1 [Reference]	NA	NA	NA	1 [Reference]	NA
Squamous	NA	NA	0.76 (0.61-0.95)	.02	NA	NA	0.60 (0.45-0.81)	<.001
≥80% Of protocol-specified concurrent chemotherapy								
No	NA	NA	1 [Reference]	NA	NA	NA	NA	NA
Yes	NA	NA	0.68 (0.49-0.95)	.02	NA	NA	NA	NA

Abbreviations: DF, distant failure; DFS, disease-free survival; HR, hazard ratio; LRF, local-regional failure; NA, not applicable; OS, overall survival; RT, radiation therapy; RTOG, Radiation Therapy Oncology Group.

^a^ RT duration and NRG Oncology trial were included in each model, and other variables were included if *P* < .05.

^b^ *P* values were from the Cox proportional hazards regression model (OS/DFS) or the Fine-Gray regression model (LRF/DF).

^c^ Race and ethnicity were derived from electronic health records.

^d^ Other category included Black, Asian, and other in the RTOG 8501 trial; Black or African American, Hispanic, Native American (Aleutian, American Indian, and Eskimo), Native Hawaiian or other Pacific Islander, and Asian (Chinese, Japanese, Korean, and Southeast Asian) in the RTOG 9405 trial; and American Indian or Alaska Native, Asian, Black or African American, more than 1 race, and unknown in the RTOG 0436 trial.

### Outcomes for RT Duration Dichotomized by Median 

In univariable analysis, RT duration longer than 39 days (vs ≤39 days) was associated with a higher risk of LRF but not DF, DFS, or OS (eFigure 2 in Supplement 4). After controlling for an NRG Oncology trial, RT duration longer than 39 days (vs ≤39 days) remained associated with increased risk of having an LRF (HR, 1.37; 95% CI, 1.10-1.71; *P* = .004) (eTable 8 in Supplement 4). After controlling for an NRG Oncology trial, age, Zubrod score, and N category, RT duration longer than 39 days (vs ≤39 days) was still associated with increased risk of having an LRF (HR, 1.32; 95% CI, 1.06-1.65; *P* = .01) (eTable 9 in Supplement 4). Radiotherapy duration longer than 39 days (vs ≤39 days) was not associated with OS, DFS, or DF in multivariable analysis.

### Outcomes for RT Interruptions

In univariable analysis, patients with RT interruptions (vs no interruptions) had patterns of inferior DFS and LRF (eFigure 3 in Supplement 4). After controlling for an NRG Oncology trial, the HR for DFS was 1.19, but the results were not statistically significant (95% CI, 0.99-1.44; *P* = .07) (eTable 10 in Supplement 4). After adjusting for important variables, RT interruptions (vs no interruptions) were not associated with OS, DFS, LRF, or DF in multivariable analysis (Table 4).

**Table 4.** Multivariable Models: Radiation Therapy Interruptions (N = 509 Patients)

Variable^a^	OS (n = 409 deaths)		DFS (n = 445 failures)		LRF (n = 288 failures)		DF (n = 219 failures)	
	HR (95% CI)	*P* value^b^	HR (95% CI)	*P* value^b^	HR (95% CI)	*P* value^b^	HR (95% CI)	*P* value^b^
RT interruptions								
No	1 [Reference]	NA	1 [Reference]	NA	1 [Reference]	NA	1 [Reference]	NA
Yes	1.09 (0.89-1.33)	.42	1.13 (0.93-1.36)	.23	1.17 (0.94-1.46)	.17	1.06 (0.81-1.39)	.69
NRG Oncology trial								
RTOG 0436	1 [Reference]	NA	1 [Reference]	NA	1 [Reference]	NA	1 [Reference]	NA
RTOG 8501	1.10 (0.84-1.45)	.47	1.07 (0.81-1.42)	.63	1.19 (0.89-1.60)	.23	0.78 (0.54-1.14)	.20
RTOG 9405	0.96 (0.73-1.25)	.74	1.08 (0.82-1.42)	.59	1.33 (0.99-1.78)	.05	0.96 (0.66-1.39)	.81
Age (continuous; unit increase = 10 y)	NA	NA	0.90 (0.82-0.99)	.05	0.86 (0.76-0.96)	.008	0.86 (0.74-0.99)	.04
Sex								
Female	1 [Reference]	NA	NA	NA	NA	NA	1 [Reference]	NA
Male	1.50 (1.14-1.97)	.004	NA	NA	NA	NA	1.52 (1.01-2.29)	.05
Race and ethnicity^c^								
White	NA	NA	NA	NA	NA	NA	NA	NA
Other^d^	NA	NA	NA	NA	NA	NA	NA	NA
Zubrod score								
0	1 [Reference]	NA	1 [Reference]	NA	1 [Reference]	NA	NA	NA
1 or 2	1.25 (1.03-1.52)	.02	1.29 (1.07-1.56)	.008	1.30 (1.05-1.62)	.02	NA	NA
Tumor size, cm								
<5	1 [Reference]	NA	1 [Reference]	NA	NA	NA	1 [Reference]	NA
≥5	1.43 (1.15-1.78)	.001	1.30 (1.06-1.60)	.01	NA	NA	1.35 (1.01-1.81)	.04
N category								
N0	1 [Reference]	NA	1 [Reference]	NA	1 [Reference]	NA	NA	NA
NX or N1	1.36 (1.10-1.69)	.005	1.37 (1.11-1.69)	.003	1.37 (1.08-1.75)	.01	NA	NA
Histological subtype								
Adenocarcinoma	NA	NA	1 [Reference]	NA	NA	NA	1 [Reference]	NA
Squamous	NA	NA	0.70 (0.56-0.87)	.002	NA	NA	0.64 (0.47-0.87)	.004
≥80% Of protocol-specified concurrent chemotherapy								
No	NA	NA	1 [Reference]	NA	NA	NA	1 [Reference]	NA
Yes	NA	NA	0.64 (0.46-0.89)	.008	NA	NA	2.03 (1.03-4.01)	.04

Abbreviations: DF, distant failure; DFS, disease-free survival; HR, hazard ratio; LRF, local-regional failure; NA, not applicable; OS, overall survival; RT, radiation therapy; RTOG, Radiation Therapy Oncology Group.

^a^ RT duration and NRG Oncology trial were included in each model, and other variables were included if *P* < .05.

^b^ *P* values were from the Cox proportional hazards regression model (OS/DFS) or the Fine-Gray regression model (LRF/DF).

^c^ Race and ethnicity were derived from electronic health records.

^d^ Other category included Black, Asian, and other in the RTOG 8501 trial; Black or African American, Hispanic, Native American (Aleutian, American Indian, and Eskimo), Native Hawaiian or other Pacific Islander, and Asian (Chinese, Japanese, Korean, and Southeast Asian) in the RTOG 9405 trial; and American Indian or Alaska Native, Asian, Black or African American, more than 1 race, and unknown in the RTOG 0436 trial.

## Discussion

In this secondary analysis of patients with esophageal cancer treated with definitive CRT in NRG Oncology trials, the frequency of RT interruptions and the length of RT duration were characterized. The association between RT interruptions and RT duration and outcomes, including LRF, DFS, and OS, was examined. Key findings were (1) RT interruptions were common, occurring in 41% of patients; (2) RT interruptions and prolonged RT duration were more common in female than male patients and in those with other vs White race and ethnicity; and (3) RT interruptions and prolonged RT duration were associated with worse LRF and DFS in several models, supporting the hypothesis of correlation with inferior outcomes.

There are few published reports evaluating the effect of RT interruptions and duration on outcomes for patients receiving definitive RT or CRT for esophageal cancer. In patients with localized esophageal cancer treated with RT alone (without chemotherapy), a meta-analysis of 11 randomized clinical trials conducted in mainland China found improved local control and survival following late-course accelerated, hyperfractionated RT regimens that delivered 60 to 70 Gy in 5 to 6 weeks compared with standard regimens delivering 60 to 70 Gy in 6 to 7 weeks.^25^ Crehange et al^26^ performed a post hoc analysis of a phase 3 randomized clinical trial examining the effect of a CRT schedule on outcomes in 446 patients with esophageal cancer treated with CRT alone vs CRT followed by surgery. The protocol allowed either continuous CRT (46 Gy in 4.5 weeks with 2 cycles of fluorouracil and cisplatin, with an additional 20 Gy in 2 weeks administered to those who were randomized to CRT alone) or split-course CRT (15 Gy in 1 week with fluorouracil and cisplatin followed by a 2-week break, then 15 Gy in 1 week with fluorouracil and cisplatin, with a third course of 15 Gy administered in 1 week to those who were randomized to CRT alone).^26^ Patients who received continuous (vs split-course) CRT had improved local recurrence–free survival (2 years: 77% vs 57%; *P* = .002) but similar survival.^26^ Di Fiore et al^27^ retrospectively compared outcomes at 2 French centers, 1 of which used a continuous course regimen of 50 Gy in 25 fractions over 5 weeks with fluorouracil and cisplatin (n = 74) and the other used a double-split course regimen of 60 Gy in 30 fractions over 10 weeks (3 courses of 20 Gy in 10 fractions with 2-week breaks) with fluorouracil and cisplatin (n = 55). Patients who received continuous course treatment had a higher rate of complete clinical response, but no differences were observed for other outcomes.^27^ Findings of these previous studies along with those of the present analysis provide evidence to support the hypothesis that prolonged RT duration has an adverse effect on disease control in patients receiving definitive CRT for esophageal cancer.

In the context of the COVID-19 pandemic, patients and oncologists have faced substantial uncertainty and challenging decisions, including consideration of cancer treatment breaks. Recommendations have been proposed regarding management of esophageal cancer in the context of COVID-19, but none specifically address the issue of RT interruptions.^12,28^ The appropriateness of RT interruptions due to COVID-19 has been addressed in management guidelines for other solid malignant neoplasms. For example, in a modified Delphi consensus process of ESTRO-ASTRO (European Society for Radiotherapy and Oncology/American Society for Radiation Oncology) lung cancer experts, there was lack of consensus regarding the decision to interrupt RT in patients with COVID-19 receiving definitive CRT, with 57% of the panel recommending interruption and 43% recommending continuation of RT.^29^ In an ESTRO-ASTRO panel of head and neck cancer experts, there was strong agreement that definitive RT should not be interrupted for patients with mild COVID-19 symptoms but should be interrupted for patients with severe COVID-19 symptoms until they have fully recovered.^30^ On the basis of observations from the present study, the same guidance for patients with esophageal cancer treated with definitive CRT could be recommended. Consistent with the National Comprehensive Cancer Network guidelines, all patients with newly diagnosed esophageal cancer who will be treated with definitive CRT are strongly recommended to undergo COVID-19 vaccination to minimize the likelihood of SARS-CoV-2 infection and the potential need for RT interruption.^31^

Patients with other race and ethnicity and/or female sex were more likely to experience RT interruptions and prolonged RT duration. Race and ethnicity were not independently associated with inferior outcomes in the multivariable models, although US population–based studies have observed a higher rate of mortality in Black than in White patients with esophageal cancer, which may be explained in part by higher T category at diagnosis and decreased use of surgery.^32,33^ In a study of 3744 patients with mixed cancer types receiving RT at a single academic center in the US between 2015 and 2017, Black (compared with White) patients had a higher rate of RT interruptions (43% vs 29%).^34^ To our knowledge, the association between race and ethnicity and RT duration and interruptions is a novel finding in the context of definitive CRT for esophageal cancer. Similarly, the association between female sex and RT duration and interruptions is novel. Further work is needed to confirm these findings and to investigate the factors associated with racial and sex disparities and potential interventions to address these disparities.

### Strengths and Limitations

Strengths of this study included the prospective data collection using NRG Oncology trials. Length of follow-up was relatively long, with a median follow-up of 4 years for surviving patients. Additionally, the cohort was relatively large with a relatively uncommon cancer.

Study limitations included the cohort not being sufficiently large to exclude the possibility of modest, although clinically relevant, differences in survival associated with RT interruptions and duration. There was an association between RT duration and inferior LRF, DFS, and OS in some, but not all, models. Because this study was an unplanned post hoc analysis, we could not account for potential sources of bias. Enrollment in these NRG Oncology trials spanned approximately 3 decades, during which there were improvements in staging, RT planning and delivery, and supportive care. Such changes contributed to the heterogeneity of these trials, although the trial was included as a variable in the models to account for this heterogeneity. Furthermore, the trials were not associated with OS, DFS, LRF, or DF in the multivariable models.

## Conclusions

In this secondary analysis of 3 NRG Oncology randomized clinical trials, prolonged RT duration was associated with inferior outcomes. Female patients and those with other race and ethnicity were more likely to have prolonged RT duration and experience RT interruptions. Radiotherapy interruptions should be minimized to optimize outcomes. As a practical recommendation, for a patient starting a course of 28 fractions of RT on a Monday, the goal should be to complete the treatment course by Friday of the sixth week (ie, <40 days). Treatment interruptions should be minimized by using aggressive supportive care and interventions to reduce patient nonadherence. If interruptions occur, incorporating weekend and/or twice-daily treatments, if appropriate, could be considered to keep the treatment duration less than 40 days. Findings from the present study suggest that investigating further reduction in RT duration (<5 weeks) might be warranted, such as through use of moderate hypofractionation RT regimens.

## Notes

Supplement 1.
RTOG 0436 Protocol

Supplement 2.
RTOG 8501 Protocol

Supplement 3.
RTOG 9405 Protocol

Supplement 4. **eTable 1.** Characteristics by RT Duration ≤45 Days vs >45 Days **eTable 2.** Characteristics by RT Duration ≤39 Days vs >39 Days **eTable 3.** Characteristics by RT Interruptions **eTable 4.** RT Duration ≤45 Days vs >45 Days, Controlling for NRG Oncology Trial **eTable 5.** RT Duration ≤45 Days vs >45 Days in Patients with Squamous Cell Carcinoma Histology **eTable 6.** RT Duration ≤45 Days vs >45 Days in Patients with Adenocarcinoma Histology **eTable 7.** RT Duration (Continuous), Controlling for NRG Oncology Trial **eTable 8.** RT Duration ≤39 Days vs >39 Days, Controlling for NRG Oncology Trial **eTable 9.** Multivariable Models: RT Duration (≤ 39 Days vs > 39 Days) **eTable 10.** RT Interruptions, Controlling for NRG Oncology Trial **eFigure 1.** Outcomes for RT Duration Dichotomized by XTile Cutpoint **eFigure 2.** Outcomes for RT Duration Dichotomized by Median **eFigure 3.** Outcomes for RT Interruption

Supplement 5.
Data Sharing Statement

## References

[1] Bese NS, Hendry J, Jeremic B. Effects of prolongation of overall treatment time due to unplanned interruptions during radiotherapy of different tumor sites and practical methods for compensation. Int J Radiat Oncol Biol Phys. 2007;68(3):654-661. doi:10.1016/j.ijrobp.2007.03.010 17467926

[2] Fowler JF, Lindstrom MJ. Loss of local control with prolongation in radiotherapy. Int J Radiat Oncol Biol Phys. 1992;23(2):457-467. doi:10.1016/0360-3016(92)90768-D 1534082

[3] Cox JD, Pajak TF, Asbell S, . Interruptions of high-dose radiation therapy decrease long-term survival of favorable patients with unresectable non-small cell carcinoma of the lung: analysis of 1244 cases from 3 Radiation Therapy Oncology Group (RTOG) trials. Int J Radiat Oncol Biol Phys. 1993;27(3):493-498. doi:10.1016/0360-3016(93)90371-2 8226140

[4] Machtay M, Hsu C, Komaki R, . Effect of overall treatment time on outcomes after concurrent chemoradiation for locally advanced non-small-cell lung carcinoma: analysis of the Radiation Therapy Oncology Group (RTOG) experience. Int J Radiat Oncol Biol Phys. 2005;63(3):667-671. doi:10.1016/j.ijrobp.2005.03.037 15927409

[5] Lanciano RM, Pajak TF, Martz K, Hanks GE. The influence of treatment time on outcome for squamous cell cancer of the uterine cervix treated with radiation: a patterns-of-care study. Int J Radiat Oncol Biol Phys. 1993;25(3):391-397. doi:10.1016/0360-3016(93)90058-4 8436516

[6] Fyles A, Keane TJ, Barton M, Simm J. The effect of treatment duration in the local control of cervix cancer. Radiother Oncol. 1992;25(4):273-279. doi:10.1016/0167-8140(92)90247-R 1480773

[7] Perez CA, Grigsby PW, Castro-Vita H, Lockett MA. Carcinoma of the uterine cervix—I: Impact of prolongation of overall treatment time and timing of brachytherapy on outcome of radiation therapy. Int J Radiat Oncol Biol Phys. 1995;32(5):1275-1288. doi:10.1016/0360-3016(95)00220-S 7635767

[8] Ben-Josef E, Moughan J, Ajani JA, . Impact of overall treatment time on survival and local control in patients with anal cancer: a pooled data analysis of Radiation Therapy Oncology Group trials 87-04 and 98-11. J Clin Oncol. 2010;28(34):5061-5066. doi:10.1200/JCO.2010.29.1351 20956625 PMC3018356

[9] Glynne-Jones R, Meadows HM, Lopes A, Muirhead R, Sebag-Montefiore D, Adams R; ACTII Study Group. Impact of compliance to chemoradiation on long-term outcomes in squamous cell carcinoma of the anus: results of a post hoc analysis from the randomised phase III ACT II trial. Ann Oncol. 2020;31(10):1376-1385. doi:10.1016/j.annonc.2020.06.012 32619648

[10] Bourhis J, Overgaard J, Audry H, ; Meta-Analysis of Radiotherapy in Carcinomas of Head and neck (MARCH) Collaborative Group. Hyperfractionated or accelerated radiotherapy in head and neck cancer: a meta-analysis. Lancet. 2006;368(9538):843-854. doi:10.1016/S0140-6736(06)69121-6 16950362

[11] Mauguen A, Le Péchoux C, Saunders MI, . Hyperfractionated or accelerated radiotherapy in lung cancer: an individual patient data meta-analysis. J Clin Oncol. 2012;30(22):2788-2797. doi:10.1200/JCO.2012.41.6677 22753901 PMC4934452

[12] Tchelebi LT, Haustermans K, Scorsetti M, . Recommendations for the use of radiation therapy in managing patients with gastrointestinal malignancies in the era of COVID-19. Radiother Oncol. 2020;148:194-200. doi:10.1016/j.radonc.2020.04.010 32342878 PMC7194719

[13] Herskovic A, Martz K, al-Sarraf M, . Combined chemotherapy and radiotherapy compared with radiotherapy alone in patients with cancer of the esophagus. N Engl J Med. 1992;326(24):1593-1598. doi:10.1056/NEJM199206113262403 1584260

[14] al-Sarraf M, Martz K, Herskovic A, . Progress report of combined chemoradiotherapy versus radiotherapy alone in patients with esophageal cancer: an intergroup study. J Clin Oncol. 1997;15(1):277-284. doi:10.1200/JCO.1997.15.1.277 8996153

[15] Cooper JS, Guo MD, Herskovic A, ; Radiation Therapy Oncology Group. Chemoradiotherapy of locally advanced esophageal cancer: long-term follow-up of a prospective randomized trial (RTOG 85-01). JAMA. 1999;281(17):1623-1627. doi:10.1001/jama.281.17.1623 10235156

[16] Suntharalingam M, Winter K, Ilson D, . Effect of the addition of cetuximab to paclitaxel, cisplatin, and radiation therapy for patients with esophageal cancer: the NRG Oncology RTOG 0436 phase 3 randomized clinical trial. JAMA Oncol. 2017;3(11):1520-1528. doi:10.1001/jamaoncol.2017.1598 28687830 PMC5710193

[17] Minsky BD, Pajak TF, Ginsberg RJ, . INT 0123 (Radiation Therapy Oncology Group 94-05) phase III trial of combined-modality therapy for esophageal cancer: high-dose versus standard-dose radiation therapy. J Clin Oncol. 2002;20(5):1167-1174. doi:10.1200/JCO.2002.20.5.1167 11870157

[18] Camp RL, Dolled-Filhart M, Rimm DL. X-tile: a new bio-informatics tool for biomarker assessment and outcome-based cut-point optimization. Clin Cancer Res. 2004;10(21):7252-7259. doi:10.1158/1078-0432.CCR-04-0713 15534099

[19] Kalbfleisch J, Prentice R. The Statistical Analysis of Failure Time Data. John Wiley & Sons, Inc; 1980:167-169.

[20] Gray RJ. A class of K-sample tests for comparing the cumulative incidence of a competing risk. Ann Stat. 1988;16(3):1141-1154. doi:10.1214/aos/1176350951

[21] Kaplan EL, Meier P. Nonparametric estimation from incomplete observations. J Am Stat Assoc. 1958;53(282):457-481. doi:10.1080/01621459.1958.10501452

[22] Mantel N. Evaluation of survival data and two new rank order statistics arising in its consideration. Cancer Chemother Rep. 1966;50(3):163-170. 5910392

[23] Cox DR. Regression models and life-tables. J R Stat Soc Series B Stat Methodol. 1972;34(2):187-220.

[24] Fine JP, Gray RJ. A proportional hazards model for the subdistribution of a competing risk. J Am Stat Assoc. 1999;94(446):496-509. doi:10.1080/01621459.1999.10474144

[25] Zhang YW, Chen L, Bai Y, Zheng X. Long-term outcomes of late course accelerated hyper-fractionated radiotherapy for localized esophageal carcinoma in Mainland China: a meta-analysis. Dis Esophagus. 2011;24(7):495-501. doi:10.1111/j.1442-2050.2010.01173.x 21309922

[26] Crehange G, Maingon P, Peignaux K, ; Federation Francophone de Cancerologie Digestive 9102. Phase III trial of protracted compared with split-course chemoradiation for esophageal carcinoma: Federation Francophone de Cancerologie Digestive 9102. J Clin Oncol. 2007;25(31):4895-4901. doi:10.1200/JCO.2007.12.3471 17971585

[27] Di Fiore F, Lecleire S, Galais MP, . Impact of radiation schedule and chemotherapy duration in definitive chemoradiotherapy regimen for esophageal cancer. Gastroenterol Clin Biol. 2006;30(6-7):845-851. doi:10.1016/S0399-8320(06)73331-0 16885868

[28] Triantafyllou T, Olson MT, Theodorou D, Zografos G, Singhal S. Esophageal cancer: challenges, concerns, and recommendations for management amidst the COVID-19 pandemic. Ann Gastroenterol. 2020;33(5):453-458. doi:10.20524/aog.2020.0519 32879590 PMC7406811

[29] Guckenberger M, Belka C, Bezjak A, . Practice recommendations for lung cancer radiotherapy during the COVID-19 pandemic: an ESTRO-ASTRO consensus statement. Int J Radiat Oncol Biol Phys. 2020;107(4):631-640. doi:10.1016/j.ijrobp.2020.05.012 32589990 PMC7836268

[30] Thomson DJ, Palma D, Guckenberger M, . Practice recommendations for risk-adapted head and neck cancer radiotherapy during the COVID-19 pandemic: an ASTRO-ESTRO consensus statement. Radiother Oncol. 2020;151:314-321. doi:10.1016/j.radonc.2020.04.019 32730830 PMC7384409

[31] National Comprehensive Cancer Network. Recommendations of the National Comprehensive Cancer Network (NCCN) COVID-19 Vaccination Advisory Committee. August 30, 2021. Accessed December 1, 2022. https://www.nccn.org/docs/default-source/covid-19/2021_covid-19_vaccination_guidance_v3-0.pdf?sfvrsn=b483da2b_60

[32] Tramontano AC, Nipp R, Mercaldo ND, Kong CY, Schrag D, Hur C. Survival disparities by race and ethnicity in early esophageal cancer. Dig Dis Sci. 2018;63(11):2880-2888. doi:10.1007/s10620-018-5238-6 30109578 PMC6738563

[33] Kim A, Ashman P, Ward-Peterson M, Lozano JM, Barengo NC. Racial disparities in cancer-related survival in patients with squamous cell carcinoma of the esophagus in the US between 1973 and 2013. PLoS One. 2017;12(8):e0183782. doi:10.1371/journal.pone.0183782 28832659 PMC5568373

[34] Wakefield DV, Carnell M, Dove APH, . Location as destiny: identifying geospatial disparities in radiation treatment interruption by neighborhood, race, and insurance. Int J Radiat Oncol Biol Phys. 2020;107(4):815-826. doi:10.1016/j.ijrobp.2020.03.016 32234552 PMC13440333

