# Supplementary material for: Association of Radiotherapy Duration With Clinical Outcomes in Patients With Esophageal Cancer Treated in NRG Oncology Trials: A Secondary Analysis of NRG Oncology Randomized Clinical Trials
Source: JAMA Netw Open. 2023 Apr 21;6(4):e238504. doi: 10.1001/jamanetworkopen.2023.8504 (PMC10122174; doi:10.1001/jamanetworkopen.2023.8504)
Supplement: Supplement 1. — RTOG 0436 Protocol [file jamanetwopen-e238504-s001.pdf]

RADIATION THERAPY ONCOLOGY GROUP

RTOG 0436

A PHASE III TRIAL EVALUATING THE ADDITION OF CETUXIMAB TO PACLITAXEL, CISPLATIN, AND RADIATION FOR PATIENTS WITH ESOPHAGEAL CANCER WHO ARE TREATED WITHOUT SURGERY

Cetuximab (IND 101157)

(9/13/2011)

Study Chairs

Radiation Oncology
Mohan Suntharalingam, MD
University of Maryland School of Medicine
22 South Greene Street
Baltimore, MD 21201
410-328-2331/FAX: 410-328-6911
msuntha@umm.edu

Medical Oncology
David Ilson, MD, PhD
Memorial Sloan Kettering Cancer Center
1275 York Avenue
New York, NY 10021
212-639-8306/FAX: 212-717-3320
ilsond@mskcc.org

Translational Research
Adam P. Dicker, MD, PhD
Department of Radiation Oncology
Thomas Jefferson University
111 South 11th Street
Philadelphia, PA 19107-5097
215-955-6527/FAX: 215-955-0412
Adam.dicker@jeffersonhospital.org

Outcomes/Quality of Life
Lisa Kachnic, MD
Department of Radiation Oncology
Boston University Medical Center
830 Harrison Avenue, LL
Boston, MA 02118
617-638-7070/FAX: 617-638-7037
lisa.kachnic@bmc.org

Outcomes/Economics (9/22/09)
André Konski, MD
Wayne State Univ. College of Medicine
540 E. Canfield Rd./1212 Scott Hall
Detroit, MI 48201
313-966-2274/FAX: 313-966-9400
akonski@med.wayne.edu

Senior Statistician
Kathryn Winter, MS
Radiation Therapy Oncology Group/ACR
1818 Market Street, Suite 1600
Philadelphia, PA 19103
215-574-3198/FAX 215-928-0153
kwinter@acr.org

ECOG Co-Chair (8/12/08)
Bapsi Chakravarthy, MD
Vanderbilt University Medical Center
TVC-902, Radiation Oncology
Nashville, TN 37232-5671
615-322-2555/FAX: 615-343-0161
bapsi.chak@vanderbilt.edu

Document History

	Version/Update Date	Broadcast Date
Closure	--	February 8, 2013
Amendment 6	May 3, 2012	May 16, 2012
Amendment 5	September 13, 2011	October 4, 2011
Amendment 4	August 19, 2010	September 21, 2010
Amendment 3	June 10, 2010	July 9, 2010
Update	June 22, 2010	June 22, 2010
Update	September 22, 2009	September 22, 2009
Amendment 2	September 9, 2009	September 22, 2009
Update	May 28, 2009	May 28, 2009
Amendment 1	February 13, 2009	March 31, 2009
Update	March 31, 2009	March 31, 2009
Update	August 8, 2008	August 8, 2008
Activation	March 28, 2008	June 30, 2008
Pre activation	April 24, 2008	April 24, 2008

RTOG Headquarters
215-574-3189
1-800-227-5463, ext. 4189

This protocol was designed and developed by the Radiation Therapy Oncology Group (RTOG) of the American College of Radiology (ACR). It is intended to be used only in conjunction with institution-specific IRB approval for study entry. No other use or reproduction is authorized by RTOG nor does RTOG assume any responsibility for unauthorized use of this protocol.

(6/22/10)

This study is supported by the NCI Cancer Trials Support Unit (CTSU).

Institutions not aligned with RTOG will participate through the CTSU mechanism as outlined below and detailed in Section 5.0.

- The **study protocol and all related forms and documents** must be downloaded from the protocol-specific Web page of the CTSU Member Web site located at https://members.ctsu.org
- Send completed **site registration documents** to the CTSU Regulatory Office. Refer to the CTSU logistical appendix for specific instructions and documents to be submitted.
- **Patient enrollments** will be conducted by the CTSU. Refer to the CTSU logistical appendix for specific instructions and forms to be submitted.
- Data management will be performed by the RTOG. **Case report forms** (with the exception of patient enrollment forms)**, clinical reports, and transmittals** must be sent to RTOG unless otherwise directed by the protocol. Do not send study data or case report forms to the CTSU Data Operations.
- **Data query and delinquency reports** will be sent directly to the enrolling site by the RTOG. Please send query responses and delinquent data to the RTOG and do not copy the CTSU Data Operations. Each site should have a designated CTSU Administrator and Data Administrator and must keep their CTEP AMS account contact information current. This will ensure timely communication between the clinical site and RTOG Headquarters.

The following Cooperative Group has endorsed this trial: (8/12/08)
ECOG: ECOG members will enroll patients to this study via the Cancer Trials Support Unit (CTSU).

(6/22/10)

CANCER TRIALS SUPPORT UNIT (CTSU) ADDRESS AND CONTACT INFORMATION

To submit site registration documents:	For patient enrollments:	Submit study data directly to the Lead Cooperative Group unless otherwise specified in the protocol:
CTSU Regulatory Office<br>1818 Market Street, Suite 1100<br>Philadelphia, PA 19103<br>Phone – 1-866-651-CTSU<br>Fax – 215-569-0206	Please refer to Section 5.0 of the protocol for instructions on using the OPEN system.	RTOG Headquarters<br>1818 Market Street, Suite 1600<br>Philadelphia, PA 19103<br><br>Please do not submit study data or forms to CTSU Data Operations. Do not copy the CTSU on data submissions.

The **study protocol and all related forms and documents** must be downloaded from the protocol-specific web page of the CTSU Member Web site located at https://www.ctsu.org. Sites must use the current form version and adhere to the instructions and submission schedule outlined in the protocol.

CTSU sites should follow procedures outlined in the protocol for Site registration, Patient Enrollment, Adverse Event Reporting, Data Submission (including ancillary studies), and Drug Procurement.

For patient eligibility or treatment-related questions Contact the Study PI of the Coordinating Group

For questions unrelated to patient eligibility, treatment, or data submission contact the CTSU Help Desk by phone or e-mail:
CTSU General Information Line – 1-888-823-5923, or ctsucontact@westat.com. All calls and correspondence will be triaged to the appropriate CTSU representative.

For detailed information on the regulatory and monitoring procedures for CTSU sites please review the CTSU Regulatory and Monitoring Procedures policy located on the CTSU members' website https://www.ctsu.org

The CTSU Web site is located at https://www.ctsu.org

INDEX (6/22/10)

Schema

Eligibility Checklist

1.0 Introduction

2.0 Objectives

3.0 Patient Selection

4.0 Pretreatment Evaluations/Management

5.0 Registration Procedures

6.0 Radiation Therapy

7.0 Drug Therapy

8.0 Surgery

9.0 Other Therapy

10.0 Tissue/Specimen Submission

11.0 Patient Assessments

12.0 Data Collection

13.0 Statistical Considerations

References

Appendix I - Sample Consent Form
Appendix II - Study Parameters
Appendix III - Performance Status Scoring
Appendix IV - Staging System
Appendix V - Biospecimen Collection Instructions

RADIATION THERAPY ONCOLOGY GROUP

RTOG 0436

A PHASE III TRIAL EVALUATING THE ADDITION OF CETUXIMAB TO PACLITAXEL, CISPLATIN, AND RADIATION FOR PATIENTS WITH ESOPHAGEAL CANCER WHO ARE TREATED WITHOUT SURGERY

SCHEMA (5/3/12)

STRATIFY		RANDOMIZE	
	**Histology:**<br>~~1. Adenocarcinoma~~<br>(**Closed 5/23/2012**)<br><br>1. Squamous<br><br>**Cancer lesion size:**<br>1. < 5 cm<br>2. ≥ 5 cm<br><br>**Celiac nodes:**<br>1. Present<br>2. Absent		**Arm 1:**<br>Radiation Therapy + Paclitaxel + Cisplatin + Cetuximab<br><br>**Arm 2:**<br>Radiation Therapy + Paclitaxel + Cisplatin

Institution must demonstrate ability to digitally submit radiation therapy data (See Section 5.0).

<u>Patient Population</u>: (See Section 3.0 for Eligibility) (**5/3/12**)
Histologic proof of primary squamous cell ~~or adenocarcinoma~~ of the esophagus or gastroesophageal junction **(accrual of adenocarcinoma patients closed 5/23/2012)**
Involvement of the GE junction with Siewert type I or II tumors
Stage T1N1M0; T2-4, Any N, M0; Any T, Any N, M1a

Required Sample Size: 420

RTOG Institution # ________

RTOG 0436 **<u>ELIGIBILITY CHECKLIST</u>** (5/3/12)

Case # ________ (page 1 of 3)

_______ (Y) 1. Does the patient have a pathologically (histologic or cytologic) proven diagnosis (via endoscopy w/ biopsy or cytology by FNA per Section 3.1.2.4 of the protocol) of primary squamous cell ~~or adenocarcinoma~~ of the esophagus or gastroesophageal junction within 12 weeks of registration? **(accrual of adenocarcinoma patients closed 5/23/2012)**

_______ (Y) 2. Is all disease encompassed in the radiotherapy field?

_______ (Y) 3. Does the patient meet the staging requirements in Section 3.1.2?

_______ (Y) 4. Were history/physical exam, PET/PET-CT scan or chest/abdominal CT scan, and EKG performed within 6 weeks of study entry?

_______(Y) 5. Is the patient ≥ 18 and < 75 years of age?

_______(Y) 6. Is the patient's Zubrod performance status 0-2?

_______(Y) 7. Do the patient's laboratory values meet the criteria in Section 3.0?

_______(Y) 8. Is the patient's total caloric intake ≥ 1500 kCal/day?

_______(Y) 9. Is the patient willing to practice adequate contraception while on study (women of childbearing potential and men)?

_______(N) 10. Is there evidence of tracheoesophageal fistula or invasion into the major bronchi?

_______(N) 11. Has the patient had prior chemotherapy for esophageal cancer?

_______(N) 12. Has the patient had prior radiation therapy that would result in overlap of planned radiation therapy fields?

_______(N) 13. Has the patient had prior therapy that directly targets the EGFR pathway?

_______(N) 14. Has the patient had prior platinum-based and/or paclitaxel-based therapy?

_______(N) 15. Has the patient had a prior allergic reaction to the study drugs involved in this protocol or a prior severe infusion reaction to a monoclonal antibody?

_______(N) 16. Does the patient have significant infection or other coexistent medical condition that would preclude protocol therapy (as outlined in Section 3.2)?

_______(N) 17. Is the patient pregnant or lactating?

_______(N/Y) 18. Has the patient had prior malignancies, except for non-melanomatous skin cancers?

_______(Y) If yes, has the patient been disease free for ≥ 2 years?

RTOG Institution # ________

RTOG 0436 **<u>ELIGIBILITY CHECKLIST</u> (6/30/08, 6/22/10)**

Case # ________

<u>The following questions will be asked at Study Registration:</u>

____________	1.	Institutional person randomizing case
____________(Y)	2.	Has the Eligibility Checklist been completed?
____________(Y)	3.	In the opinion of the investigator, is the patient eligible?
____________	4.	Date informed consent signed
____________	5.	Participant initials (FML)
____________	6.	Verifying Physician
____________	7.	Patient’s ID
____________	8.	Date of Birth
____________	9.	Race
____________	10.	Ethnicity
____________	11.	Gender
____________	12.	Country of Residence
____________	13.	Zip Code (U.S. Residents)
____________	14.	Method of payment
____________	15.	Any care at VA or military hospital?
____________	16.	Calendar Base Date
____________	17.	Randomization date

(Continued on the next page)

RTOG 0436 **ELIGIBILITY CHECKLIST** (5/3/12)

Case # ________ (page 3 of 3)

______ 18. Medical Oncologist's name

______(Y/N) 19. Did patient provide written consent/tissue specimens for research?

______(Y/N) 20. Initial consent given for specimen use unrelated to patient's cancer?

______(Y/N) 21. Did patient consent to future contact about more research?

______ 22. Histologic type (~~adenocarcinoma~~ ~~or~~ squamous cell carcinoma) **(accrual of adenocarcinoma patients closed 5/23/2012)**

______ 23. Cancer lesion size (< 5 cm or ≥ 5 cm)

______ 24. Celiac nodal status (present or absent)

______(Y/N) 25. Patient has consented to take part in the quality of life study?

______ If no, please provide the reason:
1. Patient refused due to illness
2. Patient refused for other reason: specify ______
3. Not approved by institutional IRB
4. Tool not available in patient's language
5. Other, specify________

The Eligibility Checklist must be completed in its entirety prior to web registration. The completed, signed, and dated checklist used at study entry must be retained in the patient's study file and will be evaluated during an institutional NCI/RTOG audit.

Completed by ______________ Date ______________

1.0 INTRODUCTION

1.1 Nonoperative Chemoradiation for Esophageal Cancer: Cisplatin/5-Fluorouracil (5-FU)/Radiation

Patients presenting with carcinoma of the esophagus continue to represent a significant therapeutic challenge. Despite the fact that the disease represents only 4% of all cancers in the United States with approximately 13,000 new cases diagnosed this year, more than 13,000 people will die annually as result of the aggressive biologic behavior of this illness. Over the past 20 years, the incidence rate has increased nearly 15%. Patients often present with locally advanced disease, and up to 40% of patients will be diagnosed with evidence of metastatic disease.[1]

There are 2 conventional treatments for localized esophageal cancer: surgery and non-operative combined chemotherapy plus radiation. Over the past 2 decades, well-designed clinical trials have documented the benefits of combination of chemotherapy and radiation, either as primary therapy or in the neoadjuvant setting. This expanded role has led to a greater understanding of the importance of increased tumor control and simultaneous decrease in normal tissue complications.

The Radiation Therapy Oncology Group completed a phase III randomized trial comparing daily radiation to a total dose of 64 Gy versus concurrent chemotherapy and radiation consisting of 2 cycles of 5-fluorouracil (5-FU), cisplatin and 50 Gy (RTOG 85-01). Patients randomized to receive combination therapy also received two cycles of adjuvant chemotherapy. A total of 129 patients were enrolled between 1986 and 1990. When an interim analysis revealed a difference in outcome that satisfied the early stopping rules, the study was closed. An additional 73 patients who would have been eligible for participation were prospectively registered and treated with combined modality therapy. The median survival was 8.9 months in the radiation-alone group compared with 12.5 months in patients treated with chemotherapy and radiation. The 5-year survival was 26% for those who received combination therapy. None of the patients treated with radiation alone were alive at the time of the final analysis.[2,3]

The use of combined chemoradiation helped to improve the local control of regional disease compared with radiation alone. The results of RTOG 85-01 revealed that 40 percent of patients who received radiation alone had persistent disease, and an additional 24% had local failure as the first site of recurrence, compared with 27% and 17% respectively for patients who received combined therapy ($p < 0.01$). One year following therapy, the rate of persistent or recurrent esophageal disease was 62% versus 44% in favor of chemoradiation ($p = 0.01$). The improvement in local control was also associated with a lower risk of distant failure (38% vs 22%, $p < 0.005$). The basic hypothesis of this trial was that combination chemotherapy administered concurrently would act as a promotor of the local-regional effects of radiotherapy as well as have a direct cytoxic effect on its own. The improved local control seen with combination therapy supports this theory, especially in light of the variation in radiation dose between the 2 arms (50 Gy versus 64 Gy). It also appears that systemic therapy eradicated some subclinical distant metastases, since distant failure accounted for the first site of failure in 16% of patients in the radiation-only group versus 8% in the combined modality group. This difference was maintained at a follow-up of 5 years; these findings were considered significant, since patients treated with chemoradiation lived longer and therefore had a longer time to develop distant metastases.[3]

Given the patterns of failure that were observed in trials such as RTOG 8501, researchers began to investigate ways of improving the local-regional control by intensifying the radiotherapy dose. The potential benefits of external beam radiation dose escalation were evaluated in a phase III intergroup trial (INT 0123). Patients were randomized to concurrent chemotherapy and radiotherapy 50.4 Gy versus the same chemotherapy and 64.8 Gy. The chemotherapy agents (5-FU and cisplatin) were the same in the two studies with only minor differences in the delivery schedule.[4]

This study was closed after a planned interim analysis revealed little chance of identifying any significant differences in survival between the 2 groups. The 2-year overall survival between the treatment arms (31% versus 40%) and locoregional failure (56% versus 52%) was not significantly different. The equivalence in survival between the 2 groups was clearly influenced

by a disproportionate number of deaths among those patients on the dose escalation arm (11 deaths versus 2). It is important to note that this finding is often misunderstood to suggest a detrimental effect associated with dose escalation beyond 50 Gy. However, a majority of deaths on the high-dose arm occurred in patients during therapy and before receiving a cumulative dose of greater than 50 Gy. This phase III trial helped to confirm the efficacy of the concurrent chemotherapy and radiation schedule that was defined in RTOG 85-01. The dose of 50.4 Gy remains the standard in current combined modality treatment strategies. In these trials, there was no survival difference among patients with adenocarcinoma and squamous cell cancer.[4]

The results from phase III chemoradiation trials such as RTOG 85-01 and INT 0123 defined a standard of care that is broadly applied to patients diagnosed with esophageal cancer. While the survival results certainly improved upon the outcomes achieved with single-modality therapy, it is widely recognized that there is significant room for improvement. Up to 75% of patients diagnosed with locally advanced disease will ultimately have evidence of distant metastases. In an attempt to address this substantial risk, investigators have created treatment regimens that incorporate induction chemotherapy prior to definitive chemoradiation.

For the last 30 years concurrent chemoradiation strategies have incorporated 5-FU and cisplatin. The documented response data and the radiosensitizing properties of these agents have supported their routine clinical use. At the same time, researchers have sought to incorporate novel systemic agents that ideally would improve response rates and survival while simultaneously decreasing the toxicity profile associated with the traditional 5-FU/cisplatin combination.[5-8]

1.2 Bimodality Treatment Versus Trimodality Treatment in Esophageal Cancer

Multiple concurrent chemoradiation trials have documented 5-year overall survivals in 20% to 25% of treated patients.[2,4] While there are no direct comparisons in randomized trials, these results are certainly similar to those achieved by surgery alone. Based on broad utilization of concurrent chemoradiation strategies, many researchers recognized the importance of defining the role of surgery within the context of a combined modality treatment approach.

Recently, 2 European randomized trials have been reported that attempted to answer this question. Bundenne and colleagues[9] presented the results of a phase III randomized trial designed to compare definitive chemoradiation versus neoadjuvant concurrent therapy followed by surgical resection. This study included 455 patients presenting with potentially resectable T3-4, N0-1 esophageal carcinomas. All patients received 2 cycles of 5-FU and cisplatin with 46 Gy (2 Gy per day, 23 fractions). Patients who achieved at least a partial response were then randomized to completion of definitive chemoradiation (3 more cycles of chemotherapy and an additional 20 Gy) or surgical resection. Two hundred and fifty-nine patients were eligible for randomization.

The median survival was 19.3 months for patients receiving definitive chemoradiation versus 17.7 months for those undergoing trimodality therapy. The 2-year overall survival was 40% for definitive chemoradiation versus 34% with trimodality therapy (not statistically significant). The treatment-related mortality (death within 3 months of starting therapy) was significantly higher in patients undergoing surgery after chemoradiation (9% vs. 1%, $p = 0.002$) compared with those receiving chemoradiation. Given the equivalence in survival and the additional mortality associated with trimodality therapy, the authors concluded that definitive chemoradiation was a viable alternative to surgical resection in patients achieving a response to upfront therapy.[9]

Most recently, Stahl[10] published the results of a phase III randomized trial that included 177 patients who had evidence of operable T3-4, N0-1 disease documented by CT and endoscopic ultrasound. After randomization, all patients received 3 cycles of 5-FU, leucovorin, etoposide, and cisplatin. Patients went on to definitive chemoradiation with > 66 Gy, cisplatin, and etoposide or 40 Gy, cisplatin, and etoposide followed by surgical resection. The median survival of 16 months and the 3-year overall survival of 28% among patients receiving trimodality therapy were not significantly different than the 15 months and 20% achieved by patients treated with definitive chemoradiation. Trimodality therapy was associated with a significant improvement in local control, with a 2-year local progression-free survival of 64% versus 40%; however, this improvement did not translate into a survival advantage for these patients.

Taken together, these 2 phase III randomized trials now serve as the only level 1 data sets that attempt to define the benefit of the addition of surgery to a concurrent chemoradiation treatment scheme. Regardless of one's perspective, it must be recognized that both trials defined the standard arm in the trial design to be concurrent chemoradiation. These facts highlight the need to identify concurrent chemoradiation strategies that will improve upon the results historically achieved with previous treatment regimens.

1.3 Paclitaxel-Based Chemoradiation for Esophageal Cancer

Paclitaxel has important single-agent activity in squamous cell and adenocarcinoma of the esophagus and is a radiation sensitizer.[7,11-13] There have been multiple phase II studies evaluating paclitaxel-based chemoradiation in esophageal cancer.[5,14-17] These trials demonstrated similar complete response rates and survival to cisplatin/5-FU/radiation regimens. Phase II studies from Memorial Sloan Kettering and the Brown University Oncology Group demonstrate substantially less esophagitis (< 5% grade 4 esophagitis) with the regimen of cisplatin, paclitaxel, and radiation without the need for prophylactic enteral feeding tubes.[5,14,15] Weekly paclitaxel/cisplatin/radiation regimens do not require central venous catheter devices when paclitaxel is administered by weekly 1-hour infusion compared to continuous-infusion 5-FU–based regimens. Clinical complete responses in sequential phase trials of weekly paclitaxel/cisplatin/radiation and paclitaxel/cisplatin/trastuzumab/radiation have achieved clinical complete responses of 35% to 47%.[5,14]

Paclitaxel-based chemoradiation has been the framework for the recent RTOG trials of non-operative management of esophageal cancer. RTOG 0113 evaluated 2 different paclitaxel-containing regimens. Seventy-six patients were accrued to this study; 63% had adenocarcinoma and 37% had squamous cell cancer. Patients in group 1 received induction 5-FU, cisplatin, and paclitaxel followed by radiation and concurrent continuous infusion 5-FU and weekly paclitaxel. Patients in group 2 received induction paclitaxel and cisplatin followed by radiation and concurrent weekly cisplatin and 96-hour infusion of paclitaxel. Both arms had similar toxicities. In group 1, there were 26% grade 4 toxicities and 5% grade 5 toxicities. In group 2, there were 40% grade 4 toxicities. The increased toxicities observed in this study were in part due to the intensive induction chemotherapy regimens in both arms. Furthermore, arm 2 had a more myelotoxic 96-hour weekly paclitaxel infusion with weekly cisplatin and concurrent radiation. There were no significant differences in activity. Group 1 had a 44% endoscopic (clinical) complete response rate and 76% 1-year survival. Group 2 had a 35% endoscopic complete clinical response and 67% 1-year survival.

The decision to utilize the 2-drug regimen of paclitaxel and cisplatin with daily radiation was made based on the understanding that various combinations and schedules of cisplatin, 5-FU, paclitaxel and irinotecan have not demonstrated substantive differences in efficacy.[18] For example, in a study by Urba et al of the 3-drug regimen of 5-FU, cisplatin, paclitaxel and concurrent radiation the pathologic complete response rate was 17%.[19] Recent trials have reported disappointingly low complete response rates with the doublet of cisplatin and 5-FU.[18,20] A study by Burmeister et al published this year in the *Lancet* reported a pathologic complete response of 10%.[20] ECOG 1201 compared the doublets of irinotecan/cisplatin to paclitaxel/cisplatin combined with 45 Gy radiation; pathologic complete response rates were 14% and 13%, respectively. For irinotecan/cisplatin/radiation the rates of grade 3, 4, and 5 toxicity were 45%, 29%, and 2%, respectively. For paclitaxel/cisplatin/radiation the rates of grade 3, 4, and 5 toxicity were 35%, 47%, and 2%, respectively. RTOG 0426 evaluated induction 5-FU, cisplatin, paclitaxel and granulocyte colony-stimulating factor (G-CSF) followed by concurrent 5-FU, cisplatin and radiation followed by surgical salvage. Response data are currently not available. Grade 3, 4, and 5 toxicity occurred in 55%, 15%, and 3% of patients, respectively.

Conventional 2-drug chemoradiation regimens appear to be equal to 3 drugs as a framework for adding biologic therapy. Since mucosal toxicity from chemoradiation is primarily from 5-FU,[18] a non–5-FU regimen was chosen to test the potential benefits of the addition of cetuximab. Furthermore, as described below, preclinical data suggest that cetuximab may have more synergy with taxanes and platinum than 5-FU.

1.4 Salvage Esophagectomy in Previous RTOG Studies

Surgical salvage has not affected the survival in RTOG studies of non-operative management of esophageal cancer. These studies were intended to accrue patients with esophageal cancer who were not candidates for surgical resection, such as patients with advanced age, comorbid medical conditions, and proximal esophageal cancers and those who choose to be treated non-operatively. The percentage of patients undergoing salvage surgery in previous RTOG studies has been < 10% and has not affected study outcome.[4]

1.5 EGFR Inhibitors in Esophageal Cancer

The current limitations of most systemic cytotoxic agents are often defined by the nonspecific toxicities of healthy tissues. Driven by the need to improve the therapeutic ratio, investigators have focused their interests on newer biologic agents targeting cellular protein receptors. These receptors are now known to be involved in growth regulation, angiogenesis, inflammation cell cycle control, metastatic potential, and sensitivity to systemic therapy. These agents are now being incorporated into concurrent chemoradiation approaches in an attempt to improve both local and distant disease control.

Therapies that block aberrant growth factor signal transduction pathways have substantial promise in esophageal cancer. Epidermal growth factor receptor (EGFR) is a tyrosine kinase cell surface receptor encoded by the c-erbB-1 protooncogene.[21] EGFR is a member of the erb B receptor tyrosine kinase family that includes erb B-2, erb B-3, and erb B-4. Among the known natural ligands of the EGFR are epidermal growth factor (EGF) and transforming growth factor α (TGF-α), ligands that activate the receptor by binding to the extracellular domain and inducing the formation of receptor homodimers or heterodimers, followed by internalization of the receptor/ligand complex and autophosphorylation. It is now accepted that the EGFR signal transduction network plays an important role in multiple tumorigenic processes, including cell cycle progression, angiogenesis, and metastasis, as well as protection from apoptosis.[22]

EGFR is expressed in between 50% and 80% of all esophageal cancers,[8,23,24] and its expression is associated with poor prognosis.[8,24-26] Accumulating clinical evidence suggests that EGFR represents a viable target in the treatment of esophageal cancer. The EGFR tyrosine kinase inhibitors gefitinib and erlotinib achieve response rates of 2% to 13% in esophageal squamous cell and adenocarcinoma.[27-29] In studies from the Southwest Oncology Group (SWOG) and Memorial Sloan Kettering, EGFR mutations were not detected, including from tumor tissue from one patient responding to erlotinib.[28,29]

1.5.1 Cetuximab

Cetuximab, an IgG1 chimerized, monoclonal antibody, binds specifically to EGFR on both normal and tumor cells and competitively inhibits the binding of EGF and other ligands, such as TGF-α.[30] Binding of cetuximab to EGFR blocks phosphorylation and activation of receptor-associated kinases, resulting in inhibition of cell growth, induction of apoptosis, and decreased matrix metalloproteinase and vascular endothelial growth factor production. SWOG evaluated cetuximab in a second-line, single-agent, phase II study. The primary endpoint was 6-month overall survival. The study had a 2-step design, initially evaluating for 9 or more 6-month survivors out of the first 30 patients. Ten of 30 patients survived at least 6 months. The study has reopened to complete accrual of 55 patients.

1.5.1.1 *Clinical Studies of Cetuximab With Radiation for Head and Neck Cancer*

The efficacy and safety of cetuximab in combination with radiation therapy were studied in a randomized controlled trial of 424 patients with locally or regionally advanced squamous `cell carcinoma of the head and neck (SCCHN) versus radiation therapy alone.[31] Patients with stage III/IV SCC of the oropharynx, hypopharynx, or larynx with no prior therapy were randomized 1:1 to receive cetuximab plus radiation therapy (211 patients) or radiation therapy alone (213 patients). Stratification factors were Karnofsky performance status (60-80 versus 90-100), nodal stage (N0 versus N+), tumor stage (T1-3 versus T4 using American Joint Committee on Cancer [AJCC] 1998 staging criteria), and radiation therapy fractionation (concomitant boost versus once daily versus twice daily). Radiation therapy was administered from 6 to 7 weeks as once daily, twice daily, or concomitant boost. The planned radiation therapy regimen was chosen by the investigator prior to enrollment. For

patients with > N1 neck disease, a post-radiation therapy neck dissection was recommended. Starting 1 week before radiation, cetuximab was administered as a 400-mg/m^2 initial dose, followed by 250 mg/m^2 weekly for the duration of radiation therapy (6-7 weeks). All cetuximab-treated patients received a 20-mg test dose on day 1. Cetuximab was administered 1 hour before radiation therapy, beginning at week 2.

Of the 424 randomized patients, 80% were male and 83% were Caucasian. The median age was 57 years (range 34-83). There were 258 patients enrolled in US sites (61%) and 166 patients (39%) in non-US sites. Ninety percent of patients had baseline Karnofsky performance status ≥ 80; 60% had oropharyngeal, 25% laryngeal, and 15% hypopharyngeal primary tumors; and 28% had AJCC T4 tumor stage. Patient characteristics were similar across the study arms. Fifty-six percent of patients received radiation therapy with concomitant boost, 26% received a once-daily regimen, and 18% a twice-daily regimen.

The main outcome measure of this trial was duration of locoregional control. Overall survival was also assessed. Results are presented below:

Clinical Efficacy in Locoregionally Advanced SCCHN

	Cetuximab + Radiation (n = 211)	Radiation Alone (n = 213)	Hazard Ratio (95% CI)	Stratified Log-Rank p-value
Locoregional Control<br>Median Duration	24.4 mo	14.0 mo	0.68 0.52-0.89)	0.005
Overall Survival<br>Median duration	49.0 mo	29.3 mo	0.74 (0.57-0.97)	0.03

Cl = confidence interval.

1.5.1.2 *Toxicities Associated With Cetuximab/Radiation in SCCHN*

The most serious adverse reactions associated with cetuximab in combination with radiation therapy in patients with head and neck cancer were:

- Infusion reaction (3%)
- Cardiopulmonary arrest (2%)
- Dermatologic toxicity (2.5%);
- Mucositis (6%);
- Radiation dermatitis (3%);
- Confusion (2%);
- Diarrhea (2%).

Fourteen (7%) patients receiving cetuximab plus radiation therapy and 5 (5%) patients receiving cetuximab monotherapy discontinued treatment primarily because of adverse events. The most common adverse events seen in 208 patients receiving cetuximab in combination with radiation therapy were acneform rash (87%), mucositis (86%), radiation dermatitis (86%), weight loss (84%), xerostomia (72%), dysphagia (65%), asthenia (56%), nausea (49%), constipation (35%), and vomiting (29%). The most common adverse events seen in 103 patients receiving cetuximab monotherapy were acneform rash (76%), asthenia (45%), pain (28%), fever (27%), and weight loss (27%).

The data in the table below are based on the experience of 208 patients with locoregionally advanced SCCHN treated with cetuximab plus radiation therapy compared with 212 patients treated with radiation therapy alone.

Incidence of Selected Adverse Events (≥ 10%) in Patients with Locoregionally Advanced SCCHN

Body System<br>Preferred Term	Cetuximab plus Radiation (n=208)		Radiation Therapy Alone (n=212)	
	Grades 1-4	Grades 3 and 4	Grades 1-4	Grades 3 and 4
	% of Patients			
Body as a Whole				
Asthenia/Malaise	56	4	48	5
Fever[1]	29	1	13	<1
Headache	19	<1	8	<1
Infusion Reaction[2]	15	3	2	0
Infection	13	1	9	1
Chills[1]	16	0	5	0
Digestive				
Mucositis/Stomatitis	93	56	94	52
Xerostomia	72	5	71	3
Dysphagia	65	26	63	30
Nausea	49	2	37	2
Constipation	35	5	30	5
Vomiting	29	2	23	4
Anorexia	27	2	23	2
Diarrhea	19	2	13	1
Dyspepsia	14	0	9	1
Metabolic/Nutritional				
Weight Loss	84	11	72	7
Dehydration	25	6	19	8
Respiratory				
Pharyngitis	26	3	19	4
Cough Increased	20	<1	19	0
Skin/Appendages				
Acneform Rash[3]	87	17	10	1
Radiation Dermatitis	86	23	90	18
Application Site Reaction	18	0	12	1
Pruritus	16	0	4	0

1 Includes cases also reported as infusion reactions
2 Infusion reaction is defined as any event described at any time during the clinical study as "allergic reaction" or "anaphylactoid reaction" or any event on the first day of dosing described as "allergic reaction," "anaphylactoid reaction," "fever," "chills," "chills and fever," or "dyspnea".
3 Acneform rash as defined as any event described as "acne," "rash," "maculopapular rash," "pustular rash," "dry skin," or "exfoliative dermatitis."

Cardiac arrest and/or sudden death occurred in 2% (4/208) of patients treated with radiation therapy and cetuximab compared with none of 212 patients treated with radiation therapy alone. Fatal events occurred within 1 to 43 days after the last cetuximab treatment. Cetuximab in combination with radiation therapy should be used with caution in head and neck cancer patients with known coronary artery disease, congestive heart failure, and arrhythmias. Although the etiology of these events is unknown, close monitoring of serum electrolytes— including serum magnesium, potassium, and calcium—during and after cetuximab therapy is recommended.

The cardiac safety of cetuximab in combination with radiation therapy and cisplatin has not been established. Death and serious cardiotoxicity were observed in a 21-patient single-arm study of patients with locally advanced SCCHN. Patients received cetuximab, delayed accelerated (concomitant boost) fractionation radiation therapy, and cisplatin (100 mg/m^2).

In this randomized controlled trial, cardiopulmonary arrest and/or sudden death occurred in 4/208 patients (2%) treated with radiation therapy and cetuximab compared to none of 212 patients treated with radiation therapy alone. Three patients with prior history of coronary artery disease died at home, with myocardial infarction as the presumed cause of death. One of these patients had arrhythmia and one had congestive

heart failure. Death occurred 27, 32, and 43 days after the last dose of cetuximab. One patient with no prior history of coronary artery disease died 1 day after the last dose of cetuximab.

The overall incidence of late radiation toxicities (any grade) was higher in cetuximab in combination with radiation therapy compared with radiation therapy alone. The following sites were affected: salivary glands (65% versus 56%), larynx (52% versus 36%), subcutaneous tissue (49% versus 45%), mucous membrane (48% versus 39%), esophagus (44% versus 35%), skin (42% versus 33%), brain (11% versus 9%), lung (11% versus 8%), spinal cord (4% versus 3%), and bone (4% versus 5%). The incidence of grade 3 or 4 late radiation toxicities was generally similar between the radiation therapy alone and the cetuximab plus radiation treatment groups.

1.5.1.3 *Cetuximab With Chemoradiation*

Preclinical models have suggested synergy between cetuximab, paclitaxel, cisplatin, and radiation.[32,33] Substantial toxicity was initially reported in the first phase II trial of 22 patients treated with cetuximab, cisplatin, and concurrent radiation in head and neck cancer.[34] There were 2 deaths on this trial [pneumonia (1) and unknown cause (1)]. In addition, grade 4 toxicities included anaphylaxis (1), anorexia (2), arrhythmia (1), bacteremia (1), hypokalemia (1), hyponatremia (1), myocardial infarction (1), and mucositis (2). This study used high-dose cisplatin (100 mg/m^2) and concomitant boost radiotherapy (1.8 Gy/day weeks 1-6; boost: 1.6 Gy 4-6 hours later weeks 5-6). However, it was unclear whether the toxicities were related to high-dose cisplatin and concomitant boost radiation to 70 Gy or to the addition of cetuximab. Therefore, the Brown University Oncology Group and the University of Maryland Cancer Center piloted the addition of cetuximab with chemoradiation for patients with esophagogastric cancer.[35] A poster discussion of this trial was presented at the 2006 annual meeting of the American Society of Clinical Oncology. The objective of this trial was to determine the safety of cetuximab with chemoradiation. A secondary objective was to determine clinical complete response, defined as no tumor on post-chemoradiation endoscopic biopsy. Carboplatin was utilized instead of cisplatin to attempt to distinguish whether non-hematologic toxicities were due to the addition of cetuximab or from standard chemoradiation.

In the Brown University/University of Maryland pilot study, patients received cetuximab 400 mg/m^2 for week 1, then 250 mg/m^2/week for 5 weeks; paclitaxel 50 mg/m^2/week; and carboplatin area under the curve (AUC) = 2 weekly for 6 weeks with concurrent 50.4 Gy radiation. Patients were required to have pathologically confirmed esophagogastric cancer. Since toxicity assessment was the most important objective, patients were allowed to have locally advanced disease if it could be contained in a radiotherapy field, including mediastinal, celiac, periportal, regional gastric, and retroperitoneal lymphadenopathy. Thus patients in this pilot trial could have substantially larger and more advanced disease that in RTOG 0113. Patients with distant organ metastases were not eligible. Required staging studies included a CT of the chest, abdomen, and pelvis and an endoscopy. Endoscopic ultrasound and PET scans were not required. All patients were required to have post-treatment endoscopy to assess clinical complete response. Since this protocol was designed to pilot a future non-surgical trial, patients could have comorbid medical conditions preventing surgery or extensive adenopathy at diagnosis for which the addition of surgery would not likely increase the potential for cure. Therefore, patients were not required to have surgical resection or determination of pathologic response following completion of chemoradiation.

Fifty-three patients have completed treatment. The median age was 58 (range 30-87). Fifty patients had esophageal cancer and 3 had proximal gastric cancer. Forty-two patients had adenocarcinoma and 11 had squamous cell cancer. Toxicities are shown below. Two patients had anaphylaxis to cetuximab and were not evaluable for further toxicity or response. Two patients had anaphylaxis to paclitaxel and continued treatment with cetuximab and carboplatin and radiation. Only 2 of 51 patients (4%) had grade 4 esophagitis. There have been no other grade 4 non-hematologic toxicities, and 2 patients had grade 4 neutropenia (4%). Seven of 51 patients (14%) had grade 3 esophagitis. Other grade 3 toxicities included dehydration in 10 patients (20%). Thirty-nine of 51 patients (76%) have had a clinical complete response. Of the first 51 assessable patients, 8 patients were not surgical candidates at study entry and 2 had not yet undergone surgery. Thirteen of 41 (32%) achieved a pathologic complete response. The 1-year survival rate was 70%, a rate that is comparable to the 2 intensive regimens used in RTOG 0113 (1-year survival of 67-76%). The cetuximab regimen had much less toxicity, did

not use intensive induction chemotherapy, and used much simpler chemoradiation regimens without the need for continuous infusion compared to RTOG 0113. Furthermore, in the cetuximab pilot trial, 40% of the patients with adenocarcinoma had more advanced disease (retroperitoneal, perigastric, or portal lymph nodes) that made them prone to systemic relapse. These patients would have been ineligible for RTOG 0113. The much higher endoscopic complete response rate with cetuximab (75%) versus the RTOG 0113 regimens (35%-40%) suggests that the addition of cetuximab to chemoradiation may result in more effective locoregional control and survival in patients with a comparable stage of esophageal cancer. Patients with SCC treated with cetuximab had similar staging to patients on RTOG 0113. The clinical complete response rate was 80%, and the 1-year survival rate was 80%.

All toxicities (N = 53). Multiple toxicities in the same patient are scored as separate events.

	Grade 2	Grade 3	Grade 4
Esophagitis	16	7	2
Cutaneous	15	13	0
Dehydration	14	10	0
Diarrhea	3	0	0
Fatigue/Anorexia	15	3	0
Nausea	4	2	0
LFTs	5	0	0
Magnesium	0	2	0
Anaphylaxis (paclitaxel)	0	2	2
Anaphylaxis (cetuximab)	1	1	2
Neutropenia	8	5	2
Anemia	5	4	1
Thrombocytopenia	3	0	0

The Brown University/University of Maryland trial used carboplatin instead of cisplatin to distinguish whether non-hematologic toxicities were due to cetuximab or chemoradiation. Carboplatin has similar activity to cisplatin when used with radiation[36]; however, cisplatin will be substituted in this protocol since previous cooperative group trials have used cisplatin. The FOLFOX/radiation regimen or cisplatin/5-FU/radiation could also be tested[34]; however, these regimens would seem equivalent to paclitaxel/cisplatin.[18] The weekly paclitaxel/cisplatin regimens may facilitate accrual since they would not require continuous home-infusion 5-FU. Furthermore, preclinical data suggest that cetuximab may have more synergy with taxanes and platinum than 5-FU.[32, 33]

1.6 Rationale for Proposed Study

This trial represents the next logical step in the progression of well-designed, non-surgical phase III trials for patients with locally advanced esophageal cancer. RTOG 85-01 helped to define the standard of care as combined chemotherapy and radiation. Intergroup 0123 demonstrated that radiotherapy dose escalation did not significantly improve overall survival. Improvements in chemoradiation are needed to improve local control and long-term survival. RTOG 0113 demonstrates the activity and safety of paclitaxel-based chemoradiation in esophageal cancer. To improve on these results, we have opted to investigate the addition of a biologic therapy to the cisplatin, paclitaxel, and radiation.

Paclitaxel/cisplatin/radiation was chosen given the excellent tolerance, ease of administration, and comparable treatment outcome for this regimen compared to the historic 5-FU and cisplatin combination. As described above, in the Brown University Oncology Group study of 41 patients treated with weekly cisplatin, paclitaxel, and radiation for esophageal cancer, grade 3 neutropenia was the most common significant toxicity. However, there were no episodes of grade 4 neutropenia or grade 3/4 thrombocytopenia. Grade 3 esophagitis occurred in 5 patients (12%) and grade 4 esophagitis occurred in only 2 patients (5%). Prophylactic enteral feeding tubes were not necessary. Grade 3 dehydration occurred in 17% of patients. There were no grade 3 or 4 pulmonary toxicities.[14] The RTOG and the Gastrointestinal Cancer Steering Committee decided to add cetuximab to cisplatin, paclitaxel, and radiation instead of to carboplatin, paclitaxel, and radiation, as utilized in the Brown University pilot, since the majority of previous cooperative group studies have utilized cisplatin. Low-dose weekly cisplatin and carboplatin, when used as

radiosensitizers, have similar toxicity profiles.[14,36] However, to ensure patient safety, early assessments for toxicity will be performed after 25 and 50 patients, as described in Section 13.4.

The chemotherapy platform to build upon must have an acceptable underlying toxicity profile, and paclitaxel/cisplatin represents a good candidate regimen to augment the addition of targeted therapies. The encouraging data on the combination of cetuximab and radiation in head and neck cancer provides us with a unique opportunity to introduce a biologic agent into the chemoradiation paradigm. If the addition of cetuximab improves survival when combined with chemoradiation, it will change the standard of care of patients with locoregional esophageal cancer treated with chemoradiotherapy.

This trial will build upon the previous phase III trials run by the RTOG that focus on the non-operative management of patients with esophageal cancer. The target population of this study will reflect those who have been accrued to previous studies such as INT 0123 and RTOG 0113. This protocol will allow patients with either Supraclavicular or celiac adenopathy, gastric invasion, and/or mediastinal adenopathy. These patients have not been eligible for previous cooperative group operable studies such as RTOG 0246 and CALGB 9781. We will include patients with either squamous cell or adenocarcinoma histologies; however, a separate interim evaluation of efficacy will be performed (as outlined in Section 10) to evaluate any potential response differences that may be seen with cetuximab. The patient population is expected to fall into the following categories:

1.6.1 Squamous Cell Carcinomas (SCCs)

Worldwide, esophageal cancer is among the most common causes of cancer death, with a staggering incidence across parts of central Asia and Africa.[1,37] These include regions that are not included in cancer registries, so the worldwide incidence is markedly underreported. There were approximately 7,400 cases of SCC of the esophagus in the United States in 2006.[37] Proximal esophageal cancers are generally not operable. Comorbidities from alcohol and tobacco use often make these patients with midesophageal squamous cell cancers poor surgical candidates.

1.6.2 Adenocarcinomas in Patients With Medical Contraindications to Resection

Following the presentation by Tepper et al[38] showing improved survival for trimodality therapy compared to surgery, there is increasing interest in trimodality therapy for patients with esophageal cancer. However, many patients have medical contraindications to esophagectomy and others refuse to undergo this difficult, morbid, surgical procedure. Furthermore, as described above, the addition of surgery after chemoradiation has not been definitively proven to improve survival. A substantial number of patients with esophageal adenocarcinoma will choose non-operative treatment with chemoradiation. The definition of a novel agent such as cetuximab that could potentially increase locoregional control without an increase in mucosal toxicity would be a major advance.

1.7 Quality of Life and Health Utilities

The quality of survival, in addition to the length of survival, is now accepted by oncologists as an important clinical endpoint in phase III trial design for patients with locally advanced cancers.[39, 40] To date, there has been limited available literature using formal health-related quality of life (QOL) measures for patients with esophageal cancer receiving definitive chemoradiation on prospective trials. RTOG however has evaluated QOL for patients with localized esophageal cancer on a large randomized phase III effort.[41] RTOG 9405 (Intergroup 0123) compared the QOL outcomes for patients with esophageal cancer receiving chemoradiation with conventional dose radiation (50.4 Gy) versus high dose RT (64.8 Gy). QOL was assessed using the Functional Assessment of Cancer Therapy (FACT) Head & Neck (version 2)[42] at baseline, after chemoradiation, at 8 months after therapy, and at 1 year. Two-year outcome analysis showed no survival or local control benefit for the 64.8 Gy arm.[4] In terms of QOL, functional and swallowing scores were decreased after chemoradiation in both treatment arms, with total QOL scores significantly poorer than baseline in the 64.8 Gy arm.

One factor associated with the paucity of QOL data to assess treatment efficacy for esophageal cancer has been the lack of a validated QOL instrumented tailored to this patient population. The Functional Assessment of Cancer Therapy for Esophageal Cancer (FACT-E) questionnaire has recently been developed, been used prospectively,[43] and undergone validation.[44] This QOL instrument has been specifically designed for adults with esophageal cancer. The FACT-E self-

reporting scale is comprised of the validated FACT-G core (27 general items including the four domains of physical well-being, social and family well-being, emotional well-being, and functional well –being, which had been developed for adults with various cancer diagnosis[45, 46]), combined with the new FACT-E subscale (commonly referred to as the Esophageal Cancer Subscale or ECS), which includes 17 additional items specific for symptoms and problems related to esophageal cancer such as eating, appetite, swallowing, pain, talking/communicating, mouth dryness, breathing difficulty, coughing, and weight loss. The total FACT-E score is the sum of the esophageal-specific questions and the FACT-G scores.

Each FACT-E question has a possible 5-point response of 0-4 (i.e., not at all to very much). Negatively worded items are reverse scored so that higher scores always represent better QOL or less severe symptoms. The total questionnaire takes approximately 10 minutes to complete. As with the FACT-G, higher scores indicate a better health-related QOL or functioning. The FACT-E 44-item questionnaire has recently undergone psychometric testing in patients with esophageal cancer.[44] The FACT-E had good construct validity (convergence and divergence) when compared with the EORTC QLQ 30 and its specific esophageal module. It had very good to excellent internal consistency and reliability. FACT-E scores correlated well with several important clinical factors and were found to be responsive to change in patients treated with esophagectomy alone and in those treated with neoadjuvant chemoradiotherapy. In the subset of patients treated with neoadjuvant chemoradiotherapy, a significant improvement was reported in the ECS, swallowing subscale (Swallowing Index Subscale Score) and eating subscale (Eating Index Subscale Score) at 6 to 8 weeks following chemoradiation.

There are no published data to date demonstrating how QOL is affected by the addition of cetuximab to standard chemotherapeutic (and/or chemoradiation) regimens for the treatment of gastrointestinal malignancies. The addition of cetuximab to combined modality therapy in head and neck cancer documented both a survival and local control benefit associated with the combination of cetuximab and radiation.[31] Importantly, there was no significant increase in mucositis associated with the delivery of cetuximab and radiation. QOL data from this trial has not been presented. There is also accumulating clinical evidence that cetuximab targeted therapy produces significant local response rates in advanced esophageal squamous cell and adenocarcinoma.[47] Based on the impressive local response rates associated with the use of cetuximab (with minimal morbidity) and on data from Darling and colleagues,[44] we hypothesize that the addition of this new biologic, cetuximab, to standard chemoradiation for locally advanced esophageal cancer, will improve the FACT-E ECS score by at least 5 points (see 13.2.3.3.1 for power calculations). Secondary QOL endpoints will be to determine if the addition of cetuximab to standard chemoradiation improves the Swallowing Index Subscale Score and the Eating Index Subscale Score of the FACT-E ECS by at least 2 points and if clinical complete response correlates with the ECS Score at 1 year and/or 2 years from the start of treatment.

The FACT-E, version 4, will be used to measure QOL, with the focus on the Esophageal Cancer Subscale. Protocol eligible patients will be included in the QOL analysis only if they have provided baseline and at least one subsequent measurement. Patients should complete the FACT-E, version 4, pretreatment (baseline), 6-8 weeks following chemoradiation ± cetuximab at the time of clinical complete response evaluation, at one year from the start of treatment, and at 2 years from the start of treatment.

Patient reported outcomes are increasingly being incorporated into clinical trials for documentation of effects of treatment not measured by traditional endpoints such as overall and disease-free survival. This is important with interventions that may increase treatment-related side effects without positively impacting survival. Quality-adjusted survival is an endpoint that incorporates a patient's utility or preference of the health state that is combined with the time spent in that health state. The resultant is a quality-adjusted life-year (QALY). Utility can be measured by different methods including Standard Gamble, Time Trade-Off, and Health Utilities Index III. The Euroqol or EQ-5D is another instrument for measuring utilities. It is a two-part questionnaire that takes approximately 5 minutes to complete.[48] The first part consists of 5-items covering 5-dimensions including: mobility, self-care, usual activities, pain/discomfort, and anxiety/depression. Each dimension can be graded on 3 levels including: 1-no problem, 2-moderate problems, and 3-extreme problems. There are 243 potential health states. The send part is a visual analogue scale (VAS) valuing current health state, measured on a 20 cm 10 point-

interval scale. Either the index score of the VAS score can be used in the quality-adjusted survival analysis.[49] The benefit of measuring quality-adjusted survival is that the product, quality-adjusted survival, can be compared to the outcomes of other interventions across disease sites and can be used by health policy makers to rank interventions. RTOG has incorporated the EQ-5D in clinical trials RTOG 0320 (brain metastases) and RTOG 0522 (head and neck cancer) in an attempt to further determine patient utility in a number of different disease sites.

The EQ-5D has been used to evaluate interventions in patients with esophageal cancer. Williams et al found a baseline utility of .676 and .663 in patients undergoing esophageal endoscopy by a physician and nurse, respectively, in the Multi-Institution Nurse Endoscopy Trial (MINuET).[50] A utility is a patient preferences for a certain health state, with 0 being death and 1 being perfect health. There are some health states with a utility of < 1. At 1 year, patient utility increased to .725 and .703 for endoscopy by a physician and nurse, respectively. Jones et al used the EQ-5D to assess heartburn in patients with gastroesophageal reflux disease in Germany and Sweden.[51] They found a reduction in health-related QOL in patients with heartburn, with patients with more severe heartburn symptoms having reduced quality-adjusted survival. Of note, they did not find a relationship between the findings at endoscopy and the severity of symptoms as measured by the Gastrointestinal Symptom Rating Scale (GSRS) or the EQ-5D.

The EQ-5D will be used to assess quality-adjusted survival. Protocol eligible patients will be included in the quality-adjusted survival analysis only if they have provided baseline and at least one subsequent measurement. Patients should complete the EQ-5D pretreatment (baseline), 6-8 weeks following chemoradiation ± cetuximab at the time of clinical complete response evaluation, at one year from the start of treatment, and at 2 years from the start of treatment.. Quality-adjusted survival is then calculated as the weighted sum of different time in different health states added up to a total quality-adjusted survival time [U=sum of quality (qi) of health states K times the duration (si) spent in each health state].[52]

2.0 OBJECTIVES

2.1 Primary

2.1.1 To evaluate if the addition of cetuximab to paclitaxel, cisplatin, and radiation improves overall survival compared to paclitaxel, cisplatin, and radiation alone in patients with esophageal cancer who are treated without surgery.

2.2 Secondary

2.2.1 To evaluate if the addition of cetuximab to paclitaxel, cisplatin, and radiation improves local control by increasing the clinical complete response and decreasing local recurrence for patients with esophageal cancer who are treated without surgery.

2.2.2 To evaluate adverse events.

2.2.3 To evaluate endoscopic complete response rates.

2.2.4 To evaluate if the addition of cetuximab to paclitaxel, cisplatin, and radiation improves the Esophageal Cancer Subscale score of the FACT-E quality of life tool.

2.2.5 To evaluate the quality-adjusted survival of each treatment arm using EQ-5D if the primary endpoint supports the primary hypothesis.

2.2.6 To implement a well-controlled tissue handling/storage protocol to facilitate future laboratory correlative studies.

3.0 PATIENT SELECTION (5/3/12)

NOTE: PER NCI GUIDELINES, EXCEPTIONS TO ELIGIBILITY ARE NOT PERMITTED

3.1 Conditions for Patient Eligibility (2/13/09, 8/19/10)

3.1.1 Pathologically (histologic or cytologic) proven diagnosis of primary squamous cell ~~or adenocarcinoma~~ of the esophagus or gastroesophageal junction within 12 weeks prior to registration. Patients with involvement of the gastroesophageal junction with Siewert type I or II tumors (tumors arising from the distal esophagus and involving the esophagogastric junction or tumors starting at the esophagogastric junction and involving the cardia) are eligible.[53] **(accrual of adenocarcinoma patients closed 5/23/2012)**

3.1.1.1 Disease must be encompassed in a radiotherapy field.

3.1.1.2 Patients with celiac, perigastric, mediastinal or supraclavicular adenopathy are eligible.

3.1.1.3 Patients with cervical esophageal carcinoma are eligible.

3.1.2 Stage T1N1M0; T2-4, Any N, M0; Any T, Any N, M1a, based upon the following minimum diagnostic work-up:

3.1.2.1 History/physical examination within 6 weeks prior to registration

3.1.2.2 PET/PET-CT scan (strongly recommended) or chest/abdominal CT within 6 weeks prior to registration

3.1.2.3 EKG within 6 weeks of study entry

3.1.2.4 Endoscopy with biopsy or cytology by fine needle aspiration (FNA) (must be able to document histologic subtype) within 12 weeks of study entry. Patients with T3-4 proximal thoracic esophageal tumors (15-25 cm) must undergo bronchoscopy to exclude fistula. (**NOTE**: Any images from endoscopic procedures up to the time of progression must be kept in the patient's confidential study file.)

3.1.3 Zubrod performance status 0-2

3.1.4 Age ≥ 18 and < 75

3.1.5 CBC/differential obtained within 2 weeks prior to registration on study, with adequate bone marrow function defined as follows:

3.1.5.1 Absolute neutrophil count (ANC) ≥ 1,500 cells/mm^3

3.1.5.2 Platelets ≥ 100,000 cells/mm^3

3.1.5.3 Hemoglobin ≥ 8.0 g/dl (Note: The use of transfusion or other intervention to achieve Hgb ≥ 8.0 g/dl is acceptable.)

3.1.6 Additional laboratory studies obtained within 2 weeks prior to registration on study

3.1.6.1 Creatinine ≤ 1.5 mg/dl

3.1.6.2 Bilirubin ≤ 1.5 x upper limit of normal

3.1.6.3 AST or ALT ≤ 3 x upper limit of normal

3.1.6.4 Serum pregnancy test for women of childbearing potential

3.1.7 Patient's total intake (oral/enteral) must be ≥ 1500 kCal/day

3.1.8 Patient must provide study-specific informed consent prior to study entry

3.1.9 Women of childbearing potential and male participants must practice adequate contraception

3.2 Conditions for Patient Ineligibility

3.2.1 Evidence of tracheoesophageal fistula, or invasion into the trachea or major bronchi. Patients with T3-4 proximal thoracic esophageal tumors (15-25 cm) must undergo bronchoscopy to exclude fistula.

3.2.2 Prior invasive malignancy (except non-melanomatous skin cancer) unless disease free for a minimum of 2 years (For example, carcinoma in situ of the breast, oral cavity, or cervix are all permissible).

3.2.3 Prior systemic chemotherapy for esophageal cancer; note that prior chemotherapy for a different cancer is allowable. See Section 3.2.2.

3.2.4 Prior radiation therapy that would result in overlap of planned radiation therapy fields.

3.2.5 Prior therapy that specifically and directly targets the EGFR pathway.

3.2.6 Prior platinum-based and/or paclitaxel-based therapy.

3.2.7 Prior allergic reaction to the study drugs involved in this protocol.

3.2.8 Prior severe infusion reaction to a monoclonal antibody.

3.2.9 Severe, active comorbidity, defined as follows:

3.2.9.1 Unstable angina and/or congestive heart failure requiring hospitalization within the last 3 months

3.2.9.2 Transmural myocardial infarction within the last 6 months

3.2.9.3 Acute bacterial or fungal infection requiring intravenous antibiotics at the time of registration

3.2.9.4 Chronic obstructive pulmonary disease exacerbation or other respiratory illness requiring hospitalization or precluding study therapy at the time of registration

3.2.9.5 Acquired immune deficiency syndrome (AIDS) based upon current CDC definition; note, however, that HIV testing is not required for entry into this protocol. The need to exclude patients with AIDS from this protocol is necessary because the treatments involved in this protocol may be significantly immunosuppressive. Protocol-specific requirements may also exclude immunocompromised patients.

3.2.10 Pregnancy or women of childbearing potential and men who are sexually active and not willing/able to use medically acceptable forms of contraception; this exclusion is necessary because the treatment involved in this study may be significantly teratogenic.

3.2.11 Women who are nursing.

4.0 PRETREATMENT EVALUATIONS/MANAGEMENT (2/13/09, 3/31/09)

Note: This section lists baseline evaluations needed before the initiation of protocol treatment that do not affect eligibility.

4.1 Required Evaluations/Management

4.1.1 Magnesium, calcium, potassium

5.0 REGISTRATION PROCEDURES

5.1 Pre-Registration Requirements for 3D-CRT Treatment Approach (2/13/09)

Only institutions that have met the technology requirements and that have provided the baseline physics information that are described in 3D-CRT Quality Assurance Guidelines may enter patients to this study.

5.1.1 The new Facility Questionnaire [one per institution, see Advanced Technology Consortium (ITC) website at http://atc.wustl.edu] is to be sent to RTOG for review prior to entering any cases. Upon review and successful completion of "Dry-Run" QA test, the ITC will notify both the registering institution and RTOG Headquarters that the institution is eligible to enter patients onto this study. Institutions that have previously enrolled patients on 3D-CRT trials with digital data submission may enroll patients on this study without further credentialing by the ITC.

5.1.2 Each institution must contact the Washington University Image-Guided Center (ITC) at itc@castor.wustl.edu and request an SFTP account for digital data submission.

5.2 Regulatory Pre-Registration Requirements (5/3/12)

5.2.1 This study is supported by the NCI Cancer Trials Support Unit (CTSU).

Prior to the recruitment of a patient for this study, investigators must be registered members of a Cooperative Group or a CTSU CICRS site. Each investigator must have an NCI investigator number and must maintain an "active" investigator registration status through the annual submission of a complete investigator registration packet (FDA Form 1572 with original signature, current CV, Supplemental Investigator Data Form with signature, and Financial Disclosure Form with original signature) to the Pharmaceutical Management Branch, CTEP, DCTD, NCI. These forms are available on the CTSU registered member Web site or by calling the PMB at 301-496-5725 Monday through Friday between 8:30 a.m. and 4:30 p.m. Eastern time.

Each investigator or group of investigators at a clinical site must obtain IRB approval for this protocol and submit IRB approval and supporting documentation to the CTSU Regulatory Office before they can enroll patients. Study centers can check the status of their registration packets by querying the Regulatory Support System (RSS) site registration status page of the CTSU member web site by entering credentials at https://www.ctsu.org.

Requirements for RTOG 0436 site registration:

- CTSU IRB Certification
- CTSU IRB/Regulatory Approval Transmittal Sheet
- CTSU RT Facilities Inventory Form

NOTE: Per NCI policy all institutions that participate on protocols with a radiation therapy component must participate in the Radiological Physics Center (RPC) monitoring program. For non-lead group institutions an RT Facilities Inventory From must be on file with CTSU. If this form has been previously submitted to CTSU it does not need to be resubmitted unless updates have occurred at the RT facility.

In addition to the requirements noted above, U.S. sites and Canadian institutions must fax copies of the documentation below to the CTSU Regulatory Office (215-569-0206 prior to registration of the institution's first case:

- IRB/REB approved consent (English and native language versions*)

Note: Institutions must provide certification of consent translation to RTOG Headquarters.

- IRB/REB assurance number renewal information as appropriate.

5.2.2 *Pre-Registration Requirements FOR CANADIAN INSTITUTIONS*

Prior to clinical trial commencement, Canadian institutions must complete and fax to the CTSU Regulatory Office (215-569-0206) Health Canada's Therapeutic Products Directorates' Clinical Trial Site Information Form, Qualified Investigator Undertaking Form, and Research Ethics Board Attestation Form.

5.2.3 *Pre-Registration Requirements for the Initial Shipment of Cetuximab and for Canadian Sites, Shipment of Paclitaxel (9/9/09):*

5.2.3.1 **US and Canadian institutions:**

All pre-registration requirements must be met before calling to register the first case. Institutions must electronically complete (versus hand write) a Study Agent Shipment Form (SASF) available on the RTOG web site, www.rtog.org under protocol-specific materials/regulatory resources. U.S. and Canadian institutions must fax the SASF to the CTSU Regulatory Office (Fax 215-569-0206) as soon as the individual responsible for the study agent has been identified.

5.3 OPEN Registration (6/22/10)

Patient registration can occur only after pre-treatment evaluation is complete, eligibility criteria have been met, and the study site is listed as 'approved' in the CTSU RSS. Patients must have signed and dated all applicable consents and authorization forms.

All site staff (RTOG and CTSU Sites) will use OPEN to enroll patients to this study. OPEN can be accessed at https://open.ctsu.org or from the OPEN tab on the CTSU members' web site https://www.ctsu.org.

Prior to accessing OPEN site staff should verify the following:

- All eligibility criteria has been met within the protocol stated timeframes. Site staff should use the registration forms provided on the group or CTSU web site as a tool to verify eligibility.
- All patients have signed an appropriate consent form and HIPPA authorization form (if applicable).

Access requirements for OPEN:

- Site staff will need to be registered with CTEP and have a valid and active CTEP-IAM account. This is the same account (user id and password) used for the CTSU members' web site.
- To perform registrations, the site user must have been assigned the 'Registrar' role on the relevant Group or CTSU roster.
- To perform registrations on protocols for which you are a member of the RTOG, you must have an equivalent 'Registrar' role on the RTOG roster. Role assignments are handled through the Groups in which you are a member
- To perform registrations to trials accessed via the CTSU mechanism (i.e., non-Lead Group registrations) you must have the role of Registrar on the CTSU roster. Site and/or Data Administrators can manage CTSU roster roles via the new Site Roles maintenance feature under RSS on the CTSU members' web site. This will allow them to assign staff the "Registrar" role.
- **NOTE**: **If you are enrolling as a non-RTOG site**: Prior to beginning the enrollment, call the RTOG Randomization desk at 215-574-3191 or 215-574-3192 to obtain an RTOG, non-Lead Group, site-specific institution number.

The OPEN system will provide the site with a printable confirmation of registration and treatment information. Please print this confirmation for your records.

Further instructional information is provided on the CTSU members' web site OPEN tab or within the OPEN URL. For any additional questions contact the CTSU Help Desk at 1-888-823-5923 or ctsucontact@westat.com.

5.3.1 In the event that the OPEN system is not accessible, participating sites can register a patient by calling RTOG Headquarters, at (215) 574-3191, Monday through Friday, 8:30 a.m. to 5:00

p.m. ET. The registrar will ask the site to fax in the eligibility checklist and will need the registering individual's e-mail address and/or return fax number. This information is required to assure that mechanisms usually triggered by the OPEN web registration system (e.g. drug shipment, confirmation of registration, and patient-specific calendar) will occur.

6.0 RADIATION THERAPY (8/12/08. 2/13/09. 3/31/09, 9/9/09)

Note: Intensity Modulated RT (IMRT) Is Not Allowed. All doses are to be prescribed and calculated assuming a homogeneous patient.

A second heterogeneity corrected plan should also be calculated using the same monitor units (MUs) as used for the homogeneous plan (same MUs as used for treatment). Both plans shall be submitted to the ITC (see Section 12.2).

Radiation therapy must begin on a Monday or Tuesday and within 10 business days after registration. Radiation will be given once daily for 5 days; on the days that the patient receives chemotherapy, radiation may be given prior to chemotherapy.

6.1 Dose Specifications (9/9/09)

6.1.1 The prescription dose will be specified at the ICRU-50 reference point, which is defined in Section 6.3.1.4. Note: this point will usually be the isocenter (intersection of the beams). The isodose curve representing 93% of the prescription dose must encompass the entire planning target volume (PTV), which is defined below.

6.1.2 The daily prescription dose will be 1.94 Gy at the International Commission on Radiation Units and Measurement (ICRU) reference point. 1.8 Gy (which corresponds to the 93% isodose curve) is to be delivered to the periphery of the PTV.

6.1.3 The reported doses shall include the dose to the ICRU reference point. The maximum point dose, minimum point dose, and mean dose to PTV will also be reported.

6.1.4 The total dose for both arms will be 50.4 Gy (1.8 Gy/Fx/day) prescribed to the periphery (93% isodose curve) of the PTV.

6.1.5 The Dose prescription is to be based on the **uncorrected** dose distribution calculated with heterogeneity correction turned off. A second **corrected** dose distribution will be calculated with heterogeneity correction turned on and using the same monitor units as used for the uncorrected dose distribution. Both corrected and uncorrected dose distributions shall be calculated and submitted to the ITC.

6.2 Technical Factors

6.2.1 External Beam Equipment: Megavoltage equipment is required with effective photon energies ≥ 6 MV.

6.3 Treatment Planning/Target Volumes (2/13/09, 9/9/09)

Intensity Modulated RT (IMRT) Is Not Allowed. All doses are to be prescribed and calculated assuming a homogeneous patient. A second heterogeneity corrected plan should also be calculated using the same MUs as used for the homogeneous plan (same MUs as used for treatment). Both plans shall be submitted to the ITC (see Section 12.2).

6.3.1 The definition of volumes will be in accordance with the 1993 ICRU Report #50/1999 ICRU Report #62: Prescribing, Recording and Reporting Photon Beam Therapy.

6.3.1.1 Gross tumor volume (GTV) is defined as all known gross disease as defined by the planning CT and clinical information. Gross tumor includes the primary tumor (GTV-P) only.

6.3.1.2 Clinical target volume (CTV) includes the area of subclinical involvement around the GTV. For this protocol, we have chosen to define the CTV a 4 cm proximal and distal and 1 cm lateral beyond the GTV delineated by CT scan and/or endoscopy (endoscopic ultrasound is preferable). The final CTV may be larger since for cervical primaries; the supraclavicular nodes need to be included; and for distal primaries, the celiac nodes need to be included. Planning Target Volume (PTV) will provide margin around the CTV to compensate for variations in treatment set-up, and organ motion will be included in the treatment fields.

**Esophagus lesion** (cm from the incisors)	**Nodal groups to include** (subclinical) All + nodes should be included in the port
Cervical (10-15 cm)	Supraclavicular

Mid esophageal (>15-30 cm)	Paraesophageal
Distal esophageal (> 30 cm)	Celiac

6.3.1.3 Variability in treatment setup, breathing, or motion during treatment: A margin around the CTV will define the PTV. The PTV volume must include a minimum of 1 cm and a maximum of 2 cm around the CTV. Once again, the final PTV may be larger, since the supraclavicular nodes need to be included in the treatment fields for cervical primaries and the celiac nodes need to be included in the treatment fields for distal primaries.

6.3.1.4 The ICRU reference point is to be located in the central part of PTV. Typically, this point should be located on the beam axis or at the intersection of the beam axis (isocenter).

6.4 Localization, Simulation, and Immobilization

6.4.1 A volumetric treatment planning CT study will be required to define GTV and PTV. For this study, the local regional nodes (whether clinically positive or negative) will be included in the clinical target volume (CTV).Each patient will be positioned in an individualized immobilization device in the treatment position on a flat table. Contiguous CT slices, 3-5 mm thickness of the regions harboring gross tumor and grossly enlarged nodes and 8-10 mm thickness of the remaining regions, are to be obtained starting from the level of the cricoid cartilage and extending inferiorly through the liver. The GTV and PTV and normal organs will be outlined on all appropriate CT slices and displayed using beam's eye view. Normal tissues to be contoured include both lungs, kidneys, skin, heart, spinal cord, esophagus, and liver. A measurement scale for the CT image shall be included.

6.4.2 For cervical primaries (defined as tumors above the carina), the bilateral supraclavicular nodes need to be included. The preferable method is a 3-field technique (2 anterior obliques and a posterior field). In most cases, this is not possible; therefore, it is acceptable to initially treat AP/PA to approximately 39.6 Gy, then switch to obliques to exclude the spinal cord. The supraclavicular field, which is excluded from the obliques, can be supplemented with electrons to bring the total dose up to 50.4 Gy (calculated 3 cm below the skin surface). For mid-esophageal primaries (at or below the carina), the paraesophageal nodes need to be included, not the supraclavicular or celiac. For distal/gastroesophageal primaries, the field should include the celiac nodes.

6.4.3 Barium swallow during the planning CT is optional provided a diagnostic chest CT was done with contrast to delineate the outline of the esophagus.

6.4.4 Optimal immobilization is required for this protocol. Appropriate documentation must be provided of immobilization system utilized. Patients may be placed in the supine or prone position.

6.5 Critical Structures (2/13/09)

6.5.1 Normal Tissue Volume and Tolerances: The normal tissues in the table below are to be contoured in their entirety.

6.5.2 The following organs and doses by volume, in the Guidelines on Constraints table below, are guidelines that help determine the risk of developing pneumonitis based on the delivery of the 3D treatment plan. Treating physicians must document the volume of lung receiving > than 20 Gy and attempt to maintain patients in the lowest quartile (as described below) possible. Physicians/dosimetrist should make every effort not to exceed these tolerance levels. All normal tissues assume treatment at 1.8Gy/Fx (uncorrected). There will be no heterogeneity corrections used in the definitions of these doses.

Guidelines on Constraints

Incidence of Pneumonitis (%)					
Quartile	GTV (cc)	Mean Dose (GY)	% or Ipsilateral Lung Receiving > 20 Gy	% of Total Lung Receiving >20 Gy	V eff
≥ Grade 2					
1st	32	20	7	8	0
2nd	12	21	10	23	23
3rd	27	25	38	29	25
4th	27	29	42	33	45
≥ Grade 3					
1st	11	10	7	8	0
2nd	6	11	0	0	5
3rd	3	8	21	19	14
4th	20	24	25	27	26

6.5.3 It is expected that the dose to the lungs, heart, spinal cord, kidney (for gastroesophageal junction tumors) and liver will be the primary dose-limiting structures. Every effort should be made to keep the total dose to a minimum. The spinal cord, heart, liver, and kidney dose limits are defined in the Tolerance Dose table below.

Tolerance Dose (NOT TO EXCEED THESE DOSES)

Organ	Volume	**TD 5/5**	End Point
Lung	(See **Guidelines on Constraints in Section 6.5.2)**	(See **Guidelines on Constraints in Section 6.5.2)**	Clinical Pneumonitis
Spinal Cord	5 cm<br>10 cm<br>20 cm	**50 Gy**<br>**50 Gy**<br>**47 Gy**	Myelitis<br>Myelitis<br>Myelitis
Heart	1/3<br>2/3<br>3/3	**50 Gy**<br>**45 Gy**<br>**40 Gy**	Clinical Pericarditis<br>Clinical Pericarditis<br>Clinical Pericarditis
Liver	1/2<br>2/2	**35 Gy**<br>**30 Gy**	Clinical Hepatitis<br>Clinical Hepatitis
Kidney	1/3<br>2/3<br>3/3	**50 Gy**<br>**30 Gy**<br>**23 Gy**	Renal Insufficiency

6.5.4 When planning the beam arrangement to the PTV, the lungs, heart, spinal cord, and liver should be out of the field to the greatest extent possible. The dose per fraction to the lungs, heart, and spinal cord should be maintained at 2 Gy or less per fraction to the greatest extent possible. If tolerance dose to any of the normal organs is exceeded, the alternate beam arrangements should be used.

6.6 Documentation Requirements

6.6.1 First day port films or portal images of each field must be obtained and kept by the treating institution and be available for review upon request. Twice weekly (at least 48 hours apart) verification films or images of orthogonal views (anterior to posterior and lateral projection) must be reviewed by the treating physician. The required accuracy of patient positioning and the use of multi-leaf collimator apertures suggests the daily use of on-line imaging may be desirable. If on-line daily imaging is used, this must be documented.

6.7 Compliance Criteria (2/13/09)

Maximum total dose to PTV should not exceed the prescription dose > 7%. The maximum point dose to critical normal structures outside the PTV including the unspecified tissue should not exceed the prescription dose. The treating physician must carefully consider the tolerance dose/volume to each critical normal structure and unspecified tissue.

6.8 R.T. Quality Assurance Reviews

The Radiation Oncology Co-Chair, Mohan Suntharalingam, MD, will perform a remote RT Quality Assurance Review after ITC has received complete data for the first 20 cases enrolled. Dr. Suntharalingam will perform the next review after ITC has received complete data for the next 20 cases enrolled. This will continue as complete data is available for subsequent cases. The final cases will be reviewed within 3 months after this study has reached the target accrual or as soon as complete data for all cases enrolled has been received. These reviews will be ongoing and performed remotely.

6.9 Therapy Interruptions

6.9.1 Treatment Interruptions: Therapy interruptions will usually not be necessary. However, if radiation is held for any reason, all systemic therapy must also be held. Interruptions may be kept to a minimum by the use of ancillary therapy and vigorous nutritional support. Interruptions are permitted only on the basis of toxicity. Therapy may be interrupted for absolute granulocyte counts ≤ 1000; platelet count ≤ 50,000; 6 episodes of vomiting (≥ grade 3) lasting ≥ 3 days and unresponsive to antiemetics; diarrhea ≥ 7 watery stools/day (≥ grade 3) and unresponsive to antidiarrheals; or weight loss ≥ 10% (≥ grade 2) of pretreatment weight. Rarely, non-treatment−related or unexpected toxicities may require interruption of therapy at the discretion of the treating oncologist. Interruption of therapy may continue until the toxicity has regressed to ≤ grade 2 to allow resumption of therapy; however, every effort should be made to limit treatment interruptions to 1-2 weeks.

6.9.2 Dose Modifications: Every effort must be made to deliver the full 50.4 Gy to all patients. Toxicity may be encountered that is sufficiently severe to require treatment interruption. Once the toxicity has resolved, the patient's therapy should resume and full protocol dose should be delivered. The toxicity that forced any dose reduction must be documented. If interruption of therapy (up to 2 weeks) becomes necessary, radiation therapy should be completed to the prescribed doses. Total number of fractions and elapsed days should be carefully reported. If an interruption of more than 2 weeks is necessary, resumption of treatment is at the discretion of the radiation oncology chairs. The patient's treatment plan will be considered a major deviation, but follow-up will be continued.

6.9.3 If the patient develops ≥ grade 3 treatment-related toxicity, radiation therapy and chemotherapy should be withheld. Treatment can resume once grade 3 radiation-related toxicity is no longer present. If a patient develops grade 3 esophagitis in the last week of treatment (i.e., with 5 or fewer radiation treatments remaining), radiation therapy (but not chemotherapy) may continue at the discretion of the treating physician.

6.10 Radiation Therapy Adverse Events (2/13/09)

Adverse effects related to radiation therapy include nausea/vomiting, diarrhea, weight loss, fatigue, myelosuppression, skin erythema, subcutaneous fibrosis, esophagitis, esophageal stricture, esophageal fistula, carditis, myelitis, acute radiation pneumonitis, and late pulmonary fibrosis.

6.11 Radiation Therapy Adverse Event Reporting (2/13/09)

See Section 7.8.

7.0 DRUG THERAPY (2/13/09)

Institutional participation in chemotherapy studies must be in accordance with the Medical Oncology Quality Control guidelines stated in the RTOG Procedures Manual.

Protocol treatment must begin within 10 business days after registration.

7.1 Treatment Arms

The weekly dosages of paclitaxel, cisplatin and cetuximab utilized in this study are derived from previous phase I and II studies.[5,14,35] Cetuximab will be administered first, followed by paclitaxel and cisplatin

7.1.1 Arm 1: Paclitaxel, Cisplatin, and Cetuximab -With Concurrent Radiation

Agent	Dose*	Schedule
Cetuximab	400 mg/m^2	Day 1
Cetuximab	250 mg/m^2	Day 8, 15, 22, 29 & 36
Paclitaxel	50 mg/m^2	Day 1, 8, 15, 22, 29 & 36
Cisplatin	25 mg/m^2	Day 1, 8, 15, 22, 29 & 36
Radiation	50.4 Gy, at 180 cGy/fx	Day 1-5, 8-12, 15-19, 22-26, 29-33 & 36-38

*Based on actual body weight.

7.1.2 Arm 2: Paclitaxel and Cisplatin With Concurrent Radiation

Agent	Dose*	Schedule
Paclitaxel	50 mg/m^2	Day 1, 8, 15, 22, 29 & 36
Cisplatin	25 mg/m^2	Day 1, 8, 15, 22, 29 & 36
Radiation	50.4 Gy, at 180 cGy/fx	Day 1-5, 8-12, 15-19, 22-26, 29-33 & 36-38

*Based on actual body weight.

7.2 Agents (9/13/2011)

7.2.1.1 *Cetuximab*

The initial dose of cetuximab is 400 mg/m^2 intravenously administered over 120 minutes on day 1, followed by weekly infusions of 250 mg/m^2 intravenously over 60 minutes on days 8, 15, 22, 29, and 36. The infusion rate of cetuximab must never exceed 5 mL/min.

All patients should be premedicated with diphenhydramine hydrochloride 50 mg (or an equivalent antihistamine) intravenously given 30-60 minutes prior to the first dose of cetuximab. Premedication may be administered prior to subsequent doses, but at the investigator's discretion, the dose of diphenhydramine (or a similar agent) may be reduced.

Patients must be continuously observed during the infusion for signs of anaphylaxis and must be closely monitored for treatment-related adverse events, especially infusion reactions, during the infusion and the post-infusion observation hour. For the initial cetuximab infusion, vital signs should be monitored pre-infusion andthen as needed. For subsequent infusions, vital signs should be taken pre- and post-infusion, then as needed; however, it is recommended that the patient be observed for 1 hour post-infusion. Longer observation periods may be required in patients who experience infusion reactions. See Section 7.6 for management of cetuximab-related infusion reactions.

7.2.1.2 *Paclitaxel*

Paclitaxel 50 mg/m^2 will be administered as an intravenous infusion over 1 hour on days 1, 8, 15, 22, 29 and 36.

Prior to the first dosage of paclitaxel, patients will be premedicated with dexamethasone 20 mg orally the night before and 20 mg either orally or intravenously on the morning of paclitaxel administration. On the morning of the first paclitaxel administration: if dexamethasone is given intravenously, administer 30 minutes prior to paclitaxel administration; if dexamethasone is given orally, administer 1-3 hours prior to paclitaxel administration. Also prior to the first dosage of paclitaxel, patients will be premedicated with diphenhydramine, 50 mg intravenously, and ranitidine (or other H2 blocker), 50 mg intravenously. If no allergic reactions occur, then subsequent dosages of premedications with dexamethasone, diphenhydramine, and H2 blockers may be reduced at the investigator's discretion. Patients on treatment arm 1 will receive diphenhydramine, 50 mg intravenouslyduring the first cycle of cetuximab and may be administered during subsequent cycles as needed prior to all dosages of cetuximab or continue but give prior to paclitaxel.

Patients must be attended by medical personnel for the first 15 minutes of infusion and then have blood pressure checked as needed. Medications for acute management of anaphylaxis should be readily available in the location where the patient is being treated.

7.2.1.3 *Cisplatin*

Cisplatin 25 mg/m^2 will be administered as an intravenous infusion over 30-60 minutes on days 1, 8, 15, 22, 29 and 36.

Cisplatin will be administered after paclitaxel. Patients will receive appropriate antiemetics and supplemental hydration as per their institutional protocol

7.3 Paclitaxel Agent Information (5/3/12)

Refer to package insert for additional information.

7.3.1 *Formulation*: Paclitaxel is a poorly soluble plant product from the western yew, Taxus brevifolia. Improved solubility requires a mixed solvent system with further dilutions of either 0.9% sodium chloride or 5% dextrose in water. Vials will be labeled with shelf-life. All solutions of paclitaxel exhibit a slight haziness directly proportional to the concentration of drug and the time elapsed after preparation, although when prepared as described below, solutions of paclitaxel (0.3-1.2 mg/ml) are physically and chemically stable for 27 hours at ambient temperature (27^0 C).

7.3.2 *Preparation*: A sterile solution concentrate, 6 mg/ml in 5 ml vials (30 mg/vial) in polyoxyethylated castor oil (Cremophor EL) 50% and dehydrated alcohol, USP, 50%. The contents of the vial must be diluted just prior to clinical use. Paclitaxel will be diluted to a final concentration of 0.3 to 1.2 mg/ml in D_5W, USP, in glass or polyolefin containers due to leaching of diethylhexphthalate (DEHP) plasticizer from polyvinyl chloride (PVC) bags and intravenous tubing by the Cremophor vehicle in which paclitaxel is solubilized. Each bag/bottle should be prepared immediately before administration. NOTE: Formation of a small number of fibers in solution (NOTE: acceptable limits established by the USP Particular Matter Test for LVPs) have been observed after preparation of paclitaxel. Therefore, in-line filtration is necessary for administration of paclitaxel solutions. In-line filtration should be accomplished by incorporating a hydrophilic, microporous filter of pore size not greater than 0.22 microns (e.g.: Millex-GV Millipore Products) into the i.v. fluid pathway distal to the infusion pump. Although particulate formation does not indicate loss of drug potency, solutions exhibiting excessive particulate matter formation should not be used.

7.3.3 *Administration*: Paclitaxel, at the appropriate dose and dilution, will be given as a 1-hour infusion. The paclitaxel is mixed in D_5W or NS with 0.22 m in-line filter. Paclitaxel will be administered via an infusion control device (pump) using non-PVC tubing and connectors, such as the i.v. administration sets (polyethylene or polyolefin) that are used to infuse parenteral nitroglycerin. Nothing else is to be infused through the line through which paclitaxel is administered.

Caution is warranted when paclitaxel is concomitantly administered with known substrate or inhibitors of CYP2C8 and CYP3A4.

7.3.4 *Storage*: Paclitaxel vials should be stored between 20°-25°C (68°-77°F).

7.3.5 *Adverse Effects*:

- Hematologic: Myelosuppression
- Gastrointestinal: Nausea, diarrhea, vomiting, abdominal pain
- Heart: Arrhythmias, heart block, hypertension
- Neurological: Sensory and peripheral neuropathy
- Allergy: Severe anaphylactic reactions
- Other: Alopecia, fatigue, arthralgia, myopathy, myalgia, infiltration (erythema, induration, tenderness, rarely ulceration), hypotension, irritation to the injection site, mucositis

7.3.6 *Supply* (9/9/09)

Commercially available in the U.S. and will be provided free of charge to Canadian sites.

7.3.6.1 **Canadian Sites** (9/9/09)

Paclitaxel will be distributed by a vendor, Biologics, Inc., under contract to RTOG. Biologics will ship a patient-specific supply of paclitaxel with enough quantity to complete protocol treatment for a 200-pound individual (27 vials) once the site has registered the patient. Since doses are dependent on the patient's BSA, sites can obtain additional per-patient supply for individuals over 200 pounds by contacting Biologics.

All pre-registration requirements must be met before calling to register the first case. Institutions must electronically complete (versus hand write) a Study Agent Shipment Form (SASF) available on the RTOG web site, www.rtog.org under protocol-specific materials/regulatory resources). Canadian institutions must fax the SASF to the CTSU Regulatory Office (Fax 215-569-0206) as soon as the individual responsible for the study agent has been identified.

Biologics will ship the order “same day” for all orders received before 4 p.m. EST, Monday through Thursday via FedEx International. Orders received after 4 p.m. EST, Monday through Thursday and any time on Friday will be processed and shipped the next business morning. Drug deliveries are restricted during weekends and holidays. Biologics observes the following holidays: New Years Eve, New Years Day, Memorial Day, July 4th, Labor Day, Thanksgiving Day and the Friday following Thanksgiving Day, Christmas Eve, and Christmas Day. Sites should plan ahead to accommodate patients being treated during restricted times.

Upon notification of a new patient enrollment, Biologics will place an outbound call to the site contact to confirm that the site’s shipment is being processed. Biologics’ distribution team will monitor packages throughout the duration of transit via the FedEx web site and FedEx One Call Solution (live support). Real-time monitoring enables Biologics to mitigate potential delivery delays.

Additional questions about supply and delivery should be directed to:

Karl Buer, Clinical Trial Project Manager
Biologics, Inc.
120 Westin Oaks Court
Cary, NC 27513
Clinical Trial Services Phone: (800) 693-4906
Direct Phone: (919) 459-4991
Fax (919) 256-0794
kbuer@biologicstoday.com

7.4 Cisplatin Agent Information (Cis-Diamminedichloroplatinum, DDP) (2/13/09)
Refer to package insert for additional information.

7.4.1 *Formulation*: Each vial contains 10 mg of DDP, 19 mg of sodium chloride, 100 mg of mannitol, and hydrochloric acid for pH adjustment. One vial is reconstituted with 10 ml of sterile water. The pH range will be 3.5 to 4.5. Cisplatin injection also is available from the manufacturer in aqueous solution, each ml containing 1 mg cisplatin and 9 mg NaCl and HCL or NaOH to adjust pH.

7.4.2 *Mechanism of Action:* The mechanism of action of DDP has not been clearly elucidated. However, preliminary studies have indicated that the most likely mechanism of antitumor action of this drug resides in its ability to inhibit DNA synthesis and to a lesser degree, RNA and protein synthesis. It has also been shown that DDP binds to DNA and produces inter-strand cross-links. Also DDP is not phase-sensitive and its cytotoxicity is similar in all phases of the cell cycle.

7.4.3 *Preparation*: Reconstituted solution of cisplatin is stable for 20 hours when stored at 27°C and should be protected from light if not used within 6 hours.

7.4.4 *Administration*: Intravenous.

7.4.5 *Adverse Events*

- Hematologic: Myelosuppression, often with delayed erythrosuppression; rarely, acute leukemia
- Gastrointestinal: Nausea, vomiting, anorexia, loss of taste;
- Dermatologic: Alopecia;
- Renal: Elevation of BUN, creatinine and impairment of endogenous creatinine clearance, as well as renal tubular damage which appears to be transient); hyperuricemia; much more severe and prolonged adverse events have been observed in patients with abnormal or obstructed urinary excretory tracts;
- Hepatic: Hypomagnesemia, hypokalemia, hypocalcemia,

- Neurologic: Restlessness; involuntary movements; loss of coordination; seizures; peripheral neuropathy;
- Allergic: Flushing, bronchoconstriction, tachycardia, hypotension;
- Other: Ototoxicity (with hearing loss which initially is in the high-frequency range, as well as tinnitus); muscle cramps; weakness

7.4.6 *Storage:* Intact vials of the dry powder and the aqueous injection should be stored at room temperature (15-25°C) and protected from light; the vials and injection should not be refrigerated.

7.4.7 *Supply*: Commercially available.

7.4.7.1 **Non-Canadian international institutions:**
Please refer to your LOI Approval Notification. Your institution will be responsible for acquiring any drug noted in the protocol as commercially available and not provided for the study.

7.5 Cetuximab (C225) Agent Information (IND #101157) (8/12/08, 2/13/09)

Refer to package insert and investigator brochure for additional information. The investigator brochure is available on the RTOG website at http://www.rtog.org/LinkClick.aspx?fileticket=lKAXjtejh2Q%3d&tabid=290.

7.5.1 *Formulation*
Cetuximab is an anti-EGFR receptor humanized chimeric monoclonal antibody. Cetuximab is expressed in SP2/0 myeloma cell line, grown in large scale cell culture bioreactors, and purified to a high level purity using several purification steps including protein A chromatography, ion exchange chromatography, low pH treatment, and nanofiltration. Cetuximab is not known to be a vesicant.

7.5.2 *Safety Precautions*
Appropriate mask, protective clothing, eye protection, gloves and Class II vertical-laminar-airflow safety cabinets are recommended during preparation and handling.

Patients should wear sunscreen and hats to limit sun exposure while receiving cetuximab.

7.5.3 *Preparation and Administration*
Cetuximab must not be administered as an *IV* push or bolus.
Cetuximab must be administered with the use of a low protein binding 0.22-micrometer in-line filter.

Cetuximab is supplied as a 50-mL, single-use vial containing 100 mg of cetuximab at a concentration of 2 mg/mL in phosphate buffered saline. The solution should be clear and colorless and may contain a small amount of easily visible white amorphous cetuximab particulates. **DO NOT SHAKE OR DILUTE.**

Cetuximab can be administered via infusion pump or syringe pump.

Infusion Pump:
1. Draw up the volume of a vial using a sterile syringe attached to an appropriate needle (a vented spike or other appropriate transfer device may be used).
2. Fill cetuximab into a sterile evacuated container or bag such as glass containers, polyolefin bags (e.g., Baxter Intravia), ethylene vinyl acetate bags (e.g., Baxter Clintec), DEHP plasticized PVC bags (e.g., Abbott Lifecare), or PVC bags.
3. Repeat procedure until the calculated volume has been put in to the container. Use a new needle for each vial.
4. Administration must be through a low protein binding 0.22-micrometer in-line filter (placed as proximal to the patient as practical).
5. Affix the infusion line and prime it with cetuximab before starting the infusion.
6. Maximum infusion rate should not exceed 5 mL/min.
7. Use 0.9% saline solution to flush line at the end of infusion.

Syringe Pump:
1. Draw up the volume of a vial using a sterile syringe attached to an appropriate needle (a vented spike may be used).
2. Place the syringe into the syringe driver of a syringe pump and set the rate.
3. Administration must be through a low protein binding 0.22-micrometer in-line filter rated for syringe pump use (placed as proximal to the patient as practical).

4. Connect up the infusion line and start the infusion after priming the line with cetuximab.
5. Repeat procedure until the calculated volume has been infused.
6. Use a new needle and filter for each vial.
7. Maximum infusion rate should not exceed 5 mL/min.
8. Use 0.9% saline solution to flush line at the end of infusion.

Cetuximab should be piggybacked to the patient's infusion line.

Following the cetuximab infusion, a 1-hour observation period is recommended.

7.5.4 *Adverse Events* (6/10/10)

Comprehensive Adverse Events and Potential Risks list (CAEPR)
for
Cetuximab (NSC 714692)

The Comprehensive Adverse Event and Potential Risks list (CAEPR) provides a single list of reported and/or potential adverse events (AE) associated with an agent using a uniform presentation of events by body system. In addition to the comprehensive list, a subset, the Agent Specific Adverse Event List (ASAEL), appears in a separate column and is identified with ***bold*** and ***italicized*** text. This subset of AEs (ASAEL) contains events that are considered 'expected' for expedited reporting purposes only. Refer to the 'CTEP, NCI Guidelines: Adverse Event Reporting Requirements' http://ctep.info.nih.gov/protocolDevelopment/default.htm#adverse_events_adeers for further clarification. *Frequency is provided based on 2282 patients.* Below is the CAEPR for Cetuximab.

Version 2.1, March 31, 2010[1]

**Adverse Events with Possible Relationship to Cetuximab (CTCAE 4.0 Term) [n= 2282]**			**EXPECTED AEs FOR ADEERS REPORTING Agent Specific Adverse Event List (ASAEL)**
**Likely (>20%)**	**Less Likely (<=20%)**	**Rare but Serious (<3%)**	***Expected***
BLOOD AND LYMPHATIC SYSTEM DISORDERS			
	Anemia		
EAR AND LABYRINTH DISORDERS			
	External ear inflammation		
	Tinnitus		
EYE DISORDERS			
	Conjunctivitis		***Conjunctivitis***
	Dry eye		***Dry eye***
	Uveitis		***Uveitis***
	Watering eyes		***Watering eyes***
GASTROINTESTINAL DISORDERS			
	Abdominal pain		***Abdominal pain***
	Cheilitis		***Cheilitis***
	Constipation		***Constipation***
Diarrhea			***Diarrhea***
	Dry mouth		***Dry mouth***
	Dyspepsia		***Dyspepsia***
	Mucositis oral		***Mucositis oral***
Nausea			***Nausea***
	Vomiting		***Vomiting***
GENERAL DISORDERS AND ADMINISTRATION SITE CONDITIONS			
	Chills		***Chills***
	Edema limbs		
Fatigue			***Fatigue***

Fever			***Fever***
	Flu like symptoms		***Flu like symptoms***
	Infusion related reaction		***Infusion related reaction***
	Non-cardiac chest pain		
IMMUNE SYSTEM DISORDERS			
	Allergic reaction		***Allergic reaction***
		Anaphylaxis	
INFECTIONS AND INFESTATIONS			
	Infection[2]		
		Infections and infestations – Other (aseptic meningitis)	
INVESTIGATIONS			
	Neutrophil count decreased		
	Weight loss		***Weight loss***
	White blood cell decreased		***White blood cell decreased***
METABOLISM AND NUTRITION DISORDERS			
	Anorexia		***Anorexia***
	Dehydration		***Dehydration***
	Hypocalcemia		
	Hypomagnesemia		***Hypomagnesemia***
MUSCULOSKELETAL AND CONNECTIVE TISSUE DISORDERS			
	Arthralgia		***Arthralgia***
	Back pain		***Back pain***
	Myalgia		***Myalgia***
NERVOUS SYSTEM DISORDERS			
Headache			***Headache***
	Syncope		
RESPIRATORY, THORACIC AND MEDIASTINAL DISORDERS			
	Allergic rhinitis		***Allergic rhinitis***
	Bronchospasm		
	Cough		***Cough***
	Dyspnea		***Dyspnea***
	Hoarseness		***Hoarseness***
		Pneumonitis	
		Respiratory, thoracic, and mediastinal disorders - Other (non-cardiogenic pulmonary edema)	
SKIN AND SUBCUTANEOUS TISSUE DISORDERS			
	Alopecia		***Alopecia***
Dry skin			***Dry skin***
	Nail loss		***Nail loss***
		Palmar-plantar erythrodysesthesia syndrome	
	Photosensitivity		***Photosensitivity***
	Pruritus		***Pruritus***
	Purpura		
Rash acneiform			***Rash acneiform***
Rash maculo-papular			***Rash maculo-papular***
	Skin ulceration		
	Urticaria		***Urticaria***
VASCULAR DISORDERS			
	Hypotension		***Hypotension***
	Thromboembolic event		***Thromboembolic event***

[1]This table will be updated as the toxicity profile of the agent is revised. Updates will be distributed to all Principal Investigators at the time of revision. The current version can be obtained by contacting

PIO@CTEP.NCI.NIH.GOV. Your name, the name of the investigator, the protocol and the agent should be included in the e-mail.

[2]Infection could include all 75 sites of infections under the INFECTIONS AND INFESTATIONS SOC.

Also reported on cetuximab trials but with the relationship to cetuximab still undetermined:

BLOOD AND LYMPHATIC SYSTEM DISORDERS - Disseminated intravascular coagulation; Hemolysis
CARDIAC DISORDERS - Atrial fibrillation; Atrial flutter; Chest pain - cardiac; Left ventricular systolic dysfunction; Myocardial infarction; Paroxysmal atrial tachycardia; Pericardial effusion; Sinus bradycardia; Sinus tachycardia; Supraventricular tachycardia
EAR AND LABYRINTH DISORDERS - Hearing impaired
EYE DISORDERS - Blurred vision; Extraocular muscle paresis; Eyelid function disorder; Keratitis; Photophobia; Vitreous hemorrhage
GASTROINTESTINAL DISORDERS - Colitis; Dysphagia; Esophagitis; Gastritis; Gastrointestinal disorders - Other (diverticulitis); Gastrointestinal hemorrhage (including Colonic or Gastric hemorrhage or hemorrhage in other sites under the GASTROINTESTINAL DISORDERS SOC); Gastrointestinal perforation (Colonic perforation, Duodenal perforation, or perforation in other sites under the GASTROINTESTINAL DISORDERS SOC); Gastrointestinal ulcer (ulcer includes Duodenal ulcer, Rectal ulcer, or ulcer in other sites under the GASTROINTESTINAL DISORDERS SOC); Ileus; Pancreatitis; Rectal fistula
GENERAL DISORDERS AND ADMINISTRATION SITE CONDITIONS - Edema face; Sudden death NOS
HEPATOBILIARY DISORDERS - Cholecystitis; Hepatic failure
INJURY, POISONING AND PROCEDURAL COMPLICATIONS - Bruising; Wound dehiscence
INVESTIGATIONS - Alanine aminotransferase increased; Alkaline phosphatase increased; Aspartate aminotransferase increased; Blood bilirubin increased; Creatinine increased; Platelet count decreased; Serum amylase increased
METABOLISM AND NUTRITION DISORDERS - Hyperkalemia; Hyperuricemia; Hypokalemia; Hyponatremia; Hypophosphatemia
MUSCULOSKELETAL AND CONNECTIVE TISSUE DISORDERS - Generalized muscle weakness; Musculoskeletal and connective tissue disorder - Other (myasthenia); Musculoskeletal and connective tissue disorder - Other (Sudeck's Atrophy)
NERVOUS SYSTEM DISORDERS - Ataxia; Dizziness; Dysgeusia; Extrapyramidal disorder; Intracranial hemorrhage; Nervous system disorders - Other (cholinergic syndrome); Neuralgia; Peripheral motor neuropathy; Peripheral sensory neuropathy; Seizure; Tremor
PSYCHIATRIC DISORDERS - Agitation; Depression
RENAL AND URINARY DISORDERS - Hematuria; Renal and urinary disorders - Other (acute renal failure)
REPRODUCTIVE SYSTEM AND BREAST DISORDERS - Reproductive system and breast disorders - Other (balanitis); Vaginal inflammation
RESPIRATORY, THORACIC AND MEDIASTINAL DISORDERS - Adult respiratory distress syndrome; Atelectasis; Bronchopulmonary hemorrhage; Epistaxis; Pleural effusion; Respiratory, thoracic and mediastinal disorders - Other (bronchiolitis obliterans-organized pneumonia [BOOP])
SKIN AND SUBCUTANEOUS TISSUE DISORDERS - Hirsutism; Skin hypopigmentation; Skin and subcutaneous tissue disorders - Other (skin fissures)
VASCULAR DISORDERS - Flushing; Hypertension; Lymphedema; Vasculitis

Note: Cetuximab in combination with other agents could cause an exacerbation of any adverse event currently known to be caused by the other agent, or the combination may result in events never previously associated with either agent.

7.5.5 *Storage Requirements/Stability*

Store vials under refrigeration at 2° C to 8° C (36° F to 46° F). **DO NOT FREEZE.** Increased particulate formation may occur at temperatures at or below 0°C. This product contains no preservatives. Preparations of cetuximab in infusion containers are chemically and physically stable for up to 12 hours at 2° C to 8° C (36° F to 46° F) and up to 8 hours at controlled room temperature (20° C to 25° C; 68° F to 77° F). Discard any remaining solution in the infusion container after 8 hours at controlled room temperature or after 12 hours at 2° to 8° C. Discard any unused portion of the vial.

7.5.6 *Supply*

This study will be conducted under an IND to be held by RTOG and will require FDA submission and approval as part of the IND.

Bristol-Myers Squibb (BMS) will supply cetuximab free of charge to patients on study. The drug will be distributed by a vendor, Biologics, Inc., under contract to RTOG. The product is a sterile, clear, colorless liquid of pH 7.0 to 7.4, which may contain a small amount of easily visible, white, amorphous cetuximab particulates. Each single-use 50-mL vial contains 100 mg of cetuximab at a concentration of 2 mg/mL and is formulated in a preservative-free solution containing 8.48 mg/mL sodium chloride, 1.88 mg/mL sodium phosphate dibasic heptahydrate, 0.42mg/mL sodium phosphate monobasic monohydrate, and Water for injection, USP.

7.5.6.1 **Non-Canadian international institutions:**

Please refer to your LOI Approval Notification. Your institution will be responsible for acquiring any drug noted in the protocol as commercially available and not provided for the study. Before drug can be provided your institution must comply with all pre-registration requirements and certifications and provide all necessary documentation listed in your LOI Approval Notification document.

7.5.7 *Drug Ordering and Accountability* (9/9/09)

Drug accountability records must be maintained at all sites according to good clinical practices and NCI guidelines.

The Study Agent Shipment Form for US and Canadian sites must be submitted to the CTSU Regulatory Office (Fax 215-569-0206) as soon as the individual responsible for the study agent has been identified. **Note: International sites must receive written approval of submitted LOI Forms from RTOG Headquarters prior to submitting documents to local ethics committee for approval. See** http://www.rtog.org/LinkClick.aspx?fileticket=0tMdct9KHSs%3d&tabid=117.**Approved international institutions must submit the Study Agent Shipment Form and documentation of IRB approval to RTOG Headquarters (Fax 215-574-0300).**

This must be done prior to registration of the institution's first case. Allow adequate processing time (7-10 days) before calling to randomize your first patient. Required regulatory documents (see Section 5.2) must be received before drug can be shipped.

Biologics will ship a patient-specific supply of cetuximab with enough quantity to complete protocol treatment for a 200-pound individual (40 vials) once the site has registered the patient. Since doses are dependent on the patient's BSA, sites can obtain additional per-patient supply for individuals over 200 pound by contacting Biologics.

All product will be shipped via Federal Express Priority Overnight delivery in a temperature-controlled container. Biologics will ship drug Monday through Thursday for delivery to the site Tuesday through Friday. Biologics will ship drug same day for all orders received before 4 PM EST Monday through Thursday. Orders received after 4 PM EST Monday through Thursday and any time on Friday will be processed and shipped the next business day. There will be no weekend or holiday delivery of drugs.

It is possible that sites will have more than one cetuximab clinical study ongoing at the same time. <u>It is imperative that only product designated for RTOG 0436 be utilized for this study.</u> RTOG 0436 product must be segregated from other investigational or marketed product.

Inside each shipping container will be a disposable electronic unit (TagAlert™) to ensure the product has remained at the appropriate temperature during shipping. This unit will be attached to an information card. The LCD display will show OK (indicating no alarm has been triggered) or a black bar and the number(s) 1-4 (indicating an alarm/alarms have been triggered). Should an alarm be triggered, follow the instructions on the attached information card. Display results should be recorded on the packing list. For questions regarding drug requisitioning, contact Biologics.

Additional questions about supply and delivery should be directed to:

Karl Buer, Clinical Trial Project Manager
Biologics, Inc.
120 Westin Oaks Court
Cary, NC 27513
Clinical Trial Services Phone: (800) 693-4906

Direct Phone: (919) 459-4991
Fax (919) 256-0794
clinicaltrials@biologicstoday.com
or
kbuer@biologicstoday.com

7.5.8 *Handling and Dispensing of Investigational Product*
Investigational product should be stored in a secure area according to local regulations. It is the responsibility of the Investigator to ensure that investigational product is only dispensed to study patients. The investigational product must be dispensed only from official study sites by authorized personnel according to local regulations.

7.5.9 *Drug Destruction and Return*
Opened vials must be disposed of at the site as chemotherapy or biohazardous waste, provided documented procedures for destruction are in place. At the completion of the study, all unused drugs will be destroyed at the site according to the institution's policy for drug destruction. It is the responsibility of the Investigator to ensure that a current record of investigational product disposition is maintained at each study site where investigational product is inventoried and disposed, including dates and quantities. If approved procedures for destruction are not in place and/or for questions regarding cetuximab destruction, please contact Biologics at 800-850-4306.

7.6 Dose Modifications (9/13/2011)
NOTE: No dose reduction should be made for weight loss given the brief duration of therapy
All dose modifications will reflect the most severe toxicity that is observed, including hematologic and non-hematologic toxicity, skin toxicity, and creatinine toxicity.

7.6.1 Hematologic Adverse Events
The dose of paclitaxel, cisplatin, cetuximab, and radiation will be modified according to blood counts on the day of treatment as shown in the table below. Dose reductions of paclitaxel and cisplatin are permanent.

Treatment Day Blood Counts			Dosage
ANC		Platelet Count	
≥1,000 mcL	AND	> 75,000 mcL	Full dosage paclitaxel, cisplatin, and cetuximab.
500-999 mcL	OR	50,000-75,000 mcL	Full dose cetuximab. Hold cisplatin and paclitaxel. Recheck CBC weekly. When ANC > 1,000 and Plt > 75,000, resume paclitaxel at 1 dose level reduction and cisplatin at 1 dose level reduction.
< 500 mcL	OR	< 50,000 mcL	Hold XRT, cisplatin, paclitaxel, and cetuximab; Recheck CBC weekly. When ANC > 500 and Plt > 50,000 resume XRT, full-dose cetuximab. When ANC >1,000 and Plt > 75,000 resume paclitaxel and cisplatin and reduce both by 2 dose levels.

Patients who experience 4 episodes of ANC < 500 mcl or platelets < 50,000 mcl may complete radiation and cetuximab on study but will not receive additional cisplatin and paclitaxel.

Dose Reductions for Change in Serum Creatinine

**Creatinine**	**Cisplatin Dose**
≤ Upper limit of normal	25 mg/m^2
> Upper limit of normal but ≤ 2.0 mg/dl	15 mg/m^2
> 2.0 mg/dl	Discontinue cisplatin. If serum creatinine falls below 2.0 mg/dl, cisplatin may be resumed at 15 mg/m^2.

Dose levels for paclitaxel are as follows:

Weekly Paclitaxel Dose	50 mg/m^2

Weekly Paclitaxel Dose	50 mg/m^2
Dose Level -1	40 mg/m^2
Dose Level -2	30 mg/m^2

There will be no dose level reductions below a weekly dose of 30 mg/m^2.

Dose levels for cisplatin are as follows:

Weekly Cisplatin Dose	25 mg/m^2
Dose Level -1	20 mg/m^2
Dose Level -2	15 mg/m^2

There will be no dose level reductions below a weekly dose of 15 mg/m^2.

7.6.2 Non-Hematologic Adverse Events

Non-hematologic adverse events that will require dose reductions of paclitaxel or cisplatin include diarrhea, mucositis, esophagitis, and nausea/vomiting/dehydration despite adequate with antiemetic therapy (including substance p antagonists and 5-HT3 antagonists). Cetuximab will only be dose reduced for skin toxicity and infusion reactions. Refer to the table below for dose modifications.

Toxicity	Grade	Agent	Modification
1st Episode<br>Grade 3 or 4 (for the adverse events described in 7.6.2)	≥ grade 3	Cisplatin<br>Paclitaxel<br>Cetuximab	Hold until ≤ grade 2; resume, dose, reducing cisplatin by 1 dose level and paclitaxel by 1 dose level; no dose reduction for cetuximab
2nd Episode	≥ grade 3	Cisplatin<br>Paclitaxel<br>Cetuximab	Hold until ≤ grade 2; resume dose, reducing cisplatin by 1 dose level and paclitaxel by 1 dose level; no dose reduction for cetuximab
3rd Episode	≥ grade 3	Cisplatin<br>Paclitaxel<br>Cetuximab	Discontinue all cisplatin, paclitaxel, and cetuximab
Infusion Reaction	≥ grade 2	Cetuximab	Permanently reduce infusion rate by 50%
Infusion Reaction	≥ grade 3	Cetuximab	Permanently discontinue
Pulmonary Symptoms (not related to cancer)	≥ grade 2	Cetuximab	Hold until ≤ grade 1. If interstitial lung disease is confirmed cetuximab should be permanently discontinued.
Hypersensitivity reaction (despite appropriate premedication)	≥ grade 4	Paclitaxel	Discontinue. Cetuximab, cisplatin, and radiation may be continued.
1st Episode<br>Any grade 3 or 4 dermatologic adverse event**	≥ grade 3	Cetuximab	Delay infusion 1-2 wks until improves to ≤ grade 2. Resume at full dose (250 mg/m^2).<br><br>If no improvement to ≤ grade 2 within 2 weeks, discontinue.
2nd Episode	≥ grade 3	Cetuximab	Delay infusion 1-2 weeks until improves to ≤ grade 2. Resume at Dose Level -1*.<br><br>If no improvement to ≤ grade 2 within 2 weeks, discontinue.
3rd Episode	≥ grade 3	Cetuximab	Delay infusion 1-2 weeks until improves to ≤ grade 2. Resume at Dose Level -2*.<br><br>If no improvement to ≤ grade 2 within 2 weeks, discontinue.
4th Episode	≥ grade 3	Cetuximab	Discontinue

*Dose levels for cetuximab are as follows:

Weekly Cetuximab Dose	250 mg/m^2
Dose Level -1	200 mg/m^2
Dose Level -2	150 mg/m^2

There will be no dose level reductions below a weekly dose of 150 mg/m^2.

** A rash that occurs only within the radiation field should be considered as radiation dermatitis and no cetuximab dose reduction is required.

7.6.2.1 *Additional Cetuximab Toxicity Management Interventions*

7.6.2.1.1 Infusion reactions

Severe infusion reactions require the immediate interruption of cetuximab therapy and permanent discontinuation from further treatment. Appropriate medical therapy, including epinephrine, corticosteroids, intravenous antihistamines, bronchodilators, and oxygen should be available for use in the treatment of such reactions. Patients should be carefully observed until the complete resolution of all signs and symptoms.

In clinical trials, mild to moderate infusion reactions were managed by slowing the infusion rate of cetuximab and by continued use of antihistamine pre-medications (e.g., diphenhydramine) in subsequent doses. If the patient experiences a mild or moderate (grade 1 or 2) infusion reaction, the infusion rate should be permanently reduced by 50%. For grade 1 or 2 reactions manifesting only as delayed drug fever, maintain the cetuximab dose and infusion rate. Consideration should be given to administration of acetaminophen or a non-steroidal anti-inflammatory drug (NSAID) prior to the subsequent cetuximab infusion if not contraindicated in subjects. Dose and schedule of these agents is entirely at the investigator's discretion. Cetuximab should be immediately and permanently discontinued in patients who experience severe (grade 3 or 4) infusion reactions.

Caution must be exercised with every cetuximab infusion, as there have been patients who experienced their first severe infusion reaction during later infusions. Severe infusion reactions require the immediate interruption of cetuximab therapy and permanent discontinuation from further treatment. Appropriate medical therapy, including epinephrine, corticosteroids, intravenous antihistamines, bronchodilators, and oxygen should be available for use in the treatment of such reactions. Patients should be carefully observed until the complete resolution of all signs and symptoms.

7.6.2.1.2 Dermatologic reactions

Patients developing dermatologic toxicities while receiving cetuximab should be monitored for the development of inflammatory or infectious sequelae, and appropriate treatment of these symptoms should be initiated. Dose modifications of any future cetuximab infusions should be instituted in case of severe acneform rash. Treatment with topical and/or oral antibiotics should be considered; topical corticosteroids are not recommended.

7.6.2.1.3 Hypomagnesemia

Hypomagnesemia has been reported with cetuximab when administered as a single agent and in combination with multiple different chemotherapeutic regimens. The incidence of hypomagnesemia (both overall and severe [NCI CTCAE grades 3 and 4]) is increased in patients receiving chemotherapy and cetuximab compared with those receiving chemotherapy alone based on controlled clinical trials. Patients receiving cetuximab therapy should be monitored for hypomagnesemia. Magnesium repletion may be necessary based on clinical judgment.

7.7 Modality Review

The Medical Oncology Co-Chair, David Ilson, MD, will perform a Chemotherapy Assurance Review of all patients who receive or are to receive chemotherapy in this trial. The goal of the review is to evaluate protocol compliance. The review process is contingent on timely submission of chemotherapy treatment data as specified in Section 12.1. The scoring mechanism is: **Per Protocol/Acceptable Variation, Not Per Protocol, and Not Evaluable.** A report is sent to each institution once per year to notify the institution about compliance for each case reviewed in that year.

Dr. Ilson will perform a Quality Assurance Review after complete data for the first 20 cases enrolled has been received at RTOG Headquarters. Dr. Ilson will perform the next review after complete data for the next 20 cases enrolled has been received at RTOG Headquarters. This will continue as complete data is available for subsequent cases. The final cases will be reviewed within 3 months after this study has reached the target accrual or as soon as complete data for all cases enrolled has been received at RTOG Headquarters, whichever occurs first.

7.8 Adverse Events (9/13/2011)

Beginning January 1, 2011 this study will utilize the Common Terminology Criteria for Adverse Events (CTCAE) version 4.0, for AdEERS reporting of all adverse events. A copy of the CTCAE v4.0 can be downloaded from the CTEP home page http://ctep.cancer.gov/protocolDevelopment/electronic_applications/ctc.htm.

All appropriate treatment areas should have access to a copy of the CTCAE v4.0. **All AE reporting on the case report forms will continue to use CTCAE version 3.0**

All adverse events (AEs) as defined in the tables below will be reported via the AdEERS (Adverse Event Expedited Reporting System) application accessed via the CTEP web site (https://webapps.ctep.nci.nih.gov/openapps/plsql/gadeers_main$.startup).

Serious adverse events (SAEs) as defined in the tables below will be reported via AdEERS. Sites also can access the RTOG web site (http://www.rtog.org/ResearchAssociates/AdverseEventReporting.aspx) for this information.

In order to ensure consistent data capture, serious adverse events reported on AdEERs reports also must be reported on an RTOG case report form (CRF). In addition, sites must submit CRFs in a timely manner after AdEERs submissions.

7.8.1 Adverse Events (AEs)

Definition of an AE: Any unfavorable and unintended sign (including an abnormal laboratory finding), symptom, or disease temporally associated with the use of a medical treatment or procedure regardless of whether it is considered related to the medical treatment or procedure (attribution of unrelated, unlikely, possible, probable, or definite). [CTEP, NCI Guidelines: Adverse Event Reporting Requirements. January 2005; http://ctep.cancer.gov/reporting/adeers.html]

The following guidelines for reporting adverse events (AEs) apply to **all** NCI/RTOG research protocols. AEs, as defined above, experienced by patients accrued to this protocol should be reported on the AE section of the appropriate case report form (see Section 12.1). **Note: AEs indicated in the AdEERS Expedited Reporting Requirements in text and/or table in Section 7.9 also must be reported via AdEERS.**

NOTE: If the event is a Serious Adverse Event (SAE) [see next section], further reporting may be required. Reporting AEs only fulfills Data Management reporting requirements.

7.8.2 Serious Adverse Events (SAEs) — All SAEs that fit any one of the criteria in the SAE definition below must be reported via AdEERS. Contact the AdEERS Help Desk if assistance is required.

Certain SAEs as outlined below will require the use of the 24 Hour AdEERS Notification:

- **Phase II & III Studies: All unexpected potentially related SAEs**
- **Phase I Studies: All unexpected hospitalizations and all grade 4 and 5 SAEs regardless of relationship**

Definition of an SAE: Any adverse experience occurring during any part of protocol treatment and 30 days after that results in any of the following outcomes:

- Death;
- A life-threatening adverse drug experience;
- Inpatient hospitalization or prolongation of existing hospitalization;
- A persistent or significant disability/incapacity;
- A congenital anomaly/birth defect.

Important medical events that may not result in death, be life threatening, or require hospitalization may be considered an SAE, when, based upon medical judgment, they may jeopardize the patient and may require medical or surgical intervention to prevent one of the outcomes listed in the definition. Any pregnancy occurring on study must be reported via AdEERS as a medically significant event.

Pharmaceutically supported studies will require additional reporting over and above that which is required by CTEP.

SAEs (more than 30 days after last treatment) attributed to the protocol treatment (possible, probable, or definite) should be reported via AdEERS.

All supporting source documentation indicated as being provided in the Additional Information Section of the AdEERS Report must be properly labeled with the study/case numbers and the date of the event and must be faxed to both the NCI at 301-230-0159 and the RTOG dedicated SAE FAX, 215-717-0990, before the five or ten-calendar-day deadline to allow RTOG to comply with the reporting requirements of the pharmaceutical company/companies supporting the RTOG trial. The RTOG Case Number without any leading zeros should be used as the Patient ID when reporting via AdEERS. Non-RTOG intergroup study and case numbers must also be included, when applicable. Submitted AdEERS Reports are forwarded to RTOG electronically via the AdEERS system. Use the patient's case number as the patient ID when reporting via AdEERS.

SAE reporting is safety related and separate and in addition to the Data Management reporting requirements as outlined in the previous AE reporting section. **Any event that meets the above outlined criteria for an SAE but is assessed by the AdEERS System as "expedited reporting NOT required" must still be reported for safety reasons and to fulfill the obligations of RTOG to the pharmaceutical company/companies supporting the RTOG trial. Sites must bypass the "NOT Required" assessment and complete and submit the report. The AdEERS System allows submission of all reports regardless of the results of the assessment. Note:** Sites must select the option in AdEERS to send a copy of the report to the FDA or print the AdEERS report and fax it to the FDA, FAX 1-800-332-0178.

7.8.3 <u>Acute myeloid leukemia (AML) or myelodysplastic syndrome (MDS)</u>

AML or MDS that is diagnosed during or subsequent to treatment in patients on NCI/CTEP-sponsored clinical trials must be reported via the AdEERS system and within 30 days of AML/MDS diagnosis. If you are reporting in CTCAE v 4, the event(s) may be reported as either: 1) Leukemia secondary to oncology chemotherapy, 2) Myelodysplastic syndrome, or 3) Treatment-related secondary malignancy.

7.9 AdEERS Expedited Reporting Requirements (2/13/09)(5/28/09)

CTEP defines expedited AE reporting requirements for phase 3 trials as described in the table below. **Important:** All AEs reported via AdEERS also must be reported on the AE section of the appropriate case report form (see Section 12.1).

AdEERS Expedited Reporting Requirements for Adverse Events that Occur Within 30 Days[1] of the Last Dose of the Investigational Agents in this Study (Radiation Plus Paclitaxel, Cisplatin, and Cetuximab [Arm 1] and Radiation Plus Paclitaxel and Cisplatin [Arm 2])

	Unexpected and Expected	Unexpected	Expected	Unexpected		Expected		Unex-pected	Expected
				with Hospitali-zation	without Hospitali-zation	with Hospitali-zation	without Hospitali-zation		
**Unrelated Unlikely**	Not Required	Not Required	Not Required	10 Calendar Days	Not Required	10 Calendar Days	Not Required	10 Calendar Days	10 Calendar Days
**Possible Probable Definite**	Not Required	10 Calendar Days	Not Required	10 Calendar Days	10 Calendar Days	10 Calendar Days	Not Required	24-Hour; 5 Calendar Days	10 Calendar Days

[1] **Adverse events with attribution of possible, probable, or definite that occur greater than 30 days after the last dose of treatment with an agent require reporting as follows:**
AdEERS 24-hour notification followed by complete report within 5 calendar days for:
- Grade 4 and Grade 5 unexpected events

AdEERS 10 calendar day report:
- Grade 3 unexpected events with hospitalization or prolongation of hospitalization
- Grade 5 expected events

[2] Although an AdEERS 24-hour notification is not required for death clearly related to progressive disease, a full report is required as outlined in the table.

Please see exceptions below under section entitled "Additional Instructions or Exceptions."

March 2005

Note: All deaths on study require both routine and expedited reporting regardless of causality. Attribution to treatment or other cause must be provided. "On study" is defined as during or within 30 days of completing protocol treatment.

- Expedited AE reporting timelines defined:
  - "24 hours; 5 calendar days" – The investigator must initially report the AE via AdEERS within 24 hours of learning of the event followed by a complete AdEERS report within 5 calendar days of the initial 24-hour report.
  - "10 calendar days" - A complete AdEERS report on the AE must be submitted within 10 calendar days of the investigator learning of the event.
- Any medical event equivalent to CTCAE grade 3, 4, or 5 that precipitates hospitalization (or prolongation of existing hospitalization) must be reported regardless of attribution and designation as expected or unexpected with the exception of any events identified as protocol-specific expedited adverse event reporting exclusions.
- Any event that results in persistent or significant disabilities/incapacities, congenital anomalies, or birth defects must be reported via AdEERS if the event occurs following treatment with an agent under a CTEP IND.
- Use the NCI protocol number and the protocol-specific patient ID assigned during trial registration on all reports.

8.0 SURGERY

All patients are required to undergo endoscopy 6 to 8 weeks after the last radiation treatment. Patients deemed to have residual disease or suspicion of residual disease must undergo a biopsy in order to pathologically confirm findings. Those patients who are found to be free of disease are NOT required to undergo repeat biopsy.

If a patient has pathologically proven recurrent/residual disease, further treatment is at the discretion of the treating physician. These patients will continue to be followed for the survival endpoint of this study.

9.0 OTHER THERAPY

9.1 Permitted Therapy

Patients may receive all concomitant therapy deemed necessary to provide adequate support, with the exception of the therapies detailed in Section 9.2. In addition, myeloid growth factors are permitted only to treat grade 4 neutropenia.

All supportive therapy for optimal medical care will be given during the study period at the discretion of the attending physician(s) within the parameters of the protocol and documented on each site's source documents as concomitant medication.

For specific supportive care measures for cetuximab toxicity, see Section 7.6.2.

9.2 Non-Permitted Therapy

9.2.1 Other investigational agents
9.2.2 Other cytotoxic agents
9.2.3 Other radiotherapy

10.0 TISSUE/SPECIMEN SUBMISSION (2/13/09, 3/31/09)

NOTE: Patients must be offered the opportunity to participate in the correlative components of the study, including tissue/specimen submission.

- If the patient consents to participate in the tissue/specimen component of the study, the site is required to submit the patient's specimens as specified in Section 10.0 of the protocol. **Note**: Sites are not permitted to delete the tissue/specimen component from the protocol or from the sample consent.

10.1 Tissue/Specimen Submission

The RTOG Biospecimen Resource at the University of California San Francisco acquires and maintains high quality specimens from RTOG trials. Tissue from each block is preserved through careful block storage and processing. The RTOG encourages participants in protocol studies to consent to the banking of their tissue. The RTOG Biospecimen Resource provides tissue specimens to investigators for translational research studies. Translational research studies integrate the newest research findings into current protocols to investigate important biologic questions. The RTOG Biospecimen Resource also collects tissue for Central Review of pathology. Central Review of tissue can be for eligibility and/or analysis.

In this study, tissue will be submitted to the RTOG Biospecimen Resource for the purpose of tissue banking (strongly encouraged) as described below.

10.2 Specimen Collection for Tissue Banking (strongly encouraged) (5/3/2012)

For patients who have consented to participate in the tissue component of the study (See Appendix I).

Sites may submit the following specimens:

10.2.1 Blocks/Slides

10.2.1.1 One H&E stained slide

10.2.1.2 A paraffin-embedded tissue block of the tumor or a 2-mm diameter core of tissue, punched from the tissue block containing the tumor with a punch tool and submitted in a plastic tube labeled with the surgical pathology number. **NOTE**: A kit with the punch, tube, and instructions can be obtained free of charge from the Biospecimen Resource. Block or core must be clearly labeled with the pathology identification number that corresponds to the Pathology Report.

The following must be provided in order for the case to be evaluable for the Biospecimen Resource:

10.2.1.3 A Pathology Report documenting that the submitted block or core contains tumor. The report must include the RTOG protocol number and patient's case number. The patient's name and/or other identifying information should be removed from the report. The surgical pathology numbers and information must NOT be removed from the report.

10.2.1.4 A Specimen Transmittal Form clearly stating that tissue is being submitted for the RTOG Biospecimen Resource; if for translational research, this should be stated on the form. The form must include the date of collection of the biospecimen; the RTOG protocol number , the patient's case number, time point of study, and method of storage.

10.2.2 Serum

10.2.2.1 Collection Schedule: Specimens will be collected at baseline and 6-8 weeks post-treatment, at the time of post-treatment endoscopy.

10.2.2.2 Collection Instructions: For detailed instructions, see Appendix V. Specimens will be collected in red-top tubes (5-10 mL tubes)

10.2.3 Plasma

10.2.3.1 Collection Schedule: Specimens will be collected at baseline and 6-8 weeks post-treatment, at the time of post-treatment endoscopy.

10.2.3.2 Collection Instructions: For detailed instructions, see Appendix V. Specimens will be collected in tubes containing EDTA #1(purple/lavender-top tubes)

10.2.4 DNA:Whole Blood

10.2.4.1 Collection Schedule: Specimens will be collected at baseline. If site misses this collection then this sample can be collected at any other time point or follow up appointment but this must be noted on the STF..Collection Instructions: For detailed instructions, see Appendix V. Specimens will be collected in tubes containing EDTA #2 (purple/lavender-top tubes)

10.2.5 Urine

10.2.5.1 Collection Schedule: Specimens will be collected at baseline and 6-8 weeks post-treatment, at the time of post-treatment endoscopy.

10.2.5.2 Collection Instructions: For detailed instructions, see Appendix V. 10-20mL of clean –catch urine should be collected in a sterile collection cup and aliquotted into two labeled 15 ml centrifuge tubes before freezing sample

10.2.6 Storage Conditions

Store frozen specimens at -80° C (-70°C to -90°C) until ready to ship. If a -80°C Freezer is not available:

- Samples can be stored short term in a -20°C freezer (non-frost free preferred) for up to one week (please ship out Monday-Wednesday only; Canada: Monday-Tuesday).

OR:

- Samples can be stored in plenty of dry ice for up to one week, replenishing daily (ship out Monday-Wednesday only; Canada: Monday-Tuesday).

OR:

- Samples can be stored in liquid nitrogen vapor phase (ship out Monday-Wednesday only; Canada: Monday-Tuesday).

Please indicate on Specimen Transmittal Form the storage conditions used and time store

10.2.7 Specimen Collection Summary

**Specimens taken from patient:**	**Submitted as:**	**Shipped:**
Representative H&E stained slide of the primary tumor	H&E stained slide	Slide shipped ambient
A paraffin-embedded tissue block of the primary tumor taken before initiation of treatment or a 2 mm diameter core of tissue, punched from the tissue block with a punch tool	Paraffin-embedded tissue block or punch biopsy	Block or punch shipped ambient Recommended: use cold packs to prevent the block from melting during warm weather.
SERUM: 5-10 mL of whole blood in 1 red-top tube and centrifuge f	Frozen serum samples containing 0.5 mL per aliquot in 1 mL cryovials (five to ten)	Serum sent frozen on dry ice via overnight carrier
PLASMA: 5-10 mL of anticoagulated whole blood in EDTA #1 tubes (purple/lavender top) and centrifuge	Frozen plasma samples containing 0.5 mL per aliquot in 1 mL cryovials (five to ten)	Plasma sent frozen on dry ice via overnight carrier
DNA: 5-10 mL of anticoagulated whole blood in EDTA #2 tubes (purple/lavender top) and mix	Frozen whole blood samples containing 1 mL per aliquot in 1 mL cryovials	Whole blood sent frozen on dry ice via overnight carrier
10-20 mL clean-catch urine	Two 5-10 mL urine aliquots in 2 sterile 15 ml polypropylene centrifuge tubes. Store frozen at -20° or 80° C	Urine sent frozen on dry ice via overnight carrier

10.2.7 Submit materials as follows:

US Postal Service Mailing Address: For Non-Frozen Specimens by USPS
RTOG Biospecimen Resource
University of California San Francisco
Campus Box 1800
2340 Sutter St, Room S341
San Francisco, CA 94143-1800

Courier Address (FedEx, UPS, etc.): For Frozen Specimens or Trackable shipments
RTOG Biospecimen Resource
University of California San Francisco
2340 Sutter St, Room S341
San Francisco, CA 94143-1800

Questions: 415-476-RTOG (7864)/FAX 415-476-5271; RTOG@ucsf.edu

10.3 Reimbursement (9/13/2011)

RTOG will reimburse submitting institutions $300 per case for buffy coat cells, serum, and plasma; $200 per case for a block or core of material; $100 per case for unstained slides; and $50 per case for urine. After confirmation from the RTOG Biospecimen Resource that appropriate materials have been received, RTOG Clinical Trials Administration will authorize payment according to the schedule posted with the Reimbursement and Case Credit Schedule found on the RTOG web site (http://www.rtog.org/LinkClick.aspx?fileticket=Csxzt1v1hEk%3d&tabid=323)

Biospecimen payments will be processed quarterly and will appear on the institution's summary report with the institution's regular case reimbursement. RTOG Administration will prepare the proper paperwork and send a check to the institution. Pathology payment cycles are run twice a year in January and July and will appear on the institution's summary report with the institution's regular case reimbursement.

10.4 Confidentiality/Storage (2/13/09)

(See the RTOG Patient Tissue Consent Frequently Asked Questions, http://www.rtog.org/Researchers/BiospecimenResource/BiospecimenResourceFAQs.aspx for further details.)

10.4.1 Upon receipt, the specimen is labeled with the RTOG protocol number and the patient's case number only. The RTOG Biospecimen Resource database only includes the following information: the number of specimens received, the date the specimens were received, documentation of material sent to a qualified investigator, type of material sent, and the date the specimens were sent to the investigator. No clinical information is kept in the database.

10.4.2 Specimens for tissue banking will be stored for an indefinite period of time. If at any time the patient withdraws consent to store and use specimens, the material will be returned to the institution that submitted it.

11.0 PATIENT ASSESSMENTS

11.1 Study Parameters: See Appendix II for a summary of assessments and time frames.

11.2 Measurement of Response

NOTE: Any images from endoscoping procedures up to the time of progression must be kept in the patient's confidential study file.

All patients will undergo a repeat endoscopy 6-8 weeks after the completion of chemoradiation. At the time of endoscopy a visual inspection of the site of the original primary disease must be documented.

- Those patients who are found to be free of disease are NOT required to undergo repeat biopsy. These patients will be scored as clinical complete responses.
- Patients deemed to have residual disease or suspicion of residual disease must undergo a biopsy in order to pathologically confirm findings. Any patient with pathologically confirmed residual disease will be scored as a local failure. Patients who are pathologically proven to have no evidence of disease will be scored as clinical complete responders.

11.3 Disease Assessment in Follow-Up for Complete Responders Only

For patients determined to be clinical complete responders, per Section 11.2, if there is evidence or suspicion of disease on follow-up CT scans, biopsy is required to confirm recurrence of disease.

11.4 Criteria for Discontinuation of Protocol Treatment

- Progression of disease

  If protocol treatment is discontinued, follow-up and data collection will continue as specified in the protocol.

11.5 Quality of Life and Health Utility Assessments (3/31/09)

NOTE: Patients must be offered the opportunity to participate in the correlative components of the study, including quality of life assessment.

- If the patient consents to participate in the quality of life component of the study, sites are required to administer the assessments below.

11.5.1 *Functional Assessment of Cancer Therapy for Esophageal Cancer (FACT-E)* is a multidimensional, QOL instrument specifically designed and validated for use with patients with esophageal cancer patients that the patient can complete in 10 minutes. The site research nurse or CRA is encouraged to be sensitive to each patient's demeanor. If patients appear particularly uncomfortable answering a question, they will be informed that they can skip that question. Similarly, interviewers will give patients a short break if the patient appears fatigued or otherwise in need of a few minutes break. Note: The FACT-E has been translated into 16 languages and is available free of charge to institutions with the completion of an agreement to share data, accessible at http://www.facit.org/translation/licensure.aspx.

11.5.2 *The EuroQol (EQ-5D)* is a two-part questionnaire that the patient can complete in approximately 5 minutes. Note: The EQ-5D has been translated into multiple languages; these translations are available from the EuroQol web site at http://www.euroqol.org/. The site research nurse or CRA should encourage the patient not to skip questions on the EQ-5D or take breaks during the completion of this questionnaire, as this will invalidate the assessment. If this occurs, sites will document it on the Health Utility Measurement **(HP)** form.

12.0 DATA COLLECTION

Data should be submitted to:

RTOG Headquarters*
1818 Market Street, Suite 1600
Philadelphia, PA 19103

***If a data form is available for web entry, it must be submitted electronically.**

Patients will be identified by initials only (first middle last); if there is no middle initial, a hyphen will be used (first-last). Last names with apostrophes will be identified by the first letter of the last name.

12.1 Summary of Data Submission (2/13/09)

Item	Due
Demographic Form **(A5)**<br>Initial Evaluation Form **(I1)**<br>Pathology Report **(P1)**<br>Slides/Blocks **(P2)**<br>Quality of Life/Health Utility Forms<br>▪ FACT-E **(FA)**<br>▪ EQ-5D **(HP)**	Within 2 wks of registration
Treatment Form **(TF)**	Within 1 wk of systemic treatment completion
Post-Treatment Response Form **(F2)**<br>Post-Treatment Pathology Report **(S5)**	Within 1 wk of post-treatment endoscopy
Quality of Life/Health Utility Forms<br>▪ FACT-E **(FA)**<br>▪ EQ-5D **(HP)**	Within 1 wk of post-treatment endoscopy, 1 yr from start of treatment, and 2 yrs from start of treatment
Follow-Up Form **(F1)**	Every 4 mos from the start of RT x 2 yrs, every 6 months for yrs 3 and 4, then annually<br><br>At the time of death
Death Certificate	At the time of death (with final F1)

12.2 Summary of Dosimetry Digital Data Submission for 3D-CRT (Submit to ITC; see Section 12.2.1) (9/13/2011)

Item	Due
**Preliminary Dosimetry Information (DD)**	
Digital Data Submission Form – Treatment Plan submitted to ITC via SFTP account exported from treatment planning machine by Physicist.	Within 1 week of start of RT
Digital data submission includes the following: (NOTE: Submit both heterogeneous and homogeneous treatment plans for every case. See Section 6.1.5 for specific instructions for submission)	
CT data, critical normal structures, all GTV, CTV, and PTV contours **(C1, C3)**	
Digital beam geometry for initial and boost beam sets	
Doses for initial and boost sets of concurrently treated beams	
Digital DVH data for all required critical normal structures, GTV, CTV, and PTVs for total dose plan **(DV)**	
Digital Data Submission Information **(DDSI)** – Submitted online (Form located on ATC web site, http://atc.wustl.edu/forms/DDSI/ddsi.html	
Hard copy isodose distributions for total dose plan as described in QA guidelines† **(T6)**	
**NOTE: Sites must notify ITC via e-mail (itc@castor.wustl.edu) after digital data is submitted. The e-mail must include study and case numbers or, if the data is phantom, "dry run" or "benchmark".**	
**Final Dosimetry Information**	Within 1 week of RT end
Radiotherapy Form **(T1) [copy to HQ and ITC]**	
Daily Treatment Record **(T5) [copy to HQ and ITC]**	
Modified digital patient data as required through consultation with Image Guided Therapy QA Center	

†Available on the ATC web site, http://atc.wustl.edu/

NOTE: ALL SIMULATION AND PORTAL FILMS AND/OR DIGITAL FILM IMAGES WILL BE KEPT BY THE INSTITUTION AND ONLY SUBMITTED IF REQUESTED.

12.2.1 *Digital Data Submission to ITC*

Digital data submission may be accomplished using media or the Internet.

For network submission: The SFTP account assigned to the submitting institution by the ITC shall be used, and e-mail identifying the data set(s) being submitted shall be sent to:

itc@castor.wustl.edu

For media submission: Please contact the ITC about acceptable media types and formats.

Hardcopies accompanying digital data should be sent by mail or Federal Express and should be addressed to:

Image-Guided Therapy Center (ITC)
ATTN: Roxana Haynes
4511 Forest Park, Suite 200
St. Louis, MO 63108
314-747-5415/ FAX 314-747-5423

13.0 STATISTICAL CONSIDERATIONS

13.1 Study Endpoints

13.1.1 *Primary Endpoint*

- Overall survival (OS) (failure: death due to any cause)

13.1.2 *Secondary Endpoints*

- Local control (failure: residual cancer on post-treatment endoscopic biopsy or recurrent primary disease as defined in Section 11.3)
- Adverse events
- Endoscopic complete response rate
- Health-related quality of life (FACT-E)
- Quality-adjusted survival (using EQ-5D), if primary hypothesis is supported
- Implementation of a well-controlled tissue handling/storage facility to facilitate future laboratory correlative studies

13.2 Sample Size

13.2.1 *Stratification*: Patients will be stratified before randomization with respect to histology (adenocarcinoma vs. squamous), cancer lesion size ($<$ 5cm vs. $\geq$ 5cm) and disease status of celiac nodes (present vs. absent). The treatment allocation scheme described by Zelen,[54] based on a permuted block randomization, will be used because it balances patient factors other than institution, while using a dynamic balancing within institutions. Patients will be randomized to 50.4 Gy external beam radiation given concurrently with 6 cycles of paclitaxel and cisplatin with or without cetuximab. The randomization will occur centrally at the RTOG Statistical Center. The treatment arms will be balanced within strata cell and institution.

13.2.2 *Sample Size Derivation*: The sample size calculations are based on the primary hypothesis that the addition of cetuximab to external beam radiation and paclitaxel and cisplatin will increase the 2-year survival of patients with locally advanced esophageal cancer from 41% to 53%.

The standard radiation dose arm with concurrent 5-FU and cisplatin from RTOG 94054 showed a 2-year survival of 41%. Building on RTOG 9405, this trial will use concurrent cisplatin and paclitaxel as the chemotherapy backbone, a regimen that has been shown to be effective in both institutional and cooperative group trials.14,55,56 The current study will evaluate 50.4 Gy external beam radiation given concurrently with 6 cycles of paclitaxel and cisplatin with or without cetuximab.

The required sample size for the primary endpoint of overall survival is based on the following conditions:

- Survival times are exponentially distributed with (at least approximately) constant hazards in both treatment arms
- The control arm will have a 2-year survival of 41% (yearly hazard of 0.4458)
- The experiment arm will have a 2-year survival of 53% (yearly hazard of 0.3174)
- One-sided test at α = 0.025
- Statistical power of 80%
- 4.5 years of accrual with 2 years of follow-up
- Four interim significance tests and a final test are planned using EaST® software

Using the group sequential design method with 4 interim analyses, 281 deaths are required to detect an absolute survival benefit of 12%, translating into a hazard ratio of 1.4. Given the conditions above, a total sample size of 400 will be required to be accrued uniformly over 4.5 years with an additional 2 years of follow-up. Guarding against an ineligibility or lack-of-data rate of up to 5%, **the final targeted accrual for this study will be 420 patients**.

13.2.3 *Statistical Power Information for Secondary Endpoints:*

13.2.3.1 Local Control

Assuming a 2-year local control rate of 48%, based on the RTOG 9405 control arm, 400 analyzable patients will provide 85% statistical power to detect a 12% increase in local control (hazard ratio of 1.44).

13.2.3.2 Clinical (Endoscopic) Complete Response Rate: See Section 13.4.3.1

13.2.3.3 HRQOL FACT-E

The Functional Assessment of Cancer Therapy – Esophagus (FACT-E) will be used to measure HRQOL, with the focus on the Esophageal Cancer Subscale (ECS). Protocol-eligible patients will be included in the QOL analysis only if they have provided baseline and at least one subsequent measurement. The FACT-E will be collected on all cases participating in this portion of the trial and will be collected at four time points: pretreatment (baseline), 6-8 weeks following chemoradiation with or without cetuximab at the time of clinical complete response evaluation, 1 year from start of treatment, and 2 years from start of treatment.

13.2.3.3.1 The primary HRQOL endpoint will be to determine if the addition of cetuximab to chemoradiation improves the FACT-E Esophageal Cancer Subscale (ECS) score, as measured by the proportion of patients on each treatment arm with improvement, defined as an increase in the ECS score of at least 5 points.[44] Given the recent development and validation of this tool, the power calculations shown below cover a number of possible proportions for improvement in the control arm. The power calculations are all based on a 1-sided, α=0.05, chi-squared test and the assumption of an 80% participation rate.

Power Calculations for ECS Score

p_0	p_a	n/arm*	power
0.30	0.45	160	84
0.30	0.50	160	97
0.40	0.55	160	82
0.40	0.60	160	96
0.50	0.65	160	83
0.50	0.70	160	97

* If the participation rate is higher, there will be more power to detect the hypothesized differences; if the participation rate is lower, there will be less power.

13.2.3.3.2 A secondary HRQOL endpoint will be to determine if the addition of cetuximab to chemoradiation improves the Swallowing Index Subscale Score of the FACT-E ECS (items hn7, e1, e2, e3, and e5 from the ECS), as measured by the proportion of patients on each treatment arm with improvement, defined as an increase in the ECS score of at least 4 points.[44] The power calculations, with the same assumptions, are the same as shown in 13.2.3.3.1.

13.2.3.3.3 A secondary HRQOL endpoint will be to determine if the addition of cetuximab to chemoradiation improves the Eating Index Subscale Score of the FACT-E ECS (items hn1, hn5, and e6), as measured by the proportion of patients on each treatment arm with improvement, defined as an increase in the ECS score of at least 2 points.[44] The power calculations, with the same assumptions, are the same as shown in 13.2.3.3.1.

13.2.3.3.4 A secondary HRQOL endpoint will be to determine if clinical complete response correlates with the ECS Score at 1 year and/or 2 years from the start of treatment.

13.2.3.4 The EQ-5D will be used to assess quality-adjusted survival. Protocol-eligible patients will be included in the QOL analysis only if they have provided a baseline and at least one subsequent measurement. The EQ-5D will be collected on all cases participating in this portion of the trial and will be collected at four time points: pretreatment (baseline), 6-8 weeks following chemoradiation with or without cetuximab at the time of clinical complete response evaluation, 1 year from start of treatment, and 2 years from start of treatment.

13.3 Patient Accrual

Given that there will not be any major phase III studies competing for this patient population and there will be participation from other cooperative groups through the CTSU mechanism, the patient accrual is projected to be 8 cases per month, with a ramp-up period in the first 6 months. The expected monthly accruals in months 1-3 and months 4-6, following activation, are 0 and 3 respectively. We expect to complete the accrual in 4.5 years. The total duration of the study is expected to be 6.5 years from the time the first patient is entered to the overall survival analysis. Based on CTEP's early stopping guidelines for slow accruing trials:

- If the average monthly accrual rate during months 12 to 18 following activation is below 2 cases per month, the study statistician will recommend to the RTOG Data Monitoring Committee (DMC) that the study be closed to accrual.
- If the average monthly accrual rate during months 12 to 18 following activation is [2,4) cases per month, the study will be given 6 months for the average monthly accrual to increase to at least 4. If the average accrual rate in quarter 8 is still between 2 and 4 then the trial will be amended to reflect actual accrual, including the implications of this new accrual rate on study relevance and feasibility. If the accrual rate is below 2 cases per month in quarter 8, the study statistician will recommend to the RTOG DMC that the study be closed to accrual.

13.4 Analysis Plan (9/13/2011)

All analyses will be done based on the assigned treatment arm for the following patient populations: (1) all eligible patients entered, and (2) all patients entered regardless of eligibility status.

13.4.1 *Statistical Methods*

13.4.1.1 Overall Survival*:* OS will be estimated by the Kaplan-Meier method.[57] The distribution of OS estimates between the 2 arms will be compared with a stratified log rank test,[58] using the stratification variables from randomization. Survival time will be measured from the date of randomization to the date of death or last follow-up. The Cox proportional hazard regression model will be used to analyze the effects of factors, in addition to treatment, including protocol treatment compliance that may be associated with OS.

13.4.1.2 Local Control

13.4.1.2.1 Local control will be estimated by the Kaplan-Meier method.[57] The distribution of local control estimates between the 2 arms will be compared using the stratified log rank test.[58] Local control time will be measured from date of randomization to the date of first local failure or last follow-up for patients who have not failed. Patients with residual cancer on post-treatment endoscopic biopsy will be considered failures at day 1. Patients alive without disease at time of analysis will be censored as of their last follow-up status date where local tumor status is known.

13.4.2.1.2 Adverse Events: A chi-squared test will be performed to test for differences in the proportion of grade 4+ non-hematologic treatment-related toxicity occurring from the start of treatment up to 90 days from the end of treatment.

13.4.1.3 Endoscopic Complete Response

Endoscopic complete response will be measured per Section 11.2. Early looks at endoscopic complete response will be performed per Section 13.4.3.1. At the end of the study, a chi-squared test will be performed to test for differences in the proportion of patients with an endoscopic complete response between the treatment arms.

13.4.2 *Interim Analysis to Monitor the Study Progress*: Interim reports with statistical analyses will be prepared twice per year until the initial treatment results have been presented/published. In general, the interim reports will contain the following information:

- patient accrual rate with a projected completion date (while the study is still accruing)
- total patients accrued
- distributions of important pretreatment and prognostic baseline variables
- the frequencies and severity of adverse events by treatment arm.
- compliance rates of treatment delivery

The interim reports will not contain the results from the treatment comparisons with respect to the primary endpoint, overall survival, or any secondary endpoints.

13.4.3 *Significance Testing for Early Termination and/or Reporting*:

13.4.3.1 Clinical (Endoscopic) Complete Response Effect within Histology Type

If the experimental treatment shows a promising effect on clinical complete response (CCR) in the first 150 adenocarcinoma patients, then the study will continue on to a total of 420 adenocarcinoma and squamous cell cancer (SCC) patients combined, barring early stopping as described in Section 13.4.3.3. Using a 1-sided significance level of 0.10 and assuming a CCR of 40% for the standard arm, 150 patients will provide 84% power to detect and absolute increase of 20% in CCR rates. An observed increase of 12% (40% to 52%) in CCR rates would be sufficient to achieve statistical significance at the 1-sided 0.10 level and will thus be used as the criteria for continuation of accrual. If this increase in CCR for the adenocarcinoma patients is not seen, then further accrual will be limited to SCC patients and

the same test for an effect on CCR will be performed on the first 150 eligible SCC patients under the same conditions, with one exception. If the experimental arm is determined to be promising, then accrual will continue to a total of 272 SCC patients, as the hypothesized experimental 2-year survival for SCC only patients will be 56%. All other parameters as described in Section 13.2.2 will remain the same. All patients are required to undergo an endoscopy 6 to 8 weeks after the last radiation treatment. The results of this procedure will be reported on the Post-Treatment Response Form (F2), due within 1 week of the endoscopy. The analysis for each histology will be performed when complete data is received with respect to each histology.

13.4.3.2 Unacceptable Rate of Toxicity: To address the safety of cetuximab in combination with radiation and the chosen chemotherapy regimen, the rate of unacceptable adverse events (defined as grade 4+ non-hematologic treatment-related toxicity occurring within 90 days from the end of treatment) will be evaluated for the experimental arm after 25 and 50 patients have been entered and are evaluable (eligible patients with toxicity data) on that arm. The study chairs have determined that a rate of 35% or greater will be considered to be unacceptable. According to Flemming's method[59] with a maximum overall significance level of 0.05 if there are:

10 or more patients with unacceptable adverse events out of the first 25 evaluable patients,

OR

25 or more patients with unacceptable adverse events out of the first 50 evaluable patients

The study will have exceeded the limit for unacceptable adverse events. If the number of unacceptable adverse events crosses a boundary, as described in the rules above, then the conclusion will be that the treatment-related unacceptable adverse event rate is greater than 35%. If this circumstance occurs, the study chairs, the RTOG gastrointestinal cancer committee chair, and the statistician will review the adverse event data and make appropriate recommendations to the RTOG executive committee about the study. These stopping rules provide 85% power for concluding that the unacceptable adverse event rate is equal to or exceeds 35% when in fact that is the true rate.

13.4.3.3 Primary Endpoint: Overall Survival

Four interim significance tests of treatment difference are planned. The timing of the interim analyses will be based on primary endpoint events, deaths. The maximum number of deaths required for the study is 281. Under the alternative hypothesis that the addition of cetuximab will increase overall survival from 41% to 53%, the projected numbers of deaths and the nominal significance levels for rejecting the H0 or the H1 for each of these 4 interim analyses, along with the projected timing and accrual, are shown in the table below:

Nominal Significance Levels for Interim Analyses

Interim Analysis	Efficacy: Reject H_0 if $p(H_0) \leq$	Futility: Reject H_1 if critical value ≤	# events (deaths)	Projected time (mths)	Projected accrual
#1	0.0001	**-1.3058**0.005	57	24	182
#2	0.0004	**-0.787**0.005	113	36	278
#3	0.0037	**-0.3889**0.005	169	46	368
#4	0.011	**-0.0525** 0.005	225	54	400

At each planned interim analysis, the one-sided p-value from the log-rank test assessing treatment efficacy with respect to overall survival will be compared to the nominal significance levels in the table above. These levels are based on the Lan-DeMets alpha spending function[60] that behaves like the O'Brien-Flemming boundary.[61] If the computed p-value for efficacy is less than or equal to the nominal significance level boundary for rejecting the H_0 (efficacy), then we will stop accrual to the trial (if applicable), conclude that

the overall survival rate of the cetuximab arm (Arm 1) is higher than that of the non-cetuximab arm (Arm 2), and report the results. For futility, the alternative hypothesis will be tested using rule C from Freidlin and Korn[62] based on a significance level of 0.005. If the computed critical value is less than or equal to the critical value (from the above table) for rejecting the H_1 (futility), then we will stop accrual to the trial (if applicable) and will report that we <u>cannot</u> conclude that the OS rate of the cetuximab arm (Arm 1) is higher than that of the non cetuximab arm (Arm 2). If neither of these boundaries are crossed, accrual (if applicable) and follow-up will continue until the next interim or final analysis.

As the RTOG DMC meets semi-annually, if, in preparation for an upcoming DMC meeting, the number of events is not exactly equal to the specified number in the table above and the decision is made to present the interim efficacy analysis to the DMC at that meeting, the spending function must be adjusted to reflect the actual number of events.

In addition to the results listed in Section 13.4.2, at the first RTOG DMC meeting following the required number of deaths for each planned interim analysis, blinded efficacy results will be reported to the RTOG DMC.

Phase III trials are required by NCI Cooperative Group Program Guidelines to be reviewed by a data and safety monitoring committee. This study will be reviewed by the RTOG DMC on a semi-annual basis in January and June.

13.4.4 *<u>Analysis for Endpoints Related to HRQOL</u>* *<u>(Collected for patients who consent to this component of the study)</u>*

We will describe the distributions of QOL data collection patterns over all collection points in each treatment arm. To inspect the missing data mechanism for each tool, we will use at least a graphical method. A missing completely at random (MCAR) mechanism exists when missing values are randomly distributed across all observations. A missing at random (MAR) mechanism exists when values are not randomly distributed across all observations, rather than one or more sub-samples.

If the cause of missing data is MCAR, listwise deletion (complete case analysis) will be done. If the MAR assumption is supported by the data, then an imputation method such as multiple imputation will be applied to impute missing data.

If the MAR assumption is not supported by the data, then adjusting for covariates (such as the baseline QOL score) might reduce the conditional association between outcomes and missing values. If missing data patterns look similar when stratified by such covariate(s), then an analysis that adjusts for such covariate(s) will be conducted and an imputation method such as multiple imputation will be applied. If approximate conditional independence cannot be obtained with any set of covariates, then MNAR (missing not at random) must be addressed by an explicit model for the missing data mechanism[63] and then an imputation method such as multiple imputation will be applied. All results from the imputed analysis using the multiple imputation will be compared to the complete case analysis results to assess any potential biases.

13.4.4.1 <u>FACT-E Scoring and Analysis</u>

13.4.4.1.1 The FACT-E will be scored per the FACT-E Scoring Guidelines (Version 4 www.facit.org), with higher scores indicating better QOL.

13.4.4.1.2 The primary objective in the HRQOL analysis is improvement in the ECS Score. Improvement in the ECS Score, the ECS Swallowing Index Score, and the Eating Index Score are defined as increases of 5 points, 4 points, and 2 points respectively, from baseline to the 6-8 week following treatment completion time point. Chi-squared tests will be used to test the null hypothesis that the proportion of patients categorized as "improved" will be the same for the two treatment arms, versus the alternative hypothesis that the proportion of patients categorized as "improved" is higher for the cetuximab arm.

13.4.4.1.3 Logistic regression models will be used to determine if clinical complete response at 6-8 weeks following treatment completion is associated with ECS Score.

13.4.4.2 <u>EQ-5D Scoring and Analysis</u>

13.4.4.2.1 The quality-adjusted survival of each treatment will be evaluated and compared using EQ-5D if the primary endpoint supports the primary hypothesis.

13.4.4.2.2 The EQ-5D is a two-part self-assessment questionnaire. The first part consists of five items covering five dimensions (mobility, self care, usual activities, pain/discomfort, and anxiety/depression). Each dimension is measured by a three-point likert scale (1-no problems, 2-moderate problems and 3-extreme problems). The second part is a visual analog scale (VAS) valuing the current health state measured by a 100-point scale with a 10-point interval (0-worst imaginable health state, 100-best imaginable health state). We will transform the five-item index score and VAS score into a utility score between 0 (worst health state) and 1 (best health state) for comparative purposes.

13.4.4.2.3 To examine trade-offs between the survival time and QOL, we will combine them for each patient into a single measurement: Quality Adjusted Life Years (QALY). If (and only if) the primary endpoint hypothesis is substantiated, we will conduct a quality-adjusted survival analysis. The quality-adjusted survival analysis will not be done until after the primary endpoint results are published. QALY is defined by the weighted sum of different time episodes added up to a total quality-adjusted survival time. QALY will be analyzed at two time points: at 1 year and 2 years from start of treatment, using the EQ-5D.

13.4.5 *Analysis For Reporting the Initial Treatment Results*: The primary hypothesis of this study is whether the addition of cetuximab to external beam radiation plus paclitaxel and cisplatin will increase the overall survival of patients with locally advanced esophageal cancer. This major analysis will occur after at least 281 deaths have been observed, unless an early stopping rule is satisfied. The analysis will include:

- tabulation of all cases entered and those excluded from the analyses with the reasons for exclusion given
- distributions of important prognostic baseline variables
- the frequencies and severity of adverse events by treatment arm
- compliance rate of treatment delivery
- observed results with respect to the primary and secondary endpoints

The primary hypothesis of treatment benefit will be tested using the stratified log-rank statistic with a 1-sided significance level of 0.021, given that the 4 interim analyses were carried out per Section 13.4.3.3. Additional analyses of treatment effect will be performed using the Cox proportional hazard model with the major stratification factors included as fixed covariates, as well as other possible modifying factors such as age, gender, race, and other patient characteristics. The treatment comparison of local control will be analyzed in a similar fashion. Also, where feasible, treatment comparisons with respect to the primary endpoint (overall survival) and secondary endpoints such as local control will be performed within each histology as well as within ethnic and racial categories. If feasible, these analyses will be done both univariately for treatment and multivariately with Cox proportional hazard models, adjusting for the covariates listed above.

13.4.6 CDUS Monitoring: This study will be monitored by the Clinical Data Update System (CDUS) version 3.0. Cumulative CDUS data will be submitted quarterly by electronic means. Reports are due January 31, April 30, July 31, and October 31.

13.5 Gender and Minorities

In conformance with the national Institutes of Health (NIH) Revitalization Act of 1993 with regard to inclusion of women and minorities in clinical research, we have also considered the possible interaction between race/ethnicity and treatment. Based on RTOG studies 9405 and 0113, it is projected that 72% of the patients will be men and 28% women; 3% will be of Hispanic or Latino ethnicity and 97% will not; racial distribution will be 73% white, 26% black or African American, and 1% Asian. The following table lists the projected accrual by ethnic and racial categories. Assuming no difference among races with respect to overall survival, the statistical power for detecting the hypothesized difference is 10% and 78% for Hispanic or Latino and Non-Hispanic or Latino, respectively, and 66% and 29% for white and black or African-American, respectively.

Projected Distribution of Gender and Minorities (6/22/10)

	Gender		
**Ethnic Category**	**Females**	**Males**	**Total**
Hispanic or Latino	4	9	13
Not Hispanic or Latino	118	289	407

	Gender		
**Ethnic Category**	**Females**	**Males**	**Total**
**Ethnic Category: Total of all subjects***	122	298	420
	**Gender**		
**Racial Category**	**Females**	**Males**	**Total**
American Indian or Alaskan Native	0	0	0
Asian	1	3	4
Black or African American	30	79	109
Native Hawaiian or other Pacific Islander	0	0	0
White	87	220	307
**Racial Category: Total of all subjects***	118	302	420

REFERENCES

1. Jemal A, Siegel R, Ward E, et al. Cancer Statistics, 2006. Ca Cancer J Clin 2006;56:106-30.

2. Cooper JS, Guo MD, Herskovic A, et al. Chemoradiotherapy of locally advanced esophageal cancer: long-term follow-up of a prospective randomized trial (RTOG 85-01). Radiation Therapy Oncology Group. JAMA 1999;281:1623-7.

3. Herskovic A, Martz K, al-Sarraf M, et al. Combined chemotherapy and radiotherapy compared with radiotherapy alone in patients with cancer of the esophagus. N Engl J Med 1992;326:1593-8.

4. Minsky BD, Pajak TF, Ginsberg RJ, et al. INT 0123 (Radiation Therapy Oncology Group 94-05) phase III trial of combined-modality therapy for esophageal cancer: high-dose versus standard-dose radiation therapy. J Clin Oncol 2002;20:1167-74.

5. Safran H, DiPetrillo T, Akerman P, et al. Trastuzumab, paclitaxel, cisplatin and radiation for locally advanced, HER2 overexpressing, adenocarcinoma of the esophagus. Int J Rad Oncol Biol Phy 2006. In Press.

6. Adelstein DJ, Rice TW, Rybicki LA, et al. Does paclitaxel improve the chemoradiotherapy of locoregionally advanced esophageal cancer? A nonrandomized comparison with fluorouracil-based therapy. J Clin Oncol 2000;18:2032-9.

7. Ajani JA, Ilson D, Dougherty K, et al. Activity of Taxol in patients with squamous cell carcinoma and adenocarcinoma of the esophagus. J Natl Cancer Inst 1994;86:1086-91.

8. Ozawa S, Ueda M, Ando N, et al. Prognostic significance of epidermal growth factor receptor in esophageal squamous cell carcinoma. Cancer 1989;63:2169-73.

9. Bedenne L, Michel P, Bouche O, et al. Randomized phase III trial in locally advanced esophageal cancer: radiochemotherapy followed by surgery versus radiochemotherapy alone (abstract). Proc Am Soc Clin Oncol 2002;21:130a. Abstract 519

10. Stahl M, Stuschke M, Lehmann N, et al. Chemoradiation with and without surgery in patients with locally advanced squamous cell carcinoma of the esophagus. J Clin Oncol 2005;232:2310-7.

11. Choy H, Rodriguez FF, Koester S, et al. Investigation of Taxol as a potential radiation sensitizer. Cancer 1993;71:3774-8.

12. Gupta N, Hu LJ, Deen DF. Cytotoxicity and cell-cycle effects of paclitaxel when used as a single agent and in combination with ionizing radiation. Int J Rad Oncol Biol Phys 1997;37:885-95.

13. Liebmann J, Cook JA, Fisher J, et al. In vitro studies of Taxol as a radiation sensitizer in human tumor cells. J Natl Cancer Inst 1994;86:441-6.

14. Safran H, Gaissert H, Akerman P, et al. Paclitaxel, cisplatin and concurrent radiation for esophageal cancer. Cancer Invest 2001;1:1-7.

15. Brenner B, Ilson DH, Minsky BD. Phase I trial of combined-modality therapy for localized esophageal cancer: escalating doses of continuous-infusion paclitaxel with cisplatin and concurrent radiation therapy. J Clin Oncol 2004;22:45-52.

16. Anderson SE, Minsky BD, Bains M, et al. Combined modality therapy in esophageal cancer: the Memorial experience. Semin Surg Oncol 2003;4:228-32.

17. Gray JR, Meluch AA, Kalman L. Neoadjuvant paclitaxel/carboplatin/5-FU/radiation therapy for localized esophageal cancer: update of a Minnie Pearl Cancer Research Network phase II trial. Proc Am Soc Clin Oncol 1999:18. Abstr 1040.

18. Ilson DH. Cancer of the gastroesophageal junction: current therapy options. Curr Treat Options Oncol 2006;7:410-23.

19. Urba S, Hayman J, Ianettonni M, et al. Pre-operative chemoradiation with cisplatin (CDDP),5-fluorouracil (5FU), and paclitaxel (Tax), followed by surgery and adjuvant chemotherapy, for loco-regional esophageal carcinoma. 2004 ASCO Annual Meeting Proceedings (Post-Meeting Edition). 2004;22:14S. Abstr 4029.

20. Burmeister BH, Smithers BM, Gebski V, et al. Trans-Tasman Radiation Oncology Group; Australasian Gastro-Intestinal Trials Group. Related Articles, Links Surgery alone versus chemoradiotherapy followed by surgery for resectable cancer of the oesophagus: a randomised controlled phase III trial. Lancet Oncol 2005;6:659-68.

21. Mendelsohn J, Baird A, Fan Z, Markowitz SD. Growth factors and their receptors in epithelial malignancies, in Mendelsohn J, Howley PM, Israel MA, Liotta LA, eds. The Molecular Basis of Cancer. Philadelphia, PA: W.B. Saunders Company 2001, pp 137-4.

22. Huang SM, Harari PM. Epidermal growth factor receptor inhibition in cancer therapy: biology, rationale and preliminary clinical results. Invest New Drugs 1999;17:259-69.

23. Hackel PO, Zwick E, Prenzel N, et al. Epidermal growth factor receptors: critical mediators of multiple receptor pathways. Curr Opin Cell Biol 1999;11:184-9.

24. Makuda H, Toi M, Hirai T, et al. Clinical significance of the expression of epidermal growth factor and its receptor in esophageal cancer. Cancer 1991;68:142-8.

25. Hickey K, Grehan D, Reid IM, et al. Expression of epidermal growth factor receptor and proliferating cell nuclear antigen predicts response of esophageal squamous carcinoma to chemoradiotherapy. Cancer 1994;74:1693-8.

26. Gibson MK, Abraham SC, Wu TT, et al. Epidermal growth factor receptor, p53 mutation and pathological response predict survival in patients with locally advanced esophageal cancer treated with preoperative chemotherapy. Clin Ca Res 2003;9:6461-8.

27. Janmaat ML, Gallegos-Ruiz MI, Rodriguez JA, et al. Predictive factors for outcome in a phase II study of gefitinib in second-line treatment of advanced esophageal cancer patients. J Clin Oncol 2006;24:1612-9.

28. Dragovich T, McCoy S, Fenoglio-Preiser CM, et al. Phase II trial of erlotinib in gastroesophageal junction and gastric adenocarcinomas: SWOG 0127.J Clin Oncol 2006;24:4922-7.

29. Tew WP, Shah M, Schwartz G, et al. Phase II trial of erlotinib for second-line treatment in advanced esophageal cancer. Gastrointestinal Cancer Symposium. Proc Am Soc Clin Oncol 2005. Abstr 5.

30. Wu X, Fan Z, Masui H, et al. Apoptosis induced by an anti-epidermal growth factor receptor monoclonal antibody in a human colorectal carcinoma cell line and its delay by insulin. J Clin Invest 1995;95:1897-1905.

31. Bonner JA, Harari PM, Giralt J, et al. Radiotherapy plus cetuximab for squamous-cell carcinoma for the head and neck. N Engl J Med 2006;354:567-78.

32. Raben D, Helfrich B, Bunn PA. Targeted therapies for non-small-cell lung cancer: biology, rationale, and preclinical results from a radiation oncology perspective. Int J Radiat Oncol Biol Phys 2004;59(2 Suppl):27-38.

33. Langer CJ. Emerging role of epidermal growth factor receptor inhibition in therapy for advanced malignancy: focus on NSCLC. Int J Radiat Oncol Biol Phys 2004;58:991-1002.

34. Pfister DG, Aliff TB, Kraus DH, et al. Concurrent cetuximab, cisplatin, and concomitant boost radiation therapy (RT) for locoregionally advanced, squamous cell head and neck cancer (SCCHN): preliminary evaluation of a new combined-modality paradigm. Proc Am Soc Clin Oncol 2003. Abstr 1993

35. Suntharalingam M, Dipetrilo T, Akerman P, et al. Cetuximab, paclitaxel, carboplatin and radiation for esophageal and gastric cancer. Proc Am Soc Clin Oncol 2006. Abstr 4029.

36. Meluch AA, Greco FA, Gray JR, et al. Preoperative therapy with concurrent paclitaxel/carboplatin/infusional 5-FU and radiation therapy in locoregional esophageal cancer: final results of a Minnie Pearl Cancer Research Network phase II trial. Cancer J 2003;9:251-60.

37. White RE, Abnet CC, Mungatana CK, et al. Oesophagal cancer: a common malignancy in young people of Bomet District Kenya. Lancet 2002;360:462-3.

38. Tepper JE, Krasna M, Niedzwiecki D, et al. Superiority of trimodality therapy to surgery alone in esophageal cancer: results of CALGB 9781. Proc Am Soc Clin Oncol 2006. Abstr 4012

39. Burris H, Moore M, Andersen J, et al. Improvements in survival and clinical benefit with gemcitabine as first-line therapy for patients with advanced pancreas cancer: a randomized trial. J Clin Oncol 1997;15:2403-13.

40. Outcomes of cancer treatment for technology assessment and cancer treatment guidelines. American Society of Clinical Oncology. J Clin Oncol 1996;14:671-9.

41. Kachnic LA, Scott C, Wasserman T, et al. One year follow-up reveals no difference in quality of life between high dose and conventional dose radiation: a quality of life assessment of RTOG 94-05. American Society for Therapeutic Radiology and Oncology 43rd Annual Meeting, San Francisco, CA, November, 2001.

42. Cella DF. Manual for the Functional Assessment of Cancer Therapy (FACT) Scales version 2, April 1993.

43. Brooks J, Kesler K, Johnson C, et al. Prospective analysis of quality of life after surgical resection for esophageal cancer: preliminary analysis. J Surg Oncol 2002;81:185-94.

44. Darling G, Eton DT, Sulmann J, et al. Validation of the functional assessment of cancer therapy esophageal cancer subscale (FACT-E). Cancer 2006;107:854-63.

45. Cella DF, Tulsky DS, Gray G, et al. The functional assessment of cancer therapy scale: development and validation of general measure. J Clin Oncol 1993;11:570-9.

46. Overcash J, Extermann M, Parr J, et al. Validity and reliability of the FACT-G subscale use in older person with cancer. Am J Clin Oncol 2001;24:591-6.

47. Suntharalingam M, Dipetrillo T, Wanebo H, et al. Weekly cetuximab, paclitaxel, CBDCA and daily RT for patients with locally advanced esophageal cancer. ASTRO proceedings, 2006.

48. Schulz MW, Chen J, Woo HH, et al. A comparison of techniques for eliciting patient preference in patients with benign prostatic hyperplasia. J Urol 2002;168:155-9.

49. Wu AW, Jacobson KD, Frick DL, et al. Validity and responsiveness of the EQ-5D as a measure of health-related quality of life in people enrolled in an AIDS clinical trial. Qual Life Res 2002;11:273-82.

50. Williams J, Russell I, Durai D, et al. What are the clinical outcome and cost-effectiveness of endoscopy undertaken by nurses when compared with doctors? A Multi-Institution Nurse Endoscopy Trial (MINuET) Health Technol Assess 2006;10(40):iii-iv,ix-x,1-195.

51. Jones R, Horbach S, Sander P, et al. Heartburn in patients with gastro-oesophageal reflux disease in Germany and Sweden: a study on patients' burden of disease. Pharmacoeconomics 2003;15:1091-102.

52. Glasziou PP, Simes RJ, Gelber RD. Quality Adjusted Survival Analysis. Stat Med 1990;9:1256-76.

53. Siewert JR, Feith M. Stein HJ. Adenocarcinoma of the esophagogastric junction results of surgical therapy based on anatomic/topographic classification in 1002 consecutive patients. Annals of Surgery 2000;232:353-61.

54. Zelen M. The randomization and stratification of patients to clinical trials. J Chron Dis 1974;27:365-75.

55. Kelsen D, Ilson D, Lipton R, et al. A phase I trial of radiation therapy (RT) plus concurrent fixed dose cisplatin (C) with escalating doses of paclitaxel (P) as a continuous infusion in patients (pts) with localized esophageal cancer (EC). Proc ASCO 1999;18:271a. Abstr 1039.

56. Enzinger P, Ilson D, Minsky B, et al. Phase I/II neoadjuvant concurrent 96 hour taxol, cisplatin and radiation therapy; Promising toxicity profile and response in localized esophageal cancer. Proc ASCO 1999; 18:271a. Abstr 1038.

57. Kaplan EL, Meier P. Nonparametric estimation from incomplete observations. J Stat Assoc 1958;53:457-81.

58. Mantel N. Evaluation of survival data and two new rank order statistics arising in its consideration. Cancer Chemother. 1966;Rep 50:163-70.

59. Fleming T. One-sample multiple testing procedure for phase II clinical trials. Biometrics 1982;38:143-51.

60. Lan KKG, DeMets ER. Discrete sequential boundaries for clinical trials. Biometrika 1983;70:659-63.

61. O'Brien PC, Fleming TR. A multiple testing procedure for clinical trials. Biometrics 1979;35:549-5.

62. Freidlin B, Korn EL. A comment on futility monitoring. Controlled Clinical Trials 2002;23:355-66.

63. Donaldson GW, Moinpour CM. Learning to live with missing quality of life data in advanced-stage disease trials. J Clin Oncol 2005;23:1-5.

APPENDIX I

INFORMED CONSENT TEMPLATE FOR CANCER TREATMENT TRIALS
(ENGLISH LANGUAGE)

RTOG 0436

A Phase III Trial Evaluating the Addition of Cetuximab to Paclitaxel, Cisplatin, and Radiation for Patients With Esophageal Cancer Who Are Treated Without Surgery

This is a clinical trial, a type of research study. Your study doctor will explain the clinical trial to you. Clinical trials include only people who choose to take part. Please take your time to make your decision about taking part. You may discuss your decision with your friends and family. You can also discuss it with your health care team. If you have any questions, you can ask your study doctor for more explanation.

You are being asked to take part in this study because you have esophageal cancer that is considered appropriate for treatment with a combination of chemotherapy and radiation. Based on the fact that your cancer started in the esophagus and you have not yet received any treatment for your cancer, you may be eligible for participation.

Why is this study being done?

In this study, you will get either radiation, chemotherapy, and cetuximab or radiation and chemotherapy. The purpose of this study is to compare the effects, good and/or bad, of radiation therapy and chemotherapy (paclitaxel and cisplatin) with or without the addition of cetuximab to find out which treatment is better.

Cetuximab may delay or prevent tumor growth by blocking certain cellular chemical pathways that lead to tumor development. Cetuximab is approved for the treatment of colorectal and head and neck cancers but is experimental for esophageal cancer. Cetuximab is investigational in this study.

How many people will take part in the study?

About 420 people will take part in this study.

What will happen if I take part in this research study? (2/13/09)

Before you begin the study …

You will need to have the following exams, tests or procedures to find out if you can be in the study. These exams, tests or procedures are part of regular cancer care and may be done even if you do not join the study. If you have had some of them recently, they may not need to be repeated.

- History and physical exam
- One of the following:
  1. Chest/abdominal CT (computed tomography) scan (a CT scan is a study that uses x-rays to look inside of your body) OR
  2. A PET (positron emission tomography) scan [A PET scan is a computerized image that looks at the activity of tumor cells in your entire body and that requires injection of a special marker into your vein, such as sugar (glucose) combined with a low-dose radioactive substance (a tracer). A camera records the tracer's signal as it travels through your body.] OR
  3. A combination of a PET and CT scan of your body (a PET-CT scan)

- EKG (electrocardiogram) (an EKG is a test of your heart function)
- Endoscopy with biopsy or fine needle aspiration. During an endoscopy, your study doctor will insert a tube into your throat that will allow him/her to look at your esophagus. Your study doctor will remove some of the cancerous tissue (biopsy) or extract cells (fine needle aspiration) during this procedure.
- Blood tests (about 2-3 teaspoons of blood will be taken from your vein)
  - This will include a blood pregnancy test if you are a woman of child-bearing potential
- Assessment of your daily caloric intake to be sure you are able to eat enough to provide enough energy for your body
- If you agree, blood and urine tests will be collected for research purposes. This will be discussed later in this consent form.

You will also be asked to report any use of over-the-counter (OTC) or herbal products to your study doctor, so that he or she can make sure you are not taking any products that interact with any of the study drugs. You cannot be in this study if you are having frequent chest pains ("angina"), have had frequent chest pains, or have been hospitalized for heart failure within the last 3 months. You cannot be in this study if you had a heart attack in the past 6 months. Tell your study doctor if you think any of these may apply to you.

During the study … (8/12/08)

If the exams, tests and procedures show that you can be in the study, and you choose to take part, then you will need the following tests and procedures. They are part of regular cancer care. They will be done every week while you are receiving treatment.

- History and physical exam
- Evaluation of your ability to carry out daily activities
- Blood tests (about 2-3 teaspoons of blood will be taken from your vein)
- Evaluation of any side effects you may be experiencing

You will be "randomized" into one of the study groups described below. Randomization means that you are put into a group by chance. A computer program will place you in one of the study groups. Neither you nor your study doctor can choose the group you will be in. You will have an equal chance of being placed in either group.

All patients will receive radiation. Radiation treatment will be given once a day, 5 days a week, for 5 and a half weeks. All radiation treatments will be given as an outpatient at your institution. In general, each treatment will last about 30 minutes.

All patients will also receive chemotherapy with paclitaxel and cisplatin. Starting the same day as your radiation treatments, you will receive paclitaxel and cisplatin once a week for 6 weeks (6 cycles). Paclitaxel and cisplatin will be injected into a vein (intravenously). You will be given intravenous fluids and medicines to prevent nausea. You will also be given diphenhydramine, ranitidine, and dexamethasone to prevent an allergic reaction. Your chemotherapy treatments will be given as an outpatient at your institution. The treatment will last for about 3 and a half hours, once a week.

If you are in group 1 (often called "Arm 1") … You will also receive the drug cetuximab. Starting the same day as your first dose of radiation and paclitaxel plus cisplatin, you will be given cetuximab by vein as an outpatient. Then you will receive paclitaxel and cisplatin. You will get cetuximab, paclitaxel, and cisplatin once a week for 6 weeks with the radiation. If you are randomized to this arm, you should wear sunscreen and a hat to limit sun exposure, because cetuximab causes skin problems and sun exposure can makes these problems worse.

If you are in group 2 (often called "Arm 2")… You will not receive cetuximab. You will be treated with radiation and paclitaxel plus cisplatin.

When you are finished receiving treatment on this study…(9/13/2011)

You will need to have the following exams, tests, and procedures. They are being done to see how the treatment you received affected you and your cancer.

- At the end of treatment
  - Blood tests (about 2-3 teaspoons of blood will be taken from your vein)
  - History and physical exam

- At 6-8 weeks after you've finished treatment
  - Endoscopy: If it looks like your cancer is still present, your study doctor will also do a biopsy to confirm this. Your study doctor may also do a biopsy even if it doesn't look like your cancer is still present in order to find out how well your cancer has responded to the study treatment.
  - PET/PET-CT scan or chest/abdominal CT scan
  - Surgery/Additional Treatment: If it looks like your cancer is still present but has not grown outside of your esophagus, your doctor *may* talk to you about removing your esophagus in order to prevent the tumor from possibly growing. In addition, if it looks like your cancer is still present, you may need to switch to a different treatment, such as another chemotherapy regimen.
  - If you agree, blood and urine tests will be collected for research purposes. This will be discussed later in this consent form.

- During follow up
  - History and physical exam: every 4 months from the start of your treatment for 2 years, then every 6 months for 2 more years, then every year
  - If your cancer goes away a PET/PET-CT scan or chest/abdominal CT scan i beginning at 8 months from the start of your treatment, and then every 4 months for 2 years, then every 6 months for 2 more years, then every year,. If your cancer goes away and then appears to have returned, your doctor may talk to you about an additional biopsy.

How long will I be in the study? (8/12/08)

You will receive treatment with radiation and drug therapy for approximately 6 weeks. After you are finished the treatment, your study doctor will ask you to visit the office for follow-up exams every 4 months for 2 years, every 6 months for 2 years, and then every year indefinitely.

Can I stop being in the study?

Yes. You can decide to stop at any time. Tell the study doctor if you are thinking about stopping or decide to stop. He or she will tell you how to stop safely.

It is important to tell the study doctor if you are thinking about stopping so he/she can evaluate any risks from the treatment. Another reason to tell your study doctor that you are thinking about stopping is to discuss what follow-up care and testing could be most helpful for you.

The study doctor may stop you from taking part in this study at any time if he/she believes it is in your best interest, if you do not follow the study rules, or if the study is stopped.

What side effects or risks can I expect from being in the study?

You may have side effects while on the study. Everyone taking part in the study will be watched carefully for any side effects. However, researchers don't know all the side effects that may happen. Side effects may be mild or very serious. Your health care team may give you medicines to help lessen side effects. Many side effects go away soon after you stop taking the treatment. In some cases, side effects can be serious, long lasting, or may never go away. There also is a risk of death. In addition, side effects of chemotherapy may be increased when it is given with radiation.

(8/19/10) As of September 21, 2010, patients 75 years or older are not allowed to participate in this study. This decision was based on the Study Chairs' review of toxicity data for the patients enrolled to the study. Their review revealed that patients 75 or older experienced more severe side effects than patients younger than 75. The Study Chairs' decision to exclude patients 75 years or older is to ensure adequate safety for all patients participating in this study.

You should talk to your study doctor about any side effects that you have while taking part in the study.

Risks and side effects related to the radiation include those that are:

Likely

- Fatigue
- Decrease in blood counts, which can cause infection, bleeding, and bruising
- Tanning and redness of the skin in the treatment area

Less Likely

- Growth of fibrous tissues similar to scar tissue underneath your skin
- Nausea/vomiting
- Diarrhea
- Weight loss

Rare but Serious

- Inflammation of the muscle tissue of the heart
- Inflammation and/or scarring of the lung tissue
- Inflammation of the esophagus
- Inflammation of the spinal cord
- Narrowing of the esophagus, which can cause problems with swallowing
- Hole in the esophagus

Risks and side effects related to paclitaxel include those that are:

Likely

- Fatigue
- Hair loss
- Nausea
- Vomiting
- Diarrhea
- Abdominal pain
- Decrease in blood counts, which can cause infection, (white blood cell count) or bleeding or bruising (platelet count)
- Anemia (decrease in red blood cell count)
- Tanning and redness of the skin in the treatment area
- Hardening or tenderness of the skin in the treatment area

Less Likely

- Mouth sores
- Tingling or numbness in your hands and feet
- Stiffness or pain in your joints and muscles
- Ulceration of the skin at the injection site, including redness, tenderness, swelling, and cellulitis (infection of the skin)
- Inflammation of the liver with a rise in liver function tests
- Tearing of the eyes
- Inflammation of the lining of the eye
- Low blood pressure

- Slowing of the heart rate
- Premature heart beats
- Changes in skin or nail color
- Fragility of nails
- Swelling of the legs or ankles
- Constipation

Rare but Serious

- Problems with your heart, including irregular or rapid heart beat, high blood pressure, and fainting
- Heart attack
- Congestive heart failure (heart muscle weakness, swelling, and shortness of breath)
- Blood clots in the veins
- Severe allergic reaction with low blood pressure, shortness of breath, rash, swelling of the face, redness in the face, chest pain, and shock
- Death from allergic reaction
- Death from infection due to low white blood cell count, including sepsis (blood infection), peritonitis (infection of the stomach lining), and pneumonia
- Visual changes including flashes of light
- Hearing loss
- Muscle weakness
- Liver failure
- Intestinal obstruction
- Intestinal perforation
- Pancreatitis (inflammation of the pancreas)
- Ischemic colitis (impaired blood flow to the bowel)
- Lung inflammation or fibrosis (hardening of tissue)
- Pulmonary embolism (blood clot in lung)
- Seizure
- Balance or coordination difficulty

Risks and side effects related to cisplatin include those that are:
Likely

- Decrease in blood counts, which can cause infection, (white blood cell count) or bleeding or bruising (platelet count)
- Anemia (decrease in red blood cell count)
- Nausea
- Vomiting
- Diarrhea
- Loss of appetite and taste
- Fatigue
- Weight loss
- Hair loss
- Changes in body calcium, potassium, sodium, phosphate, and magnesium levels, which can cause muscle cramps, weakness, and abnormal heart rhythms

Less Likely

- Mouth sores
- Restlessness
- Tingling or numbness in your hands and feet
- Muscle cramps
- Weakness
- Hiccups
- Increase in blood uric acid level
- Inflammation of the liver resulting in rise in liver function tests

- Blurred vision

<u>Rare but Serious</u>

- Leukemia (another type of cancer that is likely to be fatal)
- Involuntary movements, loss of coordination, and seizures
- Severe allergic reaction with low blood pressure, shortness of breath, rash, swelling of the face, chest pain, and shock
- Damage to the ears, including hearing loss and ringing in the ears
- Kidney failure
- Death from allergic reaction
- Death from infection due to low white blood cell count
- Heart attack
- Stroke
- Irregular heart beat
- Blindness

Risks and side effects related to cetuximab include those that are:

Likely:

- Diarrhea
- Nausea or the urge to vomit
- Fatigue or tiredness
- Fever
- Headache or head pain
- Dry skin
- Acne
- Skin rash with the presence of flat discolored areas (macules) and raised bumps (papules)

Less Likely:

- Lack of enough red blood cells (anemia)
- Swelling and redness (inflammation) of the skin of outer ear and canal
- Noise in the ears, such as ringing, buzzing, roaring, clicking
- Swelling and redness (inflammation) of the outermost layer of the eye and the inner surface of the eyelids (conjunctiva); commonly called "pink eye".
- Dry eye
- Swelling and redness (inflammation) of the middle layer of the eye (uvea)
- Excessive tearing in the eyes
- Belly pain
- Swelling and redness (inflammation) of the lip
- Constipation
- Dry mouth
- Heartburn
- Irritation or sores in the lining of the mouth
- Vomiting
- Chills
- Swelling of the arms and/or legs
- Flu-type symptoms (including body aches, fever, chills, tiredness, loss of appetite, cough)
- Reaction that can occur during or following infusion of the drug. The reaction may include fever, chills, rash, low blood pressure, and difficulty breathing.
- Chest pain not heart-related
- Allergic reaction by your body to the drug product that can occur immediately or may be delayed. The reaction may include hives, low blood pressure, wheezing, swelling of the throat, and difficulty breathing. Your condition will be closely monitored during doses of cetuximab and for at least one hour afterwards. If

you have a severe reaction, your doctor will treat you for the reaction, and you will not receive further treatment on this study. If you have a delayed severe reaction after receiving cetuximab, you must immediately tell your doctor.

- Infection
- Decreased number of a type of white blood cell (neutrophil/granulocyte)
- Weight loss
- Decrease in the total number of white blood cells (leukocytes)
- Loss of appetite
- Dehydration (when your body does not have as much water and fluid as it should)
- Decreased blood level of calcium
- Decreased blood level of magnesium
- Joint pain
- Back pain
- Muscle pain
- Fainting
- Stuffy or runny nose, sneezing
- Sudden constriction of the small airways of the lung that can cause wheezing and shortness of breath
- Cough
- Shortness of breath
- Hoarseness
- Hair loss
- Loss of some or all of the finger or toenails
- Increased skin sensitivity to sunlight
- Itching
- Area of bleeding within the skin causing a reddish purple discoloration
- Sores or destruction of skin
- Hives
- Low blood pressure
- Formation of a blood clot that plugs the blood vessel; blood clots may break loose and travel to another place, such as the lung

Rare but Serious:

- Serious, life-threatening allergic reaction requiring immediate medical treatment by your doctor. The reaction may include extremely low blood pressure, swelling of the throat, difficulty breathing, and loss of consciousness. Your condition will be closely monitored during doses of cetuximab and for at least one hour afterwards. If you have a severe reaction, your doctor will treat you for the reaction, and you will not receive further treatment on this study. If you have a delayed severe reaction after receiving cetuximab, you must immediately tell your doctor.
- Inflammation of the lining of the brain and spinal cord
- Inflammation of the lungs that may cause difficulty breathing and can be life-threatening
- Fluid build-up in the lungs that is not due to a heart problem and that can be life-threatening
- Swelling and redness of the skin on the palms of the hands and soles of the feet

Reproductive risks: The drugs in this study can affect an unborn baby. You should therefore not become pregnant or father a baby while on this study and, if you are receiving cetuximab, for at least 60 days after the last cetuximab dose. Women should not breastfeed a baby while on this study and, if you are receiving cetuximab, for at least 60 days after the last cetuximab dose. It is important you understand that you need to use birth control while on this study. Check with your study doctor about what kind of birth control methods to use and how long to use them. Some methods might not be approved for use in this study.

For more information about risks and side effects, ask your study doctor.

Are there benefits to taking part in the study?

Taking part in this study may or may not make your health better. While researchers hope radiation therapy, chemotherapy, and cetuximab will be more useful against cancer compared to the usual treatment, there is no

proof of this yet. We do know that the information from this study will help researchers learn more about this therapy combination as a treatment for cancer. This information could help future cancer patients.

What other choices do I have if I do not take part in this study?

Your other choices may include:

- Getting treatment or care for your cancer without being in a study. This may include surgery in some cases, either by itself or with chemotherapy and/or radiation. You could also receive chemotherapy and radiation with the same or different chemotherapy drugs as used in this study without being part of this study.
- Taking part in another study
- Getting no treatment
- Getting comfort care, also called palliative care. This type of care helps reduce pain, tiredness, appetite problems and other problems caused by the cancer. It does not treat the cancer directly, but instead tries to improve how you feel. Comfort care tries to keep you as active and comfortable as possible.

Talk to your study doctor about your choices before you decide if you will take part in this study.

Will my medical information be kept private? (9/13/2011)

Data are housed at RTOG Headquarters in a password-protected database. We will do our best to make sure that the personal information in your medical record will be kept private. However, we cannot guarantee total privacy. Your personal information may be given out if required by law. If information from this study is published or presented at scientific meetings, your name and other personal information will not be used.

Organizations that may look at and/or copy your medical records for research, quality assurance, and data analysis include:

- The Radiation Therapy Oncology Group (RTOG)
- The National Cancer Institute (NCI) and other government agencies, like the Food and Drug Administration (FDA), involved in keeping research safe for people
- Qualified representatives of ImClone and Bristol-Myers Squibb, manufacturers and distributors of cetuximab
- The Cancer Trials Support Unit (CTSU), a research group sponsored by the National Cancer Institute (NCI) to provide patients and doctors greater access to cancer trials

A description of this clinical trial will be available on http://www.ClinicalTrials.gov, as required by U.S. Law. This Web site will not include information that can identify you. At most, the Web site will include a summary of the results. You can search this Web site at any time.

What are the costs of taking part in this study?

You and/or your health plan/ insurance company will need to pay for some or all of the costs of treating your cancer in this study. Some health plans will not pay these costs for people taking part in studies. Check with your health plan or insurance company to find out what they will pay for. Taking part in this study may or may not cost your insurance company more than the cost of getting regular cancer treatment.

Bristol-Myers Squibb is supplying cetuximab at no cost to you. However, you or your health plan may need to pay for costs of the supplies for drug administration and personnel who give you the cetuximab.

You will not be paid for taking part in this study.

For more information on clinical trials and insurance coverage, you can visit the National Cancer Institute's Web site at http://www.cancer.gov/clinicaltrials/payingfor/insurance-coverage

You can print a copy of the “Clinical Trials and Insurance Coverage” information from this Web site.

Another way to get the information is to call 1-800-4-CANCER (1-800-422-6237) and ask them to send you a free copy.

What happens if I am injured because I took part in this study?

It is important that you tell your study doctor, _________________ *[investigator’s name(s)],* if you feel that you have been injured because of taking part in this study. You can tell the study doctor in person or call him/her at _________________ *[telephone number].*

You will get medical treatment if you are injured as a result of taking part in this study. You and/or your health plan will be charged for this treatment. The study will not pay for medical treatment.

What are my rights if I take part in this study?

Taking part in this study is your choice. You may choose either to take part or not to take part in the study. If you decide to take part in this study, you may leave the study at any time. No matter what decision you make, there will be no penalty to you and you will not lose any of your regular benefits. Leaving the study will not affect your medical care. You can still get your medical care from our institution.

We will tell you about new information or changes in the study that may affect your health or your willingness to continue in the study.

A Data Safety Monitoring Board will be regularly meeting to monitor safety and other data related to this study. The Board members may receive confidential patient information, but they will not receive your name or other information that would allow them to identify you by name.

In the case of injury resulting from this study, you do not lose any of your legal rights to seek payment by signing this form.

Who can answer my questions about the study?

You can talk to your study doctor about any questions or concerns you have about this study. Contact your study doctor _________________ *[name(s)]* at _________________ *[telephone number].*

For questions about your rights while taking part in this study, call the _______________________ *[name of center]* Institutional Review Board (a group of people who review the research to protect your rights) at _________________ *(telephone number). [Note to Local Investigator: Contact information for patient representatives or other individuals in a local institution who are not on the IRB or research team but take calls regarding clinical trial questions can be listed here.]*

*You may also call the Operations Office of the NCI Central Institutional Review Board (CIRB) at 888-657-3711 (from the continental US only). *[*Only applies to sites using the CIRB.]*

Please note: This section of the informed consent form is about additional research that is being done with people who are taking part in the main study. You may take part in this additional research if you want to. You can still be a part of the main study even if you say ‘no’ to taking part in this additional research.

You can say “yes” or “no” to the following studies. Below, please mark your choice for each question.

Quality of Life Study

We want to know your view of how your life has been affected by cancer and its treatment. This “Quality of Life” study looks at how you are feeling physically and emotionally during your cancer treatment. It also looks at how you are able to carry out your day-to-day activities.

This information will help doctors better understand how patients feel during treatments and what effects the medicines are having. In the future, this information may help patients and doctors as they decide which medicines to use to treat cancer.

You will be asked to complete 2 questionnaires on 4 occasions.

You will complete the questionnaires before you begin treatment, within 1 week after you having your post-treatment endoscopy, 1 year after you start treatment, and 2 years after you start treatment. It takes about 10 minutes to fill out each of the questionnaires.

If any questions make you feel uncomfortable for any reason, skip those questions.

If you decide to take part in this study, the only thing you will be asked to do is fill out the questionnaires. You may change your mind about participating at any time.

Just like in the main study, we will do our best to make sure that your personal information will be kept private.

Please circle your answer.

I choose to take part in the quality of life study. I agree to fill out the 2 quality of life questionnaires.

YES **NO**

Use of Tissue, Blood, and Urine for Research

About Using Tissue, Blood, and Urine for Research (9/13/2011)

You have had a biopsy (or surgery) to see if you have cancer. Your doctor removed some body tissue to do some tests. The results of these tests will be given to you by your doctor and will be used to plan your care.

We would like to keep some of the tissue that was left over for future research. If you agree, this tissue will be kept and may be used in research to learn more about cancer and other diseases. Please read the information sheet called "How is Tissue Used for Research" to learn more about tissue research. This information sheet is available to all at the following web site: http://cdp.cancer.gov/humanSpecimens/ethical_collection/patient.htm

As a result of your participation in the trial, you also will have blood tests performed before you start treatment and 6-8 weeks after you have finished treatment. We would like to keep for future research about three teaspoons of the blood taken at each of these times. If you agree, this blood will be kept and may be used in research to learn more about cancer and other diseases.

In addition, we would like to keep some of your urine for future research. We would collect your urine at the following times: before you start treatment and 6-8 weeks after you have finished treatment. If you agree, the urine will be kept and may be used in research to learn more about cancer and other diseases.

The research that may be done with your tissue, blood, and urine is not designed specifically to help you. It might help people who have cancer and other diseases in the future.

Reports about research done with your tissue, blood, and urine will not be given to you or your doctor. These reports will not be put in your health record. The research will not have an effect on your care.

Things to Think About

The choice to let us keep your tissue, blood, and urine for future research is up to you. No matter what you decide to do, it will not affect your care or your participation in the main part of the study.

If you decide now that your tissue, blood, and urine can be kept for research, you can change your mind at any time. Just contact us and let us know that you do not want us to use your tissue, blood, and urine. Then any material that remains will no longer be used for research. Any tissue that remains will be returned to the institution that submitted it and any blood or urine that remains will be destroyed.

In the future, people who do research may need to know more about your health. While the doctor/institution may give them reports about your health, it will not give them your name, address, phone number, or any other information that will let the researchers know who you are.

Sometimes tissue, blood, and urine are used for genetic research (about diseases that are passed on in families). Even if your tissue, blood, and urine are used for this kind of research, the results will not be put in your health records.

Your tissue, blood, and urine will be used only for research and will not be sold. The research done with your tissue, blood, and urine may help to develop new products in the future.

Benefits

The benefits of research using tissue, blood, and urine include learning more about what causes cancer and other diseases, how to prevent them, and how to treat them.

Risks

The greatest risk to you is the release of information from your health records. We will do our best to make sure that your personal information will be kept private. The chance that this information will be given to someone else is very small.

Making Your Choice

Please read each sentence below and think about your choice. After reading each sentence, circle "Yes" or "No". If you have any questions, please talk to your doctor or nurse, or call our research review board at IRB's phone number.

No matter what you decide to do, it will not affect your care.

1. My tissue, blood, and urine may be kept for use in research to learn about, prevent, or treat cancer.

Yes No

2. My tissue, blood, and urine may be kept for use in research to learn about, prevent or treat other health problems (for example: diabetes, Alzheimer's disease, or heart disease).

Yes No

3. Someone may contact me in the future to ask me to take part in more research.

Yes No

Where can I get more information? (9/13/2011)

You may call the National Cancer Institute's Cancer Information Service at:

1-800-4-CANCER (1-800-422-6237)

You may also visit the NCI Web site at http://cancer.gov/

- **For NCI's clinical trials information, go to: http://cancer.gov/clinicaltrials/**
- **For NCI's general information about cancer, go to http://cancer.gov/cancerinfo/**

You will get a copy of this form. If you want more information about this study, ask your study doctor.

Signature

I have been given a copy of all _____ *[insert total of number of pages]* **pages of this form. I have read it or it has been read to me. I understand the information and have had my questions answered. I agree to take part in this study.**

Participant ________________________________

Date ______________________________________

APPENDIX II: STUDY PARAMETER TABLE (8/12/08, 2/13/09, 9/9/09, 6/22/10, 8/19/10)

	**Pre-Treatment**			**During Treatment**			**Follow Up**	
	≤ 12 weeks prior to registration	≤ 42 days prior to registration	≤ 14 days prior to registration	Weekly during chemo/RT	At chemo/RT completion	At 6-8 weeks post-treatment	Every 4 months from chemo/RT start for 2 years, every 6 months for 2 years, then annually	At 1 and 2 years from treatment start
Histologically proven diagnosis by endoscopy w/ biopsy or cytology by FNA (See section 3.1.2.4 of the protocol)	X							
History/physical w/ weight		X		X	X		X	
PET/PET-CT/Chest/abdominal CT (PET/PET-CT strongly recommended)		X				X	For patients with a clinical complete response at post-treatment endoscopy to start with the 2nd follow up	
EKG		X						
Endoscopy*/biopsy	X					Repeat endoscopy for all patients; biopsy if persistence of tumor is present or suspected		
Performance status		X		X	X		X	
CBC w/ diff, ANC, platelets, Hgb			X	X	X			
Creatinine			X	X	X			
Bilirubin			X		X			
AST			X		X			
Magnesium			X	X	X			
Calcium			X	X	X			
Potassium			X	X	X			
Serum pregnancy test (if applicable)			X					
Caloric Assessment		X						
Informed consent		X						
Adverse event eval				X	X		X	
Tissue/blood/urine for banking (for participating patients)		≤ 42 days prior to treatment				X		
Quality of Life/Health Utility Assessments (for participating patients) ▪ FACT-E ▪ EQ-5D			X			X		X

*See Section 11.2 for exceptions and details

APPENDIX III (2/13/09)

ZUBROD PERFORMANCE SCALE

**0**	**Fully active, able to carry on all predisease activities without restriction.**
**1**	**Restricted in physically strenuous activity but ambulatory and able to carry work of a light or sedentary nature. For example, light housework, office work.**
**2**	**Ambulatory and capable of all self-care but unable to carry out any work activities. Up and about more than 50% of waking hours.**
**3**	**Capable of only limited self-care, confined to bed or chair 50% or more of waking hours.**
**4**	**Completely disabled. Cannot carry on self-care. Totally confined to bed.**
**5**	**Death.**

APPENDIX IV

AJCC STAGING SYSTEM, 6th Edition
ESOPHAGUS

DEFINITION OF TNM

Primary Tumor (T)

TX Primary tumor cannot be assessed
T0 No evidence of primary tumor
Tis Carcinoma in situ
T1 Tumor invades lamina propria or submucosa
T2 Tumor invades muscularis propria
T3 Tumor invades adventitia
T4 Tumor invades adjacent structures

Regional Lymph Nodes (N)

NX Regional lymph nodes cannot be assessed
N0 No regional lymph node metastasis
N1 Regional lymph node metastasis

Distant Metastasis (M)

MX Presence of distant metastasis cannot be assessed
M0 No distant metastasis
M1 Distant metastasis

Tumors of lower thoracic esophagus
M1a Metastasis in celiac lymph nodes
M1b Other distant metastasis

Tumors of mid-thoracic esophagus
M1a Not applicable
M1b Non-regional lymph nodes and/or other distant metastasis

Tumors of upper thoracic esophagus
M1a Metastasis in cervical nodes
M1b Other distant metastasis

STAGE GROUPING

Stage 0	Tis	N0	M0
Stage I	T1	N0	M0
Stage IIA	T2	N0	M0
	T3	N0	M0
Stage IIB	T1	N1	M0
	T2	N1	M0
Stage III	T3	N1	M0
	T4	Any N	M0
Stage IV	Any T	Any N	M1
Stage IVA	Any T	Any N	M1a
Stage IVB	Any T	Any N	M1b

APPENDIX V (5/3/12)

Appendices for RTOG Biospecimen Collection

RTOG FFPE Specimen Plug Kit Collection
RTOG Blood Collection Kit Instructions
RTOG Urine Collection Kit Instructions

Shipping Instructions:

U.S. Postal Service Mailing Address: For FFPE or Non-frozen Specimens Only
RTOG Biospecimen Resource
University of California San Francisco
Campus Box 1800
2340 Sutter St, Room S341
San Francisco, CA 94143-1800

Courier Address (FedEx, UPS, etc.): For Frozen or Trackable Specimens
RTOG Biospecimen Resource
University of California San Francisco
2340 Sutter St, Room S341
San Francisco, CA 94143-1800

- Include all RTOG paperwork in pocket of biohazard bag.
- Check that the Specimen Transmittal Form (STF) has the consent boxes checked off.
- Check that all samples are labeled with the RTOG study and case number, and include date of collection as well as collection time point (e.g., pretreatment, post-treatment).

- **FFPE Specimens:**
  - o Slides should be shipped in a plastic slide holder/slide box. Place a small wad of padding in top of the container. If you can hear the slides shaking it is likely that they will break during shipping.
  - o FFPE Blocks can be wrapped with paper towel, or placed in a cardboard box with padding. Do not wrap blocks with bubble wrap. Place padding in top of container so that if you shake the container the blocks are not shaking. If you can hear the slides shaking it is likely that they will break during shipping.
  - o Slides, Blocks, or Plugs can be shipped ambient or with a cold pack either by United States Postal Service (USPS) to the USPS address (94143) or by Courier to the Street Address (94115). **Do NOT ship on Dry Ice**.

- **Frozen Specimens:**
  - o Multiple cases may be shipped in the same cooler, but make sure each one is in a separate bag and clearly identified.
  - o Place specimens and absorbent shipping material in Styrofoam cooler filled with dry ice (at least 7 lbs). There should be plenty of dry ice under and above the specimens. If the volume of specimens is greater than the volume of dry ice then ship in a larger Styrofoam box, or two separate boxes. Any Styrofoam box can be used, as long as it is big enough.
  - o Specimens received thawed due to insufficient dry ice or shipping delays will be discarded and the site will be notified.
  - o Send frozen specimens via overnight courier to the address above. Specimens should only be shipped Monday through Wednesday (Monday-Tuesday for Canada) to prevent thawing due to delivery delays. Saturday or holiday deliveries cannot be accepted. Samples can be stored frozen at -80° C until ready to ship.

- **For Questions regarding collection/shipping please contact the RTOG Biospecimen Resource by e-mail: RTOG@ucsf.edu or phone: 415-476-RTOG(7864) or Fax: 415-476-5271.**

APPENDIX V (cont’d)

RTOG FFPE SPECIMEN PLUG KIT INSTRUCTIONS

This Kit allows sub-sampling of an FFPE block for submission to the RTOG Biospecimen Resource. The plug kit contains a shipping tube and a punch tool.

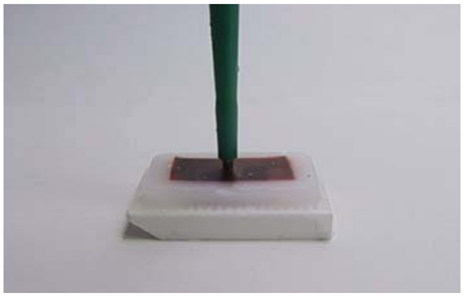

Step 1
If the block is stored cold, allow it to equilibrate for 30 minutes at room temperature. Place the punch tool on the paraffin block over the selected tumor area. (Ask a pathologist to select area with tumor.) Push the punch into the paraffin block. Twist the punch tool once around to separate the plug from the block. Then pull the punch tool out of the block. The punch should be filled with tissue sample.

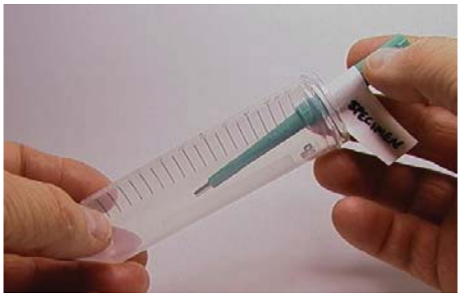

Step 2
Label the punch tool with the proper specimen ID. DON’T remove specimen from the punch.

Use a separate punch tool for every specimen. Call or e-mail us if you have any questions or need additional specimen plug kits.

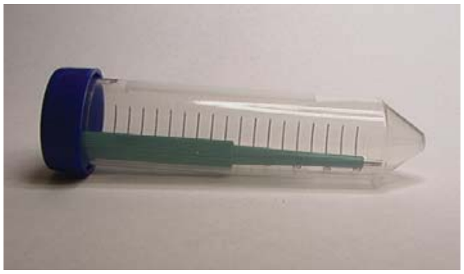

Step 3
Once punch tool is labeled, place in shipping tube and mail to address below. Please do not mix specimens in the same tube.

We will remove core specimen from the punch, embed in a paraffin block, and label with specimen ID.

***NOTE:** If your facility is uncomfortable obtaining the plug but wants to retain the tissue block, please send the entire block to the RTOG Biospecimen Resource and we will sample a plug from the block and return the remaining block to your facility. Please indicate on the submission form the request to perform the plug procedure and return of the block.
Ship specimen plug kit, specimen in punch tool, and all paperwork to the address below. For Questions regarding collection/shipping or to order an FFPE Specimen Plug Kit, please contact the RTOG Biospecimen Resource by e-mail: RTOG@ucsf.edu or call 415-476-RTOG(7864)/Fax 415-476-5271.

U.S. Postal Service Mailing Address: For Non-frozen Specimens Only
RTOG Biospecimen Resource
University of California San Francisco
Campus Box 1800
2340 Sutter St, Room S341
San Francisco, CA 94143-1800

Courier Address (FedEx, UPS, etc.): For Frozen Specimens or Trackable shipments
RTOG Biospecimen Resource
University of California San Francisco
2340 Sutter St, Room S341
San Francisco, CA 94143-1800

RTOG BLOOD COLLECTION KIT INSTRUCTIONS

This Kit is for collection, processing, storage, and shipping of serum, plasma, or whole blood (collected as required by protocol):

Kit contents:
- One Red Top tube for serum (A)
- One Purple Top EDTA tube for plasma (B)
- One Purple Top EDTA tube for Whole Blood (C)
- Twenty-five (25) 1 ml cryovials
- Biohazard bags (3) and Absorbent shipping material (3)
- Styrofoam container (inner) and Cardboard shipping (outer) box
- UN1845 DRY Ice Sticker and UN3373 Biological Substance Category B Stickers
- Specimen Transmittal Form (STF) and Kit Instructions

PREPARATION AND PROCESSING OF SERUM, PLASMA AND WHOLE BLOOD:

(A) Serum (if requested): Red Top Tube

- ❑ Label as many 1ml cryovials (5 to 10) as necessary for the serum collected. Label them with the RTOG study and case number, collection date, time, and time point, and clearly mark cryovials "serum".

Process:
1. Allow one red top tube to clot for 30 minutes at room temperature.
2. Spin in a standard clinical centrifuge at ~2500 RPM for 10 minutes at 4°C (preferred). If sites are unable to process samples at 4°C then spinning at room temperature is acceptable if done within 2 hours of draw but must be noted on the STF.
3. Aliquot 0.5 ml serum into as many cryovials as are necessary for the serum collected (5 to 10) labeled with RTOG study and case numbers, collection date/time, protocol time-point collected (e.g. pretreatment, post-treatment), and clearly mark specimen as "serum".
4. Place cryovials into biohazard bag and immediately freeze at -70 to -90° C, and store frozen until ready to ship. See below for storage conditions.
5. Store serum at -70 to -90° C until ready to ship on dry ice. See below for storage conditions.

PLEASE MAKE SURE EVERY SPECIMEN IS LABELED and include collection time point on the STF.

(B) Plasma (If requested): Purple Top EDTA tube #1

- ❑ Label as many 1ml cryovials (5 to 10) as necessary for the plasma collected. Label them with the RTOG study and case number, collection date, time, and time point, and clearly mark cryovials "plasma".

Process:
1. After collection, invert tube(s) multiple times to ensure adequate mixing of EDTA.
2. Centrifuge specimen(s) within one hour of collection in a standard clinical centrifuge at ~2500 RPM for 10 minutes at 4°C (preferred). If sites are unable to process samples at 4°C then spinning at room temperature is acceptable if done within 2 hours of draw but must be noted on the STF..
3. If the interval between specimen collection and processing is anticipated to be more than one hour, keep specimen on ice until centrifuging is performed.
4. Carefully pipette and aliquot 0.5 ml plasma into as many cryovials as are necessary for the plasma collected (5 to 10) labeled with RTOG study and case numbers, collection date/time, time point collected and clearly mark specimen as "plasma". Avoid pipetting up the buffy coat layer.
5. Place cryovials into biohazard bag and immediately freeze at -70 to -90°C.
6. Store frozen plasma until ready to ship on dry ice.
7. See below for storage conditions.

PLEASE MAKE SURE EVERY SPECIMEN IS LABELED and include collection time point on the STF.

(Appendix V Continued on next page)

RTOG BLOOD COLLECTION KIT INSTRUCTIONS (continued)

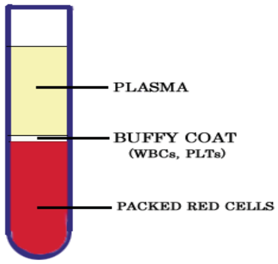

(C) Whole Blood for DNA (if requested): Purple Top EDTA tube #2

- Label as many 1ml cryovials (3 to 5) as necessary for the whole blood collected..Label them with the RTOG study and case number, collection date/time, and time point, and clearly mark cryovials "blood".

Process:

1. After collection, invert tube(s) multiple times to ensure adequate mixing of EDTA. Blood can also be mixed for 5 minutes on a mixer at room temperature.
2. Carefully pipette and aliquot 1.0 ml blood into as many cryovials as are necessary for the blood collected (3 to 5) labeled with RTOG study and case numbers, collection date/time, time point collected and clearly mark specimen as "blood".
3. Place cryovials into biohazard bag and freeze immediately at -70 to -80° Celsius.
4. Store blood samples frozen until ready to ship on dry ice.
5. See below for storage conditions.

PLEASE MAKE SURE THAT EVERY SPECIMEN IS LABELED and include collection time point on STF.

Freezing and Storage:

- Freeze Blood samples in a -80°C Freezer or on Dry Ice or snap freeze in liquid nitrogen.
- Store at –80°C (-70°C to -90°C) until ready to ship.

  If a -80°C Freezer is not available,

  - Samples can be stored short term in a -20°C freezer (non-frost free preferred) for up to one week (please ship out Monday-Wednesday only; Canada: Monday-Tuesday only).

  OR:

  - Samples can be stored in plenty of dry ice for up to one week, replenishing daily (please ship out on Monday-Wednesday only; Canada: Monday-Tuesday only).

  OR:

  - Samples can be stored in liquid nitrogen vapor phase (ship out Monday-Wednesday only; Canada: Monday-Tuesday only).
- Please indicate on Specimen Transmittal Form the storage conditions used and time stored.

Shipping/Mailing:

- Ship specimens on Dry Ice overnight **Monday-Wednesday (Monday-Tuesday from Canada)** to prevent thawing due to delivery delays. Saturday and holiday deliveries cannot be accepted.
- Include all RTOG paperwork in a sealed plastic bag and tape to the outside top of the Styrofoam box.
- Wrap frozen specimens of same type (i.e., all serum together, plasma together and whole bloods together) in absorbent shipping material and place each specimen type in a separate biohazard bag. Place specimen bags into the Styrofoam cooler and fill with plenty of dry ice (7-10 lbs/3.5kg minimum). ***Add padding to avoid the dry ice from breaking the tubes.***
- Place Styrofoam coolers into outer cardboard box, and attach shipping label and UN3373 and UN1895 stickers to outer cardboard box.
- *Multiple cases may be shipped in the same cooler, but make sure each one is in a separate bag and that there is enough room for plenty of dry ice.* ***Add padding to avoid the dry ice from breaking the tubes.***
- **For questions regarding collection, shipping or to order a Blood Collection Kit, please e-mail RTOG@ucsf.edu or call (415)476-7864.**

Shipping Address:

Courier Address (FedEx, UPS, etc.): For all Frozen Specimens
RTOG Biospecimen Resource
University of California San Francisco
2340 Sutter St, Room S341
San Francisco, CA 94143-1800
For questions, call 415-476-RTOG (7864) or e-mail: RTOG@ucsf.edu

(Appendix V Continued on next page)

APPENDIX V (cont'd)
RTOG URINE COLLECTION KIT INSTRUCTIONS

Kit Contents:

- One (1) Sterile Urine collection cup
- Two 7 ml disposable pipettes
- Absorbent paper towel
- Two 15 ml polypropylene centrifuge tubes
- Biohazard bags
- Parafilm for sealing outside of tubes

Preparation and Processing of Urine Specimens:

Process:

- A clean catch urine specimen will be collected. To collect the specimen, use the following instructions:
  - Males should wipe clean the head of the penis and females need to wipe between the labia with soapy water/cleansing wipes to remove any contaminants.
  - After urinating a small amount into the toilet bowl to clear the urethra of contaminants, collect a sample of urine in the collection cup.
  - After 10-25 mL urine has been collected, remove the container from the urine stream without stopping the flow of urine.
  - Finish voiding the bladder into the toilet bowl.
- Aliquot 5-10 mls of Urine into each of two 15 ml polypropylene centrifuge tubes (disposable pipets are provided in the kit). Do not fill with more than 10 mls to avoid cracking of tubes due to expansion during freezing. Replace the cap and tighten on the tubes. Make sure the cap is not cross-threaded or placed on incorrectly or leaking will occur.
- Use parafilm to seal the cap around the outside rim of the urine tube to prevent leakage.
- Discard remaining Urine and collection cup.
- Label the specimen with the RTOG study and case number, collection date and time, time point of collection, and clearly mark specimens as "urine".
- Wrap Urine Tubes with absorbent material (paper towels) and place into biohazard bag and seal the bag. Freeze and store Urine samples in a -20°C or -80°C freezer until ready to ship.

PLEASE MAKE SURE EVERY SPECIMEN IS LABELED with RTOG study and case numbers, collection date/time, and time point collected (e.g. pretreatment, post-treatment).

Storage and Shipping:

Freezing and Storage:

- ❑ Urine specimens may be sent in batches or with other frozen biospecimens, if within 30-60 days of collection. Store at -20°C or -80°C (-70°C to -90°C) until ready to ship. If a -80°C Freezer is not available:
  - Samples can be stored short term in a -20° C freezer (non-frost free preferred) for up to one week (please ship out Monday-Wednesday only; Canada: Monday-Tuesday only).

  OR:
  - Samples can be stored in plenty of Dry Ice for up to one week, replenishing daily (please ship out Monday-Wednesday only; Canada: Monday-Tuesday only).
- Please indicate on Specimen Transmittal Form the storage conditions used and time stored.

Shipping/Mailing:

- ❑ Ship specimens on Dry Ice overnight **Monday-Wednesday (Monday-Tuesday from Canada)** to prevent thawing due to delivery delays. Saturday and holiday deliveries cannot be accepted.
- ❑ Include all RTOG paperwork in a sealed plastic bag and tape to the outside top of the Styrofoam box.
- ❑ Place sealed specimen bags into the Styrofoam cooler and fill with plenty of dry ice (7-10 lbs/3.5kg minimum). ***Add padding to avoid the dry ice from breaking the tubes.***
- ❑ Place Styrofoam coolers into outer cardboard box, and attach shipping label and UN3373 and UN1895 stickers to outer cardboard box.
- ❑ *Multiple cases may be shipped in the same cooler, but make sure each one is in a separate bag and that there is enough room for plenty of dry ice.* ***Add padding to avoid the dry ice from breaking the tubes.***
- ❑ Samples received thawed will be discarded, and a notification will be sent immediately to the Principal Investigator and Clinical Research Assistant of the submitting institution. The institution should send a subsequent sample, if it can still be collected as close as possible to the original planned collection date.

- **For questions regarding ordering, collection, or shipping of a Urine Collection Kit, please e-mail <u>RTOG@ucsf.edu</u> or call (415)476-7864 or fax (415) 476-5271.**

<u>Shipping Address: FedEx/UPS/Courier address</u> (For all frozen samples)
RTOG Biospecimen Resource at UCSF
2340 Sutter St, Room S341
San Francisco, CA 94143-1800
Contact Phone: (415) 476-RTOG(7864)
