# Supplementary material for: Association of Radiotherapy Duration With Clinical Outcomes in Patients With Esophageal Cancer Treated in NRG Oncology Trials: A Secondary Analysis of NRG Oncology Randomized Clinical Trials
Source: JAMA Netw Open. 2023 Apr 21;6(4):e238504. doi: 10.1001/jamanetworkopen.2023.8504 (PMC10122174; doi:10.1001/jamanetworkopen.2023.8504)
Supplement: Supplement 2. — RTOG 8501 Protocol [file jamanetwopen-e238504-s002.pdf]

RADIATION THERAPY ONCOLOGY GROUP

RTOG #85-01

NCCTG 88-40-51

SWOG #85-98

PHASE III PROSPECTIVE TRIAL FOR LOCALIZED CANCER OF THE ESOPHAGUS: COMPARING RADIATION AS A SINGLE MODALITY TO THE COMBINATION OF RADIATION THERAPY AND CHEMOTHERAPY

RTOG Chairmen
(Managing Group)

Radiation Oncology Chairman: Arnold M. Herskovic, M.D.
(313) 745-9191

Medical Oncology Chairman: Muhyi Al-Sarraf, M.D.
(313) 745-2747

SWOG Chairmen

Study Chairmen: Claudia Perez-Tamayo, M.D.
(313) 936-4301

John S. McDonald, M.D.
(606) 233-5038

NCCTG Chairmen

Radiation Oncology Chairman: Jeffrey S. Brindle, M.D.
(507) 284-4561

James Martenson, M.D.
(507) 284-4561

Medical Oncology Chairmen: James Mailliard, M.D.
(402) 280-4373

Michael J. O'Connell, M.D.
(507) 284-3903

Date Activated: January 2, 1986
Current Edition: May 14, 1990
Includes Amendments 1-13

INDEX

Schema

Eligibility Check

1.0 Introduction

2.0 Objectives

3.0 Patient Selection

4.0 Pretreatment Evaluation

5.0 Registration

6.0 Radiation Therapy

7.0 Drug Treatment

8.0 Surgery

9.0 Other Therapies

10.0 Pathology

11.0 Patient Assessments

12.0 Data Collection

13.0 Statistical Considerations

14.0 Additional Treatment

References

Appendix I - Sample Consent Form

Appendix II - Late Effects Radiation Morbidity Scoring Sheet

Appendix III - Medical Oncology Toxicity Criteria

Appendix IV - Acute Radiation Morbidity Scoring Sheet

Appendix V - Staging System

Appendix VI - Intergroup Participation Instructions **(Added 7/86)**

RTOG #85-01
SWOG #85-98
NCCTG #88-40-51

PHASE III PROSPECTIVE TRIAL FOR LOCALIZED CANCER OF THE ESOPHAGUS: COMPARING RADIATION AS A SINGLE MODALITY TO THE COMBINATION OF RADIATION THERAPY AND CHEMOTHERAPY

SCHEMA

S		R	
T	1. Weight Loss in last	A	I. Chemotherapy + RT (Both chemo & RT
	6 months		to start on day 1)
R	$\geq$ 10 Lbs	N	
	vs.		CDPD - 75 mg/m^2 - first day of
A	$<$ 10 Lbs	D	wks. 1,5,8,11
T		O	5-FU - 1000 mg/m^2 x 4 days-
			wks. 1,5,8,11
I	2. Lesion Size	M	
	$\geq$ 5 cm		RT - 2 Gy x 5 days/wk x 3 wks
F	vs.	I	(30 Gy) followed by boost of
	$<$ 5 cm		2 Gy x 5 days/wk x 2 wks (20 Gy)
Y		Z	
			II. Radiation Alone (Closed 5/14/90)
	3. Histology	E	
	Squamous		2 Gy x 5 days/wk x 5 wks (50 Gy)
	Adenocarcinoma		+
	(10/14/86)		Boost - 2 Gy x 5 days/wk
			x 1.4 wks. (14 Gy)

Eligible

- Squamous cell carcinoma or adenocarcinoma (10/14/86) of the thoracic esophagus
- No evidence of disseminated cancer
- Negative bone scan
- WBC $\geq$ 4,000/mm, platelets $\geq$ 100,000/mm, creatinine $\leq$ 1.5 mg%, BUN $\leq$ 22 mg%, and/or creatinine clearance $\geq$ 60 cc/min

Amended: 10/14/86
5/14/90

ELIGIBILITY CRITERIA
RTOG #85-01
SWOG #85-98
NCCTG #88-40-51

1. Does patient have a biopsy proven squamous cell carcinoma or adenocarcinoma **(10/14/86)** of the thoracic esophagus? ________
2. Is there evidence of disseminated cancer by physical exam? ________
3. Has a CT scan of the abdomen been done? ________
4. If CT scan of the liver was abnormal, report results of liver biopsy. ________
5. If patient had enlarged retroperitoneal or celiac nodes per CT, report results of laparotomy and biopsy.
6. Report following study results:

   WBC (≥ 4,000/mm^3) ________
   Platelet (≥ 100,000/mm)^3 ________
   Serum creatinine (≤ 1.5) ________
   BUN (≤ 22 mg%) ________
   Creatinine clearance (≥ 60 cc/min) ________

7. Report results of endoscopy of tracheal bronchial tree and upper aerodigestive tract. ________
8. Has patients entry on study been sanctioned by a Medical & Radiation Oncologist? ________

   Provide Names: ________________ Medical Oncologist
   ________________ Radiation Oncologist

9. Has patient received prior chemo, chest irradiation or surgery **(6/29/87)**? ________
10. Has patient signed a study specific informed consent? ________
11. Report results of bone scan. ________
    If bone scan positive, without a benign explanation **(4/15/87)**, report results of bone biopsy. ________
12. Has patient had a second malignancy other than a curable skin cancer or cervical in-situ cancer? ________

STRATIFICATION QUESTIONS

1. Weight loss in last 6 months? (≥ 10 lbs vs. < 10 lbs.) ________
2. Lesion size? (≥ 5 cm vs. < 5 mc) ________
3. Histology? (Adenocarcinoma vs. Squamous) ________

1.0 INTRODUCTION

Surgery and radiation therapy have had a minimal impact on prolonging survival for most patients with squamous cell cancer of the esophagus. The five year survival rate for patients who are considered "operable candidates" varies in the literature between five and twenty percent. Yet, a compendium of the world's literature on this subject recently revealed that less than eight percent of patients operated on for cure survive two years after operation. The same survival figures hold true for those patients treated with radiation alone in an effort to produce a cure.(1-5)

A number of clinical trials have suggested that squamous cell tumors of the esophagus are sensitive to chemotherapy. Data regarding the efficacy of 5-fluorouracil, cisplatin, bleomycin, mitomycin (standard agents), as well as vindesine and MGBG (phase II agents) have been published and combinations of these drugs as a single modality or with radiation and surgery have been investigated and reported.(6-10) While results have been generally encouraging, the overwhelming majority of patients treated in each trial continue to succumb to their cancer or in certain instances to the treatment. For example, at Wayne State University a combination of chemotherapy and radiation therapy followed by surgery produced a median survival of over 18 months, but thirty percent of the patients treated failed to leave the hospital following surgery.(6)

The experience at Wayne State University has been particularly interesting and provocative. Leichman et. al. have reported on 55 patients treated with 5-fluorouracil and mitomycin-C (later 5-fluorouracil and cisplatin) coupled with radiation therapy (3000 rad over 3 weeks), followed by surgery: Twenty five percent of all patients treated who underwent resection were found to have no cancer in the specimen when it was examined by the surgical pathologist.(6) These patients had a laparotomy and then thoracotomy.(11) Those patients without cancer in the surgical specimen have survived without recurrent cancer of the esophagus. Of further interest is that for those patients in whom tumor is found in the surgical specimen over 80% die from distant disease after they undergo a second course of radiation therapy (20.00 Gy over 2 weeks) to the mediastinum (or upper epigastrium if tumor is found in the celiac nodes). In this patient population pre-staging with CT scans of the abdomen or thorax was inadequate to determine which patients would become complete responders.

Recently, preliminary data for a similar group of patients with potentially curable squamous cell cancer of the esophagus has become available from the Southwest Oncology Group and the Radiation Therapy Oncology Group.(7) These trials have used cisplatin at 75 mg/M^2 days 1 and 29, 5- fluorouracil 1000 mg/M^2 as a continuous infusion over 24 hours for four days on days 1-4 and 29-32. Radiation started day 1 with chemotherapy and was given at a rate of 2.00 Gy/day five days per week for a total of 3 weeks. Surgery took place approximately 4 weeks after the last day of chemotherapy. The pathology and surgical reports on over 140 patients have been reviewed and reveal that 22% of those entering the clinical trials have been operated on and found to have "no cancer" as reported by the pathologist who examined the operative specimen. In these studies the mortality rate from the treatment (those who did not leave the hospital after surgery) was 9%. While white blood cell counts and platelet counts below 1000/mm and 50,000/mm respectively have been reported, no mortality from chemotherapy and radiation has been reported.

Analysis of the 55 patients treated at WSU revealed that local control of the tumor can be achieved for the vast majority of patients. In fact 80% of the patients who develop recurrence do so in distant sites beyond the fields of radiation and surgery. This has led to the postulate that the prognosis for each patient is determined by the result of the pre-operative therapy. Ninety two percent of those without cancer in the resected esophagus have survived a median period of 3.5 years or have died of causes other than cancer of the esophagus. The patients with residual tumor present after the pre-operative therapy was completed, however, have expired from cancer of the esophagus.

The data from the sources quoted suggest, but do not prove, that chemotherapy may have an important role in the curative treatment of cancer of the esophagus. Moreover, the role of surgery in this group of patients in terms of adding to the cure rate is suspect. The fact that surgery has significant mortality and morbidity for this group of patients, whether it is used alone or after chemotherapy and radiation therapy, has led the investigators at Wayne State University to initiate a program of chemotherapy and radiation therapy without surgery.

We believe that an important question can be answered regarding the role of chemotherapy in the curative regimen for this solid tumor. We have designed a prospective randomized clinical trial in which one-half of the patients eligible will receive potentially curative radiation therapy and the other half radiation therapy plus chemotherapy.

The combined modality arm will build on the SWOG and RTOG experience. It is believed that at the very best only 20-25% of the patients entered onto the prior trials will be cured or long term (3 year) survivors, therefore, additional chemotherapy has been added after the patient has been treated with 5FU and cisplatin and 50.00 Gy given 2.00 Gy/day. The additional chemotherapy consists of another 2 similar courses of cisplatin and 5-FU.

At Wayne State University 14 patients have been treated with radiation and the 4 day drug regimen including bleomycin and mitomycin C. The first patient treated in this fashion started therapy in August 1983 and the last started in September 1984. All patients had a clinical complete response but there were 3 marginal failures. Because of that we will extend the initial RT volume for the 30 Gy arm then limit the volume for the 20 Gy boost(4/21/86).

Previous experience revealed that over 90% of patients treated and responding had no residual tumor by esophagoscopy, yet only 20-25% were truly tumor free at the time of operation.

The arm using radiation therapy alone will give a continuous course of radiation to 64.00 Gy covering a field which will include the supraclavicular fossa for patients with upper and mid esophageal cancers. Every effort will be made to guarantee identical support and follow-up to all patients treated on this protocol. Patients will be stratified by weight loss and lesion size before randomization.

2.0 OBJECTIVES

2.1 To determine the role of chemotherapy for a potentially curable subset of patients with adeno or squamous cell cancer of the esophagus. Specifically, to determine if the combination of chemotherapy and radiation will add to the overall survival and cure of patients treated with the combination when compared to patients treated by radiation alone.

2.2 To determine if the patterns of recurrence for patients treated with the combination of chemotherapy and radiation differs from those patients treated with radiation alone.

3.0 PATIENT SELECTION

3.1 Eligibility Criteria

3.1.1 The patient must have biopsy proven squamous cell carcinoma or adenocarcinoma (10/14/86) of the thoracic esophagus.

3.1.2 There must be no evidence of disseminated cancer by physical examination. Physical examination of pertinent lymph nodes and organs must appear on the "on study" forms.

3.1.3 Pre-entry CT of abdomen is required. Abnormal evaluation by CT of the liver must be followed with a negative liver biopsy before a patient can be considered eligible to enter the study. Patients with enlarged retroperitoneal or celiac lymph nodes that can be found by CT should receive a laparotomy and biopsy with proof of "no cancer" before study entry; otherwise they must be considered ineligible.

3.1.4 Bone scan is a prestudy requirement. Patients with positive bone scans, unexplained from benign disease (4/15/87), are ineligible unless a biopsy is done to rule out tumor in the positive area(s).

3.1.5 No age or sex restriction.

3.1.6 Pre-study WBC must be greater or equal to 4,000/mm and platelet count must be greater than or equal to 100,000/mm at the outset of therapy.

3.1.7 Serum creatinine of less than or equal to 1.5 mg% and BUN less than or equal 22 mg% and/or creatinine clearance $\geq$ 60cc/min before entry to study and prior to the second and third cycle of cisplatin.

3.1.8 Endoscopic appearance of the tracheal bronchial tree must be performed and must be normal. The same is true for the structures of the upper aerodigestive tract. This should be noted on the pre-study form.

3.1.9 Patient must not have had a second malignancy other than curable non-melanoma skin cancer or cervical cancer in situ and not have received chest radiation or chemotherapy.

3.1.10 Patient must sign informed consent.

3.1.11 Medical Oncologist Evaluation

3.2 Ineligibility Criteria

3.2.1 Failure to perform the studies specified in 3.1 with findings consistent with the criteria noted

3.2.2 Patients without biopsy proven adenocarcinoma or squamous cell carcinoma (10/14/86).

3.2.3 Evidence of disseminated cancer

3.2.4 Positive bone scan, unexplained from benign disease (4/15/87).

3.2.5 Positive liver biopsy

3.2.6 WBC < 4,000/mm^3, platelets < 100,000/mm^3

3.2.7 BUN > 22 mg% (5/22/87), creatinine > 1.5 mg%, creatinine clearance < 60 ml/min

3.2.8 Failure to sign the informed consent

3.2.9 Prior chemotherapy, chest irradiation or surgical resection of tumor (6/29/87)

3.2.10 No evaluation by medical oncologist prior to study entry.

4.0 PRETREATMENT EVALUATION

4.1 Mandatory Evaluation

4.1.1 Complete history and physical exam including weight with an assessment of the patient's performance status.

4.1.2 The patient must be evaluated by a Medical and Radiation Oncologist prior to study entry.

4.1.3 Laboratory Studies

4.1.3.1 CBC, platelets

4.1.3.2 Creatinine clearance if serum creatinine is > 1.5%

4.1.4 Imaging Studies:

4.1.4.1 CT Scan of Abdomen

4.1.4.2 Bone Scan

4.1.4.3 Esophagram

4.1.4.4 Chest X-ray

4.1.5 Endoscopic Study of tracheal bronchial tree

4.1.6 Bilateral audiogram

5.0 RANDOMIZATION

5.1 To place an eligible patient on study an institution will first phone the registration desk at their Cooperative Group and provide the following information:

- Study Number
- Patient's name
- Institution's Name
- Physician's Name
- Responsible Medical Oncologist
- Responsible Radiation Therapist
- Weight Loss
- Lesion Size + location
- Histology
- WBC, platelets, BUN, serum creatinine, creatinine clearance
- All information listed in 3.1 & 3.2

5.2 The Cooperative Group will then call RTOG Headquarters between 9:00 a.m. and 5:00 p.m., ET, at (215) 574-3191 and relay this information. A treatment and project case number will be assigned which will be confirmed by mail.

5.3 The Cooperative Group will then inform its member institution of the study case number and treatment assignment.

5.4 Treatment must commence within 8 days of randomization.

6.0 RADIATION THERAPY

Combined Therapy (Arm I): The initial radiation therapy is with megavoltage fields extending superiorly to the supraclavicular fossa including the same and inferior to the esophago gastric junction. If the lesion is in the distal esophagus, a minimum 5 cm margin is required. The lateral margins should

include the mediastinum, typical widths being 7 or 8 cm. This field should receive 30 Gy at 2 Gy per day (anterior/posterior 2 fields/day) 5 days a week for 3 weeks. The radiation boost should be treated with a three field technique (anterior/2 posterior obliques) or a 4 field technique (4 obliques) to avoid exceeding spinal cord tolerance. The boost field will include initial tumor on esophagram with a 5 cm margin cephalad and caudad and adjacent mediastinum with at least a 1 cm lateral margin. 20.0 Gy will be delivered 2.0 Gy daily fractions 5 times a week x 2 weeks. All portals shall be treated daily.

Radiation Alone (Arm II) (Closed 5/14/90): Target volume will include the tumor volume as identified from an esophagram with a 5 cm margin cephalad and caudad to the tumor, and a 1 cm margin around the mediastinal structures. The supraclavicular areas will be included in continuity in *lesions of* the middle and upper third of the thoracic esophagus.

50.00 Gy will be delivered in 2.00 Gy fractions daily, 5 times a week in 5 weeks to the esophagus and supraclavicular area. Localized boost to the supraclavicular area with photons and electrons is allowed if the SCF dose is less than 50.00 Gy. Total dose to the supraclavicular region is 50 Gy specified at 3 cm depth from the anterior skin surface.

Treatment may be given in a combination of anteroposterior, posterior oblique, or lateral fields, such that the maximum does not exceed minimum target volume dose by more than 15%. The normal tissue tolerances mentioned below will need to be taken into consideration.

A boost field will include initial tumor on esophagram with a 5 cm margin cephalad and caudad and adjacent mediastinum with at least a 1 cm lateral margin. 14.0 Gy will be delivered in 2.0 Gy daily fractions 5 times a week in 1 1/2 weeks. All portals shall be treated daily.

6.1 Technical Factors

6.1.1 Beam Energy megavoltage equipment is required.

6.1.2 Treatment Distance: Minimal treatment distance to skin should be 80 cm for SSD technique and minimum isocenter distance should be 80 cm for SAD techniques.

6.1.3 Blocking:

a) In the case of x-ray beams, the primary collimation may be used, and blocking will be required only for shaping of the ports to exclude volume of tissues that are not to be irradiated.

b) However, with Cobalt 60, beam trimmers or secondary blocking all the way around the ports will be required.

6.1.4 Compensating Filters:

a) In case of sloping surface such as the thoracic inlet, compensating filters are recommended.

b) If compensating filters are not available, appropriate reductions in field size must be done at prescribed dose levels to avoid excessive irradiation of a particular area.

6.1.5 Normal Tissue Doses:

The spinal cord dose must not exceed 42.5 Gy maximum for radiation therapy with chemotherapy (arm I), and 45.0 Gy maximum must not be exceeded for the radiation therapy alone arm (arm II).

Normal Lung (more than 2 cm outside the target volume) must not receive more than 45.0 Gy. The entire heart dose should be no more than 40 Gy with $< 50\%$ of the organ receiving a maximum of 45 Gy.

6.1.6 Fractionation:

a) Each field to be treated every day.

b) Adherence to the fractionation scheme is required although slight deviations in the daily dose fractions are allowed (plus or minus 10% daily dose).

6.1.7 Therapy Interruptions:

a) If interruption of therapy, up to two weeks, becomes necessary, irradiation should be completed to the prescribed doses. Total number of fractions and elapsed days should be carefully reported.

b) If more than two weeks' interruption, resumption of treatment is at the discretion of the radiation therapist. Patient will be considered a major deviation although follow-up will be continued.

6.1.8 Radiotherapy should be discontinued if absolute granulocyte count becomes equal or less than

1000/mm and/or platelets less than or equal to 100,000/mm. Treatment may be resumed when these values are exceeded.

6.2 Treatment Planning

(Use of CT strongly recommended):

a) Isodose distribution at the mid-transverse plane of the tumor should be submitted. For the purpose of the distribution, it may be assumed that the central axis passes through the midplane of the tumor.

b) In addition to this distribution, two specific points of calculation are requested.

(i) The upper most irradiated spinal cord dose 2 cm below the superior margin of the field, and

(ii) Supraclavicular node dose at 3 cm anterior depth, if applicable.

(iii) Doses are prescribed and calculated without tissue inhomogeneity connection.

6.3 Dose Specifications

For the following portal arrangements, the target dose shall be specified as follows:

a) For two opposed coaxial equally weighted beams: on the central ray at mid-separation of beams.

b) For an arrangement of 2 or more intersecting beams: at the intersection of the central ray of the beams.

c) For complete rotation or arc therapy: in the plane of rotation at the center of rotation.

d) For a single beam: on the central ray at the center of the target area.

e) For two opposing coaxial unequally weighted beams: on the central ray at the center of the target area.

f) Other or complex treatment arrangements: at the center of the target area (Note: there may be several target areas).

6.3.1 The dose should not vary by more than 15% over the entire target volume.

6.4 Localization Methods

Localization films taken on treatment simulators are required using contrast material in the esophagus. Verification portal films taken on the treatment unit are required. Supraclavicular nodes that are involved should be outlined with a wire on the simulation film.

6.5 Dosimetry Monitoring: The Radiological Physics Center will conduct a field survey and will monitor studies of quality control in the participating institutions. Copies of the daily radiation therapy treatment records and localization films of each port are to be sent to headquarters offices with appropriate radiation therapy forms.

7.0 DRUG THERAPY

7.1 Drug Therapy

7.1.1 5-fluorouracil (NSC 19893) 5 FU

7.1.1.1 Pharmaceutical Data:

7.1.1.1.1 Dosage Formulation: 500 mg ampules containing 50 mg/cc.

7.1.1.1.2 Storage: Room temperature

7.1.1.1.3 Administration: 96 hour continuous infusion via an indwelling catheter.

7.1.1.1.4 Supplier: Commercially available.

7.1.1.2 Side Effects in Man: Side effects from the 96 hours infusion of 5FU include anorexia, nausea, and vomiting, mucositis, myelosuppression, maculopapular eruption, alopecia, hyperpigmentation, and photosensitization.

7.1.2 Cis-Diaminedichloroplatinum II NSC 119885 Cisplatin

7.1.2.1 Pharmaceutical Data:

7.1.2.1.1 Dosage Formulation: Vial containing 10 mg cisplatin with 10 mg mannitol and 9 mg NaC1.

7.1.2.1.2 Storage: Refrigeration

7.1.2.2 Administration and Prep: 1 vial diluted with 10 ml of sterile water. The drug should be given immediately after preparation. It should be diluted in no less than 250 cc of appropriate isotonic fluids and given at 1 mg/minute.

7.1.2.3 Side Effects in Man: Clinical toxicity consists primarily of nausea and vomiting. There is renal toxicity manifested with elevation of BUN, creatinine and the clinical manifestations of

azotemia. There is histologic evidence of renal tubular damage which appears to be transient. This drug also causes ototoxicity with tinnitus and high frequency hearing loss--this appears to be dose related. Those with prior renal disease or hearing impairment may be more severely affected by these toxicities. The drug may cause myelosuppression, platelet suppression and delayed erythropoeisis. The bone marrow effect is generally seen only after 300 mg/M^2 of cisplatin or for those patients previously treated with chemotherapy. In doses greater than 300 mg/M^2 peripheral neuropathy is seen in 5-10% of patients.

7.1.2.4 Supplier: cisplatin is now commercially available.

7.2 Chemotherapy Treatment (Arm I only)

7.2.1 Chemotherapy (To begin first day of weeks 1,5,8,11)

7.2.1.1 Cisplatin

7.2.1.1.1 Dose of Cisplatin will be 75 mg/M^2 given at a rate of 1 mg/min on days 1,29,50,71 **(4/21/86)**. No dose to exceed 150 mg.

7.2.1.1.2 The patient must receive at least 2000 cc fluid by either the IV or oral route during the 24 hours prior to cisplatin administration.

7.2.1.1.3 Mannitol 12.5 grams will be administered IV bolus one half hour prior to cisplatin administration.

7.2.1.1.4 Mannitol 25 grams in 1000 cc 5% dextrose and one half normal saline will be administered intravenously over 4 hours immediately after cisplatin administration.

7.2.1.2 5-fluorouracil will be administered in a dose of 1,000 mg/M^2/day as a continuous infusion for first four days of weeks 1,5,8,11. DAILY DOSE MUST NOT EXCEED 2000 MG. 5-fluorouracil may be diluted in 5% dextrose and water alone or with 5% dextrose and water with a desired concentration of NaCl.

7.2.1.3 Dose Modifications for 5-fluorouracil and Cisplatin:

7.2.1.3.1 If BUN and serum creatinine do not return to normal (less than or equal to 20 mg% and 1.5 mg% respectively) by days 29, 50, and 71, the specific cycle of cisplatin due to be administered that day will be withheld permanently for the remainder of the study.

7.2.1.3.2 No dose reduction for cisplatin is mandated for transient elevations of BUN and serum creatinine.

7.2.1.3.3 Dose modification for cisplatin based on hematologic toxicity:

NADIR MODIFICATIONS

CISPLATIN DOSE	WBC	PLATELETS
No Change	3000/mm and	75,000/mm
Decrease 25%	1000-2999 and/or	50,000-74,999
Decrease 50%	< 1000 and/or	< 50,000

7.2.1.3.4 If stomatitis develops during the 5-fluorouracil infusion, the infusion should be stopped and reinstituted at a 25% reduction in dose on the first day of the next cycle. If grade 1 or 2 stomatitis develops between administrations, no dose modification is necessary. If stomatitis (gr 3) develops between administrations the next cycle dose of 5-FU is to be reduced by 25%.

7.2.1.3.5 The day 29,50, and 71 **(4/21/86)** 5-fluorouracil infusion can be administered even if bone marrow suppression is evident, provided absolute granulocyte count is equal to or greater than 1,000/mm and platelets equal to or greater than 50,000/mm.

7.2.2 Radiation Therapy with Chemotherapy (Arm I)

7.2.2.1 First Course (Begin Day 1): The fields should include the esophagus, supraclavicular fossa and adjacent mediastinum with 1 cm margin. The cephalad boundary should be 5 cm above the level of the lesion, and the caudad boundary 5 cm below the lower level of the lesion based on the esophagram. Width of port usually will be approximately 8 cm.

Radiation therapy may be given with parallel opposed anterior and posterior fields.

7.2.2.2 Dose: 30.00 Gy at 2.00 Gy daily, 5 times a week.

7.2.2.3 Second course: Following the first course, the volume is defined as having 5 cm proximal and distal margins around the tumor; it will be treated using a three field technique or 4 oblique fields. The treatment plan will be chosen to keep the cord dose < total 42.50 Gy. 20.00 Gy will be delivered at 2.00 Gy daily, 5 days a week.

8.0 SURGERY

Not applicable to this protocol.

9.0 OTHER THERAPIES

Not applicable to this protocol.

10.0 PATHOLOGY

The Pathology Committee has not planned a central review for this study. The committee feels that the review can be done adequately on the institutional level.

11.0 PATIENT ASSESSMENT

Study Parameters

Assessment	Pre Rx	Weekly	Biweekly	Post Rx 3 mos	6 mos	at each follow-up (Amended 6/12/89)
History and Physical Exam (with K.P.S.)	X	X		X		X
Toxicity Notation		X		X		X
Endoscopy w/ Biopsy of Esoph.**	X[1]			X[2]		
Pre-study Weight Loss	X					
Esophagram	X[3]			X[4]		
CT Abdomen & mediastinum	X				X	
Bronchoscopy	X					
Total Bilirubin	X			3 mos	6 mos	X[5]
CBC, Platelets	X	X			X	X[5]
BUN, serum creatinine, creatinine clearance	X	X				X[5]
Serum Electrolytes	X	X				X[5]
Chest x-ray	X			X	X	X[5]
Bone Scan	X			X		X[5]
Pulmonary Function	X				X	X[5]
Serum Calcium	X				X	X[5]
SGOT, Alk phos, LDH	X				X	X[5]
Bilateral audiogram	X					
Medical Oncologist's Evaluation	X					

1. Endoscopist should describe tumor, including an estimate of the length from incisors to proximal margins of the tumor.
2. May be done as soon as 4 weeks after therapy is completed. Needs to be done only once if no symptoms develop.
3. Radiologist must report dimensions of lesions in esophagram reports. pre- and post-treatment.
4. Every 6 months thereafter.
5. As indicated.

11.1 Criteria for Response

11.1.1 All tumor measurements must be recorded in centimeters and should consist of the longest

diameter and the perpendicular diameter at the widest portion of the tumor. Measurements are to be taken from the barium swallow.

11.1.2 This is a clinical trial in which it is assumed that the majority of patients will respond to therapy. The goal of therapy is cure (complete response, histologically documented) and the endpoint for each patient on the study will be evidence of the following:

11.1.2.1 If twelve weeks after all therapy is completed tumor remains in the esophagus as proven by biopsy, then the patient has failed therapy but continues to be followed for survival.

11.1.2.2 Recurrent cancer in either a local area or distant area. Biopsy proof of recurrence should be sought but doubtless evidence such as hypercalcemia with new lesions on bone scan without evidence of new primary tumor will be accepted as a failure to therapy and the patient taken off study but followed for survival.

11.1.2.3 Death due to any cause.

11.1.3 Patients with no evidence of tumor upon esophagoscopy and esophagram of the esophagus after therapy will be considered "response to therapy" and will be followed until relapse or death.

11.1.4 There is no definition of partial or complete response for this clinical trial. Those without cancer visable via esophagoscopy and esophagram have responded to therapy. Those with histologically confirmed tumor post-treatment via re-biopsy have "failed therapy".

12.0 DATA COLLECTION

All material with the exception of Dosimetry and Initial Medical Oncology data will be sent to the responsible Cooperative Group according to the following schedule and then forwarded onto RTOG Headquarters. Preliminary and final dosimetry material and the Medical Oncology Treatment Planning Form must be sent directly to RTOG Headquarters, 1101 Market Street, 14th Floor, Philadelphia, PA 19107. All dosimetry material (films, etc.) must be identified by the use of labels available from your Cooperative Group.

Data	Schedule
Initial Evaluation Form (I1)<br>Diagnostic Pathology Report (P1)	Within one week of randomization
Operative Report (S2)<br>Surgical Path Report (S5)	Within 2 weeks of "salvage surgery"
Preliminary Dosimetry Information:<br>Prescription (T5), Central Axis Calculation (T4), Localization Film (T3)	Within one week of commencement of RT
* Medical Oncology Treatment Planning Form (M2)	One week after randomization
* Chemotherapy Flow Sheet (M1)	Within 2 weeks after completion of each course of chemotherapy
Radiotherapy Form (T1)<br>Final Dosimetry Information:<br>Treatment Sheets (T5),<br>Isodose Computations (T6)	At completion of RT
Follow-up Assessment Form (F1)	At completion of radiation, and/or chemotherapy (if applicable) every 3 months through year 2; then every 6 months through year 5; then yearly thereafter and at death.

Autopsy Report (D2) If done

* When assigned chemotherapy

13.0 STATISTICAL CONSIDERATIONS (Amended 11/11/88, 5/14/90)

13.1 Study Endpoints

The primary objective for this study is to determine if the addition of chemotherapy to radiation therapy prolongs survival as compared to radiotherapy alone survival.

13.2 Sample Size

To ascertain an appropriate sample size, an estimate of percent survival is required. The survival experience in esophageal carcinoma patients has been shown in the literature (2) to vary widely, depending upon the population under study and the intent in treatment. The proposed study is to be done with curative intent. A number of different studies involving esophageal cancer patients similar to the target population for this protocol have yielded similar results when therapeutic radiation only was administered. A retrospective Scandinavian study (3) and two RTOG trials, 70-02 (13) and 81-11 (unpublished) all showed two year survival probabilities of around 10%. Thus, for this sample size determination, 10% is taken as the estimate of 2 year percent survival for the standard treatment of radiation therapy alone.

Although the alternative hypothesis for the statistical test is that of improved survival with the addition of chemotherapy, this regimen may prove to be rather toxic. The sample size, therefore, is to be based upon a two-tailed rather than a one-tailed-test, in order to take into account possible worse response due to toxicity. The following table of sample sizes is based upon the logrank test (two-sided) with a α = .05, 1-β = .80 and .90, and 20% and 25% improvement. Also incorporated into these sizes is an assumed dropout rate of 10%:

		Improvement	
		20%	25%
Power	.80	112	81
	.90	150	109

13.3 Patient Accrual

Since accrual to the pilot study has averaged about 21 cases/year, and with group-wide activation of this protocol, the anticipated annual accrual is 36 cases per year. The addition of participation by the other cooperative groups will add to the projected accrual. Given this accrual rate and above required sample sizes, the following times are projected for patient entry:

		Improvement	
		20%	25%
Power	.80	3.1 years	2.2 years
	.90	4.2 years	3.0 years

Assuring a high chance of detecting at least a 20% improvement in survival, the statistical power was set at .80 in order that eight out of ten times such differences will be identified. The accrual target of the study is 112 patients.

13.4 Randomization Schema

The treatment allocation will be one using a randomized permuted block within strata to balance for patient factors other than institution. Each patient is initially given a provisional treatment assignment and the difference in the number of assignments between the treatment options

is computed. If that difference is less than a prespecified value, then the provisional assignment becomes the actual treatment assignment fo the particular patient. If the difference exceeds the prespecified value, then the patient is assigned a treatment other than the provsional assignment. The assigned treatment is noted in the stratum.

The stratifying variables will be weight loss in the last 6 months (>=10 lbs vs. <10 lbs.), lesion size (>=5 cm vs. <5 cm), and histology (squamous vs. adenocarcinoma).

13.5 Analyses Plans

13.5.1 Interim analyses

Interim reports with statistical analyses will be prepared every six months until the initial paper reporting the treatment results has been submitted. In general, the interim reports will contain information about patient accrual, with a projected completion date for the accrual phase, data quality, compliance rate of treatment delivery to the protocol, distributions of important prognostic baseline variables and the frequencies and severity of the toxicities.

Interim analysis of response and survival data will be performed after data on 56 cases (50% of the required sample size) is available. If the survival on the CT+RT arm shows a highly significant improvement over the survival on the RT only arm (log-rank $p<.005$), the recommendation will be made to discontinue the RT arm. Conversely, if the survival on the CT+RT arm shows a highly significant decline from the survival on the RT arm (log-rank $p<.005$), the recommendation will be made to discontinue the CT+RT arm. If either arm is discontinued, the recommendation will be made to close the study. Otherwise case accrual will continue until the required sample size is satisfied.

Measures of treatment efficacy such as survival, will be reported in a blinded fashion only to the Study Chairman, Dr. Arnold Herskovic, the Site Chairman, and the Group Chairman, until all the required patients have been entered on study and completed their treatment.

13.5.2 Analysis for Reporting the Initial Treatment Results

An analysis of the survival data will be performed shortly after the study is closed and the initial follow-up data are available in all evaluable cases. Reporting of the results will be considered if they are significant at the $p<.006$ level (log-rank test).

Otherwise a major analysis will be undertaken when each patient has been potentially followed for a minimum of 12 months. This analyses will include tabulation of all cases entered, and any excluded from the analyses, the distribution of the important prognostic baseline variables and observed results with respect to the endpoints described in section 13.1. The significance level of .047 will be used in the final analysis to preserve an overall significance level of .05[14]

13.6 Early Stopping

At an interim analysis performed in May, 1990, the difference in survival curves was statistically significant at $P=.0045$ (log-rank test, 2 sided), and favored the RT and Chemotherapy arm. This was based on 90 cases who were eligible with some follow-up and represents 90/112 or 80% of originally required accrual. This meets the criterion for early stopping as specified in section 13.5.1 of the protocol.

After consultation with clinicians and statisticians at the NCI, the decision was made to suspend the randomization of the RT alone arm, and to continue assignment of all cases to the RT and Chemotherapy arm pending development of a pilot successor study.

14.0 ADDITIONAL TREATMENT

Patients who develop local or distant recurrence following therapy may be treated by any means considered appropriate by the responsible physician. However, such therapy must be reported and submission of follow-up data must continue.

REFERENCES

1. Earlam R, and Cunha-Melo, JR: Oesophageal Squamous Cell Carcinoma: I. A Critical Review of Surgery. Br J Surg 67:457-461, 1980.

2. Earlam R, and Cunha-Melo JR: Oesophageal Squamous Cell Carcinoma: II. A Critical Review of Radiotherapy. Br J Surg 67:457-461, 1980; Ref 102

3. Cedarquist C., Nielsen J., Berthelsen A, and Hansen HS: Cancer of the Oesophagus: II. Therapy and Outcome. Acta Chir Scan 144:233-240, 1978.

4. Parker E, and Gregorie HB: Carcinoma of the Esophagus: Long Term Results. JAMA 235(10):1018-1030, 1976.

5. Wilson SE, Phested WB, and Carey JS: Esophagogastrectomy versus Radiation Therapy for Mid-Esophageal Carcinoma. Ann Thor Surg 10:195-202, 1970.

6. Leichman L, Steiger Z, Seydel HG, Dindogru A, Kinzie J, Toben S, MacKenzie G, and Shell J: Pre-operative Chemotherapy + Radiation Therapy for Patients with Cancer of the Esophagus: A Potentially Curative Approach. J Clin Oncol (Accepted for February 1984).

7. Leichman L, Steiger Z, Seydel HG and Vaitkevicius VK: Combined Pre-operative chemotherapy and Radiation Therapy for Cancer of the Esophagus. Semi Oncol (Accepted)

8. Franklin R, Steiger Z, Vaishampayan G, et al: Combined Modality Therapy for Esophageal Squamous Cell Carcinoma. Cancer 51:1062-1071, 1983.

9. Moore GE, Bross IDJ, Ausman R, et al: Effects of 5-Fluorouracil (NSC-19893) in 389 Patients with Cancer. Can Chemo Rep 52:641-653, 1968.

10. Kelsen D, Hilaris B, Coonley C, et al: Cisplatin, Vindesine and Bleomycin Chemotherapy of Local-Regional and Advanced Esophageal Carcinoma. Am J Med 75:645-652, 1983.

11. Steiger A, Franklin R, Wilson W, et al: Complete Eradication of Squamous Cell Carcinoma of the Esophagus with Combined Chemotherapy and Radiotherapy. Am Surg 47;95-98, 1981.

12. Nabaya K. The use of Bleomycin in the Treatment of Carcinoma of the Esophagus. Gann Monogr. 19:177-186, 1976.

13. Keys, H, Hanley, J, Rulin, P, Brady, L, Marcial, V: Experimental approaches to esophageal carcinoma radiotherapy. Proc. of ASTR, Abs. 86. In J Rad Oncol Biol Phys 5 (2):119, 1979.

14. Fleming TR, Harrington DP, O'Brien PC: Designs for group sequential tests. Controlled Clin Trials 5:348-361, 1984.

APPENDIX I

RTOG 85-01
NCCTG 88-40-51
SWOG 85-98

SUGGESTED PATIENT CONSENT FORM

I understand that I am being asked to participate in a research study involving the use of radiation with chemotherapy and must give my permission to receive this treatment. This research study is being conducted by the Radiation Therapy Oncology Group and by ________________.

My physician(s) in Radiation and Medical Oncology have informed me of the nature of a new study utilizing radiation therapy with chemotherapy for the treatment of esophageal cancer.

I understand that I have cancer of the esophagus and treatment is recommended. The standard treatment for cancer of the esophagus is radiation sometimes with chemotherapy and sometimes surgery. At some institutions some patients are treated with surgery alone, but most of the patients are treated with radiation. The purpose of this research study is to determine the safety and effectiveness of adding chemotherapy to radiation in treating cancer of the esophagus. The patients treated in this program will receive not only radiation treatment, but chemotherapy treatment along with the radiation. The reason for this is that physicians who have treated patients with both radiation and chemotherapy have claimed those patients so treated may live longer with less chance of tumor recurring than those who receive radiation alone.

Chemotherapy is medicine, which in this program, will be given into my vein. Although it is not absolutely necessary, most patients who will receive chemotherapy will have to be in the hospital as the treatment is given slowly over a period of four days and repeated four times during the entire treatment.

If I agree to participate in this study. I will to receive the following treatment plan:

Radiation plus chemotherapy - The chemotherapy will begin on day 1 of radiation with cis-Platinum and 5-Fluorouracil, and again on days 29, 50 and 71

Radiation Therapy: Treatments will be daily for 5 days per week, Monday-Friday. The duration of treatment will be approximately 5 weeks.

Chemotherapy: I will be in the hospital for a minimum of 5 days during each chemotherapy course (a total of 4). Chemotherapy will begin on the same day as radiation therapy begins. The beginning drug combination will be cis-Platinum and 5-Fluorouracil. Cis-Platinum will be given through and intravenous catheter the morning after admission to the hospital. Because cis-Platinum can cause kidney damage, intravenous fluids are started the night before treatment to "flush" the kidneys. Mannitol, a drug which increases urine production is given intravenously just prior to and after receiving cis-Platinum. So that you may better assess my kidney function during this time, I will be asked to save all urine specimens and to keep a record of all fluids taken by mouth during the first 24-48 hours after cis-Platinum. The second medicine is called 5-Fluorouracil (5-FU). This medicine is more beneficial and less likely to cause side effects when it is given at a slow constant rate. This drug will be given over a 4-day span. I will be given an IV pole to carry the 5-FU so I need not be in bed. After the first treatment, doses of drugs may be changed to fit individual needs. both 5-FU and cis-Platinum may cause nausea and vomiting. In order to minimize these side effects, a medicine called Compazine will be given either as a pill, shot or suppository prior to chemotherapy and every 3-4 hours thereafter as needed.

Amended: 2/10/87
5/14/90

I also understand that the following pretreatment procedures will include, blood and urine studies, x-rays, audiogram (hearing test), biopsy of the tumor, and any other tests which my physician feels are necessary.

Side effects of the treatments are:

1. Radiation Therapy - May include reddening of the skin in the treatment area, tiredness, and sore throat causing difficulty with swallowing.

2. Chemotherapy
   a) 5-Fluorouracil (Fluorouracil) - the possible adverse effects of this drug are, nausea, vomiting, sore mouth, diarrhea, skin rash, reversible hair loss, increased skin pigmentation, and a depression of the bone marrow, the blood forming organ. The bone marrow when depressed leads to a depression of: white cells (important for fighting infection), of blood platelets (important to blood clotting) and of red cells or hemoglobin (transfusion may be required to correct this). If you become pregnant changes may occur in the sex cells that might produce abnormal development of the unborn child.
   b) Cis-platinum - possible side effects of this drug may include: nausea and vomiting, kidney impairment, hearing loss, and depression of the bone marrow. As with the 5-FU, bone marrow depression leads to a depression: of white cells (important for fighting infection), blood platelets (improtant for blood clotting) and red cells (may cause anemia). You will be watched closely for these side effects and appropriate intervention taken if necessary. Those with prior renal disease or hearing impairment may be more severely affected by these toxicities.

My doctor(s) will closely monitor my condition and in order for him/her to recognize and treat all these undesirable side effects early, repeated blood tests, x-rays and other history and physical examinations will be required.

As part of the evaluation of my therapy, I will permit my doctors, or their designated nurses to withdraw samples of my blood during the course of treatment. Possible side effects include minimal discomfort from veinpuncture and possible hematoma (black and blue marks), and rare instances of fainting. On the days blood is drawn, two or three samples may be required, each sample amounting to less than two teaspoons.

I understand the purpose of this study is to develop improved methods of treatment, but at the present time no definite statement can be made as to what extent my participation will be directly beneficial to me. I understand that other methods of treatment are available to my doctor and I need not participate in this study to remain under his/her care.

Participation in this study is voluntary. No payment for participation will be given. I understand that in the event of injury resulting from the research procedures, no compensation and no free medical care or reimbursement is offered by ____________________.

Emergency treatment is available at ____________________.

I voluntarily consent to undergo the above described treatments and understand the known possible effects or hazards that might occur in the course thereof, and further understand that not all side effects of these treatments are known. I further recognize that I may withdraw from the study at any time for any reason without any interference with the care I receive from my physicians.

I understand that a record of my progress while on the study will be kept in confidential form at ____________________.

and also in the computer file at headquarters of the Radiation Therapy Oncology Group. A qualified representative of the FDA (Food & Drug Administration) and of the NCI (National Cancer Institute) may inspect the records. Strict confidentiality will be enforced.

Upon signing this consent form, I will receive a signed copy. The doctor(s) involved with my care is available to answer any questions I may have concerning this research study. In case of a problem or an emergency I may reach Dr. ___________ at ________________.
_______________ can be reached at ____________ if I have any questions regarding my rights as a research subject.

By ______________________________ ______________________________
(signature of patients or person responsible for signing for patient) relationship to patient

Date ______________________________ ______________________________
Witness

Note: If there is anything that you do not understand about this explanation ask the doctor for the information.

PHYSICIAN'S STATEMENT

I have offered an opportunity for further explanation of this study to the individual whose signature appears on the above statement.

Signed ______________________________
Managing Physician

RTOG/EORTC Late Radiation Morbidity Scoring Scheme

ORGAN/TISSUE	0	GRADE 1	GRADE 2	GRADE 3	GRADE 4	GRADE 5
SKIN	None	Slight atrophy Pigmentation change Some hair loss	Patchy atrophy Moderate telangiectasia Total hair loss	Marked atrophy Gross telangiectasia	Ulceration	
SUBCUTANEOUS TISSUE	None	Slight induration (fibrosis) and loss of subcutaneous fat	Moderate fibrosis but asymptomatic/Slight field contracture/ Less than 10% linear reduction	Severe induration and loss of subcutaneous tissue Field contracture > 10% linear measurement	Necrosis	
MUCOUS MEMBRANES	None	Slight atrophy and dryness	Moderate atrophy and telangiectasia/ Little mucous	Marked atrophy with complete dryness/ Severe telangiectasia	Ulceration	
SALIVARY GLANDS	None	Slight dryness of mouth/Good response on stimulation	Moderate dryness of mouth/Poor response on stimulation	Complete dryness of mouth/No response on stimulation	Fibrosis	DEATH DIRECTLY RELATED TO RADIATION LATE EFFECT
SPINAL CORD	None	Mild L'Hermitte's syndrome	Severe L'Hermitte's syndrome	Objective neurological findings at or below cord level treated	Mono, para quadriplegia	
BRAIN	None	Mild headache/ Slight lethargy	Moderate headache/ Great lethargy	Severe headache Severe CNS dysfunction (partial loss of power or dyskinesia)	Seizures or paralysis/coma	
EYE	None	Asymptomatic cataract Minor corneal ulceration or keratitis	Symptomatic cataract Moderate corneal ulceration/Minor retinopathy or glaucoma	Severe keratitis Severe retinopathy or detachment/Severe glaucoma	Panophthalmitis Blindness	
LARYNX	None	Hoarseness/Slight arytenoid edema	Moderate arytenoid edema/Chondritis	Severe edema/ Severe chondritis	Necrosis	
LUNG	None	Asymptomatic or mild symptoms (dry cough) Slight radiographic appearances	Moderate symptomatic fibrosis or pneumonitis (severe cough)Low grade fever/Patchy radiographic appearances	Severe symptomatic fibrosis or pneumonitis Dense radiographic changes	Severe respiratory insufficiency/Continuous O2/Assisted ventilation	
HEART	None	Asymptomatic or mild symptoms/Transient T wave inversion and ST changes/Sinus tachycardia >110 (at rest)	Moderate angina on effort/Mild pericarditis/ Normal heart size/Persistent abnormality T wave and ST changes/ Low QRS	Severe angina/Pericardial effusion/Constrictive pericarditis/Moderate heart failure/Cardiac enlargement/ EKG abnormalities	Tamponade/Severe heart failure Severe constrictive pericarditis	
ESOPHAGUS	None	Mild fibrosis/Slight difficulty in swallowing solids/No pain on swallowing	Unable to take solid food normally/Swallowing semi-solid food/ Dilatation may be indicated	Severe fibrosis/Able to swallow only liquids/May have pain on swallowing Dilatation required	Necrosis/Perforation/Fistula	
SMALL/LARGE INTESTINE	None	Mild diarrhea/Mild cramping/Bowel movement 5 times daily Slight rectal discharge or bleeding	Moderate diarrhea and colic/Bowel movement >5 times daily/Excessive rectal mucus or intermittent bleeding	Obstruction or bleeding requiring surgery	Necrosis/Perforation/Fistula	
LIVER	None	Mild lassitude/Nausea dyspepsia/Slightly abnormal liver function	Moderate symptoms/Some abnormal liver function tests/Serum albumin nral	Disabling hepatitic insufficiency/Liver function tests grossly abnormal/Low albumin/Edema or ascites	Necrosis/Hepatic coma or encephalopathy	
KIDNEY	None	Transient albuminuria No hypertension/Mild impairment renal function/Urea 25-35 mg% Creatinine 1.5-2.0 mg% Creat. clearance > 75%	Persistent moderate albuminuria (2+)/Mild hypertension/No related anemia/Moderate impairment renal function/Urea > 36-60 mg% Creatinine 2.5-4.0 mg% Creatinine clearance (50-74%)	Severe albuminuria/Severe hypertension/Persistent anemia (< 100%) Severe renal failure/Urea >60 mg% Creatinine > 4.0 mg% Creatinine clearance < 50%	Malignant hypertension/Uremic coma Urea > 100%	
BLADDER	None	Slight epithelial atrophy/Minor telangiectasia (microscopic hematuria)	Moderate frequency/Generalized telangiectasia Intermittent macroscopic hematuria	Severe frequency & dysuria Severe generalized telangiectasia (often with petechiae) Frequent hematuria. Reduction in bladder capacity (< 150 cc)	Necrosis/Contracted bladder (capacity <100 cc) Severe hemorrhagic cystitis	
BONE	None	Asymptomatic/No growth retardation Reduced bone density	Moderate pain or tenderness/Growth retardation Irregular bone sclerosis	Severe pain or tenderness Complete arrest bone growth Dense bone sclerosis	Necrosis Spontaneous fracture	
JOINT	None	Mild joint stiffness Slight limitation of movement	Moderate stiffness/Intermittent or moderate joint pain/Moderate limitation of movement	Severe joint stiffness/Pain with severe limitation of of movement	Necrosis Complete fixation	

APPENDIX III

RTOG TOXICITY CRITERIA - Chemotherapy/Radiosensitizers/Radioprotectors (See instructions on reverse sid

		[0]	[1]	[2]	[3]	[4]
HEMATOLOGIC	a) BLOOD COUNT					
	WBC x 1000	=> 4.0	< 4.0 - 3.0	< 3.0 - 2.0	< 2.0 - 1.0	< 1.0
	Neutrophils x 1000	=> 1.9	< 1.9 - 1.5	< 1.5 - 1.0	< 1.0 - 0.5	< 0.5
	Platelets x 1000	=> 100	< 100 - 75	< 75 - 50	< 50 - 25	< 25
	Hemaglobin gm %	=> 11.0	< 11.0 - 9.5	< 9.5 - 7.5	< 7.5 - 5.0	-----
	Hematocrit %	=> 32.0	< 32. - 28.	-----	-----	-----
	b) CLINICAL	No change from baseline	-----	Symptoms of anemia	Requires immediate transfusion	-----
	c) HEMORRHAGE	None	Minimal	Moderate-elective transfusion	Requires immediate transfusion	Lifethreatening
	d) INFECTION	None	No active therapy	Requires active Rx	Bacteremia	Lifethreatening
GU	a) BUN mg %	<= 20.0	> 20 - 40	=> 41 - 60	-----	-----
	b) CREATININE	<= 1.3	> 1.3 - 2.0	=> 2.1 - 4.0	-----	-----
	c) CREAT. CLEARANCE	=> 50 ml/min	< 50-40 ml/min	< 40-30 ml/min	< 30-20 ml/min	< 20 ml/min
	d) PROTEINURIA	No change from baseline	1+	2+ - 3+	4+	-----
	e) HEMATURIA		Microscopic-Cult Neg.	Gross - Cult 0	Gross & Clots	With obstructive uropathy
	f) BLADDER		Mild cystitis(symptomatic but no medication required)	Moderate cystitis	Hemorrhagic cystitis	Cystectomy required
BLOOD PRESSURE	-----	No change from baseline	Drop 15 - 20% of baseline systolic	Drop 20-40% of baseline systolic	Drop 40% of baseline systolic or in presence of hypertension or coronary disease, drop below 90 systolic/requires Rx, but not including pressor	Pressor treatment required
HEPATIC	a) SGOT	< 1.5 x normal	1.5 - 2 x normal	2.1 - 5 x normal	-----	-----
	b) ALKALINE PHOSPH.	< 1.5 x normal	1.5 - 2 x normal	2.1 - 5 x normal	-----	-----
	c) BILIRUBIN	< 1.5 x normal	1.5 - 2 x normal	2.1 - 5 x normal	-----	-----
	d) CLINICAL	No change from baseline	-----	-----	Precoma	Hepatic coma
GASTRO-INTESTINAL	a) NAUSEA & VOMITING	No change from baseline	Nausea/no vomiting	Controllable < 6 times per day	Vomiting-intractable/ > 6/day despite medication	Severe and continuou vomiting/requires hospitalization,
	b) STOMATITIS/ORAL		Faint pinkish enanthema, soreness (no medication required)	Ulcerous or patchy mucositis requiring medication; can eat	Confluent mucositis &/or confluent ulceration, cannot eat	Necrosis, hemorrhage requires hospitalization
	c) DIARRHEA		Increase in frequency of bowel movments semisolid (no medication required)	Watery and frequent movements (3-6/day)	Watery and frequent movements(> 6/day despite medication	Hemorrhagic diarrhea requires hospitalization
PULMONARY	a) RADIOGRAPH CHANGES	No change from baseline	Minimal changes	-----	-----	-----
	b) PFT		25 - 50% decrease in Dco and/or VC	> 50% decrease in Dco &/or VC	-----	-----
	c) CLINICAL		Mild symptoms	Moderate symptoms, no specific Rx required	Severe Sx/intermittent O2/corticosteroid Rx initiated	Assisted vent or continuous O2
CARDIAC	-----	No change from baseline	St-T changes, sinus tachycardia > 110 at rest	Atrial arrhythmias Unifocal PVC's PEP/LVET >0.42	Mild CHF, multifocal PVC's pericarditis	Severe or refractory CHF/ventricular tachycardia/tamponad
SKIN	a) SKIN	No change from baseline	Slight or transient erythema or mild pigmentation; dry desquamation	Brisk erythema, vesiculation/predominant depigmentation/ mild fibrosis	Moist desquamation/ Telangiectasia/atrophy/severe symptomatic fibrosis	Necrosis/ulceration
	b) HAIR		Limited hair loss	Moderate hair loss (less than total)	Total hair loss	-----

		[0]	[1]	[2]	[3]	[4]
	a) REFLEX		Decreased DTR's	Absent DTR's	-----	-----
NEUROLOGIC	b) STRENGTH		-----	Detectable or moderate weakness	Severe weakness, paresis/cannot squat or sit up in bed unassisted	Paralysis Transverse myelitis
PERIPHERAL NEUROPATHY	c) SENSORY	No change from baseline	Objective sensory changes/mild paresthesia or hypesthesia or mild pain	Moderate pain/moderate paresthesia a) Some interference with activity; requires analgesics b) Interferes with sleep, despite analgesics	Severe pain/severe paresthesia/severe interference with daily functions	-----
	d) GI MOTILITY		Mild constipation	Moderate constipation	Severe obstipation manageable without surgery	Obstipation requirin[illegible] surgery
	e) OTHER		-----	-----	Bladder dysfunction	Respiratory dysfunction, 2 to weakness[illegible]
	a) MENTAL STATUS (mood, ideation, memory or consciousness)		Transient alteration &/or minimal lethargy	Alteration substantially affecting function; severe anxiety or hyperactivity	Alteration substantially affecting function => 50% of time or of function	Comatose
NEUROLOGIC	b) MOTOR PARESIS		Mild or transient	Substantially affects function, <50% decrement in baseline capabilities	Substantially affects function, => 50% decrement in baseline capabilities	Paralysis
	c) CEREBELLAR FUNCTION		Mild or transient alteration	Substantially affects function, <50% decrement in baseline capabilities	Substantially affects function, => 50% decrement in baseline capabilities	Confined to bed
[illegible]NTRAL [illegible]VOUS [illegible]STEM	d) SEIZURES	No change from baseline	-----	Transient or satisfactorily controlled by medical therapy	Seizure disorder not controlled by medical Rx	Status epilepticus
	e) MUSCLE DYSFUNCTION Muscle Strength		Subjective symptoms; no measurable weakness	Detectable or moderate weakness/mild muscle tenderness	Severe weakness/cannot squat or sit up in bed unassisted/moderate muscle tenderness	Paralysis
	Enzyme dysfunction SGOT		> Normal to <= 1.5 x normal	> 1.5 to 2 X normal	> 2 - 10 X normal	> 10 X normal
	CPK		> Normal to <= 1.5 x normal	> 2 to 4 X normal	> 4 - 10 X normal	> 10 X normal
ALLERGY	-----	None	Transient rash, drug fever <=38C/<=100.4F	Urticaria/drug fever > 38C (> 100.4F)	Serum sickness/bronchospasm/requires parenteral medication	Anaphylaxis
FEVER	-----	None	>37.5 - 38C/<=100.4F	> 38C (> 100.4F)	> 40C (> 104F)	Fever with hypotension
LOCAL TOX	-----	No change from baseline	Pain	Pain and phlebitis	Ulceration	Necrosis
OTOTOXICITY	a) HEARING	No change from baseline*	=> 10 db change in 1 or more frequencies; difficulty only with faint speech	=> 10 db change in 2 or more frequencies; Frequent difficulty with faint speech	=> 15 db change in 2 or more frequencies; Frequent difficulty with loud speech	=> 20 db change in 2 or more frequencies; Understands only amplified speech
	b) TINNITUS	*Baseline: Threshold of patient's better ear at time of initial pretreatment test	Mild/transient	Moderate/transient or mild/persistent	Severe/transient or moderate/persistent	Severe/persistent

INSTRUCTIONS

1. Toxicity grade should reflect the most severe degree occurring during the evaluated period, not an average

When two criteria are available for similar toxicities, the one resulting in the more severe grade should be used.

3. Toxicity grade = 5 if that toxicity caused the death of the patient.

4. Refer to detailed toxicity guidelines or to study chairman for toxicity not covered on this table.

5. The evaluator must attempt to discriminate between disease/treatment and related signs/symptoms.

6. An accurate baseline prior to commencement of therapy is necessa

Acute Radiation Morbidity Scoring Criteria

	[0]	[1]	[2]	[3]	[4]
SKIN	No change over baseline	Follicular, faint or dull erythema/epilation/dry desquamation/decreased sweating	Tender or bright erythema, patchy moist desquamation/moderate edema	Confluent, moist desquamation other than skin folds, pitting edema	Ulceration, hemorrhage, necrosis
MUCOUS MEMBRANE	No change over baseline	Erythema/may experience mild pain not requiring analgesic	Patchy mucositis which may produce an inflammatory serosanguinous discharge/may experience moderate pain requiring analgesic	Confluent fibrinous mucositis/may include severe pain requiring narcotic	Ulceration, hemorrhage or necrosis
EYE	No change	Mild conjunctivitis with or without scleral injection/increased tearing	Moderate conjunctivitis with or without keratitis requiring steroids &/or antibiotics/dry eye requiring artificial tears/iritis with photophobia	Severe keratitis with corneal ulceration/objective decrease in visual acuity or in visual fields/acute glaucoma/panopthalmitis	Loss of vision (unilateral or bilateral)
EAR	No change over baseline	Mild external otitis with erythema, pruritis, secondary to dry desquamation not requiring medication. Audiogram unchanged from baseline.	Moderate external otitis requiring topical medication/serous otitis medius/hypoacusis on testing only	Severe external otitis with discharge or moist desquamation/symptomatic hypoacusis/tinnitus, not drug related	Deafness
SALIVARY GLAND	No change over baseline	Mild mouth dryness/slightly thickened saliva/may have slightly altered taste such as metallic taste/these changes not reflected in alteration in baseline feeding behavior, such as increased use of liquids with meals	Moderate dryness/thick sticky saliva/markedly altered taste	Complete dryness	Acute salivary gland necrosis
PHARYNX & ESOPHAGUS	No change over baseline	Mild dysphagia or odynophagia/may require topical anesthetic or non-narcotic analgesics/may require soft diet	Moderate dysphagia or odynophagia/may require narcotic analgesics/may require puree or liquid diet	Severe dysphagia or odynophagia with dehydration or weight loss (>15% from pretreatment baseline) requiring N-G feeding tube, I.V. fluids, or hyperalimentation	Complete obstruction, ulceration, perforation, fistula
LARYNX	No change over baseline	Mild or intermittent hoarseness/cough not requiring antitussive/erythema of mucosa	Persistent hoarseness but able to vocalize/referred ear pain, sore throat, patchy fibrinous exudate or mild arytenoid edema not requiring narcotic/cough requiring antitussive	Whispered speech, throat pain or referred ear pain requiring narcotic/confluent fibrinous exudate, marked arytenoid edema	Marked dyspnea, stridor or hemoptysis with tracheostomy or intubation necessary
UPPER G.I.	No change	Anorexia with <=5% weight loss from pretreatment baseline/nausea not requiring antiemetics/abdominal discomfort not requiring parasympatholytic drugs or analgesics	Anorexia with <=15% weight loss from pretreatment baseline/nausea &/or vomiting requiring antiemetics/abdominal pain requiring analgesics	Anorexia with >15% wt. loss from pretreatment baseline or requiring N-G tube or parenteral support. Nausea &/or vomiting requiring N-G tube or parenteral support/abdominal pain, severe despite medication/hematemesis or melena/abdominal distention (flat plate radiograph demonstrated distended bowel loops)	Ileus, subacute or acute obstruction, perforation, GI bleeding requiring transfusion/abdominal pain requiring tube decompression or bowel diversion
LOWER G.I. INCLUDING PELVIS	No change	Increased frequency or change in quality of bowel habits not requiring medication/rectal discomfort not requiring analgesics	Diarrhea requiring parasympatholytic drugs (e.g. Lomotil)/mucous discharge not necessitating sanitary pads/rectal or abdominal pain requiring analgesics	Diarrhea requiring parenteral support/severe mucous or blood discharge necessitating sanitary pads/abdominal distention (flat plate radiograph demonstrates distended bowel loops)	Acute or subacute obstruction, fistula or perforation GI bleeding requiring transfusion abdominal pain or tenesmus requiring tube decompression or bowel diversion
LUNG	No change	Mild symptoms of dry cough or dyspnea on exertion	Persistent cough requiring narcotic, antitussive agents/dyspnea with minimal effort but not at rest	Severe cough unresponsive to narcotic antitussive agent or dyspnea at rest/clinical or radiologic evidence of acute pneumonitis/intermittent O2 or steroids may be required	Severe respiratory insufficiency/continuous oxygen or assisted ventilation
GENITOURINARY	No change	Frequency of urination or nocturia twice pretreatment habit/dysuria, urgency not requiring medication	Frequency of urination or nocturia which is less frequent than every hour. Dysuria, urgency, bladder spasm requiring local anesthetic (e.g. Pyridium)	Frequency with urgency and nocturia hourly or more frequently/dysuria, pelvis pain or bladder spasm requiring regular, frequent narcotic/Gross hematuria with/without clot passage	Hematuria requiring transfusion/acute bladder obstruction not secondary to clot passage, ulceration or necrosis
HEART	No change over baseline	Asymptomatic but objective evidence of EKG changes or pericardial abnormalities without evidence of other heart disease	Symptomatic with EKG changes and radiologic findings of congestive heart failure or pericardial disease/no specific treatment required	Congestive heart failure, angina pectoris, pericardial disease responding to therapy	Congestive heart failure, angina pectoris, pericardial disease, arrhythmias not responsive to non-surgical measures
CNS	No change	Fully functional status (i.e.. able to work) with minor neurologic findings, no medication needed	Neurologic findings present sufficient to require home care/nursing assistance may be required/meds. including steroids/anti-seizure agents may be required.	Neurologic findings requiring hospitalization for initial management	Serious neurologic impairment which includes paralysis, coma or seizures >3 per week despite medication/hospitalization required
HEMATOLOGIC WBC (X 1000)	=>4.5	3.0 - <4.5	2.0 - <3.0	1.0 - <2.0	<1.0
PLATELETS (X 1000)	>130	90 - <130	50 - <90	25 - <50	<25 or spontaneous bleeding
NEUTROPHILS (X 1000)	=>1.9	1.5 - <1.9	1.0 -<1.5	0.5 - <1.0	<0.5 or sepsis
HEMOGLOBIN (GM %)	>11	9.5 - 11	<9.5	Packed cell transfusion required	--
HEMATOCRIT (%)	=>32	28 - <32	28	Packed cell transfusion required	--

GUIDELINES

The acute morbidity criteria are used to score/grade toxicity from radiation therapy. The criteria are relevant from day 1, the commencement of therapy, through day 90. Thereafter, the EORTC/RTOG Criteria for Late Effects are to be utilized.

The evaluator must attempt to discriminate between disease and treatment related signs and symptoms.

An accurate baseline evaluation prior to commencement of therapy is necessary.

All toxicities grade 3, 4 or 5* must be verified by the Principal Investigator.

* ANY TOXICITY WHICH CAUSED DEATH IS GRADED 5.

APPENDIX V

PRE-TREATMENT INTRATHORACIC ESOPHAGUS STAGING SYSTEM*

Protocol __________ Case # ___________ Inst. # _______ Date ___________

Histology

[ ] Squamous cell ___________
[ ] Other________(specify)________

Site Specific Information

Length of tumor ________________ cm
[ ] Encircles esophagus
[ ] Evidence of obstruction
[ ] Extraesophageal extension
[ ] Nerve involvement
[ ] Tracheobronchial tree
[ ] Caval obstruction
[ ] Pleural effusion

[ ] Mediastinal widening (not necessarily evidence of extra-esophageal spread)
[ ] Other ________ specify

Type of Lesion

[ ] polypoid
[ ] ulcerating
[ ] infiltrating

Tumor Distance from Incisors

[ ] Cervical 18 cm
[ ] Upper thoracic 18-30 cm
[ ] Lower thoracic 30 cm

Primary Tumor (T)*

[ ] T0 No demonstrable tumor in the esophagus
[ ] Tis Carcinoma in situ
[ ] T1 A tumor that involves 5 cm or less of esophageal length, that produces no obstruction, and that has no circumferential involvement and no extraesophageal spread
[ ] T2 A tumor that involves more than 5 cm of esophageal length without extraesophageal spread or a tumor of any size that produces obstruction or that involves the entire circumference but without extra-esophageal spread
[ ] T3 Any tumor with evidence of extra-esophageal spread

* Include information gained from esophagram, endoscopy, & CT scan

Regional Lymph Nodes (N) for thoracic esophagus are adjacent mediastinal

[ ] NX Minimum requirements to assess the regional nodes cannot be met
[ ] N0 No evidence of regional node involvement per CT
[ ] N1 evidence of mediastinal node involvement
[ ] A unilateral
[ ] B bilateral

Distant Metastasis (M)

[ ] MX Minimum requirements to assess the presence of distant metastasis cannot be met.
[ ] M0 No (known) distant
[ ] M1**Distant metastasis present (any nodes other than mediastinal and/or liver involvement) Specify ____________
[ ] M2 Involvement of other distant site ______________ specify

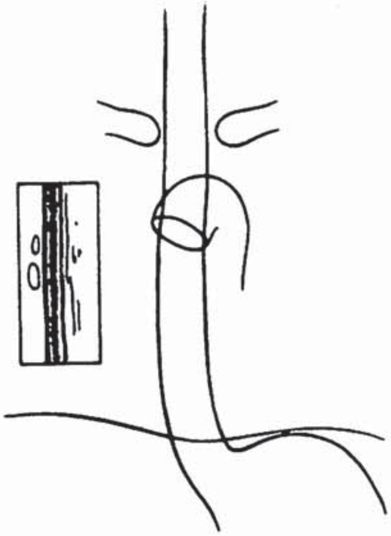

Length of tumor: _______ cm

Indicate on diagram primary tumor and regional nodes involved.

** Any cervical, scalene or abdominal LN are considered distant metastatic sites

Case# ____________

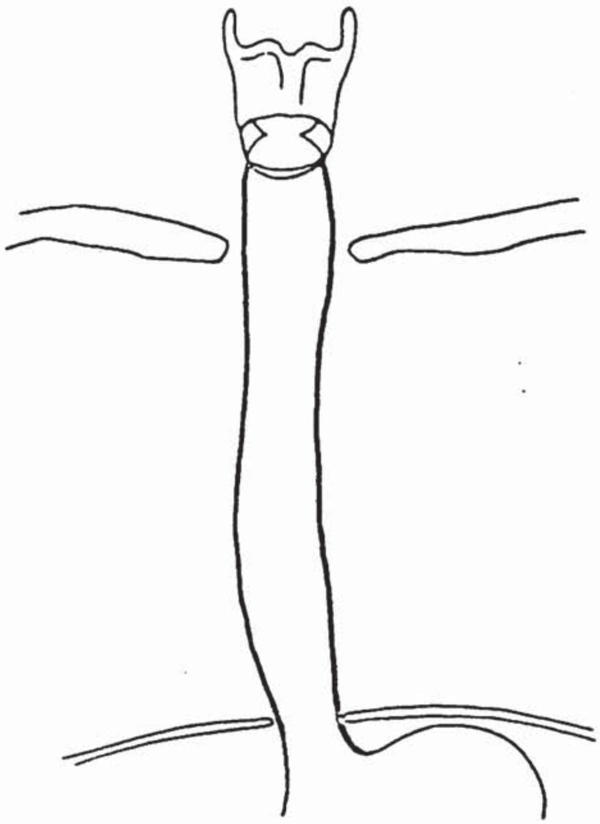

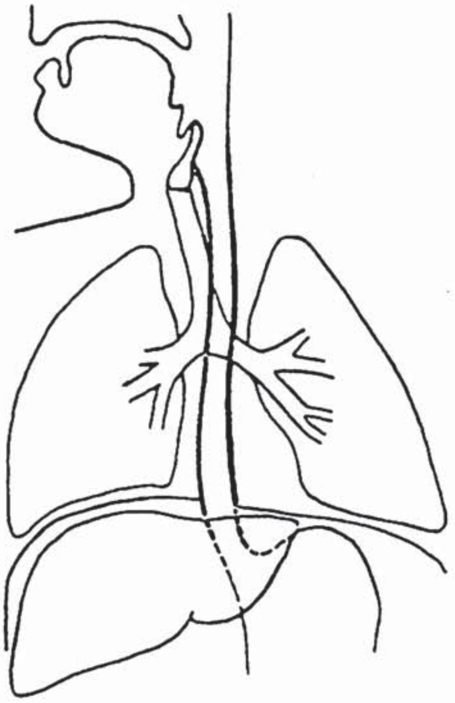

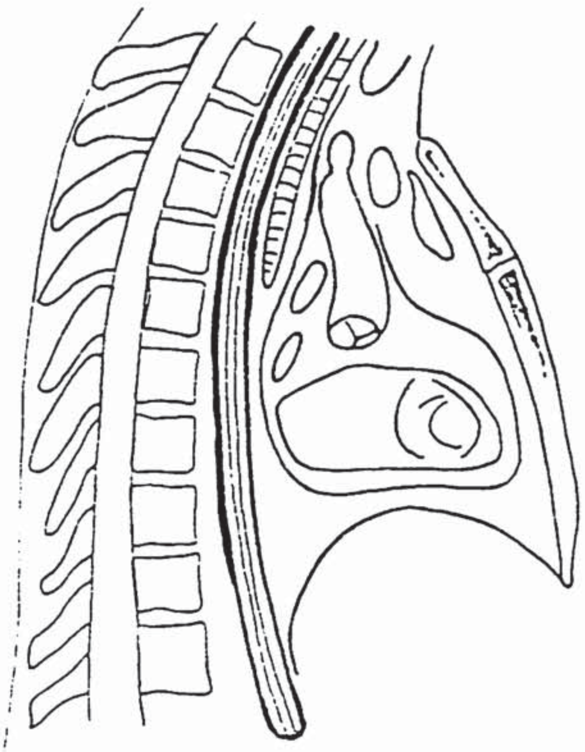

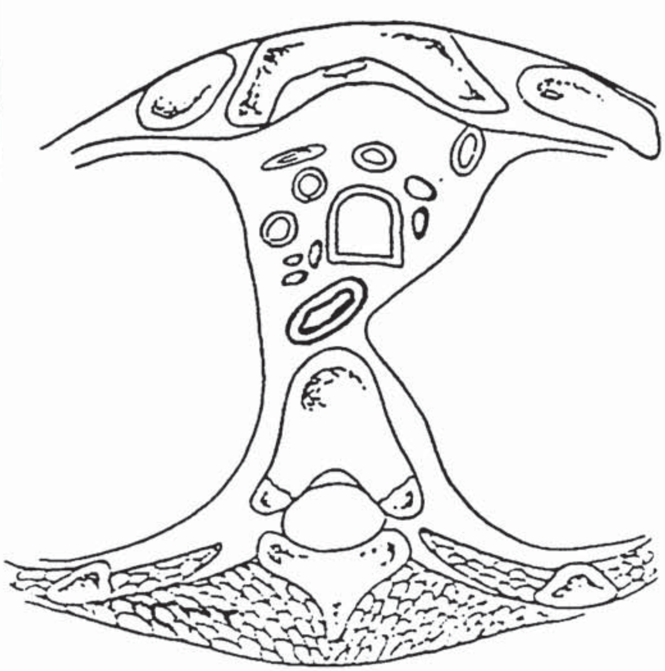

INTERGROUP PARTICIPATION IN RTOG STUDIES

GUIDELINES

RTOG STUDY 85-01

I. REGISTRATION:

RTOG will be responsible for all registration/randomizations. The procedure is:
-Each institution affiliated with a Cooperative Group will phone their group and supply the eligibility check information.
-The participating Cooperative Group will then telephone RTOG 215/574-3191 between 8:30 a.m. and 5:00 p.m. ET and supply the necessary eligibility and stratification information. RTOG will then assign a case number and treatment assignment. The participating Cooperative Group will then inform its member institution.
-RTOG will send a Confirmation of Registration and a Forms Due Calendar to the participating Cooperative Group for each case registered. The participating Group should then forward a copy of the calendar to the participating institution.

II. PROTOCOL DISTRIBUTION:

Each participating cooperative group is responsible for distribution of the protocol to its members. All protocol amendments will be sent by RTOG to each participating Group office for distribution to member institutions. All communication with NCI regarding this protocol will be routed through the RTOG.

III. INSTITUTIONAL PARTICIPATION:

It is the responsibility of each participating Cooperative Group to decide which of its member institutions may participate in this protocol. Each participating Cooperative Group must insure that IRB approval was obtained prior to accession of cases.

IV. CONFIRMATION/CALENDARS:

A Confirmation of Registration notice and a Data Collection Calendar is produced for each case registered and/or randomized. These will be distributed by RTOG to the appropriate cooperative group office for distribution to their members, if appropriate.

The form identification code which appears on the Calendars in the "key" columns is found on the form in the lower right corner.

You are expected to respond to each of the items listed either by submitting the item, by notifying us in writing that the item is not available or that the assessment was not done. The calendar may also list items which are not forms (CAT Scan reports, pathology reports) but are specific source documents. These items will be noted in the data collection section of the protocol but will not be listed on the Forms Package Index.

Additional items/forms may be required depending on events that occur e.g. if surgery was done a surgical report may be required. See the protocol for conditional requirements.

Unless specified otherwise, all patients are followed until death or termination of the study.

V. FORMS:

Forms packages may be obtained from the participating Cooperative Group office. Attached is a list (Forms Package Index) of all data collection forms used in the study, the toxicity criteria for this study, if applicable and a sample of the data collection forms.

The RTOG assigned case and study number must be recoded on all data items submitted. Except for material which requires rapid review (see below), data should be routed according to the mechanism set up by the participating Group. Generally the participating group will require forms to be routed through their office and they will send the forms to

American College of Radiology
Radiation Therapy Oncology Group - 14th Floor
1101 Market Street
Philadelphia, PA 19107

VI. LABELS

Preprinted labels are available for source document data items (radiographic reports, etc.) Supplied white labels are to be used for film identification.

The blank labels will be supplied to the participating Group for distribution to the individual institutions as patients are registered at RTOG.

When completing the labels, be specific when describing films, e.g.: "Pre op CT Brain Scan, "Large Photon Localization Film", "Follow-up Bone Scan", etc.

Data managers are advised to consult technical staff for assistance when labeling radiotherapy films. Correct film identification is the responsibility of the institutions and is essential to maintain efficient data flow.

VII. CANCELLATION/INELIGIBILITY:

Patients who are found to be ineligible subsequent to registration are to be followed according to plan unless you receive written instructions to the contrary.

Patients who receive no treatment whatsoever may be cancelled, however, written notification and an explanation must be received at RTOG Headquarters as soon as this has been determined. We must receive this notification not later than two weeks after registration. We will notify you of the determination made regarding the status of the case and instructions regarding subsequent data submission.

VI. RAPID REVIEW ITEMS:

Time critical data which requires rapid submission must be sent directly to RTOG (See address in Forms Section). These items are:

M2 - Medical Oncology Treatment Planning Form (if required by the Protocol)
T2 - Protocol Treatment Form
T3 - Photon Localization film (for all fields treated initially)
T4 - Photon dose calculations (for all fields treated initially)

IX. REQUEST FOR STUDY INFORMATION AND FORMS REQUEST:

Requests for additional information or clarification of data will be routed through the participating Cooperative Group office for distribution to the individual institution.

The memo requesting the additional information must be returned with the response. Responses should be returned according to the procedure used to submit data forms. You may receive reminders prompting response.

Periodically (generally three times per year) computer generated lists identifying delinquent material are prepared. These are routed by RTOG through the participating group for distribution.

X. **QUESTIONS REGARDING:

**Randomization/Registration**	Registration Secretary (215) 574-3191
**Pathology**	Registration Secretary (215) 574-3191
**Data/Eligibility/Treatment/ Adverse Reactions Data Management Procedures**	Data Management (215) 574-3214
**Protocols/Amendments**	Sharon Hartson (215) 574-3205
**Radiotherapy data items (films, radiographs, isodose summations, treatment records, scans, reports and calculations)**	Dosimetry Clerk (215) 574-3219

****If you are unable to reach the person noted, and your call is urgent, ask to speak to any data manager.**

XI. ADVERSE REACTIONS/AND TOXICITY

From Radiotherapy: Unusual toxicities, and all grade 4-5 toxicities are to be reported by telephone to RTOG Headquarters, the Group Chairman and to the Study Chairman. If the Chairman is unavailable, ask to speak to the Data Manager for this study.

From Investigational Agents: Are to be reported according to NCI guidelines. In addition, RTOG Headquarters and the Study Chairman are to receive notification as outlined by the NCI procedures, i.e. if telephone notification is necessary, RTOG and the Study Chairman must also be called.

Copies of all toxicity reports and forms submitted to NCI must be sent to RTOG Headquarters also.

From Commercial Drugs: Are to be reported according to NCI/FDA guidelines. A copy of the reports and forms submitted to FDA must be sent to RTOG.
