# Supplementary material for: Association of Radiotherapy Duration With Clinical Outcomes in Patients With Esophageal Cancer Treated in NRG Oncology Trials: A Secondary Analysis of NRG Oncology Randomized Clinical Trials
Source: JAMA Netw Open. 2023 Apr 21;6(4):e238504. doi: 10.1001/jamanetworkopen.2023.8504 (PMC10122174; doi:10.1001/jamanetworkopen.2023.8504)
Supplement: Supplement 3. — RTOG 9405 Protocol [file jamanetwopen-e238504-s003.pdf]

INT 0123
RADIATION THERAPY ONCOLOGY GROUP

RTOG 94-05

A PHASE III INTERGROUP RANDOMIZED COMPARISON OF COMBINED MODALITY THERAPY FOR CARCINOMA OF THE ESOPHAGUS: HIGH DOSE VS. CONVENTIONAL DOSE RADIATION THERAPY

ECOG ***(R9405)***
Thomas Pisansky, M.D.
Radiation Oncology
(507) 284-4655

Al Benson, M.D.
Medical Oncology
(312) 908-9412

Richard Feins, M.D.
Surgical Oncology
(716) 275-1509

NCCTG ***(91-40-51)***
James A. Martenson, M.D.
Radiation Oncology
(507) 284-4561

Paul Schaefer, M.D.
Medical Oncology
(419) 473-3561

RTOG *(94-05)*
(Coordinating Group)
Study Chairmen
Bruce Minsky, M.D.
Radiation Oncology
(212) 639-6817
FAX# (212) 639-8876

David Kelsen, M.D.
Medical Oncology
(212) 639-8470
FAX# (212) 794-7186

Robert Ginsberg, M.D.
Surgical Oncology
(212) 639-2806
FAX# (212) 639-2807

Todd Wasserman, M.D.
Quality of Life
(314) 362-8501
FAX# (314) 362-8521

Activation Date: June 12, 1995 Closure Date: July 1, 1999
Termination Date: June 30, 2010

Current Edition: July 1, 1999 (Includes Revisions 1-5)

This protocol was designed and developed by the Radiation Therapy Oncology Group *(RTOG)* of the American College of Radiology *(ACR)*. It is intended to be used only in conjunction with institution-specific IRB approval for study entry. No other use or reproduction is authorized by RTOG nor does RTOG assume any responsibility for unauthorized use of this protocol.

INDEX

Schema

Eligibility Check

1.0 Introduction

2.0 Objectives

3.0 Patient Selection

4.0 Pretreatment Evaluation

5.0 Registration

6.0 Radiation Therapy

7.0 Drug Treatment

8.0 Surgery

9.0 Other Therapies

10.0 Pathology

11.0 Patient Assessments

12.0 Data Collection

13.0 Statistical Considerations

References

Appendix I - Sample Consent Form
Appendix II - Karnofsky Performance Status
Appendix III - Staging System
Appendix IV - Toxicity Criteria
Appendix V - Adverse Reaction Reporting Guidelines
Appendix VI - Recommendations for Hydration
Appendix VII - General Intergroup Participation Instructions

INT 0123
RADIATION THERAPY ONCOLOGY GROUP

RTOG 94-05

A PHASE III INTERGROUP RANDOMIZED COMPARISON OF COMBINED MODALITY THERAPY FOR CARCINOMA OF THE ESOPHAGUS: HIGH DOSE VS. CONVENTIONAL DOSE RADIATION THERAPY

SCHEMA

STRATIFY

Weight Loss

1. ≥ 10%
2. < 10%

Lesion Size

1. ≤ 5 cm
2. > 5 cm

Histology

1. Squamous
2. Adenocarcinoma

RANDOMIZE

Arm 1:
HIGH DOSE RT
(Both chemo & RT to start on first day of week 1)

CDDP - 75 mg/m^2 - day 1 of wks 1 and 5; then repeat 4 weeks after the end of RT.

5-FU - 1000 mg/m^2 - days 1-4 of wks 1 and 5; then repeat 4 wks after the end of RT

RT - 1.8 Gy x 5 days/wk x 5.5 wks *(50.4 Gy in 28 fx)* followed by a conedown of 1.8 Gy x 5 days/wks x 1.5 wks *(14.4. Gy in 8 fx)* to a total dose of 64.8 Gy in 36 fractions *(start first day of week 1)*

Arm 2:
CONVENTIONAL DOSE RT
(Both chemo & RT to start first day of week 1)

CDDP - 75 mg/m^2 - day 1 of wks 1 and 5: then repeat 4 weeks after the end of RT.

5-FU - 1000 mg/m^2 - days 1-4 of wks 1 and 5; then repeat 4 wks after the end of RT

RT - 1.8 Gy x 5 days/wk x 5.5 wks *(50.4 Gy in 28 fx)* *(to start first day of week 1)*

Eligibility *(See Section 3.0 for details)*

- No TE fistula
- T1-4, Nx, M0
- Must have abdominal CT
- No prior chemotherapy, chest RT
- No evidence of disseminated cancer
- Squamous cell or adenocarcinoma of the esophagus
- WBC ≥ 4,000/mm, platelets ≥ 150,000/mm, Hgb ≥ 10 gm%, creatinine ≤ upper normal limit.
- No tumor extension within 2 cm proximal to the stomach
- Karnofsky Performance Status *(KPS)* ≥ 60

ARM 1: ***HIGH DOSE RT***

WEEK:	1	2-4	5	6-7.5	7.5-11.5	1	5
RT	X XXXX	X XXXX	X XXXX	X XXXX	Rest		
CDDP X		X			X	X	
5-FU	X X X X		X X X X			XX X X	XX X X

ARM 2: ***CONVENTIONAL DOSE RT***

WEEK:	1	2-4	5-5.5	5.5-9.5	1	5
RT	X XXXX	X XXXX	X XXXX	Rest		
CDDP X		X		X	X	
5-FU	X X X X		X X X X		X X X X	X X X X

Required Sample Size: 298 **3/1/96**

RTOG Institution # ______________ **INT 0123**

RTOG 94-05/ECOG R9405/NCCTG 91-40-51 **<u>ELIGIBILITY CHECK</u> (9/8/98)**

RTOG Case # ______________ ***(page 1 of 2)***

Other Seq# ______________

_______(Y) 1. Does the patient have biopsy-proven squamous cell or adenocarcinoma of the esophagus?

________________________ Specify histology

_______(Y) 2. Is the tumor confined to the esophagus with no extension within 2 cm of the proximal stomach?

________________________ Specify lesion size *(cm)*

_______(N) 3. Is there evidence of distant disease?

_______(N) 4. Is there evidence of tracheal bronchial tree invasion or tracheal esophageal fistula?

_______(N) 5. Does the patient have multiple carcinomas of the esophagus?

_______(Y) 6. Did the patient have a CT of the abdomen?

_______(≥ 4) 7. What is the WBC *(x 1000)* ?

_______(≥ 150) 8. What are the platelets *(x 1000)*?

_______(≥ 10) 9. What is HGB?

_______(Y) 10. Was a serum creatinine and/or creatinine clearance done?

_______(Y/NA) 11. If the serum creatinine was done, are the results ≤ upper institutional normal?

_______(Y/NA) 12. If the creatinine clearance was done, are the results ≥ 65 cc min?

_______(Y) 13. Have the required studies in Section 4.1 been done within the protocol time frame?

_______(N) 14. Has the patient had prior chemotherapy or chest radiation?

_______(N) 15. Has the patient had a surgical resection of the tumor?

_______(Y) 16. Is the patient ≥ 18 years of age and the Karnofsky Performance Status ≥ 60?

_______(Y/N) 17. Has the patient had a previous malignancy other than curable non-melanoma skin cancer or cervical cancer *in-situ*?

_______(Y) If yes, has the patient been disease free ≥ 5 years?

RTOG Institution # ______________ **INT 0123**

RTOG 94-05/ECOG R9405/NCCTG 91-40-51 **ELIGIBILITY CHECK** **(9/8/98)**

RTOG Case # ______________ ***(page 2 of 2)***

Other Seq# ______________

_______(Y) 18. Has the patient had evaluations by the medical oncologist and the radiation oncologist?

_______(Y) 19. Has the patient signed a study-specific consent form?

_____________________ Patient's Name

_____________________ Verifying Physician

_____________________ Patient ID #

_____________________ Referring Institution # *(if different)*

_____________________ Percent Weight Loss *(≥ 10% vs. < 10%)*

_____________________ Medical Oncologist

_____________________ Birthdate

_____________________ Sex

_____________________ Race

_____________________ Social Security Number

_____________________ Zip Code *(9 digit if available)*

_____________________ Method of Payment

_____________________ Will any component of the patient's care be at a VA or military facility?

_____________________ Treatment Start Date *(must be within 14 days after randomization)*

_____________________ Treatment Assignment

Completed by _________________________________ Date ________________________

1.0 INTRODUCTION

1.1 Given the limited success of radiation therapy either as a primary modality or in the pre-operative or post-operative setting, a number of investigators had explored the use of systemic chemotherapy in conjunction with radiation therapy. There is good rationale for combining systemic chemotherapy with radiation therapy for the treatment of esophageal cancer. These include an objective response rate of 40-60% in patients with metastatic disease, the observation that most patients with esophageal cancer die of distant metastasis, and some of the active agents in esophageal cancer *(i.e., 5-FU, Cisplatin, Mitomycin C)* are radiation sensitizers.

1.2 Some investigators advocate surgery following pre-operative combined modality therapy.[1-7] However, it is unclear whether the addition of surgery following combined modality therapy is of benefit. In a non-randomized trial by Gill et al, patients received two cycles of 5-FU, Cisplatin, and radiation therapy.[8] Those patients who were not treated either palliatively or were medically inoperable underwent surgery. Therefore, the better prognostic patients were selected for surgery. The local failure and distant failure rates were higher in the patients who underwent surgery compared with those who did not undergo surgery however the differences did not reach statistical significance. A randomized trial of this approach is being performed at the University of Michigan.

1.3 The issue of histology further complicates the issue. There is an increasing incidence of adenocarcinoma of the esophagus compared with squamous cell carcinoma. In the series by Coia and associates,[9] patients with adenocarcinoma had improved survival. In contrast Gill et al.[8] and Forastiere and colleagues[5,6] reported that patients with squamous cell carcinoma had an improved survival compared with adenocarcinoma. Naunheim and associates report no survival difference with histology.[3] Until randomized trials are performed where patients are stratified by histology, this question cannot be adequately answered.

1.4 There are a number of single arm phase II trials of combined modality therapy alone for esophageal cancer. In the series from Coia and associates,[9] patients received 5-FU, Mitomycin C, and 60 Gy. John et al.[10] limited the radiation dose to 40-50 Gy. Radiation doses of up to 66 Gy following 3 cycles of CDDP and bleomycin have been used.[11]

1.5 The trial reported by Coia and associates is the only combined modality therapy trial in which patients with clinically early stage esophageal cancer *(stages I and II)* were treated and analyzed separately.[9] Combining clinical stages I and II, the local failure rate was 25%, the 5-year actuarial survival rate was 30%, and the 5-year actuarial local relapse free survival rate was 70%.

1.6 The series reported by John et al. includes 30 patients with clinical stages I-III disease and reported a similar local failure rate of 27%.[10] The 2-year actuarial survival rate was 29%.

1.7 In order to confirm these phase II data of combined modality therapy alone, four randomized trials comparing radiation therapy alone with combined modality therapy have been performed.[12-17] Unfortunately, in three of the trials, inadequate doses of systemic chemotherapy were delivered. For example, in the small trial from Araujo and colleagues,[12] patients received only one cycle of 5-FU, Mitomycin C and Bleomycin. In the EORTC trial reported by Roussel et al., subcutaneous Methotrexate was used.[14] In the Scandinavian trial reported by Hatlevoll and associates, patients received inadequate doses of chemotherapy *(Cisplatin 20 mg/m^2 and Bleomycin 10 mg/m^2 for a maximum of two cycles).*[15]

1.8 The only non-operative trial which was designed to deliver reasonable doses of systemic chemotherapy with concurrent radiation therapy was reported by Herskovic et al, from the RTOG *(RTOG 85-01)*[13]. In this trial, patients received four cycles of 5-FU *(1000 mg/m2 x 4 days)* and Cisplatin *(75 mg/m^2, day 1)*. It should be emphasized that cycles 1 and 2 of chemotherapy were delivered every four weeks whereas cycles 3 and 4 were delivered every three weeks. This dose intensification following the combined modality segment may explain, in part, why patients had difficulty completing all four cycles. Radiation therapy *(50 Gy)* was given concurrently with chemotherapy beginning day 1. A higher dose of radiation *(64 Gy)* was used in the radiation therapy control arm. At two years, patients who received combined modality therapy had a significant improvement in survival *(38% versus 10%)* as well as a significant decrease in local failure *(44% versus 65%)*, and distant failure *(12% versus 26%)*. With longer follow-up, the three-year actuarial survival of patients who received combined modality therapy was 31%. There were no 3 year survivors in the radiation therapy control arm.[17]

1.9 The ECOG performed a similar trial of radiation therapy alone versus combined modality therapy *(EST1282)*.[16] However, since patients had the option of surgery after 40 Gy, the results are more difficult to interpret. An interim analysis revealed a significant improvement in median survival *(14.9 months versus 9 months)* in patients who received combined modality therapy. Final results of this trial are pending.

1.10 Based on the positive results of the RTOG 85-01 trial, the conventional non-operative treatment for esophageal cancer is combined modality therapy rather than radiation therapy alone. However, despite the positive results of the RTOG 85-01 combined modality arm, the local failure rate was high *(44% at two years)* and the survival rate is modest *(31% at three years)*. New treatment programs which deliver more intensive therapy are needed.

1.11 A recently completed Intergroup phase II pilot trial *(ECOG PE289/RTOG 90-12)* attempted to intensify both the radiation as well as chemotherapy doses. Patients with non-operable squamous cell carcinoma of the esophagus received three cycles of neoadjuvant 5-FU/CDDP followed by an additional two cycles of concurrent 5-FU/CDDP and 64.8 Gy. Of the 46 patients entered, 5 *(11%)* had treatment related mortality *(unpublished results)*. Therefore, in patients with esophageal carcinoma selected for this non-operative approach, intensification of 5-FU/CDDP is not possible. Therefore, this phase II Intergroup pilot will not be brought into phase III studies.

1.12 There are preliminary data which suggest that patients with esophageal carcinoma are able to tolerate larger doses of radiation therapy. Calais et al. reported the results of 53 patients with unresectable esophageal carcinoma who received 5-FU/CDDP/Mitomycin-C and 65 Gy.[34] The full dose of radiation was able to be delivered in 96% of patients. The incidence of WHO grade 3+ toxicity was 30% and the overall two-year survival was 42%. In the ECOG PE289/RTOG 90-12 pilot, of the 31 patients who started radiation therapy, 29 finished. Two patients died due to nadir sepsis; one patient refused the last two treatments. Therefore, these preliminary data suggest that doses of radiation in the range of 65 Gy are tolerable.

1.13 Therefore since 1) the local failure rate with conventional 5-FU/Cisplatinum and 50 Gy is high, and 2) attempts to further intensify both the radiation dose plus chemotherapy dose were not successful, the present trial *(RTOG 94-05)* will attempt to further intensify the radiation therapy dose. The control arm *(Arm 2)* of this trial is a modification of the positive arm from RTOG 85-01. Minor modifications have been made. These include 1) using 1.8 Gy fractions to 50.4 Gy rather than 2 Gy fractions to 50 Gy, 2) treating with 5 cm proximal and distal margins rather than the whole esophagus, and 3) Cycle #3 of chemotherapy will not begin until 4 weeks following the completion of radiation therapy. The experimental arm *(Arm 1)* will be the same as Arm 2 except the radiation dose will be intensified to 64.8 Gy from 50.4 Gy.

1.14 In view of prior disappointing survival rates and intensification of treatment regimes utilizing combined modalities, consideration of how various treatments affect this study population in a variety of ways is warranted. Quality of life measurement augments morbidity and mortality evaluations and uniquely contributes to the "cost-benefit" ratio involved in assessments of these treatments.[22] Not only are quality of life endpoints useful in therapy evaluations and treatment comparisons, but also in providing guidance in future clinical decisions.[23] There has not been any prior systemic study of the esophageal patient's quality of life who is undergoing therapy similar to that used in this investigation. Therefore, due to differences in the duration and intensity of the two treatment arms in this protocol, an appraisal of patients' quality of life complements outcome and toxicity analyses.

Upon general consensus, health related quality of life is viewed as a multidimensional construct, best assessed prospectively via the patient's perspective. Core quality of life domains within a health-oriented framework include, at the minimum, physical functioning, disease-related and treatment-related symptoms, social functioning, and psychologic functioning.[24]

A review of the medical and nursing English literature over the last decade revealed few investigations of the "quality" of esophageal patients' lives. Primarily these have focused upon post-treatment measurement of one dimension of the construct, including pain assessments or dysphagia parameters.[25,26,27,28] While disturbance in the swallowing mechanism is the most commonly reported complaint, other reported disease or treatment concerns include esophagitis, stomatitis, myelosuppression, weight loss, pain, hoarseness, dyspnea, cough and nausea.[13,18,27,29,30]

Two retrospective investigations of esophageal cancer patients' records apparently relied solely on reviewer or physician appraisal of patient's quality of life by assessing for dysphagia, pain, performance status or

autonomous physical function, and weight.[29,30] Bluett et al.[30] described 263 patients' post-treatment quality of life as good, fair or poor dependent upon degree of each item while Flores'[29] compared pre- and post-treatment assessments in 483 patients. Sugimachi et al.[31] performed prospective, subjective and objective quality of life assessments with 64 post-operative esophageal patients. Items assessed included food tolerance, occupational ability, body weight, and performance status. None of the reviewed studies provided an esophageal site-specific assessment scale with published validity or reliability analyses.

Neither was a quality of life measure incorporating the minimally recommended domains or tool specific for esophageal patients undergoing combined modality treatment found. An appreciation of the esophageal patient as a whole, with specific disease-related concerns, undergoing multi-modality treatment requires the use of a multi-dimensional, disease and treatment-specific quality of life tool to address the unique impact upon this patient's sense of well-being and satisfaction with life. In this study, quality of life measurement using a cancer-specific instrument with demonstrated adequate reliability and validity, the FACT-H&N *(version 2)*[32], will be amended by 17 supplemental site and treatment-specific items to provide a sufficient balance of measurement sensitivity, specificity, and generalizability.[31]

2.0 OBJECTIVES

2.1 To compare, using a prospective controlled randomized study design, the outcomes *(survival and failure patterns)* of therapy with conventional dose RT *(50.4 Gy)* and 5-FU/CDDP versus high dose RT *(64.8 Gy)* and 5-FU/CDDP combined chemotherapy in patients with esophageal cancer.

2.2 To compare the tolerance and overall quality of life in patients receiving these therapies. Quality of Life at the end of all therapy will be specifically examined.

3.0 PATIENT SELECTION

3.1 Eligibility Criteria (3/1/96)

3.1.1 The patient must have biopsy-proven primary *(non-recurrent)* squamous cell or adenocarcinoma of the esophagus. Disease must be entirely confined to the esophagus, and peri-esophageal soft tissue. There must be no tumor extension within 2 cm proximal to the stomach.

3.1.2 There must be no evidence of disseminated cancer *(patients must have clinical stage T1-4 Nx, M0, [Appendix III] excluding those patients with a TE fistula).*

3.1.3 Pre-entry CT of abdomen is required. A CT of the liver suspicious for metastatic disease must be followed with a negative liver biopsy before a patient can be considered eligible to enter the study.

3.1.3.1 Patients with enlarged *(≥ 1.5 cm)* retroperitoneal or celiac lymph nodes *(M1 disease)* that can be seen on CT should have biopsy proof of "no cancer" in these nodes before study entry; otherwise they are considered ineligible. Any method of biopsy is allowed.

3.1.4 Age ≥ 18 years and Karnofsky Performance Status ≥ 60 *(Appendix II).*

3.1.5 WBC must be ≥ 4,000/mm, platelet count must be ≥ 150,000/mm., and Hgb ≥ 10 gm%.

3.1.6 Serum creatinine ≤ institutional upper normal limit and/or creatinine clearance ≥ 65cc/min. If both are done, both must be within these limits.

3.1.7 Bronchoscopy of tracheal bronchial tree is required, in order to exclude TE fistula if the lesion is < 30 cm from the incisors.

3.1.8 Patient must not have had a second malignancy, other than curable non-melanoma skin cancer or cervical cancer *in situ*, unless disease free for ≥ 5 years. May not have received chest radiation or chemotherapy.

3.1.9 Study-specific signed informed consent.

3.1.10 Medical and Radiation Oncologist Evaluations.

3.1.11 Biopsy of S/C node if clinically or radiographically positive. Patients with cervical primaries with positive S/C lymph nodes *(N1)* are eligible.

3.2 Ineligibility Criteria

3.2.1 Failure to perform the studies specified in Section 4.1 with findings consistent with the criteria noted.

3.2.2 Patients without biopsy proven squamous cell or adenocarcinoma of the esophagus.

3.2.3 Evidence of disseminated cancer *(M1)*

3.2.4 WBC < 4,000/mm3, platelets < 150,000/mm^3, Hgb < 10 gm %.

3.2.5 Failure to sign a study-specific informed consent form prior to randomization.

3.2.6 Prior chemotherapy, chest radiation, or surgical resection of tumor.

3.2.7 Biopsy proven invasion of the tracheal bronchial tree or tracheal esophageal *(TE)* fistula shown by bronchoscopy or extension of the tumor within 2 cm proximal from the stomach.

3.2.8 Patients with non cervical primaries with positive S/C lymph nodes *(M1)* are ineligible.

3.2.9 No prior *(or concurrent with protocol treatment)* growth factor *(GSF)* administration is permitted.

3.2.10 Patients with multiple carcinomas of the esophagus are ineligible.
3.2.11 Pregnant or lactating women.

4.0 PRETREATMENT EVALUATION

4.1 Required Evaluations (3/1/96)

4.1.1 Complete history and physical exam including weight with an assessment of the patient's performance status *(KPS).*

4.1.2 The patient must be evaluated by a Medical and Radiation Oncologist prior to study entry.

4.1.3 Laboratory Studies *(within 2 weeks prior to randomization)*
- CBC, platelets
- SMA-12 *(serum creatinine, electrolytes, SGOT, LDH, alkaline phosphatase, total bilirubin, total protein, albumin, uric acid, inorganic phosphorous, calcium).*
- Creatinine clearance *(optional)*

4.1.4 Imaging Studies*(within 4 weeks prior to randomization)*
- CT Scan of the Chest and Abdomen
- Esophagram
- Chest X-ray

4.1.5 Bronchoscopy of tracheal bronchial tree is required if the lesion is < 30 cm from the incisors to exclude TE fistula.

4.1.6 PFTs *(Spirometry and diffusing capacity of the lung for carbomonoxide [DLCO]) (optional).*

4.1.7 Biopsy of S/C node if clinically or radiographically positive.

4.1.8 EKG; bone scan *(if alkaline phosphatase is elevated ≥ 1 .5 x normal)*

4.1.9 Barium Contrast UGI

4.1.10 *Quality of Life Baseline Assessments*
The FACT tool is available in English and Spanish. If the patient is unable to complete these forms due to language barriers, this will not affect enrollment in the study treatment but must be appropriately documented *(See Section 11.6.4).* All other patients must complete the FACT tool.

4.1.11 *Nutritional Assessment*
Patients should be ingesting either more than 1.5 x their Basal Energy Expenditure *(BEE)* as measured by the Harris-Benedict equation or more than 1,000 calories per square meter of body surface area *(1700 calories for the average 1.7 meter individual).* If the patient is not able to ingest this amount by mouth or tube feeding, then it is strongly recommended that the patient be hyperalimented intravenously. The i.v. hyperalimented patient must receive, as a minimum, 1.75 x the BEE or 1200 calories per square of body surface area but no more than 2.25 x the BEE or 1600 calories per meter square of body surface area unless the patient can be shown to be hypometabolic.

4.1.11.1 Patients should be instructed about food intake during treatment. Instructions should include recommending the avoidance of irritants *(including alcohol, citrus/acidic foods, sharp foods, or foods with extreme temperatures).* Documentation of any nutritional intervention, including oral high protein nutritional supplements, feeding tubes, and parenteral or enteral nutrition is required.

4.1.11.2 Harris-Benedit Equation to Measure BEE

Men
66.4730 + *(13.7516 x wt in kg)* + *(5.0033 x ht in cm)* - *(6.75 x age)*

Women
655.0955 + *(9.5634 x wt in kg)* + *(1.8496 x ht in cm)* - *(4.6756 x age)*

Daily Caloric Requirement = BEE x 1.75

Daily Protein Requirement = $\frac{\text{Caloric Requirement}}{150} \times 6.25$

4.2 Optional Evaluations

4.2.1 Bilateral audiogram

4.2.2 Endoscopic ultrasound *(U/S)*

5.0 REGISTRATION PROCEDURES

5.1 Randomization

It is the responsibility of each participating Cooperative Group to decide which of its member institutions may participate in this protocol. Each participating Cooperative Group must ensure that IRB approval was obtained prior to accession of cases. Patients who meet the eligibility criteria in Section 3.0, sign the consent form, and pass the pretreatment evaluation, will be entered into the study prior to any protocol therapy. Member institutions will phone their respective Cooperative Group headquarters Mondays through Fridays. The following information will be required at the time of patient entry:

- Institution's Name and RTOG Institution Identification Number *(when calling RTOG)*
- Patient's name *(or initials)* and ID Number
- Verifying Physician's Name
- Eligibility Criteria Information
- Stratification Information
- IRB Approval Date
- Name of Medical and Radiation Oncologists
- Demographic Data
- Treatment Start Date ***(must be within 14 days after randomization)***

5.2
- **NCCTG** *(507)* 284-4130, 8:00 am - 4:30 pm, Central Time
- **RTOG** *(215)* 574-3191, 8:30 am - 5:00 pm, Eastern Time.
- **ECOG** *(617)* 632-2022, 8:00 am - 4:30 pm, Eastern Time.

A signed HHS 310 Form, a copy of the Institution's IRB-approved informed consent document, and written justification for any changes made to the informed consent in this protocol must be on file at the ECOG Operations Office before an ECOG institution may enter patients. The signed HHS 310, institution's informed consent, and investigator's justification for changes will be submitted to the following address:

ECOG
Attn: IRB
303 Boylston Street
Brookline, MA 02146-7648
FAX 617/632-2990

Patients must not start protocol treatment prior to registration. The eligibility checklist should be completed and signed prior to calling for registration. To register eligible patients on study, ECOG institutions will telephone the Central Randomization Desk at the ECOG Statistical Center Data Management Office at *(617)* 632-2022. Monday through Friday, 8:00 a.m. - 4:30 p.m., Eastern Standard Time. ECOG members must not call RTOG directly. The following information will be requested: a) Protocol Number; b) Investigator Identification (*institution name and/or affiliate, and investigator's name)*; c) Patient Identification *(Patient's name or initials and hospital chart number)* patient's Social Security number; and Patient Demographics *(sex, birth, date, race, nine-digit zip code, and method of payment)*; d) Eligibility verification, and e) any additional information listed in Section 5.1. Patients must meet all of the eligibility requirements listed in Section 3.0. The randomization specialist will verify eligibility by asking questions from the Eligibility Checklist.

If a patient does not receive any protocol therapy, the patient may be canceled. Reasons for cancellation should be noted on the data forms and submitted to the ECOG Statistical Center Data Management Office *(ATTN: DATA)* as soon as possible. The on-study form and Eligibility Checklist should be submitted.

Note: A patient may be canceled only if no protocol therapy is administered. Written notification and an explantion must be received at RTOG as soon as this has been determined. RTOG will notify ECOG if the patient may be canceled. Once a patient has been given protocol treatment, all forms must be submitted.

5.3 The Cooperative Group will then phone RTOG Headquarters, Monday-Friday, between 8:30 a.m. to 5:00 p.m. ET and RTOG will assign the treatment option and RTOG case number.

5.4 After receiving the case number and treatment assignment, the Cooperative Group will phone their registering institution and relay this information.

5.5 The case number and treatment option will be confirmed by mail. RTOG will send a Confirmation of Registration and a Forms Due Calendar to the participating Cooperative Group for each case. The participating Group should then forward a copy of the calendar and the confirmation to the participating institution.

6.0 RADIATION THERAPY

6.1 External Beam

6.1.1 External beam RT with megavoltage linear accelerators *($\geq$ 6 MV)* will be used to deliver multiple *($\geq$ 2)* field techniques. Patients will be treated 5 days/week at 1.8 Gy/day. See Section 6.7.

6.1.2 Inital and conedown volumes will be based on the pretreatment simulation-esophagram films. At least 2 fields will be treated each day and portal films will be obtained of at least 2 fields per week or more often if needed. Portal verification films taken on the treatment unit should be copied and submitted to RTOG Headquarters for each field treated and when field modifications occur. It is not necessary to submit weekly quality assurance check films provided no changes have been made. Treatment may be given with combination of anterior/posterior posterior oblique, or lateral fields, such that the dose to the target volume

does not differ from the dose specified at isocenter by > 10%. Oblique fields cannot be used for the entire course. If a 4 field technique is used, the AP/lateral field can be alternated with the PA/lateral fields. If a 3 field technique is used, all 3 fields must be treated daily. The patient treatment position may be either supine or prone *(which may allow a shift in the esophagus in cases where sparing of the spinal cord is difficult).* Simulation on a diagnostic quality RT simulator which reproduces the geometry of the treatment machine is required. Refer to Section 6.5.

6.2 Technical Factors

6.2.1 *Beam Energy*: megavoltage equipment with photon energies ≥ *6 Mv is required)* is required.

6.2.2 *Treatment Distance*: The treatment distance to skin should be 100 cm for SSD technique and minimum isocenter distance should be 100 cm for SAD techniques.

6.2.3 *Blocking*: Primary collimation may be used, and blocking will be required only for shaping of the ports to exclude volume of tissues that are not to be radiated. Spinal cord blocking on the AP or PA field is prohibited. The use of off cord oblique or lateral block field arrangements will be necessary to meed the spinal cord normal tissue dose requirements *(Section 6.2.5).*

6.2.4 *Compensating Filters*: In case of sloping surface such as the thoracic inlet, compensating filters are recommended. If compensating filters are not available, appropriate reductions in field size must be done at prescribed dose levels to meet the dose homogeneity requirements as specified in Section 6.2.5.

6.2.5 *Normal Tissue Doses*: The spinal cord dose must not exceed 45 Gy maximum. Normal lung *(more than 2 cm outside the target volume)* must not receive more than 45 Gy. The entire heart dose should be no more than 30 Gy with < 50% of the organ receiving a maximum of 40 Gy.

6.2.6 *Fractionation*: At least 2 fields are to be treated daily, or 3 fields for a three-field technique. The daily fraction size will not deviate from that specified in Sections 6.7.1.1, 6.7.1.2, and 6.7.2.1 by more than ± 5 %. Deviations of ± 6-10% will be regarded as a minor deviation. Deviations greater than 10% will be regarded as a major treatment deviation.

6.2.7 *Therapy Interruptions*: If interruption of therapy *(up to two weeks)* becomes necessary, RT should be completed to the prescribed doses. Total number of fractions and elapsed days should be carefully reported. If more than two weeks interruption is necessary, resumption of treatment is at the discretion of the radiation oncologist. Patient will be considered a major deviation although follow-up will be continued.

6.3 Treatment Planning

(Use of CT strongly recommended, submit to RTOG with initial dosimetry data):

6.3.1 Isodose distribution at the mid-transverse plane of the tumor with tumor volumes clearly identified should be submitted. For the purpose of the distribution, it may be assumed that the central axis passes through the midplane of the tumor.

6.3.2 In addition to this distribution, two specific points of calculation are required.

6.3.2.1 A point 2 cm below the superior margin of the field or the point found to receive the maximum spinal cord dose. Maximum cord dose should be recorded daily on treatment record.

6.3.2.2 Supraclavicular node dose at 3 cm anterior depth, if applicable. Record daily on treatment chart.

6.3.2.3 Doses are prescribed and calculated **without** tissue inhomogeneity connection.

6.3.2.4 The dose delivered to the prescription point must not deviate by more than ± 5% relative to the doses specified in Section 6.7.1.1. Deviations of ± 6-10% will be scored as a minor deviation. Deviations greater than ± 10% will be scored as a major treatment deviation.

6.4 Dose Specifications

6.4.1 *Photon Beam*: For the following portal arrangements, the target dose shall be specified as follows:

6.4.1.1 For two opposed coaxial equally weighted beams: on the central ray at mid-separation of beams.

6.4.1.2 For an arrangement of 2 or more intersecting beams: at the intersection of the central ray of the beams.

6.4.1.3 For complete rotation or arc therapy: in the plane of rotation at the center of rotation.

6.4.1.4 For a single beam: on the central ray at the center of the target area.

6.4.1.5 For two opposing coaxial unequally weighted beams: on the central ray at the center of the target area.

6.4.1.6 Other or complex treatment arrangements: at the center of the target area *(Note: there may be several target areas).*

6.4.2 *Electron Beams*:

6.4.2.1 The target dose shall be prescribed at the depth of the maximum dose.

6.4.2.2 The energy and field size shall be chosen so that the target volume is encompassed within 90% *(or other appropriate minimum dose)* of the prescribed dose.

6.4.2.3 The allowed variation of dose across the target area shall be stated relative to the target dose.

6.4.2.4 The dose calculation point shall not be in a high dose gradient *(e.g. within 2 cm of the edge of a photon field)* and generally not in a blocked area. *(Several exceptions might be the cord dose under a cord block, or if a field reduction technique is used instead of compensators).*

6.5 Localization Methods

Localization films taken on treatment simulators are required using contrast material in the esophagus. For proximal 1/3 lesions, *(defined as primary tumors above the carina)* a metal marker *(BB)* should be placed on the lateral extent of the bilateral S/C fossa to be certain that they are included in the lateral and/or oblique fields. Verification portal films taken on the treatment unit are required until satisfactory, then at least weekly. Supraclavicular nodes that are involved should be outlined with a wire on the simulation film. Simulation films are to be taken of each field treated *(initial and boost)*, and submitted to RTOG Headquarters as described in *Section 12.0.*

6.6 **Dosimetry Monitoring:** The RTOG Radiation Oncology Quality Assurance office will conduct an initial rapid turn-around review and a final retrospective review by the Study Chairman for all participating groups. **Initial review and final RT materials from all groups should be sent directly to RTOG**. Participation in the Radiological Physics Center's TLD program is required.

6.7 **Doses and Fractionation of Radiation Therapy**

The maximum tumor extent superiorly, inferiorly, and laterally should be defined by esophagram, CT, or ultrasound. It should be noted that the RT doses are different for Arms 1 and 2.

6.7.1 ***Arm 1**: High Dose RT*

6.7.1.1 Initial Course *(50.4 Gy)* Target Volume: RT will begin on the first day of week 1. The superior and inferior borders of the field will be 5 cm beyond the tumor and the lateral borders of the field will be 2 cm beyond the lateral borders of the tumor as defined by endoscopic U/S, esophagram, or CT *(whichever is larger)*. The peri-esophageal nodes will be included. A barium swallow will also be obtained at the time of simulation to confirm the location of the esophagus. If the primary tumor is above the carina *(proximal esophagus)*, the supraclavicular nodes will be included in the initial *(50.4 Gy)* RT field. A localized photon or electron boost to the supraclavicular fossa *(SCF)* is allowed if the SCF dose is < 50.4 Gy *(specified at 3 cm depth from the anterior skin surface)*. The daily fraction size will be 1.8 Gy/day x 28 fractions.

6.7.1.2 Cone-Down *(14.4 Gy)* Target Volume: will be performed in a similar manner, however, the superior and inferior field will be decreased to 2 cm beyond the tumor. The lateral field will remain 2 cm beyond the lateral borders of the tumor as defined by CT or esophageal U/S, whichever is larger. A dose of 14.4 Gy *(1.8 Gy x 8 fractions)* will be delivered. The maximum dose to the spinal cord will be limited to 45 Gy.

6.7.2 ***Arm 2**: Conventional Dose RT*

6.7.2.1 The doses and techniques of RT will be identical to Arm 1 *(Section 6.7.1.1)* except patients will not receive the 14.4 Gy conedown *(Section 6.7.1.2)*. Therefore, the total dose will be limited to 50.4 Gy in 28 fractions.

6.8 **Radiation Toxicity**

6.8.1 For acute RT toxicity *(within 90 days from starting RT)* the RTOG Acute Radiation Morbidity Criteria should be used *(Appendix IV)*. If the patient develops ≥ grade 3 RT-related toxicity, RT and chemotherapy should be withheld. Treatment can resume once grade 3 RT-related toxicity is no longer present.

6.8.2 For long-term toxicity *(persisting or beginning beyond 90 days of treatment start)* refer to the RTOG Late Effects Radiation Morbidity Criteria in Appendix IV.

6.8.3 Unusual toxicities, and all grade 4-5 toxicities are to be reported by telephone to RTOG Headquarters.

6.8.4 A written report containing all relevant clinical information concerning the reported event will be sent to RTOG Headquarters via the respective Cooperative Group offices.

7.0 DRUG THERAPY

RTOG Institutional participation in chemotherapy studies must be in accordance with the Medical Oncology Quality Control Guidelines stated in the RTOG Procedure Manual.

7.1 **Dose Details**

7.1.1 *Cisplatin*: In both treatment arms, cisplatin *(75 mg/m^2)* will be given by bolus and will be delivered on the first day of weeks 1 and 5. Four weeks following the completion of RT, this will be repeated *(first day of post-RT weeks 1 and 5)*. Recommended guidelines for the use of mannitol, prehydration, and antiemetic coverage is seen in Appendix VI for those patients who receive therapy as an inpatient. Those who receive outpatient treatment should receive adequate pre-and post cisplatin hydration as clinically indicated.

7.1.2 *5-Fluorouracil*: In both treatment arms, 5-fluorouracil *(1000 mg/m^2/24 hrs)* will be delivered on days 1-4 of weeks 1 and 5. Four weeks following the completion of RT this will be repeated *(first day of post-RT weeks 1 and 5)*.

7.1.3 The insertion of a mediport for chemotherapy infusion is highly recommended.

7.1.4 *Attenuation schedule*: See Section 7.5.

7.1.5 Radiation therapy will begin on the first day of week 1 *(concurrent with cycle 1 of chemotherapy)*.

7.2 **5-Fluorouracil *(5-FU)***

7.2.1 *Dose Formulation*: 5-FU is available in 10-ml ampules, as a colorless to faint yellow aqueous solution containing 500 mg 5-FU, with pH adjusted to approximately 9.0 with sodium hydroxide. Administration of 5-FU should be only by the intravenous route taking care to avoid extravasation.

7.2.2 *Pharmacology*: 5-FU is a marketed drug available in 500 mg vials. It is fluorinated pyrimidine belonging to the category of antimetabolites. 5-FU resembles the natural uracil molecule in structure, except that a hydrogen atom has been replaced by a fluorine atom in the 5 position.

There is evidence that the metabolism of fluorouracil in the anabolic pathway blocks the methylation reaction of deoxyuridylic acid to the thymidylic acid. In this fashion 5-FU interferes with the synthesis of DNA and to a lesser extent inhibits the formation of ribonucleic division and growth, the effect of fluorouracil may be to create a thymidine deficiency which provides unbalanced growth and death of the cell.

7.2.3 *Supplier* 5-FU is available commercially.

7.2.4 *Storage*: Although 5-FU solution may discolor slightly during storage, the potency and safety are not adversely affected. Store at room temperature *(49°-86°F)*. Protect from light. If a precipitate occurs due to exposure to low temperatures, resolubilize by heating to l40°F with vigorous shaking; allow to cool to body temperature before using.

7.2.5 *Side Effects and Toxicities*: The spectrum of toxicity includes stomatitis and esophagopharyngitis *(which may lead to sloughing and ulceration)*, diarrhea with cramping and/or bleeding, anorexia, nausea and emesis are commonly seen during therapy. Leukopenia usually follows every course of adequate therapy with fluorouracil. The lowest white blood cell counts are commonly observed between the 9th and l4th days after the first dose, although uncommonly the maximal depression may be delayed for as long as 20 days. By the 30th day the count has usually returned to the normal range. Alopecia and dermatitis may be seen. The dermatitis most often seen is a pruritic maculopapular rash usually appearing on the extremities and less frequently on the trunk. Other side effects include myocardial ischemia, angina, lethargy, malaise, headache, allergic reactions, disorientation, confusion, euphoria, dizziness, uncoordination, visual changes, photosensitivity *(eyes and skin)*, nail changes including loss of nails, skin thickening, cracking, dryness or sloughing, vein pigmentation, biliary sclerosis, or acaculous cholecystitis.

7.3 Cisplatin *(CDPP)*

7.3.1 *Formulation*: Cisplatin *(Platinol)* is available as 10 mg and 50 mg vials of dry powder which are reconstituted with 10 ml and 50 ml of sterile water for Injection USP, respectively. Cisplatin is also available as a 1 mg/ml solution in 50 and 100 mg vials.

7.3.2 *Pharmacology*: The dominant mode of action of cisplatin appears to involve the formation of a bifunctional adduct resulting in DNA crosslinks. How this kills the cell remains unclear. There are data to indicate that its mode and sites of action are different from those of nitrogen mustard and the standard alkylating agents. Plasma levels of cisplatin decay in a biphasic mode with an initial half-life of 18 to 37 minutes, and a secondary phase ranging from 44 to 190 hours. This prolonged phase is due to protein binding which exceeds 90%. Urinary excretion is incomplete with only 27 to 45% excreted in the first five days. The initial fractions are largely unchanged drugs.

7.3.3 *Supplier*: Cisplatin is available commercially.

7.3.4 *Storage*: The intact vials should be stored at room temperature. Once reconstituted, the solution should be kept at room temperature to avoid precipitation. Due to a lack of preservatives, the solution should be used within eight hours of reconstitution. The solution may be further diluted in a chloride containing vehicle such as D5NS, NS, or D5-1/2NS *(ppt. occurs in D5W)*. Cisplatin has been shown to react with aluminum needles, producing a black precipitate within 30 minutes. Cisplatin should be given immediately after preparation as a slow intravenous infusion.

7.3.5 *Side Effects and Toxicity*: Includes anorexia, nausea, vomiting, renal toxicity *(with an elevation of BUN, creatinine, and impairment of endogenous creatinine clearance, as well as renal tubular damage which appears to be transient)*, ototoxicity *(with hearing loss which initially is in the high-frequency range, as well as tinnitus)*, hyperuricemia, seizures, rash, ocular toxicities, rare cardiac abnormalities, or possible acute myeloid leukemia. Much more severe and prolonged toxicity has been observed in patients with abnormal or obstructed urinary excretory tracts. Myelosuppression, often with delayed erythrosuppresion, is expected. In the high-dose treatment regimen with osmotic diuresis, the nadir of white cells and platelets occurred regularly at about two weeks with recovery generally at about three weeks after the initiation of therapy. Rare complications are loss of taste, allergic reactions, and loss of muscle or nerve function.

7.4 Cisplatin and 5-FU

The results of combination chemotherapy for esophageal cancer have been reviewed in detail. In brief, CDDP and vinca-alkaloid based combinations or CDDP/5-FU combinations have overall response rates of 45-50%. Response rates in patients with local-regional disease are substantially higher than in those patients with extensive disease *(50-60% vs 25-35%)*. Toxicity has been tolerable. The combination of CDDP and 5-FU was

chosen because of widespread familiarity with it in a variety of other tumor types including head and neck cancer. Dose limiting toxicities of the combination include nausea and vomiting, mucositis, and renal toxicity. Myelosuppression is relatively mild.

7.5 Dose Modifications/Attenuation Schedule for Chemotherapy

7.5.1 Dose modifications are based on findings since the last cycle of chemotherapy. Note that for hematological toxicity,there are separate attenuation schedules for toxicities based on day 1 of the current cycle as well as for interim *(between cycle)* toxicities, whichever is greater. See the Cooperative Group Common Toxicity Criteria for grading *(Appendix IV)*. Patients who develop grade ≥ 3 toxicity unrelated to RT *(oral mucositis, GU toxicity, and hand-foot syndrome [see Section 7.5.3.4])* should continue receiving RT but the chemotherapy should be withheld. Chemotherapy should resume when these grade ≥ 3 toxicities are no longer present. Chemotherapy doses which are withheld will not be made up. Growth stimulating factors are not allowed.

Hematology Toxicity (5-FU, Cisplatin)

Attenuation on the basis of Day 1 of Current Cycle Toxicities

WBC		Platelets	% Dose 5-FU, Cisplatin
≥ 3000	and	≥ 100,000	100%
2000 - < 3000	or	75,000 - < 100,000	50%
< 2000	or	< 75,000	0%* - and hold RT

Attenuation of the Basis of Interim *(between cycles)* Toxicities

WBC		Platelets	% Dose 5-FU, Cisplatin
≥ 1,000	and	≥ 75,000	100%
< 1,000 or		< 75,000	75%

*If therapy is held, parameters should be checked again in one week and treatment resumed if parameters are satisfied. The day on which therapy is reinitiated should be considered the study day following the last day therapy was given before the break *(e.g., if therapy is held on day 85, and begun again 7 days later, then day 85 is considered the day treatment was resumed)*. RT can resume when grade ≥ 3 toxicity is no longer present.

7.5.2 *GU Toxicity (Cisplatin)*

Creatinine Clearance		Serum Creatinine *	% Dose Cisplatin
≥ 65 cc/min	and/or	< 1.6 mg%	100%
≥ 55 cc/min to < 65 cc/min	and/or	1.6 mg% - 2.0 mg%	50%
< 55 cc/min		--	0%**

* If serum creatinine is used to adjust the dose, patient should be euvolemic and the value confirmed by a second serum creatinine.

** If Cisplatin is held due to poor renal function, 5-FU should also be held until cisplatin can be administered again. Parameters should be re-checked in one week.

7.5.3 *Cutaneous/Gastrointestinal Toxicity*

7.5.3.1 Mucositis *(5-FU)*

Patients with Grade ≥ 3 *(see Appendix IV)* stomatitis *(stomatitis is defined as limited to the oral cavity)* should receive no further 5-FU on that cycle. The 5-FU should be permanently reduced by 25% on all subsequent cycles. Intercurrent stomatitis of Grade ≥ 3 also requires a permanent 25% dose reduction.

7.5.3.2 Diarrhea *(5-FU)*

Any patient experiencing a Grade 3 *(Appendix IV)* toxicity must have their 5-FU discontinued for the remainder of that cycle, and reinstituted at a 25% permanent reduction. Intercurrent diarrhea of Grade ≥ 3 also requires a permanent 25% dose reduction.

7.5.3.3 Skin *(cutaneous)* toxicity due to 5-FU, or Cisplatin

For skin toxicities listed in Appendix IV, chemotherapy should be held for any listed Grade ≥ 3 or worse, and resumed when toxicity clears.

7.5.3.4 "Hand-Foot Syndrome" *(Palmer-Planter Erythrodysesthesia Syndrome) (5-FU only)*

Toxicity grades are defined in this way:

Grade 1 *(mild)* - any of the following: mild erythema, pain desquamation, dysesthesia, increased pigmentation, minimal fissures of edema. Toxicity does not interfere with daily activity.

Grade 2 *(moderate)* - any of the following: moderate erythema, blanching, edema, pain, dysesthesia, fissures or desquamation. Toxicity interferes with daily activity.

Grade 3 *(severe)* - any of the following: severe pain, dysesthesia, fissures, desquamation; any ulceration, necrosis. Toxicity incapacitating.

Dose modification of 5-FU for Hand-Foot Syndrome
(5 FU will be modified for all grades ≥ 1 even if no longer present)

Dose of 5-FU		
Grade 1	100%	on schedule
Grade 2	75%	delay until clear
Grade 3	50%	delay until clear

7.5.4 Neurotoxicity *(Cisplatin, 5-FU)*

Patients with Grade 3 toxicity *(see Appendix IV, usually peripheral neuropathic or auditory)* should have their Cisplatin dose held, then resumed at 50% when toxicity clears. 5-FU will also be held; radiotherapy should continue. Grade 4 toxicity would indicate that the drug be discontinued.

7.5.5 Anaphylaxis *(Cisplatin)*

Severe allergic reactions to Cisplatin are not uncommon. Patients who exhibit anaphylactic-type allergic reactions should not receive further Cisplatin. RT and 5-FU can continue unless otherwise contraindicated.

For delays, Cisplatin should also be held, but not radiotherapy unless other toxicities are present that would delay radiation. These should be considered permanent reductions.

7.6 Adverse Drug Reaction Reporting

7.6.1 The following guidelines for reporting adverse drug reactions *(ADRs)* apply to any research protocol which uses commercial anticancer agents. The following ADRs experienced by patients accrued to these protocols and attributed to the commercial agent*(s)* should be reported to the Investigational Drug Branch, Cancer Therapy Evaluation Program, within 10 working days:

7.6.1.1 Any ADR which is both serious *(life threatening, fatal)* and unexpected.

7.6.1.2 Any increased incidence of a known ADR which has been reported in the package insert or the literature.

7.6.1.3 Any death on study if clearly related to the commercial agent*(s)*.

ECOG Institutions - Any death from any cause while a patient is receiving treatment on this protocol or up to 30 days from the last dose of protocol treatment, or any death which occurs more than 30 days after protocol treatment has ended but which is felt to be treatment related must be reported.

7.6.1.4 Acute myeloid leukemia *(AML)*. The report must include the time form original diagnosis to development of AML, characterization such as FAB subtype, cytogenetics, etc. and protocol identification.

7.6.2 The ADR report should be documented on the FDA form 3500 *(Appendix V)* and mailed to:

Investigational Drug Branch *(IDB)*
P.O. Box 30012
Bethesda, MD 20824
(301)* 230-2330 *(24 hours)
FAX # 301-230-0159

ECOG Institutions - Submit written reports on the Adverse Reaction *(ADR)* Form for Investigational Drugs *(#391RF)* in lieu of FDA Form 3500.

7.6.3 A copy of reports and forms submitted to the IDB must be sent to RTOG via the respective group offices.

Eastern Cooperative Oncology Group **(ECOG)***
Data Management Office
303 Boylston Street
Brookline, MA 02146-7648
(ATTN: ADR)

North Central Cancer Treatment Group **(NCCTG)**
Operations Office
200 First Street, S.W.
Rochester, MN 55905

Radiation Therapy Oncology Group **(RTOG)**
American College of Radiology
1101 Market Street, 14th Floor
Philadelphia, PA 19107

* ECOG Institutions must also submit copies of reports and forms submitted to the IDB to their local IRB within 10 days.

8.0 SURGERY

Not applicable to this protocol.

9.0 OTHER THERAPIES

Not applicable to this protocol.

10.0 PATHOLOGY (9/8/98)

10.1 Required Material

10.1.1 All pathology reports from patients in the study will be submitted. Reports of the initial biopsy are necessary for the patient to meet eligibility criteria. However, all subsequent pathology reports if patient undergoes surgery must also be submitted. Reports must contain information about gross and microscopic contamination of surgical resection margins.

10.1.2 One H & E stained slide and either ten <u>un</u>stained slides or a tumor tissue block of all biopsies <u>must</u> be submitted. All submitted materials must include a Pathology Submission Form. If ECOG institutions submit paraffin blocks, they will be returned to the contributing institution and slides will be requested. Slides and blocks will be retained at LDS Hospital unless their return is requested.

10.1.3 Slides, blocks, and reports will be evaluated for the following specific features:

1. Presence of malignancy.
2. Type of carcinoma: only epidermoid or adenocarcinoma of the esophagus is eligible for the study.
3. Grade of carcinoma.
4. Presence or absence of keratinization.
5. The unstained slides will be used to assess genetic and molecular features.
6. Other special studies.

10.1.4 To encourage compliance, your Pathology Department will be reimbursed for obtaining blocks or cutting slides. Contact RTOG Administration.

10.1.5 Patient consent form should give the Pathology Department authority and responsibility to comply with this request *(pathology blocks belong to the patient from whom tissue has been removed).*

10.2 Pathology Submission

10.2.1 RTOG and NCCTG members will send pathology materials directly to:

LDS Hospital
Dept. of Pathology
E.M. Laboratory
8th Ave & C Street
Salt Lake City, UT 84143

10.2.2 ECOG members will send pathology materials to the ECOG Pathology Office:

Evanston Hospital
Room B624
2650 Ridge Avenue
Evanston, IL 60201-1797

11.0 PATIENT ASSESSMENTS

11.1 Study Parameters (11/13/95, 3/1/96, 4/22/96)

Parameter	Prior to Random-ization *(≤ 28 days)*	Prior to each chemo cycle	After each cycle chemo	*≤ 28 days* after RX Completion	At Follow-up
History & Physical	X	X		X	X
CBC, Platelets	X*(f)*	X*(a)*			X*(e)*
SMA-12*	X*(f)*		X	X	X*(e)*
Chest X-ray	X			X	X*(e)*
Bronchoscopy[i]	X				
Audiography	X*(b)*	X*(b)*			
Barium Contrast UGI	X			X	X*(e)*
Abdominal & Chest CT	X				X*(e)*
EKG	X				
Endoscopic Ultrasound	X*(c)*				X*(c,e)*
Bone Scan	X*(d)*				
PFTs *(Spirometry + DLCO)*	X*(c)*				X*(c, g)*
Biopsy	X				X*(j)*
Quality of Life	X			X	X*(e)*
Toxicity		X		X*(h)*	X *(e)*

* SMA-12 = AST, LDH, Alk phos, total bilirubin, total protein, albumin, uric acid, phos, calcium, BUN

a) must be done on day 1 of each chemo cycle and q 2 weeks during chemotherapy.
b) if clinically indicated
c) optional
d) if serum alkaline phosphatase elevated ≥ 1.5 times normal
e) q 4 months for 1 year then q 6 months for 2 years then yearly.
f) within 2 weeks prior to randomization.
g) at 8 months only
h) weekly during RT
i) if tumor is < 30 cm from the incisors.
j) at the time of x-ray evidence of local recurrence.

11.2 Criteria for Response

11.2.1 Assessments for response will be performed following the completion of all chemotherapy and RT, as outlined in Section 11.1. until progression of disease.

11.2.1.1 All tumor measurements will be recorded in centimeters *(cm)*.

11.2.1.2 Complete Clinical Remission: Disappearance of all clinical evidence of active tumor. The patient must be free of all symptoms.

11.2.1.3 Complete Pathologic Response: No tumor found at pathologic review of the biopsy.

11.2.1.4 Partial Remission: 50% or greater decrease in the size of the primary lesion, but positive washing, brushing or biopsy and/or residual tumor on barium swallow.

11.2.1.5 Minor Response: Greater than 25% decrease in all measurable disease, but less than that required for a partial remission.

11.2.1.6 Stable Disease: No objective change in any disease parameter throughout the period.

11.2.1.7 Progressive Disease: Unequivocal increase in the size of any measurable lesion. Appearance of significant new lesions will also constitute progressive disease.

11.3 Criteria for Progression of Disease

11.3.1 While it is recognized that it is not always possible to obtain pathologic proof of progressive disease, biopsy or autopsy material confirming recurrent cancer is highly desirable and every reasonable attempt to obtain such is encouraged.

11.3.2 In the absence of histologic or cytologic proof of recurrence, clinical evidence *(including new masses on CT scan, new lesions on bone scan, ascites not explained by other causes or enlarging mass by endoscopic U/S)*,

although highly suspicious of recurrent disease will not result in change in patient's management. These findings should lead to a search for a mass that could be biopsied.

11.3.3 Patients who develop progression of disease at the primary site while receiving RT + chemotherapy or develop metastatic disease outside the RT field will be considered treatment failures. They may be treated with any form of palliative therapy at the discretion of their physician.

11.3.4 Patients who develop local recurrence only may be offered surgery; they will be considered treatment failures. Those who develop metastases may be offered chemotherapy. They will be considered treatment failures. The regimen chosen may include a variety of phase II agents under study or conventional chemotherapy.

11.3.5 Cumulative patterns of failure should be obtained. The dates and sites of all failure patterns must be reported.

11.4 Criteria for Toxicity

11.4.1 The following toxicities are anticipated: nausea, vomiting, diarrhea, mucositis, phlebitis, fatigue, anorexia, myelosuppression, renal dysfunction, ototoxicity, peripheral neuropathy.

11.4.2 *On treatment*, toxicity will be graded according to the RTOG Acute Radiation Morbidity Scoring Sheet. Chemotherapy toxicity will be scored according to the Cooperative Group Common Toxicity Criteria.

11.4.3 *Post-treatment*, toxicity will be graded according the RTOG Late Radiation Morbidity Scoring Sheet. Chemotherapy toxicity will be scored according to the Cooperative Group Common Toxicity Criteria.

11.5 Criteria for Removal From Study Analysis

11.5.1 Efforts shall be made to account for all patients entered into the study during the evaluation of results. However, in detailed evaluation, the following patient categories will be considered.

11.5.2 *Early Deaths*: Those patients who died within six weeks of beginning therapy as a result of an event not related to esophageal cancer or to the study drugs. Complete records of these patients will be reviewed by the Data Monitoring Committee.

11.5.3 *Lost to Follow-up*: Those patients in whom there is inadequate information to judge tumor response because of loss of contact in which repeated attempts to obtain information are unsuccessful.

11.5.4 *Major Protocol Violations*: Patients who receive further therapy or deviate from the treatment program by either adding a chemotherapeutic agent, by substantially modifying the dosage and schedule of the study drugs *(as defined in Section 7.0)* or radiation *(as defined in Section 6.0)*.

11.5.5 Criteria for eligibility are not met.

11.6 Quality of Life Assessments

11.6.1 *The Functional Assessment of Cancer Therapy (FACT) - H&N (version 2)* is the quality of life assessment tool used. Based on previously reported esophageal patients' concerns, symptoms and treatment side effects, 17 additional items are included as disease-specific concerns.[13,18,31,33,34]

11.6.2 *The FACT-H&N (version 2)* scale is a 43-item self-report quality of life tool. Using a Likert-type format, items are grouped into five subscales assessing physical well-being, social/family well-being, relationship with doctor, emotional well-being, functional well-being, and additional concerns. Following each subscale, an additional item attempts to reflect patients' perception of the degree to which his/her quality of life is affected by a subscale's domain. These additional items may be used to weight subscale scores in the composite quality of life score.[36]

11.6.3 The FACT-H&N is comprised of the FACT-C, a 33-item tool, and the last "additional concern" subscale. Prior use of the FACT-G with 630 cancer patients supports tool construct validity with correlations to a shortened Taylor Manifest Anxiety Scale *(.57)*, Brief Profile of Mood States *(.69)*, ECOG Performance Status Rating of anxiety level *(-.56)* and no correlation with the brief Marlowe-Crowne Social Desirability Scale *(.03)*. A high correlation with the Functional Living Index -Cancer *(.80)* supports concurrent validity. Internal consistency *(coefficient alpha)* of .89 was found with the 28-item FACT; the individual subscale alpha ranged from .65 to .82.

11.6.4 *Administration*: Consistent with the belief that the most meaningful information is elicited from the patient, this tool is designed for self-administration and self-report of patient's perceived quality of life. Assistance from family members is not permitted. However, assistance by the tool administor through reading the items and responses to the patients or circling response indicated by the patient is allowed in cases where the respondent is unable to read or writing capacity is hindered. If assistance is provided in any manner, this is to be so noted on the first page of the QOL Form *(cover page)*. The patient will complete this tool while alone in an undisturbed setting. The same person should explain the tool to the patient at each administration if possible. The administrator will avoid influencing responses.

11.6.4.1 If the patient is unable to complete these forms due to language barriers, this must be appropriately documented on the QOL form. Note that the FACT-H&N is available in English or Spanish.

11.6.4.2 If the due date of QOL assessment does not coincide with a scheduled appointment, the QOL Assessment forms may be mailed to the patient with appropriate information/instructions and preferably with an

enclosed self-addressed, stamped return envelope. Another method is to conduct a telephone "interview", reading each item verbatim. If this is done, a form should be mailed to the patient in advance.

11.6.4.3 The following is to be read to the patient at each administration: "As part of our evaluation of your treatment, we would like to learn about how you see your quality of life at several times before and after treatment. This is why we are asking you to take a few minutes to respond to these statements. Please read the directions at the top of page." After the patient reads the instructions, ask: "Do you have any questions about how to complete this form? Address individual questions/concerns as needed. Then state, "please try not to skip any items." Upon completion, review the forms to ensure all items have one and only one response.

NOTE: The initial baseline questionnaires must be completed prior to the commencement of any protocol therapy. Retain original forms as primary source documentation and forward copies to headquarters.

12.0 DATA COLLECTION (9/8/98, 7/1/99)

12.1 All material with the exception of Dosimetry and initial Medical Oncology data will be sent to the responsible Cooperative Group according to the following schedule and then forwarded to RTOG Headquarters. **All preliminary and final dosimetry materials must be sent directly to RTOG Headquarters**, 1101 Market Street, 14th Floor, Philadelphia, PA 19107. All dosimetry material *(films, etc.)* must be identified with labels available from RTOG. **All data items must be identified with both RTOG and other Group's study and case numbers. Unidentified data/films will be returned.**

Item	Due
Demographic Form **(A5)**	Within one week of randomization
Initial Evaluation Form **(I1)**<br>Diagnostic Pathology Report **(P1)**<br>Pathology Slides **(P2)**<br>Baseline FACT-H&N Forms **(FA)**<br>*(prior to protocol RX)*	Within two weeks of randomization
Preliminary Dosimetry Information:<br>Tx Prescription **(T2)**, Central Axis Calculation **(T4)**, Initial Field Films *(Sim & Portal)* **(T3)**<br>Planning CT, CT Report, if done **(C1, C3)**	Within one week of commencement of RT
Chemotherapy Flow Sheet **(M1)**	Within 2 weeks after completion of each course of chemotherapy.
Radiotherapy Form **(T1)**<br>Final Dosimetry Information:<br>Treatment Sheets **(T5)**,<br>Isodose Computations **(T6)**<br>Boost Field Films *(Sim & Portal)* **(T8)**<br>FACT H&N Forms **(QF)**	Within 2 weeks of completion of RT
Surgery Form **(S1)**<br>Operative Report **(S2)**<br>Surgical Path Report **(S5)**	Within 2 weeks of "salvage surgery" as applicable
Follow-up Assessment Form **(F1)**	Every 4 months from start of treatment x 3; then every 6 months to year 5, then annually, at progression/relapse, and at death

FACT H&N Forms **(QF)*** At 8 months from start of treatment, at 1 year, then q 6 months to year 5, then annually.

Autopsy Report **(D3)** As applicable.

* The end-of treatment evaluation should be done not less than 1 week, but not more than 4 weeks, after the end of RX.

12.2 RTOG will send a forms package to RTOG member for each case registered. Other groups will attach a forms appendix to their members' version. It will be the responsibility of other groups' members to copy the attached forms and to maintain a supply of available forms for data submission. **The RTOG and other group's assigned case and study numbers must be recorded on all data items submitted.** Data should be routed according to the mechanism set up by each participating Group. Generally the participating Group will require forms to be routed through their offices and they will send the forms to:

American College of Radiology
Radiation Therapy Oncology Group - 14th Floor
1101 Market Street
Philadelphia, PA 19107

12.2.1 *For ECOG Institutions* - Originals of completed forms must be sent by the institutions to the ECOG Statistical Center Data Management Office *(ATTN: DATA)*, 303 Boylston Street, Brookline, MA 02146-7648. The RTOG case number as well as the ECOG case number should appear on every form. Investigators should retain a copy for their records. The ECOG Statistical Center Data Management Office will forward date-stamped originals to the RTOG Headquarters. ECOG members should NOT send forms directly to RTOG. **DO NOT use ECOG data forms**. Only RTOG forms are to be used.

12.3 Rapid Review Items:

Time critical data which requires rapid submission must be sent directly to RTOG :

M2 - Medical Oncology Treatment Planning Form *(fax #215/928-0153)*
T2 - Protocol Treatment Form *(fax #215/928-0153)*
T3 - Photon localization film *(for all fields treated initially)*
T4 - Photon dose calculations *(for all fields treated initially)*

12.4 Request for Study Information and Forms Request:

Requests for additional information or clarification of data will be routed through the appropriate Cooperative Group office for distribution to the individual institution. The RTOG memo requesting the additional information must be returned with the response. Responses should be returned according to the procedure used to submit data forms. You may receive reminders prompting response. Periodically *(generally three time a year)*, computer-generated lists identifying delinquent material are prepared and distributed.

13.0 STATISTICAL CONSIDERATIONS

13.1 Endpoints

13.1.1 Overall Survival *(Failure: death from any cause)*
13.1.2 Disease Free Survival *(Failure: disease relapse or second primary or death without progression)*
13.1.3 Patterns of Failures associated with diseasefree survival
13.1.4 Local Control Rate *(Failure: disease never clear or if clear, time to local disease recurrence)*
13.1.5 Time to Distant Failure *(Failure: first appearence of distant metastasis)*
13.1.6 Toxicity
13.1.7 Quality of Life

13.2 Sample Size

The estimated two year survival rate for the control arm of 50 Gy with concurrent chemotherapy, based upon the results of the randomized and non-randomized RT+Chemo arms from the completed intergroup study *(RTOG 85-01/SWOG 8598/NCCTG 88-40-51)*, is 36%[18]. It is projected that increasing of the radiation dose to 64.8 Gy will produce a two year survival rate of 50%. The survivals are assumed to approximately follow an exponential distribution. Patients will enter the study uniformly over 4 years with 2 additional years follow up.

For significance level of .05, statistical power of .80, and a two sided test, a total of 270 patients will be required.[19,20] Guarding against an ineligibility/unevaluability *(no data)* rate up to 10%, **a total of 298 patients will be entered.**

In conformance with the National Institute of Health *(NIH)* Revitalization Act of 1993 with regard to inclusion of women and minority in clinical research, we have also considered the possible interaction between gender/race and treatments. The results from the randomized and non-randomized RT+Chemo arms in the RTOG 85-01 study revealed that there were no significant differences between males *(99/124=80%)* and females *(25/124=20%)* in overall survival outcome *(p-value=0.37)*.[35] If 80% of patients recruited in this study are males, the powers to detect two year survival rate of 36% from 50%, 55%, and 60% are respectively 71%, 92%, and 99%. Likewise, if 20% of patients recruited in this study are females, the powers to detect two year survival rate of 36% from 50%, 55%, and 60% are respectively 24%, 40%, and 57%. The same study also indicated that there were no significant differences between whites *(85/124=69%)* and nonwhites *(39/124=31%)* in overall survival outcome *(p-value=0.45)*.[35] If 69% of patients recruited in this study are white, the powers to detect two year survival rate of 36% from 50%, 55%, and 60% are respectively 64%, 88%,

and 97%. Likewise, if 31% of patients recruited in this study are nonwhites, the powers to detect two year survival rate of 36% from 50%, 55%, and 60% are respectively 35%, 56%, and 75%.

There is one quality of life tool in this trial; the Functional Assessment of Cancer Therapy *(FACT-H&N)*, with additional disease-specific questions. FACT-H&N is a multi-dimensional questionnaire that qualitatively assesses how the patient feels with respect to physical, social and emotional well-being, and relationship with doctor and fulfillment.

FACT-H&N and additional questions will administered according to the schedule in Section 12.1. Global quality of life change, the total score of the first 33 questions of FACT-H&N, will be measured at each time point with an average difference between groups ≥ 5.4 being clinically significant.[33] Sufficient sample size will be required to compare changes in quality of life for: High Dose RT + Chemo vs. Conventional RT + Chemo. The variance and FACT-H&N participation rates for each time point and each group will be determined at each interim analysis point. Completion of FACT-H&N will be requested of all patients unless accrual to this portion of the trial is terminated early *(Section 13.5.1)*. The additional questions are experimental, administration of these questions will be terminated in conjunction with FACT-G.

13.3 Accrual for the Study

Results from the preceding intergroup study provide an estimate for the accrual rate. During the last two years of RTOG 85-01 *(1989-1990)*, the accrual rate was 4.2 patients per month. Participation from ECOG and NCCTG, is planned in this study. With this broad based intergroup participation, the required accrual of 6.2 cases per month to complete the accrual in four years seems feasible.

13.4 Randomization

The treatment allocation scheme described by Zelen[21] will be used because it balances patient factors other than institution. The stratifying variables include: weight loss, lesion size and histology.

13.5 Analyses plan

13.5.1 *Interim Analyses to monitor the study progress*:

Interim reports with statistical analyses will be prepared twice a year until the initial paper reporting the treatment results has been submitted. In general, the interim reports will contain information about the patient accrual rate with a projected completion date for the accrual phase, data quality, compliance rate of treatment delivery with the distributions of important prognostic baseline variables and the frequencies and severity of the toxicities by treatment arm. They will not contain the results from the treatment comparisons with respect to the efficacy endpoints. *(Overall survival, disease free survival, patterns of failure)*

After 150 patients are randomized *(75 per arm)*, the variance and completion rates for FACT-H&N at each time point *(see Section 12.1)* will be computed. Assuming a clinically significant difference for global quality of life change is 5.4, the power will be calculated with the variance estimate and completion rate at the time of analysis for each group and time point of interest *(see Section 13.2).* The results of this analysis will be presented to the RTOG Data Monitoring Committee*(DMC).*

13.5.2 Significance testing for early termination

The first significance test comparing the survivals between the two treatment arms will be performed for the first RTOG semi-annual meeting after 50% of the required sample size is available. The result will be then reported to the DMC. If the difference is highly significant with $p < .001$, the study statistician will recommend to the DMC that randomization be discontinued and the study published.

The second significance test comparing the survivals between the two treatment arms will be performed for the first RTOG semi-annual meeting after 75% of the required sample size is available. The result will be then reported to the DMC. If the difference is highly significant with $p < .001$, the study statistician will recommend to the DMC that randomization be discontinued and the study be published.

The third significance test comparing the survivals between the two treatment arms will be performed for the first RTOG semi-annual meeting after 100% of the required sample size is available and the result will be then reported to the DMC. If the difference is highly significant with $p < .001$, the study statistician will recommend to the DMC that the study be published.

13.5.3 Analysis for Reporting the Initial Treatment Results:

This major analysis will occur after each patient has been potentially followed for a minimum of 24 months unless the study is stopped earlier. It will include tabulation of all cases entered, and those excluded from the analyses with the reasons, the distribution of the important prognostic baseline variables, and observed results with respect to the endpoints mentioned in Section 13.1. The primary hypothesis for the study is whether the control and the experimental arms have different effects on overall survival. All eligible patients randomized will be included in the comparison. All eligible patients randomized will be grouped by assigned treatment arm in the analysis. The significance level of 0.047 will be used in this analysis to preserve an overall significance level of .05 for the study. The primary hypothesis of treatment benefit will be tested using the Cox proportional hazard model with the stratification factor of weight loss, lesion size, and histology included as covariates. Additional analyses of treatment effect will include modifying factors such as age, sex, race, and other patient characteristics. These analyses will also use the Cox proportional hazard model. The treatment comparison on disease free survival, local control rrate, and time to distant failure will be analyzed in a similar fashion. The treatment comparison on the patterns of treatment failures and of 3+ grade toxicity will use the z-statistic for testing binomial proportions.

Analysis of quality of life data will be performed on all patients that complete baseline questionnaires. The analysis will utilize analysis of variance, repeated measures analysis of variance, and quality adjusted survival methods for comparing treatments. The additional questions will be examined for internal consistency, content and construct validity, and responsiveness. Subgroup analysis of global quality of life will be undertaken for groups with sufficient sample size.

REFERENCES

1. Leichman L, Steiger Z, Seydel HG, et al: Preoperative chemotherapy and radiation therapy for patients with cancer of the esophagus: A potentially curative approach. J Clin Oncol 2:75-79, 1984.

2. Poplin E, Fleming T, Leichman.L. , et al: Combined therapies for squamous-cell carcinoma of the esophagus, a Southwest Oncology Group Study (SWOG-8037). J Clin Oncol 5:622-628, 1987.

3. Naunheim KS, Petruska PJ, Roy TS, et al: Preoperative chemotherapy and radiotherapy for esophageal carcinoma. J Thorac Cardiovasc Surg 5:887-895, 1992.

4. Urba SG, Orringer MB, Perez-Tamayo C, et al: Concurrent preoperative chemotherapy and radiation therapy in localized esophageal adenocarcinoma. Cancer 69:285-291, 1992.

5. Forastiere A: Treatment of localregional esophageal cancer. Sen Oncol 19:57-63, 1992.

6. Forastiere A, Orringer MB, Perez-Tamayo C, et al: Concurrent chemotherapy and radiation therapy followed by transhiatal esophagectomy for local-regional cancer of the esophagus. J Clin Oncol 8:119-127, 1990.

7. Kavanagh B, Anscher M, Leopold K, et al: Patterns of failure following combined modality therapy for esophageal cancer, 1984-1990. Int J Radiation Oncol Biol Phys 24:633-642, 1992.

8. Gill PG, Denham JW, Jamieson GG, et al: Patterns of treatment failure and prognostic factors associated with the treatment of esophageal carcinoma with chemotherapy and radiotherapy either as sole treatment of followed by surgery. J Clin Oncol 10:1037-1043, 1992.

9. Coia LR, Engstrom PF, Paul AR, et al: Long-term results of infusional 5-FU, mitomycin-C, and radiation as primary management of esophageal cancer. Int J Radiation Oncol Biol Phys 20:29-36, 1991.

10. John MJ, Flam M, Ager Mowry PA, et al: Radiotherapy alone and chemoradiation for nonmetastatic esophageal carcinoma. Cancer 63:2397-2403, 1989.

11. Izquierdo MA, Marcuello E, Gomez de Segura G, et al: Unresectable nonmetastatic squamous cell carcinoma of the esophagus managed by sequential chemotherapy (cisplatin and bleomycin) and radiation therapy. Cancer 71:287-292, 1993.

12. Araujo CMM, Souhami L, Gil RA, et al: A randomized trial comparing radiation therapy versus concomitant radiation therapy and chemotherapy in carcinoma of the thoracic esophagus. Cancer 67:2258-2261, 1991.

13. Herskovic A, Martz LK, Al-Sarraf M, et al: Combined chemotherapy and radiotherapy compared with radiotherapy alone in patients with cancer of the esophagus. New Eng J Med 326:1593-1598, 1992.

14. Roussel A, Jacob JH, Jung GM, et al: Controlled Clinical Trial for the Treatment of Patients with Inoperable Esophageal Carcinoma: A Study of the EORTC Gastrointestinal Tract Cancer Cooperative Group, in Recent Results in Cancer Research. Berlin, Springer-Verlag, 1988, pp 21-30

15. Nygaard K, Hagen S, Hansen HS, et al: Pre-operative radiotherapy prolongs survival in operable esophageal carcinoma: A randomized, multicenter study of pre-operative radiotherapy and chemotherapy. The second Scandinavian trial in esophageal cancer. World J Surg 16:1104-1110, 1992.

16. Sischy B, Ryan L, Haller D, et al: Interim report of EST 1282 phase III protocol for the evaluation of combined modalities in the treatment of patients with carcinoma of the esophagus. Proc ASCO 9:105, 1990.(Abstract)

17. Al-Sarraf M, Pajak T, Herskovic A, et al: Progress report of combined chemo-radiotherapy (CT-RT) vs. radiotherapy (RT) alone in patients with esophageal cancer. An intergroup study. Proc ASCO 12:197, 1993.(Abstract)

18. Herskovic A, Martz K, Al-Sarraf M, Leichman L, Brindle J, Vaitkevicius V, Cooper J, Byhardt R, Davis L, and Emami B. Significant advantage of chemo-radiotherapy compared to radiation alone in randomized phase III trial for patients with esophageal cancer: an intergroup study. New Eng Med 326; 1593-1598, 1992

19. Makuch, R.W. and R. W. Simon.: Sample size requirements for comparing time-to-failure among treatment groups. Journal of Chronic Disease 35:861-867, 1982.

20. Donner, A.: Approaches to samples size estimation in the design of clinical trials - a review. Statistics in Medicine. Vol. 3, 199-214, 1984.

21. Zelen M. The randomization and stratification of patients to clinical trials. J Chron Dis 27: 365-375, 1974.

22. Moinpour, CM, Feigl, P, Metch, B, Hayden, KA, Meyskens, FL, & Crowley, J. Quality of life endpoints in cancer clinical trials: Review and recommendations. Journal of the National Cancer Institute 81: 485-495, 1989.

23. Aaronson, NK, Meyerowitz, BE, Bard, M, Bloom, JR, Fawzy, FI, Feldstein, M, Fink, D, Holland, JC, Johnson, JE, Lowman, JT, Patterson, WB, and Ware, JE. Quality of life research in oncology: Past achievements and future priorities. Cancer 67: 839-843, 1991.

24. Aaronson, NK Methodologic issues in assessing the quality of life of cancer patients. Cancer 67: 844-850, 1991.

25. Watson, A. A study of the quality and duration of survival following resection, endoscopic intubation and surgical intubation in oesophageal carcinoma. British Journal of Surgery 69: 585-588, 1982.

26. Kai, H, Matsufuji, H, Okudaira, Y, and Sugimachi, K. Heat, drugs, and radiation in combination is palliative for unresectable esophageal cancer. International Journal of Radiation Oncology Biology and Physics 14: 1147-1152, 1988.

27. Karlin, DA, Fisher, RS, & Krevsky, B. Prolonged survival and effective palliation in patients with squamous cell carcinoma of the esophagus following endoscopic laser therapy. Cancer 59: 1969-72, 1987.

28. Welvaart, K and DeJong, PL. Palliation of patients with carcinoma of the lower esophagus and cardia: The question of quality of life. Journal of Surgical Oncology 32: 197-199, 1986.

29. Flores, AD, Nelems, B, Evans, K, Hay, JH, Stoller, J, and Jackson, SM. Impact of new radiotherapy modalities on the surgical management of cancer of the esophagus and cardia. International Journal of Radiation Oncology Biology and Physics 17: 937-944, 1989.

30. Bluett, JK, Sawyers, JL, and Healy, D. Improved quality of survival with resection. The American Surgeon 53: 126-132, 1987.

31. Sugimachi, K, Maekawa, S, Koga, Y, Ueo, H, and Inokuchi, K. The quality of life is sustained after operation for carcinoma of the esophagus. Surgery, Gynecology and Obstetrics 162: 544-546, 1986.

32. Cella, DF et al. Manual for the Functional Assessment of Cancer Therapy (FACT) Scales version 2, April 1993.

33. Cella, DF et al. The Functional Assessment of Cancer Therapy (FACT) Scale: Development and validation of the general measure. Journal of Clinical Oncology, 870-879, 1993.

34. Calais, G, Jadavil, E, Chapet C, et al. High dose radiotherapy (RT) and concomitant chemotherapy (CT) for nonresectable esophageal cancer. Results of a phase II study. Proc. Asco 13: 197, 1994.

35. JD Cox, chair of the Radiation Therapy Oncology Group: Personal communications.

36. Cella, DF. Preliminary Manual Functional Assessment of Cancer Therapy (FACT) Scales. 1992.

APPENDIX I

INT 0123

RADIATION THERAPY ONCOLOGY GROUP

RTOG 94-05

A PHASE III INTERGROUP RANDOMIZED COMPARISON OF COMBINED MODALITY THERAPY FOR CARCINOMA OF THE ESOPHAGUS: HIGH DOSE VS. CONVENTIONAL DOSE RADIATION THERAPY

SAMPLE PATIENT CONSENT FORM

RESEARCH STUDY

I have the right to know about the procedures that are used in my participation in clinical research so as to afford me an opportunity to make the decision whether or not to undergo the procedure after knowing the risks and hazards involved. This disclosure is not meant to frighten or alarm me; it is simply an effort to make me better informed so I may give or withhold my consent to participate in clinical research.

PURPOSE OF THE STUDY

My physician*(s)* in Radiation and Medical Oncology have informed me of a new study using chemotherapy and radiation therapy for the treatment of esophageal cancer.

I understand that I have cancer of the esophagus and treatment is recommended. The standard treatment for cancer of the esophagus is radiation plus chemotherapy and/or surgery. At some institutions some patients are treated with surgery alone, but most of the patients are treated with radiation plus chemotherapy. The purpose of this research study is to determine if chemotherapy plus high dose radiation therapy is more effective than chemotherapy plus conventional dose radiation therapy in treating cancer of the esophagus. The reason for this is that patients treated with conventional dose chemotherapy and radiation therapy still have a moderate chance of tumor recurring both in the esophagus and other parts of the body. Giving higher doses of radiation therapy may decrease the recurrence rate. This point however has not been proven and that is why my physicians are participating in this clinical trial.

Treatment for cancer of the esophagus may affect my life and how I feel in many ways. It is important to understand how I view my quality of life. In the future this information will help health care providers and patients make more informed treatment decisions

DESCRIPTION OF PROCEDURES

This study involves at random *(by chance)* assignment to one of two treatment arms. It is not clear at the present time which of the two is better. For this reason the therapy which is to be offered to me will be based upon a the method of selection called randomization. Randomization means that my physician will call a statistical office which will assign me one of the two treatments by computer. The chance of my receiving one of the two therapies is approximately equal. I will to receive one of the following treatment plans:

Treatment 1

The 5-Fluorouracil *(5-FU)* and cis-Platinum chemotherapy will begin on day 1 of weeks 1 and 5. 5-FU will be given over 4 days. Radiation therapy will begin on day 1 and be given at the same time as chemotherapy. The radiation therapy treatments will be given daily for 5 days per week, Monday-Friday. The duration of the radiation treatment will be approximately 7 1/2 weeks. One month following the end of radiation therapy, the chemotherapy will be repeated. It will begin on day 1 of weeks 1 and 5. I may be in the hospital a minimum of 4 days for each of the 4 chemotherapy cycles.

Treatment 2

The 5-Fluorouracil and cis-Platinum chemotherapy will begin on day 1 of weeks 1 and 5. 5-FU will be given over 4 days. Radiation therapy will begin on day 1 and be given at the same time as chemotherapy. The radiation therapy treatments will be given daily for 5 days per week, Monday-Friday. The duration of the radiation treatment will be approximately 5 1/2 weeks. One month following the end of radiation therapy, the chemotherapy will be repeated. I will be in the hospital a minimum of 4 days for each of the 4 chemotherapy cycles.

Chemotherapy is medicine which will be given into my vein. I may have to be in the hospital as the treatment is given slowly over a period of four days and given four times during the entire treatment. The drug combination will be cis-Platinum and 5-Fluorouracil.

Cis-Platinum will be given through an intravenous *(in the vein)* tube over a few minutes in the morning. Because cis-Platinum can cause kidney damage, intravenous fluids may be needed to "flush" the kidneys. Mannitol, a drug which increases urine production, is given intravenously just prior to and after receiving cis-Platinum. So that my physician may better assess my kidney function during this time, I may be asked to save urine specimens and to keep a record of all fluids taken by mouth during the first 24-48 hours after cis-Platinum.

The second medicine is called 5-Fluorouracil *(5-FU)*. It will be infused over a 4-day period. This medicine is less likely to cause side effects when it is given at a slow constant rate like this. After the first treatment, doses of drugs may be changed to fit my needs. Both 5-FU and cis-Platinum may cause nausea and vomiting. In order to minimize these side effects, Ondansetron or a similar antivomiting medication will be given either as a pill, shot or suppository prior to chemotherapy and every 3-4 hours thereafter as needed. I understand that procedures will also include blood studies, x-rays, biopsy of the tumor, and any other tests which my physician feels are necessary.

I will be asked to complete the quality of life assessment forms at several points in time before and after I complete treatments. It will take approximately 15 minutes each time to complete these forms.

Also, at the time of my diagnosis by biopsy, all or some of my tumor was removed. As is usually done, this tissue went to the hospital's pathology department for routine testing and diagnosis. After that process was complete, remaining tumor samples were stored in the pathology department. I am being asked for permission to use the remainder of the tumor for additional tests. Since this tissue was removed at the time of surgery or biopsy, the permission to use my tissue will not involve any additional procedure or expense to me. The tumor tissue's cells will be examined to see if any special "markers", tests which predict how a patient with tumors like mine responds to treatment, can be identified.

RISKS AND DISCOMFORTS

Side effects of the treatments are:

Radiation Therapy - Common side effects include reddening of the skin and hair loss in the treatment area, tiredness, and sore throat causing difficulty with swallowing. Rare complications include esophageal stricture *(tightening)*, fistula or perforation , as well as radiation pneumonitis *(scarring of the lungs)* and myelitis *(nerve damage)*.

Chemotherapy

5-Fluorouracil *(5-FU)* may cause nausea, loss of appetite, vomiting, diarrhea with cramping or bleeding, skin rash, tiredness, headache, confusion, inflammation of the fingers and toes, mouth sores, sore throat, reversible hair loss, chest pain, increased sensitivity to sunlight, skin, nail or vein darkening or thickening, and a depression of the bone marrow *(the blood forming organ)* which increase the risk of anemia, infection, or bleeding. If the bone marrow is depressed extensively, transfusions may be required to correct the problem. Escape of drug from the infection site may cause chronic ulceration of the skin or severe local reaction. It is also possible that changes may occur in the sperm of males which might produce birth defects in future children. Additional, more serious side effects which rarely occur include chest pain with some damage to the heart, loss of coordination or balance, or other manifestations of brain or nerve damage.

Cisplatin frequently causes loss of appetite, nausea, vomiting, hearing loss, loss of taste, damage to kidneys, and bone marrow suppression *(which can lead to anemia, infections, bruising or bleeding or, rarely acute leukemia)*. Other less common but serious side effects include numbness and tingling of fingers and toes and other neurological side effects, allergic reactions,

chemical abnormalities of the blood *(high uric acid or low magnesium)*, facial swelling, involuntary shaking, decreased vision, muscle cramps or spasms. There is a risk of leukemia when cisplatin is given with other anticancer drugs.

Other unforeseeable or unexpected risks may occur. It is unknown what effects this medication (treatment) may have on an unborn child. For this reason, I will be asked to practice an effective method of birth control while participating in this study. My doctor(s) will closely monitor my condition and in order for him/her to recognize and treat all these undesirable side effects early, repeated blood tests, x-rays and other history and physical examinations will be required.

As part of the evaluation of my therapy, I will permit my doctors, or their designated nurses to withdraw samples of my blood during the course of treatment. Possible side effects include minimal discomfort from needle punctures, possible hematoma *(black and blue marks)*, and rare instances of fainting. On the days blood is drawn, two or three samples may be required, each sample amounting to less than two teaspoons.

CONTACT PERSONS

In the event that injury occurs as a result of this result, treatment for injury will be available. I understand, however, I will not be provided with reimbursement for medical care nor will I receive other compensation. For more information concerning the research and research-related risks or injuries, I can notify Dr.______________________
the investigator in charge, at ______________________. In addition, I may contact _____________________________________
at ___________________ for information regarding patients' rights in research studies.

BENEFITS

It is not possible to predict whether or not any personal benefit will result from the use of the treatment program. Possible benefits are shrinkage of my tumor and an increase in my survival. I have been told that should my disease become worse, should side effects become very severe, should new scientific developments occur that indicate the treatment is not in my best interest, or should my physician feel that this treatment is no longer in my best interest, the treatment would be stopped. Further treatment would be discussed.

ALTERNATIVES

Alternatives which could be considered in my case include surgery alone or radiation therapy. An additional alternative is no further therapy, which would probably result in continued growth of my tumor. I understand that my doctor can provide detailed information about my disease and the benefits of the various treatments available. I have been told that I should feel free to discuss my disease and my prognosis with the doctor. The physician involved in my care will be available to answer any questions I have concerning this program. In addition, I understand that I am free to ask my physician any questions concerning this program that I wish in the future.

My physician will explain any procedures related solely to research which would not otherwise be necessary. Some of these procedures may result in added costs and some of these costs may not be covered by insurance. My doctor will discuss these with me.

VOLUNTARY PARTICIPATION

Participation in this study is voluntary. No compensation for participation will be given. I understand that I am free to withdraw my consent to participate in this treatment program at any time without prejudice to my subsequent care. Refusal to participate will involve no penalty, or loss of benefits. I am free to seek care from a physician of my choice at any time. If I withdraw from the study, I will continue to receive care. In the event of a research-related injury, I understand my participation has been voluntary.

CONFIDENTIALITY

I understand that records of my progress while on the study will be kept in a confidential form at this institution

and also in a computer file at the headquarters of the Radiation Therapy Oncology Group *(RTOG)*. Records for patients at ECOG institutions are kept in a confidential form at the ECOG institution and also in a computer file at the statistical headquarters of the Eastern Cooperative Oncology Group. The confidentiality of the central computer record is carefully guarded. During their required reviews, representatives of the Food and Drug Administration *(FDA)*, the National Cancer Institute *(NCI)*, qualified representatives of applicable drug manufacturers, and other groups or organizations that have a role in the conduct of this study may have access to medical records which contain my identity. However, no information by which I can be identified will be released or published. Histopathologic material, including tissue and/or slides, may be sent to a central office for review and research investigation associated with this protocol.

I have read all of the above, asked questions, received answers concerning areas I did not understand, and willingly give my consent to participate in this program. Upon signing this form I will receive a copy.

______________________________________ ____________________

Patient Signature *(or Legal Representative)* Date

______________________________________ ____________________

Witness Date

______________________________________ ____________________

Principal Investigator Date

APPENDIX II

KARNOFSKY PERFORMANCE SCALE

100	Normal; no complaints; no evidence of disease
90	Able to carry on normal activity; minor signs or symptoms of disease
80	Normal activity with effort; some sign or symptoms of disease
70	Cares for self; unable to carry on normal activity or do active work
60	Requires occasional assistance, but is able to care for most personal needs
50	Requires considerable assistance and frequent medical care
40	Disabled; requires special care and assistance
30	Severely disabled; hospitalization is indicated, although death not imminent
20	Very sick; hospitalization necessary; active support treatment is necessary
10	Moribund; fatal processes progressing rapidly
0	Dead

APPENDIX III

ESOPHAGUS , AJCC 4th Edition, 1992

DEFINITION OF TNM

Primary Tumor (T)

TX Primary tumor cannot be assessed
T0 No evidence of primary tumor
Tis Carcinoma *in situ*
T1 Tumor invades lamina propria or submucosa
T2 Tumor invades muscularis propria
T3 Tumor invades adventitia
T4 Tumor invades adjacent structures

Regional Lymph Nodes (N)

NX Regional lymph nodes cannot be assessed
N0 No regional lymph node metastasis
N1 Regional lymph node metastasis

Distant Metastasis (M)

MX Presence of distant metastasis cannot be assessed
M0 No distant metastais
M1 Distant metastasis

HISTOPATHOLOGIC TYPE

The staging classification applies to all carcinomas *(squamous cell and adeoncarcinomas)*. Adenocarcinomas that arise in Barrett's esophagus are also included in the classification.

HISTOPATHOLOGIC GRADE (G)

GX Grade cannot be assessed
G1 Well differentiated
G2 Moderately differentiated
G3 Poorly differentiated
G4 Undifferentiated

STAGE GROUPING

Stage 0	Tis	N0	M0
Stage I	T1	N0	M0
Stage IIA	T2	N0	M0
	T3	N0	M0
Stage IIB	T1	N0	M0
	T2	N1	M0
Stage III	T3	N1	M0
	T4	Any N	M0
Stage IV	Any T	Any N	M1

APPENDIX V

ADVERSE REACTION REPORTING GUIDELINES

A. GENERAL GUIDELINES

In order to assure prompt and complete reporting of toxicities, the following general guidelines are to be observed. These apply to all RTOG studies and Intergroup Studies in which RTOG participates. **When a protocol toxicity requires special handling, study-specific reporting procedures supercede the General Guidelines.**

1. The Principal Investigator will report the details of any unusual, significant, fatal or life-threatening protocol treatment reaction to the RTOG Group Chairman. In the absence of the Group Chairman, the report should be made to the Headquarters Data Management Staff *(215/574-3214)*. When telephone reporting is required, the Principal Investigator should have all relevant material available. See the protocol-specific criteria to grade the severity of the reaction.

2. The Principal Investigator will also report the details of the significant reaction to the Study Chairman by telephone .

3. A written report containing all relevant clinical information concerning the reported event will be sent to RTOG Headquarters by the Principal Investigator. This must sent within 10 working days of the discovery of the toxicity unless specified sooner by the protocol *(FAX #215/928-0153).*

4. The Group Chairman in consultation with the Study Chairman will take appropriate and prompt action to inform the membership and statistical personnel of any protocol modifications and/or precautionary measures.

5. For those incidents requiring telephone reporting to the National Cancer Institute *(NCI)*, Investigational Drug Branch *(IDB)* or Food and Drug Administration *(FDA)*, the Principal Investigator should first call RTOG *(as outlined above)* unless this will unduly delay the notification process required by the federal agencies.

A copy of all correspondence submitted to NCI, or to another Cooperative Group *(in the case of RTOG-coordinated intergroup studies)* must also be submitted to RTOG Headquarters when applicable.

6. The Principal Investigator, when participating in RTOG-coordinated Intergroup studies, is obligated to comply with all additional reporting specifications required by an individual study.

7. Institutions must also comply with their individual Institutional Review Board policy with regard to toxicity reporting procedure.

8. Failure to comply with reporting requirements in a timely manner may result in suspension of patient registration.

B. RADIATION TOXICITY GUIDELINES

1. All fatal toxicities *(grade 5)* resulting from protocol treatment must be reported by telephone to the Group Chairman, to RTOG Headquarters Data Management and to the primary Study Chairman within 24 hours of discovery.

2. All life-threatening *(grade 4)* toxicities resulting from protocol treatment must be reported by telephone to the Group Chairman, to RTOG Headquarters Data Management and to the primary Study Chairman within 24 hours of discovery.

3. Appropriate data forms, and if requested a written report, must be submitted to Headquarters within 10 working days of the telephone report.

C. ADVERSE DRUG REACTIONS - DRUG AND BIOLOGICS

An adverse reaction is a toxicity or an undesirable effect usually of severe nature. Specifically, this may include major organ toxicities of the liver, kidneys, cardiovascular system, central nervous system, skin, bone marrow, or anaphylaxis. These undesirable effects may be further classified as "known" or "unknown" toxicities.

Known toxicities are those which have been previously identified as having resulted from administration of the agent. They may be identified in the literature, the protocol, the consent form or noted in the drug insert.

Unknown toxicities are those thought to have resulted from the agent but have not previously been identified as a known side effect.

Commercial and Non-Investigational Agents

i. Any fatal *(grade 5)* or life threatening *(grade 4)* adverse reaction which is due to or suspected to be the result of a protocol drug must be reported to the Group Chairman or to RTOG Headquarters' Data Management Staff and to the Study Chairman by telephone within 24 hours of discovery. Known grade 4 hematologic toxicities need not be reported by telephone.

ii. Unknown adverse reactions *(≥ grade 2)* resulting from commercial drugs prescribed in an RTOG protocol are to be reported to the Group Chairman or RTOG Headquarters' Data Management, to the Study Chairman and to the IDB within 10 working days of discovery. FDA Form 3500 is to be used in reporting details. All relevant data forms must accompany the RTOG copy of Form 3500.

iii. All neurotoxicities *(≥ grade 3)* from radiosensitizer or protector drugs are to be reported within 24 hours by phone to RTOG Headquarters and to the Study Chairman.

iv. All relevant data forms must be submitted to RTOG Headquarters within 10 working days on all reactions requiring telephone reporting. A special written report may be required.

Reactions definitely thought not to be treatment related should not be reported, however, a report should be made of applicable effects if there is a reasonable suspicion that the effect is due to protocol treatment.

Investigational Agents

Prompt reporting of adverse reactions in patients treated with investigational agents is mandatory. Adverse reactions from NCI sponsored drugs are reported to:

Investigational Drug Branch *(IDB)*
P. O. Box 30012
Bethesda, MD 20824
Telephone number available 24 hours
(301) 230-2330 FAX # 301-230-0159

i. *Phase I Studies Utilizing Investigational Agents*

- All deaths during therapy with the agent.	Report **by phone** within 24 hours to IDB and RTOG Headquarters. **A written report to follow within 10 working days.
- All deaths within 30 days of termination of the agent.	As above
- All life threatening *(grade 4)* events which may be due to agent.	As above
- First occurrence of any toxicity *(regardless of grade).*	Report by **phone within 24 hours** to IDB drug monitor and RTOG Headquarters.

**A written report may be required.

ii. *Phase II, III Studies Utilizing Investigational Agents*

- All fatal *(grade 5)* and life threatening *(grade 4)* known adverse reactions due to investigational agent.	Report **by phone** to RTOG Headquarters and the Study Chairman within 24 hours<br>**A written report must be sent to RTOG within working days with a copy to IDB.<br>*(Grade 4 myelosuppression not reported to IDB)*
- All fatal (grade 5) and life threatening (grade 4) unknown adverse reactions resulting from or suspected to be related to investigational agent.	Report **by phone** to RTOG Headquarters, the Study Chairman and IDB within **24 hours**.<br>**A written report to follow within 10 working days.
- All grade 2, 3 unknown adverse reactions resulting from or suspected to be related to investigational agent.	**Report **in writing** to RTOG Headquarters and IDB within 10 working days.

** See attached *(if applicable to this study)* NCI Adverse Drug Reaction Reporting Form

APPENDIX VI

INPATIENT HYDRATION RECOMMEDATIONS

DAILY: creatinine, ,electrolytes x 3 days.

EVENING PRIOR TO CISPLATIN:

INPATIENTS

D5 1/2 NS + 20 MEQ KC/L @ 175 cc/hr. Minimum total in 12-18 hours: 2000 cc.
STRICT Intake and Output. Begin 12 hour urine collection for creatinine clearance.
Send specimen STAT to Biochemistry in A.M. Label specimen "PRE-CHEMO".

OUTPATIENTS

2 liters of fluid p.o.

ANTIEMETICS:

DECADRON 20 mg IVPB 20 min prior to chemo. over 5 min x 1 dose.
ONDANESETRON 10-20 mg IVPB 20 min prior to chemo
BENADRYL 50 mg IV q3h PRN-for dystonic reactions.

FIRST 6 hrs AFTER Cisplatin

IVFs D51/2 NS with 20 KCL/L @ 250cc/hr
20% MANNITOL @ 50cc/hr x 6 hrs *(10 gm/hr)*
Monitor I&Os Hourly; Replacement to begin 1 hour post CDDP administration.
If output *(urine, diarrhea, emesis)* exceeds 200 cc/hr ,replace this excess cc/cc in addition to the maintenance IVFs.
Using the same fluid for replacement.

FROM 6 to 24 hrs AFTER Cisplatin

IVFs D51/2 NS with 20 meq KCL/L @ 150cc/hr
Monitor I&Os q 2 hrs. If output *(urine, diarrhea, emesis)* exceeds 150 cc/hr, replace this excess cc/cc in addtion to maintainance IVFs. Using the same fluid for replacement.
If urine output drops below 150 cc/hr during this period, increase IVF rate to 250cc/hr for 2 hours.
If no change notify MD/NP/PA.

FROM 24 to 48 hrs AFTER Cisplatin

IVFs D51/2 NS with 20 meq KCL @ 150 cc/hr
Draw creatinine, lytes, Mg daily for 2 days
Monitor strict I&Os

APPENDIX VII

INTERGROUP PARTICIPATION IN RTOG STUDIES

GENERAL GUIDELINES

I. REGISTRATION: RTOG will be responsible for all registration/ randomizations. The procedure is:
-Each institution affiliated with a Cooperative Group will phone their group and supply the eligibility check information.
-The participating Cooperative Group will then telephone RTOG 215/574-3191 between 8:30 a.m. and 5:00 p.m. ET and supply the necessary eligibility and stratification information. RTOG will then assign a case number and treatment assignment. The participating Cooperative Group will then inform its member institution.
-RTOG will send a Confirmation of Registration and a Forms Due Calendar to the participating Cooperative Group for each case registered. The participating Group forwards a copy of the calendar to the participating institution.

II. PROTOCOL DISTRIBUTION: Each participating cooperative group is responsible for distribution of the protocol to its members. All protocol amendments will be sent by RTOG to each participating Group office for distribution to member institutions. All communication with NCI regarding this protocol will be routed through the RTOG.

III. INSTITUTIONAL PARTICIPATION: It is the responsibility of each participating Cooperative Group to decide which of its member institutions may participate in this protocol. Each participating Cooperative Group must ensure that IRB approval was obtained prior to accession of cases.

IV. CONFIRMATION/CALENDARS: A Confirmation of Registration notice and a Data Collection Calendar is produced for each case registered and/or randomized. These will be distributed by RTOG to the appropriate cooperative group office for distribution to their members, if appropriate.

The form identification code, which appears on the Calendars in the "key" columns, is found on the form in the lower right corner.

You are expected to respond to each of the items listed either by submitting the item, by notifying us in writing that the item is not available or that the assessment was not done. The calendar may also list items that are not forms *(CT or MRI scan reports, pathology reports)* but are specific source documents. These items will be noted in the data collection section of the protocol but will not be listed on the Forms Package Index.

Additional items/forms may be required depending on events that occur e.g. if surgery was done a surgical report may be required. See the protocol for conditional requirements.

Unless specified otherwise, all patients are followed until death or termination of the study.

V. FORMS: Other groups will attach a forms appendix to their members' version. It will be the responsibility of the other group's member to copy the attached forms and to maintain a supply of available forms for data submission.

The Demographic Data Form **(A5)** is required on all RTOG enrollments. This form is ideally completed by the patient. Instructions are found on the form.
The RTOG assigned case and study number must be recorded on all data items submitted. Except for material which requires rapid review *(see below)*, data should be routed according to the mechanism set up by the participating Group. Generally the participating group will require forms to be routed through their office and they will send the forms to:

American College of Radiology
Radiation Therapy Oncology Group - 14th Floor

1101 Market Street
Philadelphia, PA 19107

VI. LABELS: Patient specific labels will be supplied to the participating Group for distribution to the individual institutions as patients are registered at RTOG.

When completing the labels, be specific when describing films, e.g.: "Pre op CT Brain Scan, "Large Photon Localization Film", "Follow-up Bone Scan", etc.

Research associates are advised to consult technical staff for assistance when labeling radiotherapy films. Correct film identification is the responsibility of the institutions and is essential to maintain efficient data flow.

VII. CANCELLATION/INELIGIBILITY: Patients who are found to be ineligible subsequent to registration are to be followed according to plan unless you receive written instructions to the contrary.

Patients who receive no treatment whatsoever may be canceled, however, written notification and an explanation must be received at RTOG Headquarters as soon as this has been determined. We must receive this notification not later than two weeks after registration. We will notify you of the determination made regarding the status of the case and instructions regarding subsequent data submission. RTOG requires all patients in randomized trials to be followed with data submission according to protocol schedule.

VI. RAPID REVIEW ITEMS: Time critical data which require rapid submission must be sent directly to RTOG. These items are:

T2 - Protocol Treatment Form
T3 - Photon Localization film *(for all fields treated initially)*
T4 - Photon dose calculations *(for all fields treated initially)*

IX. REQUEST FOR STUDY INFORMATION AND FORMS REQUEST: Requests for additional information or clarification of data will be routed through the participating Cooperative Group office for distribution to the individual institution.

The memo requesting the additional information must be returned with the response. Responses should be returned according to the procedure used to submit data forms. You may receive reminders prompting response.

Periodically *(generally three times per year)* computer-generated lists identifying delinquent material are prepared. These are routed by RTOG through the participating group for distribution.

X. QUESTIONS REGARDING:

**Data/Eligibility/Treatment/ Adverse Events/Data Management Procedures**	RTOG Research Associate *(215)* 574-3214
**Forms Packets *(RTOG Members)***	Registration Secretary *(215)* 574-3191
**Pathology**	Pathology Clerk *(801)* 321-1929<br>*(unless specified otherwise in Section 10.0)*
**Protocols/Amendments**	Director, Protocol Development *(215)* 574-3195
**Radiotherapy data items *(films, radiographs, isodose summations, treatment records, scans, reports and calculations)***	Dosimetry Clerk *(215)* 574-3219

Randomization/Registration Registration Secretary (215) 574-3191

If you are unable to reach the person noted, and your call is urgent, ask to speak to any HQ Research Associate.

XI. ADVERSE EVENTS AND TOXICITY

From Radiotherapy: Unusual toxicities, all grade 5 toxicities, and grade 4 toxicities in altered fractionation studies are reported by telephone within 24 hours of discovery to RTOG Headquarters, to the Group Chairman Dr. Walter Curran, to the Study Chair*(s)*, and to the RTOG Research Associate for this study.

From Investigational Agents: Are to be reported according to NCI guidelines. In addition, RTOG Headquarters, RTOG Data Management and the Study Chair*(s)* are to receive notification as outlined by the NCI procedures. If telephone notification is necessary, RTOG and the Study Chair*(s)* must also be called.

Copies of all toxicity reports and forms submitted to NCI must be sent to RTOG Headquarters also.

From Commercial Drugs: Are to be reported according to NCI/FDA guidelines. A copy of the reports and forms submitted to FDA must be sent to RTOG.

Data Submission: Events that require telephone reporting will require current updating of data forms through the date of the event. Submit within 10 working days of the telephone call.

Second Malignancy: All second primary tumors that are diagnosed during or following protocol treatment must be reported on the study data collection forms. AML/MDS must be reported on the NCI/CTEP Secondary Reporting Form. Instructions for submission are on the data form.
