# Supplementary material for: Association of Radiotherapy Duration With Clinical Outcomes in Patients With Esophageal Cancer Treated in NRG Oncology Trials: A Secondary Analysis of NRG Oncology Randomized Clinical Trials
Source: JAMA Netw Open. 2023 Apr 21;6(4):e238504. doi: 10.1001/jamanetworkopen.2023.8504 (PMC10122174; doi:10.1001/jamanetworkopen.2023.8504)
Supplement: Supplement 4. — eTable 1. Characteristics by RT Duration ≤45 Days vs >45 Days eTable 2. Characteristics by RT Duration ≤39 Days vs >39 Days eTable 3. Characteristics by RT Interruptions eTable 4. RT Duration ≤45 Days vs >45 Days, Controlling for NRG Oncology Trial eTable 5. RT Duration ≤45 Days vs >45 Days in Patients with Squamous Cell Carcinoma Histology eTable 6. RT Duration ≤45 Days vs >45 Days in Patients with Adenocarcinoma Histology eTable 7. RT Duration (Continuous), Controlling for NRG Oncology Trial eTable 8. RT Duration ≤39 Days vs >39 Days, Controlling for NRG Oncology Trial eTable 9. Multivariable Models: RT Duration (≤ 39 Days vs > 39 Days) eTable 10. RT Interruptions, Controlling for NRG Oncology Trial eFigure 1. Outcomes for RT Duration Dichotomized by XTile Cutpoint eFigure 2. Outcomes for RT Duration Dichotomized by Median eFigure 3. Outcomes for RT Interruption [file jamanetwopen-e238504-s004.pdf]

Supplemental Online Content

Hallemeier CL, Moughan J, Haddock MG, et al. Association of radiotherapy duration with clinical outcomes in patients with esophageal cancer treated in NRG oncology trials: a secondary analysis of NRG Oncology Randomized Clinical Trials. *JAMA Netw Open*. 2023;6(4):e238504. doi:10.1001/jamanetworkopen.2023.8504

eTable 1. Characteristics by RT Duration ≤45 Days vs >45 Days

eTable 2. Characteristics by RT Duration ≤39 Days vs >39 Days

eTable 3. Characteristics by RT Interruptions

eTable 4. RT Duration ≤45 Days vs >45 Days, Controlling for NRG Oncology Trial

eTable 5. RT Duration ≤45 Days vs >45 Days in Patients with Squamous Cell Carcinoma Histology

eTable 6. RT Duration ≤45 Days vs >45 Days in Patients with Adenocarcinoma Histology

eTable 7. RT Duration (Continuous), Controlling for NRG Oncology Trial

eTable 8. RT Duration ≤39 Days vs >39 Days, Controlling for NRG Oncology Trial

eTable 9. Multivariable Models: RT Duration (≤ 39 Days vs. > 39 Days)

eTable 10. RT Interruptions, Controlling for NRG Oncology Trial

eFigure 1. Outcomes for RT Duration Dichotomized by XTile Cutpoint

eFigure 2. Outcomes for RT Duration Dichotomized by Median

eFigure 3. Outcomes for RT Interruption

This supplemental material has been provided by the authors to give readers additional information about their work.

© 2023 Hallemeier CL et al. *JAMA Network Open.*

eTable 1: Characteristics by RT Duration ≤45 days vs >45 days

Pretreatment, Tumor, or Treatment Characteristics	≤45 days (n=446)	>45 days (n=63)	p-value[a]
Age (years)			
Median (IQR)	64 (57-70)	62 (54-69)	
Min-Max	38-87	32-83	
Sex			0.02
Male	373 (84%)	45 (71%)	
Female	73 (16%)	18 (29%)	
Race			0.05
Non-white	110 (25%)	23 (37%)	
White	336 (75%)	40 (63%)	
Zubrod			0.23
0	227 (51%)	27 (43%)	
1 or 2	219 (49%)	36 (57%)	
Histology			0.97
Adenocarcinoma	190 (43%)	27 (43%)	
Squamous	256 (57%)	36 (57%)	
Tumor Size			0.02
< 5 cm	172 (39%)	15 (24%)	
≥ 5 cm	274 (61%)	48 (76%)	
T Stage			0.47
T1/T2	163 (37%)	26 (41%)	
T3/T4/Unknown	283 (63%)	37 (59%)	
N Stage			0.34
N0	234 (52%)	29 (46%)	
N1/NX	212 (48%)	34 (54%)	
≥80% of Protocol-Specified Concurrent Chemo			0.02
No	32 (7%)	10 (16%)	
Yes	414 (93%)	53 (84%)	
Trial			0.87
NRG/RTOG 0436	251 (56%)	36 (57%)	
NRG/RTOG 8501	104 (23%)	13 (21%)	
NRG/RTOG 9405	91 (20%)	14 (22%)	

Data are n (%) unless otherwise indicated.
Abbreviations: RT, radiation therapy; IQR, interquartile range; min, minimum; max, maximum; NRG/RTOG, NRG Oncology/Radiation Therapy Oncology Group
[a]p-value from Chi-square/Fisher's Exact Test

© 2023 Hallemeier CL et al. *JAMA Network Open.*

eTable 2: Characteristics by RT Duration ≤39 days vs >39 days

Pretreatment, Tumor, or Treatment Characteristics	≤39 days (n=271)	>39 days (n=238)	p-value[a]
Age (years)			
Median (IQR)	64 (58-70)	64 (56-70)	
Min-Max	38-87	32-83	
Sex			0.08
Male	230 (85%)	188 (79%)	
Female	41 (15%)	50 (21%)	
Race			0.003
Non-white	56 (21%)	77 (32%)	
White	215 (79%)	161 (68%)	
Zubrod			0.17
0	143 (53%)	111 (47%)	
1 or 2	128 (47%)	127 (53%)	
Histology			0.53
Adenocarcinoma	119 (44%)	98 (41%)	
Squamous	152 (56%)	140 (59%)	
Tumor Size			0.17
< 5 cm	107 (39%)	80 (34%)	
≥ 5 cm	164 (61%)	158 (66%)	
T Stage			0.80
T1/T2	102 (38%)	87 (37%)	
T3/T4/Unknown	169 (62%)	151 (63%)	
N Stage			0.38
N0	145 (54%)	118 (50%)	
N1/NX	126 (46%)	120 (50%)	
≥80% of Protocol-Specified Concurrent Chemo			0.04
No	16 (6%)	26 (11%)	
Yes	255 (94%)	212 (89%)	
Trial			0.24
NRG/RTOG 0436	152 (56%)	135 (57%)	
NRG/RTOG 8501	69 (25%)	48 (20%)	
NRG/RTOG 9405	50 (18%)	55 (23%)	

Data are n (%) unless otherwise indicated.
Abbreviations: RT, radiation therapy; IQR, interquartile range; min, minimum; max, maximum; NRG/RTOG, NRG Oncology/Radiation Therapy Oncology Group
[a]p-value from Chi-square/Fisher's Exact Test

© 2023 Hallemeier CL et al. *JAMA Network Open.*

eTable 3: Characteristics by RT Interruptions

Pretreatment, Tumor, or Treatment Characteristics	No (n=302)	Yes (n=207)	p-value[a]
Age (years)			
Median (IQR)	64 (57-70)	64 (57-70)	
Min-Max	38-87	32-83	
Sex			0.06
Male	256 (85%)	162 (78%)	
Female	46 (15%)	45 (22%)	
Race			0.04
Non-white	69 (23%)	64 (31%)	
White	233 (77%)	143 (69%)	
Zubrod			0.02
0	164 (54%)	90 (43%)	
1 or 2	138 (46%)	117 (57%)	
Histology			0.82
Adenocarcinoma	130 (43%)	87 (42%)	
Squamous	172 (57%)	120 (58%)	
Tumor Size			0.01
< 5 cm	124 (41%)	63 (30%)	
≥ 5 cm	178 (59%)	144 (70%)	
T Stage			0.83
T1/T2	111 (37%)	78 (38%)	
T3/T4/Unknown	191 (63%)	129 (62%)	
N Stage			0.15
N0	164 (54%)	99 (48%)	
N1/NX	138 (46%)	108 (52%)	
≥80% of Protocol-Specified Concurrent Chemo			0.53
No	23 (8%)	19 (9%)	
Yes	279 (92%)	188 (91%)	
Trial			0.21
NRG/RTOG 0436	170 (56%)	117 (57%)	
NRG/RTOG 8501	76 (25%)	41 (20%)	
NRG/RTOG 9405	56 (19%)	49 (24%)	

Data are n (%) unless otherwise indicated.
Abbreviations: RT, radiation therapy; IQR, interquartile range; min, minimum; max, maximum; NRG/RTOG, NRG Oncology/Radiation Therapy Oncology Group
[a]p-value from Chi-square/Fisher's Exact Test

© 2023 Hallemeier CL et al. *JAMA Network Open.*

eTable 4: RT Duration ≤45 days vs >45 Days, Controlling for NRG Oncology Trial

Endpoint	Variable	Comparison	n	Events	HR (95% CI)	p-value[a]
OS	RT Duration	≤ 45 days	446	354	1 (ref)	..
		> 45 days	63	55	1.32 (0.99-1.76)	0.05
	Trial	NRG/RTOG 0436	287	206	1 (ref)	..
		NRG/RTOG 8501	117	110	1.07 (0.84-1.36)	0.61
		NRG/RTOG 9405	105	93	0.91 (0.71-1.18)	0.49
DFS	RT Duration	≤ 45 days	446	386	1 (ref)	..
		> 45 days	63	59	1.41 (1.07-1.86)	0.01
	Trial	NRG/RTOG 0436	287	239	1 (ref)	..
		NRG/RTOG 8501	117	110	0.84 (0.66-1.06)	0.14
		NRG/RTOG 9405	105	96	0.89 (0.70-1.14)	0.37
LRF	RT Duration	≤ 45 days	446	247	1 (ref)	..
		> 45 days	63	41	1.38 (0.99-1.90)	0.05
	Trial	NRG/RTOG 0436	287	157	1 (ref)	..
		NRG/RTOG 8501	117	66	1.03 (0.79-1.35)	0.83
		NRG/RTOG 9405	105	65	1.22 (0.92-1.61)	0.16
DF	RT Duration	≤ 45 days	446	195	1 (ref)	..
		> 45 days	63	24	0.87 (0.56-1.35)	0.54
	Trial	NRG/RTOG 0436	287	132	1 (ref)	..
		NRG/RTOG 8501	117	43	0.70 (0.50-0.98)	0.04
		NRG/RTOG 9405	105	44	0.84 (0.60-1.19)	0.33

Abbreviations: OS, overall survival; DFS, disease-free survival; LRF, local-regional failure; DF, distant failure; HR, hazard ratio; CI, confidence interval; ref, reference level; RT, radiation therapy; NRG/RTOG, NRG Oncology/Radiation Therapy Oncology Group
[a]p-value from the Cox proportional hazards model (OS/DFS) or the Fine-Gray regression model (LRF/DF).

© 2023 Hallemeier CL et al. *JAMA Network Open.*

eTable 5: RT Duration ≤45 days vs >45 Days in Patients with Squamous Cell Carcinoma Histology (n=292)

	OS		DFS		LRF		DF	
	3y-% (95% CI)	6y-% (95% CI)	3y-% (95% CI)	6y-% (95% CI)	3y-% (95% CI)	6y-% (95% CI)	3y-% (95% CI)	6y-% (95% CI)
≤ 45 days (n=256)	39 (33,45)	23 (17,29)	30 (24,35)	19 (14,24)	52 (45,58)	54 (47,60)	35 (30,41)	37 (31,43)
> 45 days (n=36)	22 (10,37)	16 (6,30)	14 (5,27)	11 (4,24)	68 (49,81)	68 (49,81)	25 (12,40)	28 (14,43)
p-value	0.08[a]		0.03[a]		0.04[b]		0.29[b]	

Abbreviations: OS, overall survival; DFS, disease-free survival; LRF, local-regional failure; DF, distant failure; y, year; CI, confidence interval
[a]p-value from log-rank test
[b]p-value from Gray's test

© 2023 Hallemeier CL et al. *JAMA Network Open.*

eTable 6: RT Duration ≤45 days vs >45 Days in Patients with Adenocarcinoma Histology (n=217)

	OS		DFS		LRF		DF	
	3y-% (95% CI)	6y-% (95% CI)	3y-% (95% CI)	6y-% (95% CI)	3y-% (95% CI)	6y-% (95% CI)	3y-% (95% CI)	6y-% (95% CI)
≤ 45 days (n=190)	29 (22,35)	14 (7,23)	15 (11,21)	7 (3,15)	57 (50,64)	58 (51,65)	50 (43,57)	55 (47,62)
> 45 days (n=27)	17 (6,34)	9 (1,28)	7 (1,21)	7 (1,21)	63 (41,79)	63 (41,79)	52 (31,69)	52 (31,69)
p-value	0.42[a]		0.45[a]		0.51[b]		0.98[b]	

Abbreviations: OS, overall survival; DFS, disease-free survival; LRF, local-regional failure; DF, distant failure; y, year; CI, confidence interval

[a]p-value from log-rank test

[b]p-value from Gray's test

© 2023 Hallemeier CL et al. *JAMA Network Open.*

eTable 7. RT Duration (Continuous), Controlling for NRG Oncology Trial

Endpoint	Variable	Comparison	n	Events	HR (95% CI)	p-value[a]
OS	RT Duration	(continuous; unit increase=7 days)	509	409	1.10 (0.98-1.23)	0.10
	Trial	NRG/RTOG 0436	287	206	1 (ref)	..
		NRG/RTOG 8501	117	110	1.08 (0.85-1.38)	0.53
		NRG/RTOG 9405	105	93	0.92 (0.71-1.19)	0.52
DFS	RT Duration	(continuous; unit increase=7 days)	509	445	1.17 (1.04-1.32)	0.009
	Trial	NRG/RTOG 0436	287	239	1 (ref)	..
		NRG/RTOG 8501	117	110	0.87 (0.69-1.10)	0.25
		NRG/RTOG 9405	105	96	0.90 (0.71-1.16)	0.42
LRF	RT Duration	(continuous; unit increase=7 days)	509	288	1.17 (1.04 -1.31)	0.01
	Trial	NRG/RTOG 0436	287	157	1 (ref)	..
		NRG/RTOG 8501	117	66	1.06 (0.81-1.39)	0.66
		NRG/RTOG 9405	105	65	1.22 (0.93-1.62)	0.15
DF	RT Duration	(continuous; unit increase=7 days)	509	219	0.88 (0.74-1.05)	0.15
	Trial	NRG/RTOG 0436	287	132	1 (ref)	..
		NRG/RTOG 8501	117	43	0.68 (0.48-0.95)	0.02
		NRG/RTOG 9405	105	44	0.84 (0.60-1.18)	0.32

Abbreviations: OS, overall survival; DFS, disease-free survival; LRF, local-regional failure; DF, distant failure; HR, hazard ratio; CI, confidence interval; ref, reference level; RT, radiation therapy; NRG/RTOG, NRG Oncology/Radiation Therapy Oncology Group
[a]p-value from the Cox proportional hazards model (OS/DFS) or the Fine-Gray regression model (LRF/DF).

© 2023 Hallemeier CL et al. *JAMA Network Open.*

eTable 8: RT Duration ≤39 days vs >39 Days, Controlling for NRG Oncology Trial

Endpoint	Variable	Comparison	n	Events	HR (95% CI)	p-value[a]
OS	RT Duration	≤ 39 days	271	215	1 (ref)	..
		> 39 days	238	194	1.07 (0.88-1.30)	0.51
	Trial	NRG/RTOG 0436	287	206	1 (ref)	..
		NRG/RTOG 8501	117	110	1.08 (0.84-1.37)	0.56
		NRG/RTOG 9405	105	93	0.92 (0.71-1.18)	0.50
DFS	RT Duration	≤ 39 days	271	231	1 (ref)	..
		> 39 days	238	214	1.21 (1.00-1.46)	0.05
	Trial	NRG/RTOG 0436	287	239	1 (ref)	..
		NRG/RTOG 8501	117	110	0.84 (0.67-1.07)	0.16
		NRG/RTOG 9405	105	96	0.89 (0.70-1.14)	0.35
LRF	RT Duration	≤ 39 days	271	139	1 (ref)	..
		> 39 days	238	149	1.37 (1.10-1.71)	0.004
	Trial	NRG/RTOG 0436	287	157	1 (ref)	..
		NRG/RTOG 8501	117	66	1.04 (0.80-1.36)	0.77
		NRG/RTOG 9405	105	65	1.20 (0.91-1.59)	0.19
DF	RT Duration	≤ 39 days	271	124	1 (ref)	..
		> 39 days	238	95	0.85 (0.65-1.12)	0.25
	Trial	NRG/RTOG 0436	287	132	1 (ref)	..
		NRG/RTOG 8501	117	43	0.69 (0.49-0.97)	0.03
		NRG/RTOG 9405	105	44	0.86 (0.61-1.21)	0.37

Abbreviations: OS, overall survival; DFS, disease-free survival; LRF, local-regional failure; DF, distant failure; HR, hazard ratio; CI, confidence interval; ref, reference level; RT, radiation therapy; NRG/RTOG, NRG Oncology/Radiation Therapy Oncology Group
[a]p-value from the Cox proportional hazards model (OS/DFS) or the Fine-Gray regression model (LRF/DF).

© 2023 Hallemeier CL et al. *JAMA Network Open.*

eTable 9: Multivariable Models: RT Duration (≤ 39 days vs. > 39 days) (n=509)

	OS (deaths = 409)		DFS (failures = 445)		LRF (failures = 288)		DF (failures = 219)	
**Variables[a]**	**HR (95% CI)**	**p-value[b]**	**HR (95% CI)**	**p-value[b]**	**HR (95% CI)**	**p-value[b]**	**HR (95% CI)**	**p-value[b]**
RT Duration								
≤ 39 days	1 (ref)	..	1 (ref)	..	1 (ref)	..	1 (ref)	..
> 39 days	1.04 (0.85-1.26)	0.70	1.18 (0.98-1.43)	0.08	1.32 (1.06-1.65)	0.01	0.83 (0.64-1.09)	0.19
Trial								
NRG/RTOG 0436	1 (ref)	..	1 (ref)	..	1 (ref)	..	1 (ref)	..
NRG/RTOG 8501	1.11 (0.84-1.45)	0.46	1.06 (0.80-1.40)	0.70	1.20 (0.90-1.61)	0.22	0.78 (0.53-1.14)	0.20
NRG/RTOG 9405	0.96 (0.73-1.25)	0.74	1.07 (0.81-1.41)	0.62	1.32 (0.99-1.77)	0.06	1.01 (0.70-1.47)	0.95
Age (continuous; unit increase=10)	---	---	---	---	0.86 (0.77-0.97)	0.01	0.84 (0.73-0.97)	0.02
Sex								
Female	1 (ref)	..	---	---	---	---	---	---
Male	1.49 (1.13-1.95)	0.004	---	---	---	---	---	---
Race								
Other	---	---	---	---	---	---	---	---
White	---	---	---	---	---	---	---	---
Zubrod								
0	1 (ref)	..	1 (ref)	..	1 (ref)	..	---	---
1 or 2	1.26 (1.04-1.53)	0.02	1.27 (1.05-1.54)	0.01	1.30 (1.04-1.61)	0.02	---	---
Tumor size (cm)								
< 5	1 (ref)	..	1 (ref)	..	---	---	1 (ref)	..
≥ 5	1.44 (1.16-1.79)	0.001	1.32 (1.08-1.62)	0.008	---	---	1.35 (1.01-1.79)	0.04
N Stage								
N0	1 (ref)	..	1 (ref)	..	1 (ref)	..	---	---
NX/N1	1.37 (1.10-1.70)	0.005	1.37 (1.11-1.69)	0.004	1.37 (1.08-1.74)	0.01	---	---
Histology								
Adenocarcinoma	---	---	1 (ref)	..	---	---	1 (ref)	..
Squamous	---	---	0.72 (0.58-0.89)	0.003	---	---	0.60 (0.45-0.81)	<0.001
≥80% of Protocol-Specified Concurrent Chemo								
No	---	---	1 (ref)	..	---	---	---	---
Yes	---	---	0.66 (0.47-0.92)	0.01	---	---	---	---

Abbreviations: OS, overall survival; DFS, disease-free survival; LRF, local-regional failure; DF, distant failure; HR, hazard ratio; CI, confidence interval; ref, reference level; RT, radiation therapy; NRG/RTOG, NRG Oncology/Radiation Therapy Oncology Group; Chemo, chemotherapy

© 2023 Hallemeier CL et al. *JAMA Network Open.*

[a]RT duration and NRG Oncology trial are included in each model and other variables are included if p-value <0.05.
[b]p-value from the Cox proportional hazards model (OS/DFS) or the Fine-Gray regression model (LRF/DF).

© 2023 Hallemeier CL et al. *JAMA Network Open.*

eTable 10: RT Interruptions, Controlling for NRG Oncology Trial

Endpoint	Variable	Comparison	n	Events	HR (95% CI)	p-value[a]
OS	RT Interruptions	No	302	240	1 (ref)	..
		Yes	207	169	1.15 (0.94-1.40)	0.16
	Trial	NRG/RTOG 0436	287	206	1 (ref)	..
		NRG/RTOG 8501	117	110	1.08 (0.84-1.37)	0.55
		NRG/RTOG 9405	105	93	0.91 (0.71-1.18)	0.47
DFS	RT Interruptions	No	302	259	1 (ref)	..
		Yes	207	186	1.19 (0.99 -1.44)	0.07
	Trial	NRG/RTOG 0436	287	239	1 (ref)	..
		NRG/RTOG 8501	117	110	0.85 (0.67-1.07)	0.16
		NRG/RTOG 9405	105	96	0.89 (0.69-1.14)	0.34
LRF	RT Interruptions	No	302	162	1 (ref)	..
		Yes	207	126	1.22 (0.98-1.52)	0.08
	Trial	NRG/RTOG 0436	287	157	1 (ref)	..
		NRG/RTOG 8501	117	66	1.04 (0.79-1.36)	0.79
		NRG/RTOG 9405	105	65	1.21 (0.92-1.60)	0.17
DF	RT Interruptions	No	302	128	1 (ref)	..
		Yes	207	91	1.04 (0.80-1.37)	0.75
	Trial	NRG/RTOG 0436	287	132	1 (ref)	..
		NRG/RTOG 8501	117	43	0.70 (0.50-0.98)	0.04
		NRG/RTOG 9405	105	44	0.84 (0.60-1.18)	0.32

Abbreviations: OS, overall survival; DFS, disease-free survival; LRF, local-regional failure; DF, distant failure; HR, hazard ratio; CI, confidence interval; ref, reference level; RT, radiation therapy; NRG/RTOG, NRG Oncology/Radiation Therapy Oncology Group
[a]p-value from the Cox proportional hazards model (OS/DFS) or the Fine-Gray regression model (LRF/DF).

© 2023 Hallemeier CL et al. *JAMA Network Open.*

eFigure 1. Outcomes for RT duration dichotomized by XTile cutpoint

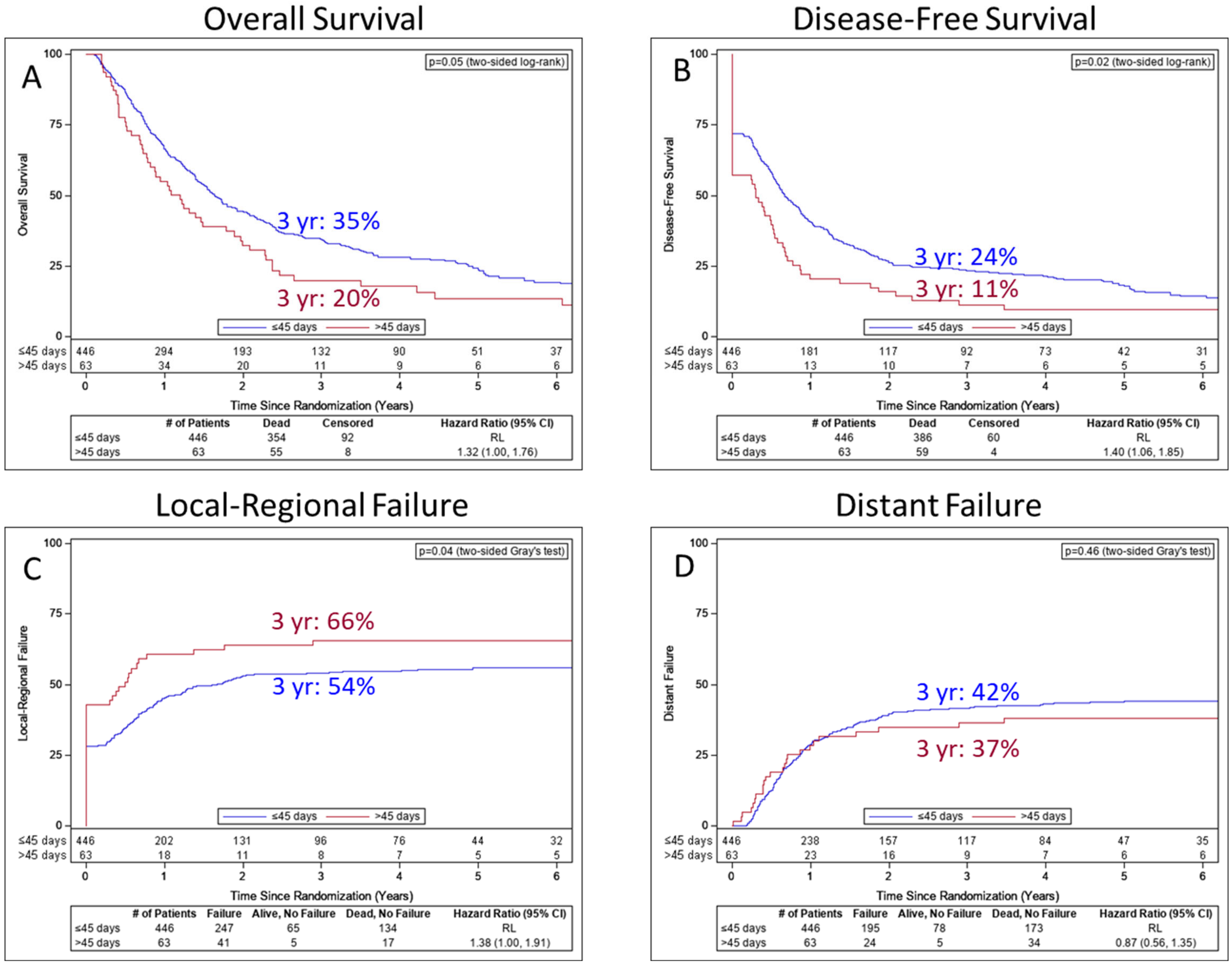

© 2023 Hallemeier CL et al. *JAMA Network Open.*

eFigure 2. Outcomes for RT duration dichotomized by median

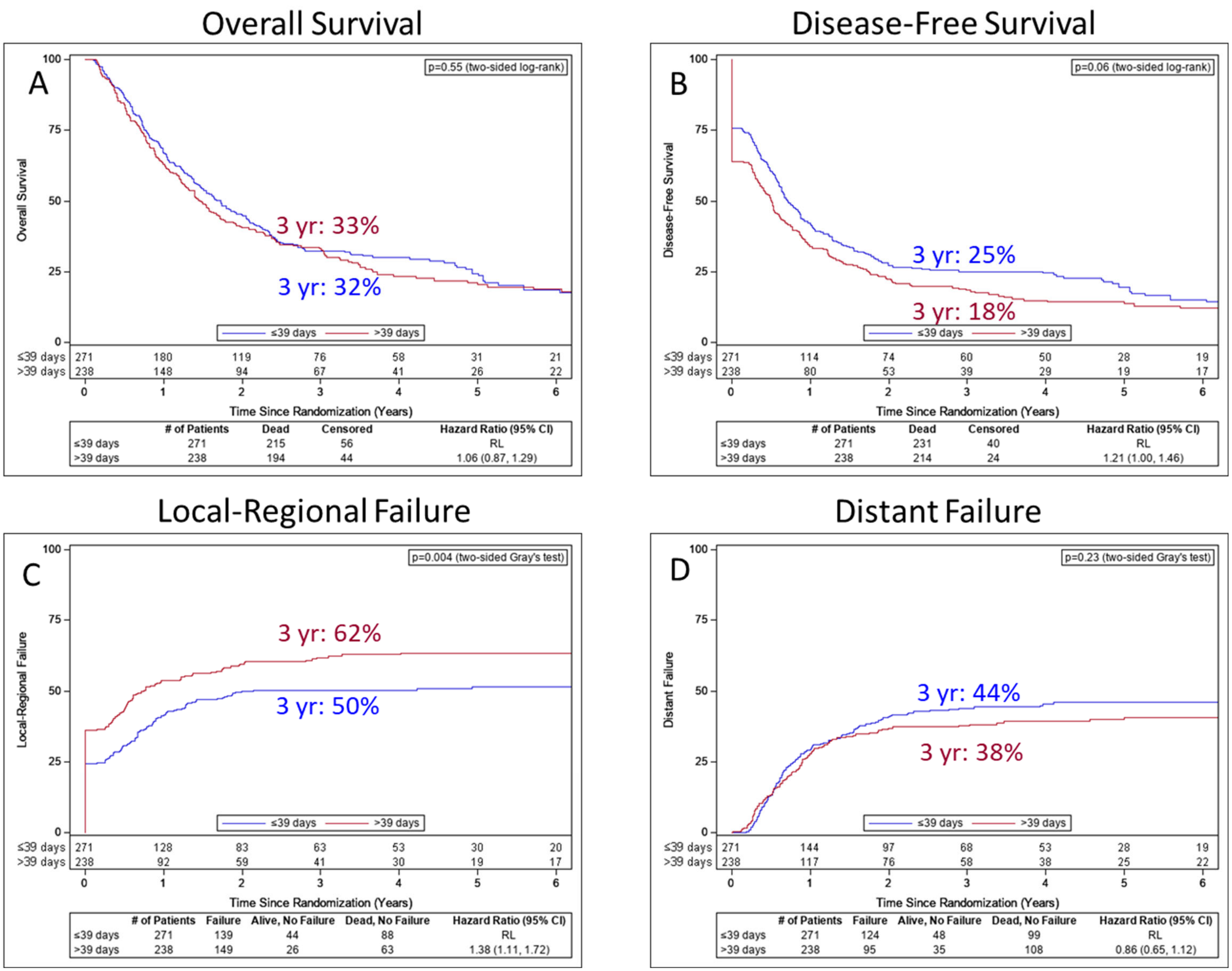

© 2023 Hallemeier CL et al. *JAMA Network Open.*

eFigure 3. Outcomes for RT interruption

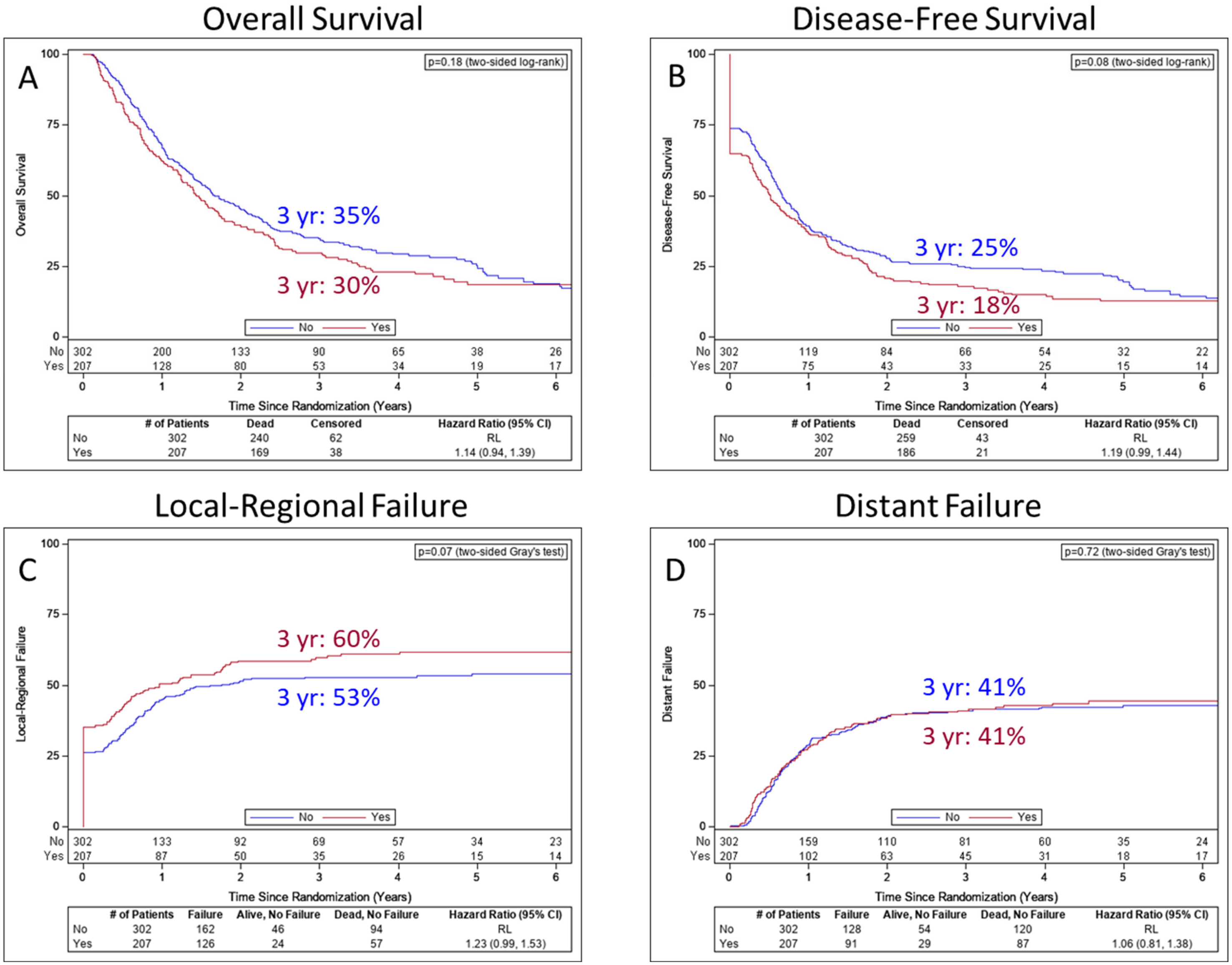

© 2023 Hallemeier CL et al. *JAMA Network Open.*
