# Supplementary material for: Association of Radiotherapy Duration With Clinical Outcomes in Patients With Esophageal Cancer Treated in NRG Oncology Trials: A Secondary Analysis of NRG Oncology Randomized Clinical Trials
Source: JAMA Netw Open. 2023 Apr 21;6(4):e238504. doi: 10.1001/jamanetworkopen.2023.8504 (PMC10122174; doi:10.1001/jamanetworkopen.2023.8504)
Supplement: Supplement 5. — Data Sharing Statement [file jamanetwopen-e238504-s005.pdf]

Data Sharing Statement

Hallemeier. Association of Radiotherapy Duration With Clinical Outcomes in Patients With Esophageal Cancer Treated in NRG Oncology Trials. *JAMA Netw Open*. Published April 21, 2023. doi:10.1001/jamanetworkopen.2023.8504

Data
Data available: No

Additional Information
Explanation for why data not available: Data will not be published but would be available on request.
